# A global phylogeny of Pelomedusoides turtles with new material of *Neochelys franzeni* Schleich, 1993 (Testudines, Podocnemididae) from the middle Eocene, Messel Pit, of Germany

**DOI:** 10.7717/peerj.1221

**Published:** 2015-08-27

**Authors:** Edwin Cadena

**Affiliations:** Alexander von Humboldt Foundation, Senckenberg Naturmuseum, Frankfurt am Main, Germany

**Keywords:** Podocnemididae, Eocene, Germany, Messel Pit, Pleurodira, Phylogeny

## Abstract

**Background.** *Neochelys franzeni* Schleich, 1993 is the only pleurodire or side-necked turtle from the middle Eocene, Messel Pit (the first UNESCO, World Natural Heritage Site in Germany, since 1995). The original description of the species is based on two specimens SMF ME 1091 (Holotype) and 715 (Paratype) housed at the Senckenberg Museum Frankfurt. The excellent preservation of complete and articulated skeletons of this species makes it a key taxon for understanding the evolution and phylogeny of the European *Neochelys* genus and its relationships with South American and African-Madagascar podocnemidids.

**Results.** Five new specimens of *Neochelys franzeni* from Messel Pit are described here, together with the redescription of SMF ME 1091 and 715. Specimens correspond to individuals of different ontogenetic stages showing conservative morphology from hatching to adults. A revised diagnosis for the species is presented here, together with its inclusion in a global phylogenetic analysis of Pelomedusoides that shows that this species and the whole *Neochelys* spp. is sister to the *Erymnochelys madagascariensis*-*Peltocephalus dumerilianus* clade within Podocnemididae.

## Introduction

Pleurodires or side-necked turtles are restricted today to South America, Africa, Madagascar, and Australia (Rhodin et al., 2010). However, their fossil record in particular that of the clades Podocnemididae and Bothremydidae shows a wider global distribution during the Late Cretaceous and Cenozoic, with abundant occurrences in Europe (Gaffney, Tong & Meylan, 2006; Gaffney et al., 2011; Georgalis et al., 2013; Pérez-García & Lapparent de Broin, 2013). European podocnemidids are included in a single genus (*Neochelys*) with at least eight clearly defined species (*N. arenarum*, *N. capellinii, N. eocaenica, N. franzeni, N. laurenti, N. liriae, N. salmanticensis*, and *N. zamorensis*) restricted to the Eocene (Early Ypresian to Bartonian, ∼56–38 Ma) (see Pérez-García & Lapparent de Broin, 2013) for details about species authorities, localities, morphological comparison of species, and issues relating to fragmentary material.

Despite of the large number of named species of *Neochelys*, only three of them (*N. arenarum*, *N. laurenti*, and *N. franzeni*) are known from both skull and shell specimens, and of these three, *N. franzeni* Schleich, 1993 is the only one with complete articulated skeletons including skull, shell, neck, and other postcranial elements. Thus, *N. franzeni* is a key taxon for understanding the major differences in morphology between *Neochelys* and the other podocnemidids, as well as for clarifying its phylogenetic position within Podocnemididae, which has been inferred in previous studies to be nested within the clade Erymnochelyinae (Lapparent de Broin, 2000; Meylan, Gaffney & Campos, 2009; Gaffney et al., 2011; Cadena, Bloch & Jaramillo, 2012; Cadena et al., 2012).

*Neochelys franzeni* was originally described based on two specimens, SMF ME 1091 (holotype) and SMF ME 715 (paratype), as the first pleurodire turtle from Messel Pit (Schleich, 1993) (Supplemental Information 1, Fig. 1). Messel Pit was the first UNESCO, World Natural Heritage Site in Germany, since 1995, with highly diverse and exceptionally preserved fossils of vertebrates, invertebrates, and plants from the middle Eocene (Early Lutetian, MP11, ∼47 Ma) (Lehmann & Schaal, 2012, and references therein). Besides the pleurodire *N. franzeni*, cryptodires or hidden-necked turtles also occur at Messel Pit including a geoemydid “*Ocadia*” sp., a trionychid (soft shell turtle) “*Trionyx*” sp., and the world best skeletons of carettochelyid turtle species *Allaeochelys crassesculpta* including specimens that died while they were copulating (Joyce et al., 2012; Schleich, 1993). For the last decades, continuous seasonal digging at Messel pit by Hessisches Landesmuseum Darmstadt (HLMD) and Senckenberg Forschungsinstitut und Naturmuseum (SMF) resulted in the discovery of five new specimens of *N. franzeni*, two of them constituting the largest and the smallest individuals for the species so far collected. Here I described those five new specimens of *N. franzeni*, and redescribe the holotype and paratype previously reported by Schleich (1993). In order to resolve the phylogenetic position of *N. franzeni* among other podocnemidids, I include this species in a phylogenetic analysis, refining also the morphological character list for Pelomedusoides (particularly podocnemidids and bothremydids). Although specimens show some degree of variability, I decided to define all specimens as belonging to one species as I will explain in detail in the discussion.

**Figure 1:**
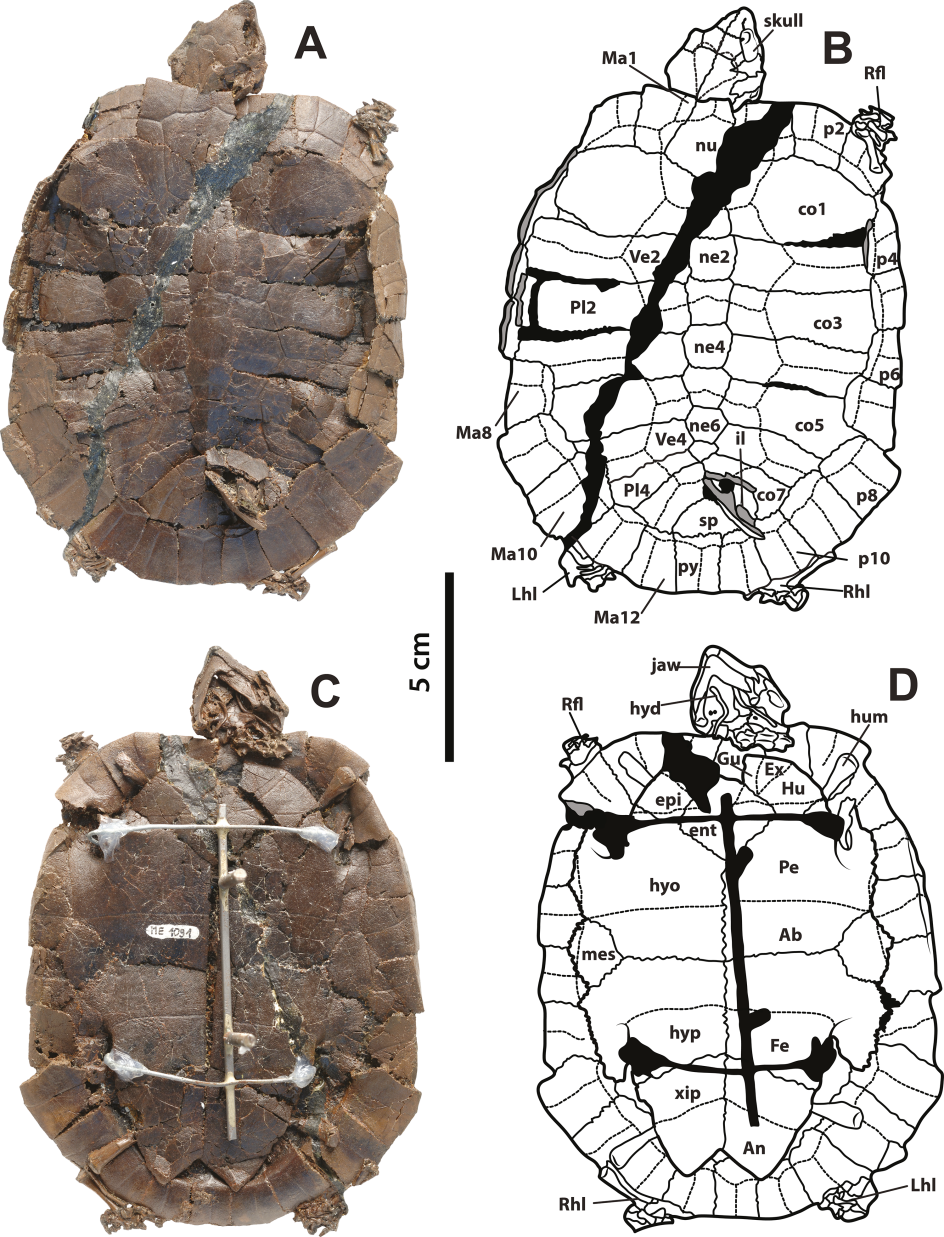
*Neochelys franzeni* SMF ME 1091 holotype. (A)–(B) Articulated skeleton in dorsal view. (C)–(D) Articulated skeleton in ventral view. Abbreviations: Ab, abdominal scute; An, anal scute; co, costal bone; ent, entoplastron; epi, epiplastron; Ex, extragular; Fe, femoral scute; fem, femur; Gu, gular; Hu, humeral scute; hum, humerus; hyd, hyoid apparatus, ceratobrachial 2; hyo, hyoplastron; hyp, hypoplastron; il, illium; LhL, left hindlimb elements, Ma, marginal scute; mes, mesoplastron; ne, neural bone; nu, nuchal; p, peripheral; Pl, pleural scute; py, pygal; Rfl, right forelimb elements; Rhl, right hindlimb elements; sp, suprapygal; Ve, vertebral scute; xip, xiphiplastron. Free spaces between bones or resins used during curation of fossils are shadowed in black. Broken areas of bone facing vertical or transversally are shadowed in grey.

## Methods

### Fossil specimens

The seven specimens of *Neochelys franzeni* described here are housed in three different institutions: three at the Senckenberg Research Institute and Natural History Museum, Frankfurt am Main, Germany (SMF ME 1091, 715, and 1267), three at the Hessisches Landesmuseum Darmstadt, Germany (HLMD-Me 15576, 14981, and 15375), and one at the Institut royal des Sciences naturelles de Belgique, Brussels, Belgium (NR 202/617). Measurements of the specimens as preserved were taken with a precision caliper (Table 1). Specimens were photographed with a professional Nikon camera and Leica binocular microscope, and some of them were coated in ammonium chloride for better visualization of sutures, sulci, and general morphology (Supplemental Information 1, plates 1–7). Fossil specimens of *N. arenarum*, *N. laurenti*, and *N. liriae* described by Broin (1977), Pérez-García & Lapparent de Broin (2013) and Tong (1998) and housed at the Muséum National d’Histoire Naturelle de Paris, France, were also directly examined for comparisons.

**Table 1:** Measurements *Neochelys franzeni* specimens in centimeters as preserved.

Specimen	Total length carapace	Total width carapace	Total length skull	Total width skull
SMF ME 1267	25.3	22.2	4.9	4.2
SMF ME 715	13.5	11.2	3.5	2.7
SMF ME 1091	15.3	12.3	3.9	2.8
HLMD ME 15576	11.1	8.2	2.2	1.7
HLMD ME 14981	6.6	5.3	1.6	1.8
HLMD ME 15375	6.3	5.6	1.6	1.4
NR 202/617	6.5	5.5	1.7	1.9

### Phylogenetic analysis

A character-taxon matrix was built comprising 187 morphological characters and 101 taxa. The complete list of characters, changes to scoring to matrices from previous matrices, and composite plates with the figures of all characters are found in Supplemental Information 2. Additionally, modifiable .eps files for individual characters can be requested by emailing the author. Most of the characters were taken from Gaffney, Tong & Meylan (2006); Cadena et al. (2012) and Romano et al. (2014). Molecular sequence data from GenBank (characters 188–5,518) for extant species was taken from Cadena et al. (2012). Mesquite Version 2.75 (Maddison & Maddison, 2014) was used to built the matrix and saved as a Nexus file (Supplemental Information 3) for subsequent analysis using PAUP Version 4.0a136 (Swofford, 2002) and TNT Version 1.1 (Goloboff, Farris & Nixon, 2008). Phylogenetic analyses were run using a heuristic search (in PAUP) and New Technology search (in TNT), for both 1000 random taxon addition replicates and TBR collapsing rule were used, keeping all trees found. All morphological characters were equally weighted and unordered. Multistate taxa were treated as polymorphic. *Proganochelys quenstedti* Gaffney, 1990 and *Palaeochersis talampayensis* Rougier, de la Fuente & Arcucci, 1995 were considered as the outgroup taxa. Bootstrap values were calculated from 1,000 replicates using the same settings as the primary search, and Decay (Bremer) Indices were obtained using TreeRot v. 2 (Sorenson & Franzosa, 2007).

## Systematic Paleontology

**Table:** 

**Figure 2:**
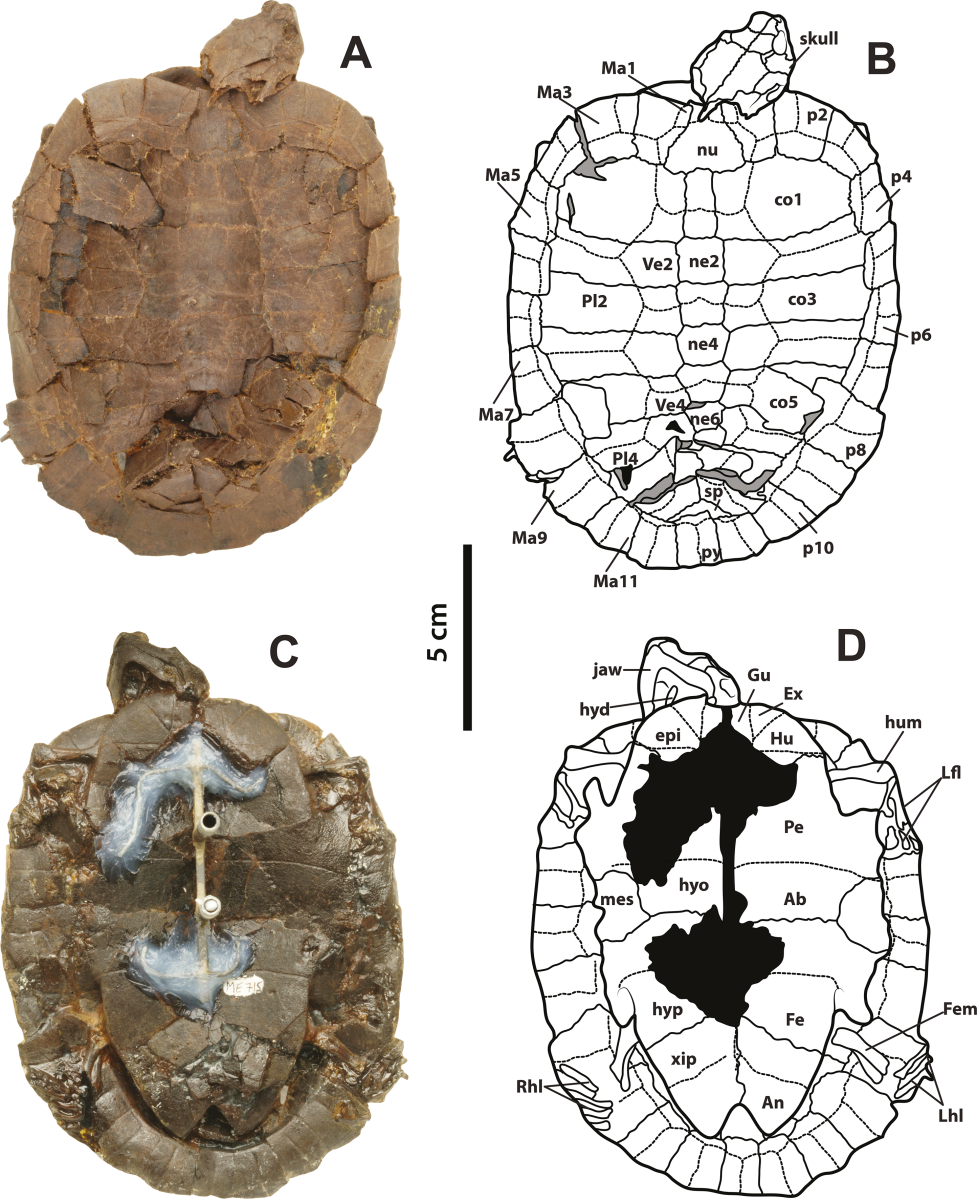
*Neochelys franzeni* SMF ME 715. (A)–(B) Articulated skeleton in dorsal view. (C)–(D) Articulated skeleton in ventral view. Abbreviations and shadowed areas legend as in Fig. 1.

**Figure 3:**
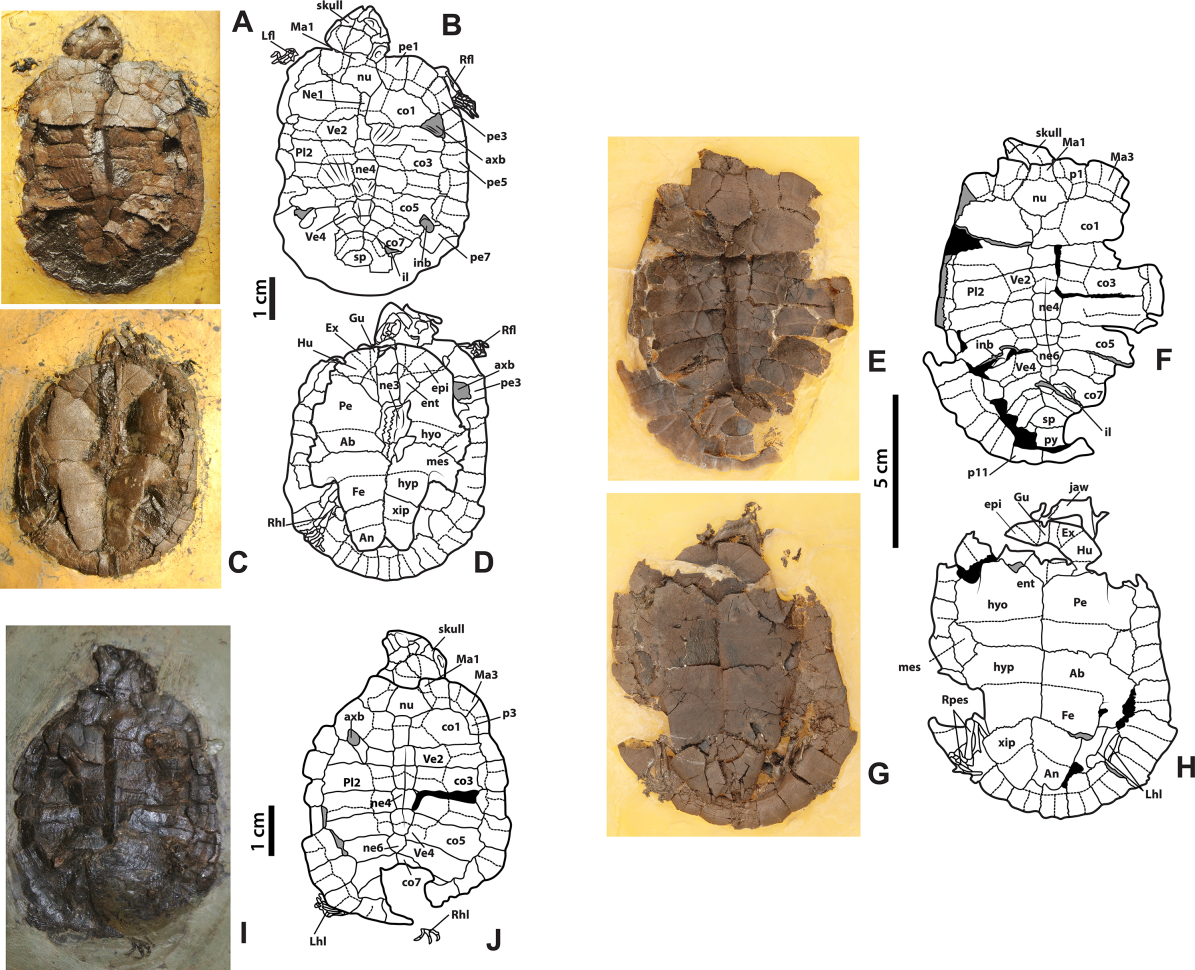
*Neochelys franzeni* HLMD-ME 14981, 15576, and 15375. (A)–(B) Articulated skeleton in dorsal view. (C)–(D) Articulated skeleton in ventral view. HLMD-ME 15576: (E)–(F) articulated skeleton in dorsal view. (G)–(H) Articulated skeleton in ventral view. NR 202/617: (I)–(J) articulated skeleton in dorsal view. Abbreviations: axb, axillary buttress; inb, inguinal buttress (see Fig. 1 for the other abbreviations and shadowed areas legend).

**Figure 4:**
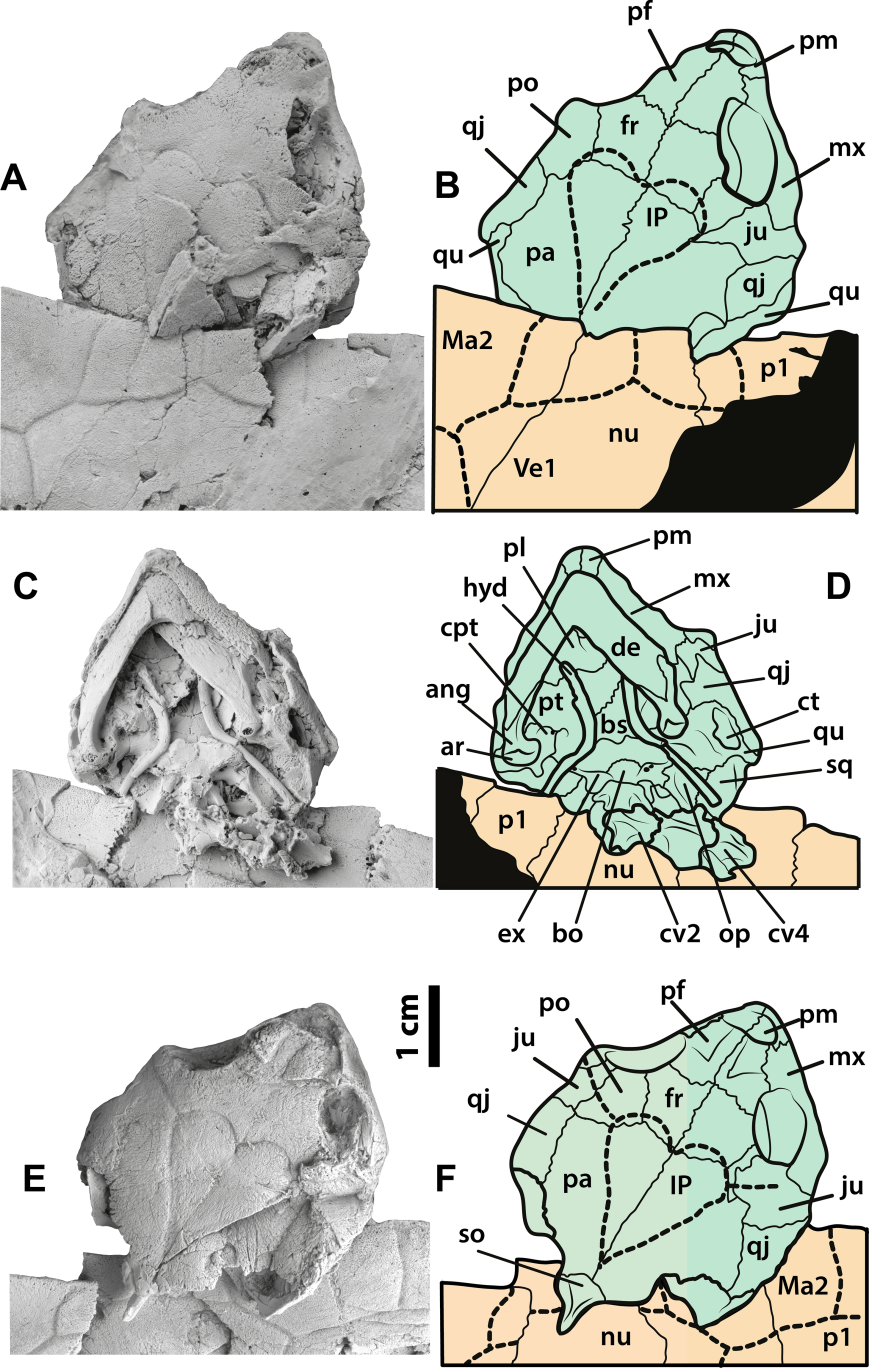
*Neochelys franzeni* SMF ME 1091 and 715 skulls. SMF ME 1091: (A)–(B) skull in dorsal view. SMF ME 715: (C)–(D) skull in dorsal view. (E)–(F) Skull in ventral view. Abbreviations: ang, angular; ar, articular; bo, basioccipital; bs, basisphenoid; cpt, cavum pterygoidei; ct, cavum tympani; cv, cervical vertebra; de, dentary; ex, exoccipital; fr, frontal; hyd, hyoid apparatus, ceratobrachial 2; IP, interparietal scute; ju, jugal; Ma, marginal scute; mx, maxilla; nu, nuchal; op, opisthotic; p, peripheral; pa, parietal; pf, prefrontal; pl, palatine; pm, premaxilla; po, postorbital; pt, pterygoid; qj, quadratojugal; qu, quadrate; sq, squamosal; so, supraoccipital; ti, tibia; Ve, vertebral scute.

**Figure 5:**
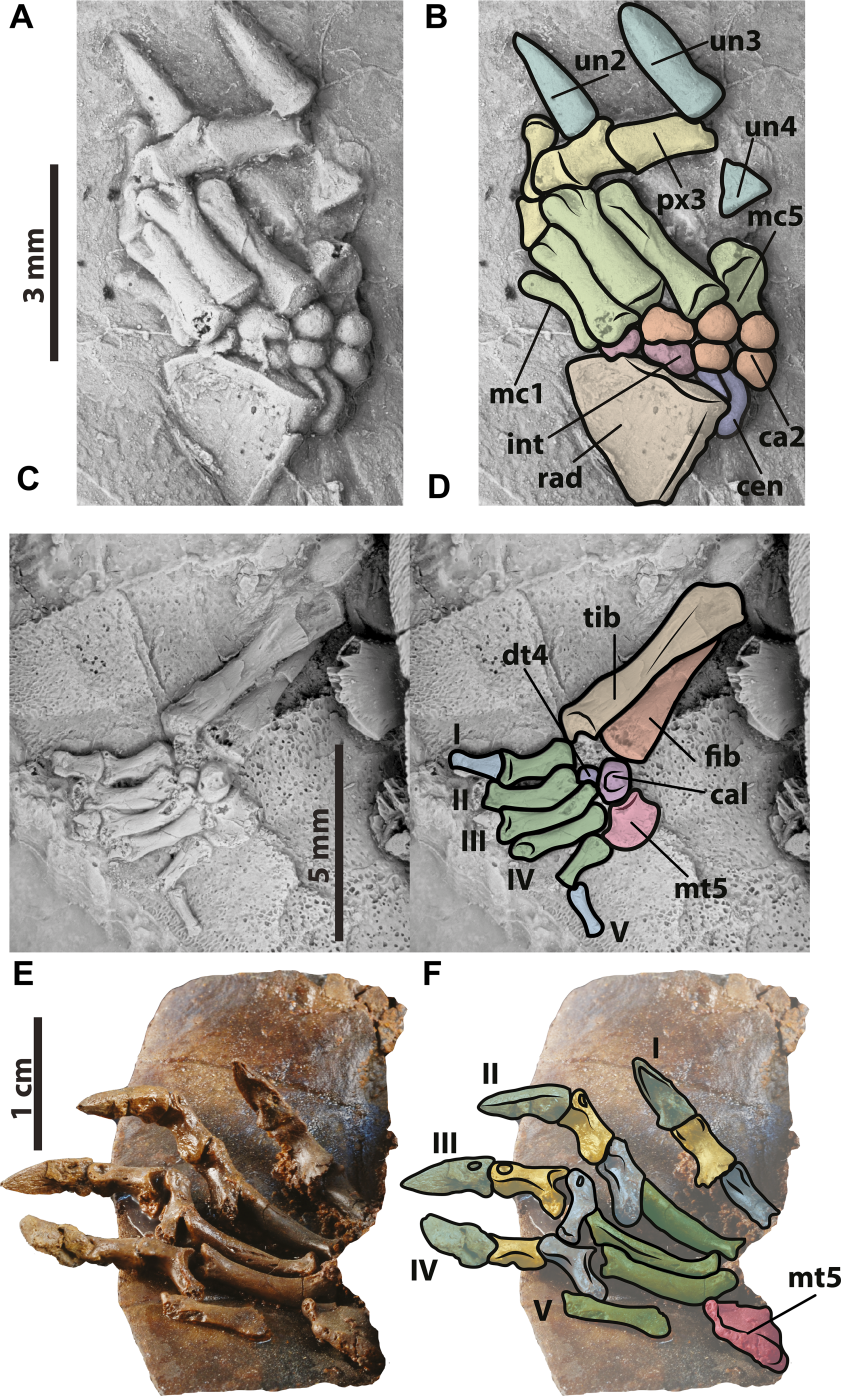
*Neochelys franzeni* HLMD-ME 15576 and 14981, and SMF ME 1267. (A)–(B) Right manus elements. HLMD-ME 14981: (E)–(F) right pes elements. SMF ME 1267: (E)–(F) right pes elements. Abbreviations: ca, carpals; cal, calcaneum; cen, central; dt, distal tarsal; fib, fibula; int, intermedium; mc, metacarpal; mt, metatarsal; px, phalanx; rad, radiale; tib, tibia; un, ungual.

TESTUDINES Batsch, 1788
PANPLEURODIRA *sensu* Cadena & Joyce, 2015
PELOMEDUSOIDES Cope, 1868
PODOCNEMIDIDAE Cope, 1868
ERYMNOCHELYINAE Broin, 1988
*Neochelys* Bergounioux, 1954
*Neochelys franzeni* Schleich, 1993
(Figs. 1–5)

### Holotype

SMF ME 1091, almost complete articulated skeleton (Figs. 1A–1D and 4A–4B, and Supplemental Information 1, plate 1), including carapace, plastron, skull and mandible, both ceratobrachial 2 bones, three cervical vertebrae, both femora, both humeri, and most of the pes and manus bones.

### Referred specimens

**Figure 6:**
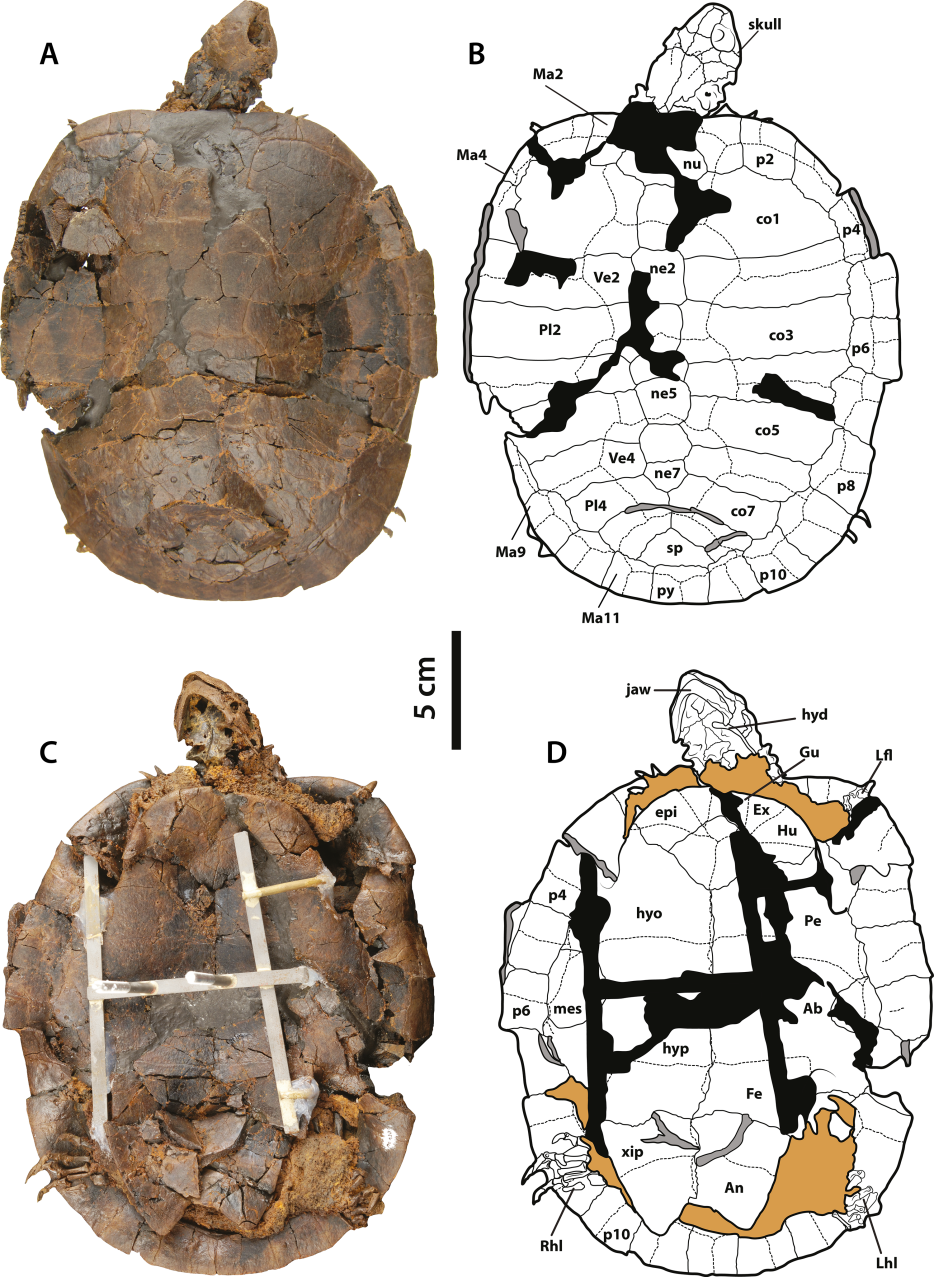
*Neochelys franzeni* SMF ME 1267. (A)–(B) Articulated skeleton in dorsal view. (C)–(D) Articulated skeleton in ventral view. Abbreviations and shadowed areas legend as in Fig. 1, plus rock matrix shadowed in orange.

**Figure 7:**
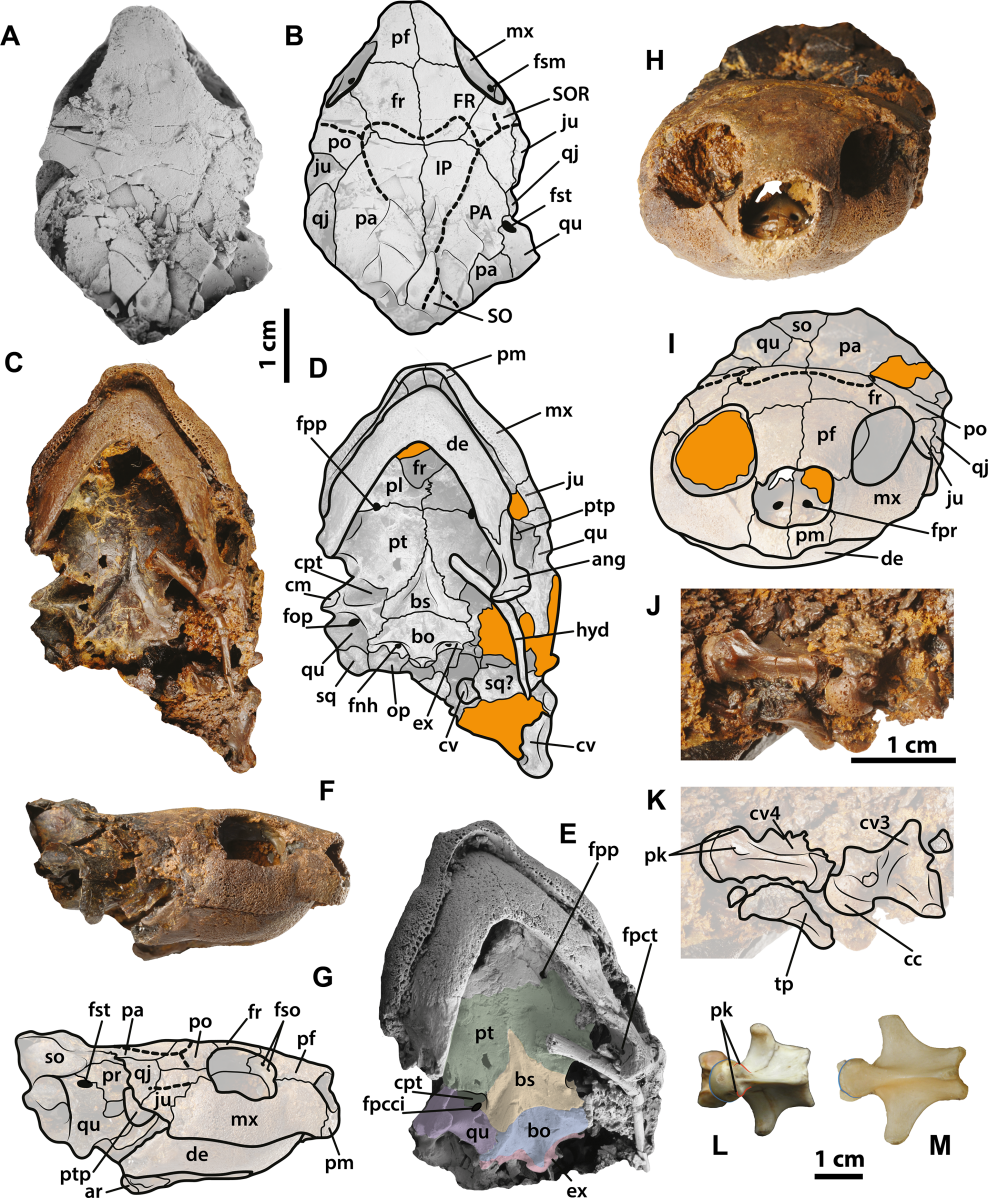
*Neochelys franzeni* SMF ME 1267 skull and extant podocnemidids. (A)–(B) Skull in dorsal view. (C)–(D) Skull in ventral view. (F)–(G) Skull in left lateral view. (H)–(I) Skull in anterior view. (J)–(K) Cervical vertebrae 3 and 4 in ventrolateral view. *Erymnochelys madagascariensis* NMW 422: L, cervical 4 in ventral view. *Podocnemis expansa* AMNH 62947: M, cervical 4 in ventral view. Abbreviations: fpct, foramen posterius chorda tympani; cc, cervical centrum; fnh, foramen nervi hypoglossi; fpcci; foramen posterior canalis caroticum interni; fpp, foramen platinum posterius; fpr, foramen praepalatinum; fso, foramen supraorbitale; fst, foramen stapedio-temporale; pk, posterolateral keel, ptp, processus trochlearis pterygoidei; SO, supraoccipital scute; tp, transversal process (see Fig. 4 for the other abbreviations). Rock matrix shadowed in orange.

SMF ME 715, almost complete articulated skeleton (Figs. 2A–2D and 4C–4F Supplemental Information 1, plate 2), including carapace, plastron, skull and mandible, right ceratobrachial 2 bone, left femur, both humeri, and some elements of left manus and both pes; HLMD-Me 14981, almost complete articulated skeleton (Figs. 3A–3D and 5C–5D, Supplemental Information 1, plates 3 and 4), including carapace, plastron, skull and mandible, right forelimb articulated, some of the right manus bones, and the right hindlimb articulated; HLMD-Me 15576, almost complete articulated skeleton (Figs. 3E–3H and 5A–5B, Supplemental Information 1, plate 5), including carapace (missing most of right posterior and middle peripherals), plastron, skull and mandible, most of right hindlimb and left femur; HLMD-Me 15375, nearly complete articulated skeleton, extremely crushed and poorly preserved, including carapace, plastron, skull and mandible, and some elements of the left hindlimb) (Supplemental Information 1, plate 7); NR 202 / 617, almost complete articulated carapace and skull, with most of the both pes bones (Figs. 3I–3J, Supplemental Information 1, plate 8); SMF ME 1267, almost complete articulated skeleton (Figs. 5E–5F, 6 and 7A–7K), including carapace, plastron, skull and mandible, three cervical vertebrae, some left and right phalanges, some left pes bones, and the almost complete articulated right pes.

### Locality and horizons

All specimens were collected in the Messel Pit, near Darmstadt, Germany. SMF ME 1091, 715, and 1267 were collected from excavation pit H7; HLMD-Me 14981 and 15576 from “Turtle Hill” (PQ HI 7), at 90 and 52 cm, respectively, below marker bed Gamma. HLMD-Me 15375 from excavation pit PQ E 10, close to marker bed Alpha (see Supplemental Information 1, Fig. 1, for figure of the Messel Pit stratigraphy and marker beds).

### Revised diagnosis

*Neochelys franzeni* differs from all other species of *Neochelys* by the presence of the following autapomorphies: a very acute anterior tip of the basisphenoid; a heart-shaped interparietal scute with a deeply incised anteromedian margin; and a ridge running along the neural series of the carapace. It shares with *N. capellinii* MCSNV 2353 a vertebral scute 1 distinctly narrower than vertebrals 2–4, and vertebral scutes 2 and 3 being hexagonal, wider than long, with tapering lateral margins.

## General Description

The following morphological description applies to all seven specimens of *Neochelys franzeni*, except as noted specifically with the respective specimen number. I follow the anatomical proposal of Gaffney (1972) and Hutchison & Bramble (1981) used in recent studies (Cadena & Joyce, 2015; Cadena, Bloch & Jaramillo, 2012; Cadena et al., 2012; Joyce, 2007; Pérez-García & Lapparent de Broin, 2013). Instead of “scale(s)”, the term “scute(s)” is used here for skull and shell. Series of bones or scutes are referred using ordinal numbers. Morphological features that correspond to characters used in the phylogenetic analysis are indicated in parenthesis. It was not considered necessary to remove the metal support originally attached to the specimens for their exhibition or the resins used to stabilize the fossils, because these do not obscure key morphological features like sutures or sulci.

### Skull, roofing elements

The skull of *Neochelys franzeni* is slightly longer than wide (see Table 1, for measurements). All the skulls are crushed, except SMF ME 1267, which preserves most of its original three-dimensional volume and shape, only slightly deformed and fractured posteriorly (Fig. 7). The prefrontals nearly reach the anteroposterior midpoint of the orbits. Anteriorly they contact one another along the midline (Character 3), but posteriorly they are separated by the frontals. Anterolaterally they contact the maxillae, and anteriorly completely cover the aperture narium externa (Character 5) (Figs. 7A–7B). The prefrontal-frontal anterodorsal shape (Character 10) seen in lateral view of SMF ME 1267 (Figs. 7F–7G) is slightly convex. The frontals are wider than long, laterally reaching the orbital margin, posterolaterally they contact the postorbitals and posteriorly the parietals. The direction of the orbits (Character 11), best preserved in SMF ME 1267 (Figs. 7A–7B), is laterally facing without dorsal exposure of the maxilla-jugal contact. The parietals contact the postorbitals anterolaterally, the quadratojugals laterally, and the supraoccipital posteriorly, as seen in SMF ME 715 (Figs. 4E–4F). *Neochelys franzeni* has postorbitals that reach the posterior orbital margin, and posteriorly contact the quadratojugals. The jugals also contribute to the orbital margin, having a medial contact with parietals, and an apparent very shallow cheek emargination (Character 25) (Figs. 7F–7G). The quadratojugals contact the parietals medially and are posteriorly extensive, forming together with the parietals a partial roof over the fossa temporalis, with a straight to slightly convex margin (Character 14) (Figs. 4A–4B and 4E–4F). Sulci of the skull scutes are well preserved in all specimens, particularly the interparietal scute, which has a heart-like shape (Character 19) (Figs. 4E–4F, Supplemental Information 1, plate 1). There is also evidence of a posterior supraoccipital scute in SMF ME 1267 (Figs. 7A–7B). On the ventral surface of the orbital ring, the foramen supramaxillare is visible in NR 202/617 (Supplemental Information 1, plate 8) and SMF ME 1267 (Figs. 7A–7B).

### Skull, palatal-braincase elements

Only SMF ME 1267 (Figs. 7C–7E) and SMF ME 1091 (Figs. 4C–4D) provide good views of the palatal and braincase elements. The premaxillae meet medially and define the lateral and ventral margins of the aperture narium externa, which lack a ventral peak, and possess a large pair of foramina praepalatinum (Figs. 7H–7I). The maxillae are robust with a highly pitted bone surface (where the ramphoteca was attached), contacting only the jugals posteriorly (Figs. 7F–7G). Because all the skulls have articulated jaws, the presence or absent of the vomer is unknown (Character 46). The palatines meet medially, and the foramen palatinum posterius lies at the sutural contact with pterygoids (Character 48) (Figs. 7C–7D). The pterygoids are relatively flat, laterally exhibiting the processus trochlearis pterygoidei (Character 68), which is mostly hidden by the jaw (Figs. 7C–7D). A cavum pterygoidei is present and deep (Character 65), and partially covered by the pterygoid flange (Character 67), but whether the flange projected ventrally is unknown, because its margin is broken or hidden by the ceratobrachial 2 of the hyoid apparatus (Figs. 4C–4D and 7C–7E). The basisphenoid has a pentagonal, anteriorly elongated shape (Character 103) (Figs. 7C–7D) with a shallow ventral concavity; laterally it contacts the quadrates (Character 102) and posteriorly the basioccipital. The basioccipital is much longer than wide (Character 88), lacking of horizontal occipital shelf (Character 91). Together with the exoccipitals, the basioccipital participates in the formation of the condylus occipitalis (Character 84). The exoccipitals exhibit the two foramina nervi hypoglossi separated on occipital surface (Character 87) (Figs. 4C–4D), and the foramen jugulare posterius is completely closed by bone (Character 83). The quadrates of *N. franzeni* contact the basioccipital (Character 61) and basisphenoid medially through the medial process. Due to crushing it is not clear if the quadrates contact the exoccipitals. The cavum tympani is completely closed (Character 56) (Figs. 7F–7G), without evidence of a fossa precolumellaris (Character 59). SMF ME 715 preserves in good condition the posterolateral region of left quadrate (Figs. 4E–4F) showing a highly to almost completely reduced antrum postoticum (Character 55) and a very large and anterodorsally oriented foramen stapedio-temporale (Character 93) (Figs. 7A–7B and 7F–7G). The most medial portion of the condylus mandibularis is preserved on the right quadrate of SMF ME 1267, however it is difficult to establish its original outline (Character 63). The opisthotic and squamosal bones are preserved on the right side of SMF ME 1091, the first one exhibiting a very small processus paroccipitalis (Character 100) restricted anteriorly before the posterior end of the squamosal. Ceratobrachials 2 are preserved in SMF ME 1091, 1267 and 715 (Figs. 2C–2D, 4C–4D and 7C–7D); they are very low and thin elements, preserved almost in the original position.

### Lower jaw

All specimens except NR 202/617 preserve the lower jaw articulated with the skull. The dentaries have a V-shaped junction, which subtends an angle of less than 90° at the symphysis (Character 114). The medial and lateral margins of both dentaries are parallel, showing a width that is almost equal to the length at the symphysis. The processus retroarticularis of the articulars is very short and projects slightly posterolaterally (Character 118) (Figs. 4C–4D and 7C–7D). On the left ramus of SMF ME 1267, the foramen posterius chorda tympani is visible and enclosed by the angular (Fig. 7E).

### Cervical vertebrae

Three cervical vertebrae of *Neochelys franzeni* are visible in SMF ME 1267 and two in 1091 (Figs. 4C–4D and 7J–7K), but most of these have the transverse and dorsal elements broken or hidden by the rock matrix. Additional mechanical preparation could jeapardize the stability of the skull, so it was forgone. Cervicals 2 to 4 are preserved in SMF ME 1091 and 3 to 4 in ME 1267; the last two show a nearly circular central shape (Character 124), and each bears a pair of short ventrolateral keels.

### Limbs

*Neochelys franzeni* specimens from Messel Pit preserve most of their fore- and hindlimb elements, although generally disarticulated or slightly displaced between them. Manual elements comprise the long and narrow unguals, phalanges that are shorter than the five metacarpals, and distal carpals that are circular to slightly oval in shape (Figs. 5A–5D). The left humerus is well preserved in SMF ME 1091 (Supplemental Information 1, plate 1) exhibiting the laterally open ectepicondylar foramen. As in the manus, the pes of *N. franzeni* has unguals long and narrow, with two non-ungual phalanges per digit that are shorter than metacarpals and are particularly well preserved and articulated in SMF ME 1267 (Figs. 5E–5F. Supplemental Information 1, plate 6). The right tibia and fibula are well preserved in HLMD-Me 14981 and 15576 (Figs. 5C–5D, Supplemental Information 1, plate 6), as is the left femur in HLMD-Me 15576 (Supplemental Information 1, plate 6).

### Carapace

The carapace of *Neochelys franzeni* is oval in shape, slightly longer than wide, with rounded anterior and posterior lateral margins. The bone surface of the carapace is finely micropitted, with dichotomous sulci (Supplemental Information 1, plate 3) and occasional radial striations over the vertebral scutes regions, particularly marked in HLMD-Me 14981 (Supplemental Information 1, plate 3). A mid-sagittal carapacial ridge is present on the neural series (Character 141), strongly marked in HLMD-Me 15576 (Supplemental Information 1, plate 5).

*Neochelys franzeni* has a nuchal bone slightly wider than long (Character 135) (Supplemental Information 1, plate 6). The neural series is composed of seven bones (Character 139), except for NR 202/617, which has six, but in all specimens the neural series reaches costals 7 (Character 137). Neural 1 is rectangular in shape and only contacts costals 1 laterally, while neural 3 contacts costals 2 (Character 140), except for SMF ME 1267, where neural 3 only contacts costals 3. There are eight pairs of costal bones, costals 7 and 8 meeting medially. Costal 1 is almost twice as long of costal 2 (Character 143). The inguinal buttress is restricted to costal 5 (Character 145) (Figs. 3A–3B). *Neochelys franzeni* has eleven pairs of peripheral bones. The axillary buttress reaches the posteromedial margin of peripheral 3 (Character 146) (Fig. 6C–6D). The width of the anterior margin of peripheral 1 is almost equal to its lateral margin length (Character 148). The posterior series of peripherals (7 to 11) are all rectangular in shape, longer than wide.

A cervical scute is absent in *Neochelys franzeni* (Character 150) (Figs. 3E–3F. Supplemental Information 1, plate 6). Marginals 1 have a distinctive anterolateral notch (Supplemental Information 1, plate 6); their anterior margin is longer than the lateral one (Character 150), and overlaps less than 30% of peripheral 1 along its anterior margin (Character 151). All twelve pairs of marginal scutes are restricted to the peripherals. There are five vertebral scutes, vertebral 2 to 4 being hexagonal, 2 and 3 being wider than the other three. Vertebral 1 is slightly wider than long, with the lateral margins slightly divergent anterolaterally, and the anterior margin slightly convex (Character 156) (Supplemental Information 1, plate 2), overlapping most of the nuchal and the most medial portions of peripherals 1 (Character 157). The lateral position of the sulcus between vertebral 3 and 4 is positioned on costal 5 (Character 158). Vertebral 5 is slightly wider than long, and posteriorly overlaps pygal and peripherals 10 (Character 159) (Figs. 6A–6B). *N. franzeni* has four pairs of pleural scutes, nearly rectangular in shape, pleural 1 being the largest.

### Plastron

The plastron of *Neochelys franzeni* is smaller than its carapace, having a pair of mesoplastra roughly equidimensional and restricted to its lateral portions (Character 164) (Figs. 1C–1D, Supplemental Information 1, plate 5). The anterior plastral lobe almost reaches the anterior margin of the carapace (Character 170), is slightly convex with almost straight medial edge (Character 168), and twice as wide as long (Character 169). The entoplastron is rhomboidal (Character 165), with slightly convex posterior margin. The hyoplastra are slightly longer than the hypoplastra at the midline. The right hyoplastron of SMF ME 1267 exhibits a single axillary musk duck located close to the sutural contact with peripherals (Character 173). The xiphiplastra have very acute posterior tips and form a V-shaped anal notch.

*Neochelys franzeni* has a gular scute overlapping most of the anterior portion of entoplastron, generally excluding extragulars and humerals, with extragulars reaching in some cases the anterolateral portion of entoplastron (Character 181) (Figs. 6C–6D, Supplemental Information 1, plate 3). The pectoral scutes cover most of the entoplastron (Character 183) and the posterior portions of epiplastra (Character 184). The pectoral-abdominal sulcus crosses the most anterior corner of the mesoplastron (Character 185) (Figs. 6C–6D). The abdominal scutes have almost the same length as the femorals at the midline (Character 187), and the anal scutes cover almost half of the xiphiplastra.

## Comparisons

Recently Pérez-García & Lapparent de Broin (2015) redescribed and redefined the systematic paleontology of “*Papoulemys*” *laurenti* Tong, 1998, now *Neochelys laurenti,* discussing and comparing not only *N. laurenti* but also all other species of *Neochelys* with extant and fossil podocnemidid turtles. However, only the *N. franzeni* material described by Schleich (1993) was considered in their discussion. Here I add all known specimens of *N. franzeni*, as described in this study, to the general discussion of *Neochelys* and its morphological comparisons with other podocnemidids. See Supplemental Information 4 for figures of dorsal, ventral, and lateral views of some podocnemidids and *Neochelys* spp. skulls.

### Skull and lower jaw

The snout of *Neochelys franzeni* is very similar in shape and proportions of the prefrontal and frontal bones to the snout of *N. arenarum* MNHN RI7 (Supplemental Information 4, plate 1), with edges slightly convergent. A narrow snout with parallel edges is considered by Pérez-García & Lapparent de Broin (2015) to be exlusively present in *Neochelys* clade, but it is also observed in some specimens of *Erymnochelys madagascariensis* Grandidie, 1867 and *Peltocephalus dumerilianus* Schweigger, 1812, as I conclude from direct examination of extant species specimens (Supplemental Information 4, list 1). As in almost all members of the clade that includes *E. madagascariensis* (*sensu* Gaffney et al., 2011), excluding *Bardemys* spp., *Schweboemys pilgrimi* Swinton, 1939, *Lemurchelys diasphax* Gaffney et al., 2011 and *Cordichelys antiqua* Andrews, 1903, the anterior margin of prefrontals of *N. franzeni* is convex and completely covers the aperture narium externa (Character 5, Supplemental Information 2). The orbits of *N. franzeni* are laterally oriented (Character 11), as in all other species of *Neochelys* and *E. madagascariensis*. In *P. dumerilianus*, the orbits are even more vertically oriented, almost not visible in dorsal view, this is also the condition of *N. arenarum* MNHN RI7 and *Dacquemis paleomorpha* Williams, 1954. The size (height and length) of the orbits in *N. franzeni*, as in all other species of *Neochelys*, is relatively larger than in some other members of *E. madagascariensis* clade (see Pérez-García & Lapparent de Broin, 2013, for list of species forming this clade) (Supplemental Information 4, plate 3). The interorbital space of *N. franzeni*, considered at the mid-orbit is similar to that in *N. arenarum* MNHN RI6, *N. laurenti* and *E. madagascariensis*, but slightly wider than in *N. arenarum* MNHN RI7, showing that there is intraspecific variation in this feature. As in all other podocnemidids (except for *Podocnemis* spp. and *Cerrejonemys wayuunaiki* Cadena, Bloch & Jaramillo, 2010), the jugals of *N. franzeni* do not contact the parietals (Character 26). The temporal emargination (Character 14) is very reduced in *N. franzeni*, with the posterior margins of parietals convex to slightly straight, as in other species of *Neochelys*, *E. madagascariensis*, and *P. dumerilianus*, but less extreme than in *D. paleomorpha*. As in all other species of *Neochelys*, the interparietal scute (Character 19) of *N. franzeni* has a slightly elongated heart-like shape, with anterior margin located anterior to the frontoparietal suture; this is an autapomorphy of the genus within *E. madagascariensis* clade. Outside *E. madagascariensis* clade, a heart-shaped interparietal scute is only present in *Bauremys elegans* Suárez, 1969. Pérez-García & Lapparent de Broin (2013) considered that *D. paleomorpha* shares the same interparietal scute shape with *Neochelys* spp. and *B. elegans*, but in *D. paleomorpha* the median notch at the anterior margin of the scute is very shallow—nearly absent—and located posterior to the frontoparietal suture. *N. franzeni* shares with all other species of *Neochelys* and other podocnemidids (except *P. dumerilianus* and *Bairdemys* spp.) a medial contact between parietal scutes (Character 20).

In the lateral view of the skull of *N. franzeni* SMF ME 1267 (Supplemental Information 4, plate 3), the most ventral preserved portions of right jugal and quadratojugal show that the cheek emargination (Character 25) was potentially very shallow, similar to the other *Neochelys* species, slightly less advanced than in *E. madagascariensis* and *P. dumerilianus*, but much better developed than in *Podocnemis* spp., *Bairdemys* spp., *B. elegans*, *Lapparentemys vilavilensis* Broin, 1971 (*sensu* Gaffney et al., 2011), *Peiropemys mezzalirai* Gaffney et al., 2011, *Caninemys tridentata* Meylan, Gaffney & Campos, 2009 and *D. paleomorpha*.

The ventral view of *Neochelys franzeni* skull (Supplemental Information 4, plate 2) shows the absence of a secondary palate (Character 44), as in other species of *Neochelys* and other podocnemidids, except Stereogenyina (*sensu* Gaffney et al., 2011). The cavum pterygoidei (Character 65) of *N. franzeni* is deep and partially covered by the pterygoid flange (Character 67) with a large anterior opening (Character 66), similar to other *Neochelys* species and all other podocnemidids. Pérez-García & Lapparent de Broin (2015) pointed out that the disappearance of most of the base of the prootic distinguishes *Neochelys* spp. from *P. dumerilianus*, but after the direct examination of the skulls of *N. arenarum*, *N. laurenti* and several specimens of *P. dumerilianus* (Supplemental Information 4), I conclude that this condition is highly variable in *P. dumerilianus*, and does not represent a good feature to differentiate these taxa. The orientation of the processus trochlearis pterygoidei (Character 69) in *N. franzeni*, as in other species of *Neochelys* and other podocnemidids except for *P. dumerilianus*, has a low inclination compared with the horizontal axis of the processus. Although it is not preserved in *N. franzeni*, the shape of the condylus mandibularis (Character 63) in other species of *Neochelys* and all other podocnemidids, except for *Podocnemis* spp. is much wider than long, with anterior and posterior edges straight to concave. *N. franzeni* has a shorter medial contact between pterygoids, due to the more acute, elongate anterior tip of the basisphenoid compared to all other podocnemidids.

The lower jaw of *Neochelys franzeni* is only visible on its ventral surface in all specimens, due to its articulation with the skull. The dentaries are fused at the symphysis, as in all other podocnemidids (Character 110). It also shares with all other podocnemidids except Stereogenyina (*sensu* Gaffney et al., 2011) a narrow triturating surface (Character 111) and a V-shaped and angle between rami less than 90° (Character 114). As in *E. madagascariensis*, *P. dumerilianus*, and other *Neochelys* preserving the lower jaw, the processus retroarticularis of the articular is very short and projects slightly posterolaterally (Character 118). In *N. franzeni* the foramen posterius chorda tympani is enclosed by the angular (Character 119), as in *E. madagascariensis* clade (Gaffney et al., 2011).

### Postcrania

The centrum of the cervical vertebrae of *N. franzeni* have an oval shape (Character 124), similar to that in *E. madagascariensis* clade (Fig. 7L) with well-defined posterolateral keels, but lacking the saddle-shape present in *Podocnemis* spp. and *Cerrejonemys wayuunaiki* (Cadena, Bloch & Jaramillo, 2010; Lapparent de Broin, 2000). Cervicals 3 to 5 of *Podocnemis* spp. can also exhibit oval and less saddle-shape centra, but they lack the posterolateral keels (Fig. 7M). The general morphology and relative size proportions of limb elements of *N. franzeni* are similar to those of Podocnemididae, including a humerus exhibiting a laterally open ectepicondylar foramen, as in *Podocnemis expansa* (Gaffney, 1990, Fig. 149D).

*Neochelys franzeni* shares with other species of *Neochelys* a slightly longer than wide carapace (Character 134) (see also Supplemental Information 3, plate 4). Small variations in the outline shape of the carapace occurs among specimens of *N. franzeni*,  which could reflect a combination of different ontogenetic stages, sexual dimorphism, or just deformation. Variation was also observe in extant specimens of *Erymnochelys madagascariensis* (Supplemental Information 4, list 1). In extant *Podocnemis* spp. the carapace tend to be wider posteriorly. The nuchal geometry (Character 135) of *N. franzeni* resembles other species of the genus, although slightly variations occur in *N. capellinii* and *N. zamorensis* (Pérez-García & Lapparent de Broin, 2013). All specimens of *N. franzeni* (except NR 202/617) have seven neural bones, as in all other species of *Neochelys* except for *N. arenarum*, which has six. In general, podocnemidids have seven neurals, but pathologies in terms of shape and number of neurals or other series of bones can occur, sometimes with ventral but not dorsal exposure. Most specimens of *N. franzeni* exhibit a ridge running along the neural series (Character 141), which distinguishes this species within *Neochelys*, but a ridge is found in other podocnemidids, e.g., *Podocnemis sextuberculata*. *Neochelys franzeni* also shares the following features with all other *Neochelys* species and other podocnemidids: costals 7 and 8 meet medially, neural 1 is rectangular and neural 7 pentagonal in shape, eight pairs of costals are present as well as eleven pairs of peripherals and one suprapygal, and the inguinal buttress restricted to costal 5 (Character 145). The axillary buttress reaches the posteromedial margin of peripheral 3 in *N. franzeni* (Character 146), as in all other podocnemidids, except for *Po. lewyana*, *Po. negrii*, *E. madagascariensis*, *C. wayuunaiki* (reaching the most anterior margin of peripheral 3), and *P. dumerilianus* (restricted to peripheral 4) (Cadena, Bloch & Jaramillo, 2010; Lapparent de Broin & Wermer, 1998). A cervical scute is absent in *Neochelys franzeni* (Character 150) as in all other pelomedusoids (Gaffney, Tong & Meylan, 2006). The shallow anterolateral notch of marginal 1 in *N. franzeni* is also present in *N. laurenti* as figured in Pérez-García & Lapparent de Broin (2015). Marginals 1 of *N. franzeni* have the same geometrical arrangement (Character 151 & 152) as in *N. arenarum* (see Pérez-García & Lapparent de Broin, 2013) for differences between other species of *Neochelys*). The anterior shape of vertebral 1 (Character 156) (see also Supplemental Information 4, plate 5) of *N. franzeni* ressembles *N. capellinii, N. zamorensis, N. salmanticensis* and most of other podocnemidids, *N. laurenti* and *N. liriae* have a lyre-shaped vertebral 1 (Pérez-García & Lapparent de Broin, 2013).

The morphology of bones and scutes of the anterior plastron of *Neochelys* species is highly variable, particularly in the size of the gular, extragulars, and humerals, and also because almost all specimens exhibit some degree of fracturing, crushing, or deformation (see Pérez-García & Lapparent de Broin, 2013, Table 2). *Neochelys franzeni* shares with all other species of *Neochelys* (except *N. arenarum, N. liriae*, and the holotype of *N. eocaenica*) and all other podocnemidids a subrounded and highly convex anterior plastral lobe (Character 168) (see also Supplemental Information 4, plate 6). The entoplastron of *N. franzeni* is generally similar in shape to that in all other podocnemidids, with variations in the length of the entoplastron versus its separation from the pectoro-abdominal sulcus (Pérez-García & Lapparent de Broin, 2013, character 12). Variation is also found in *N. franzeni* (cf. the holotype SMF ME 1091 and SMF ME 1267), proving that this particular character is intraspecifically variable, even considering the effect of breaking and crushing effect. In terms of the arrangement between anterior plastral scutes (gular, extragulars, humerals, and pectorals) (see Supplemental Information 4, plate 6) Pérez-García & Lapparent de Broin (2013, character 14) defined a single character state for each one of the eight species of *Neochelys*, and in the particular case of *N. franzeni* the arrangement was: “intergular (gular of this study) narrower than each gular (extragular of this study); gulars slightly overlapping the entoplastron; relatively narrow to narrow intergular-pectorals contact.” Comparing *N. franzeni* SMF ME 715 (Figs. 2C–2D), in which the extragulars do not reach the entoplastron, and SMF ME 1267 (Figs. 6C–6D), in which the extragulars advance widely onto the entoplastron, it is evident the arrangement of anterior plastral scutes shows high variability. Despite the variability in the arrangement of anterior plastral scutes, all *Neochelys* species share a humero-pectoral sulcus located well anterior of the epiplastron-hyoplastron suture (Character 184), whereas in all other podocnemidids the sulcus is located closer to the suture or crosses it laterally, and in some specimens of *Peltocephalus dumerilianus* (see Supplemental Information 4, plate 6) the sulcus is located posterior to the suture. All *Neochelys* species, like all other podocnemidids (except *Erymnochelys madagascariensis* and *Erymnochelys* sp. Righi, 2002 (undescribed specimen from the Eocene of Sardinia, Italy), lack of a long medial contact between extragulars (Character 180). The extragulars can be only narrowly separated or just touching, as in *N. laurenti* figured in Pérez-García & Lapparent de Broin (2015) and *N. eocaenica* (Broin, 1977), but the gular reaches the entoplastron in both cases. This is not the case in *E. madagascariensis* and *Erymnochelys* sp., which have an extremely reduced gular.

## Phylogenetical Results

**Figure 8:**
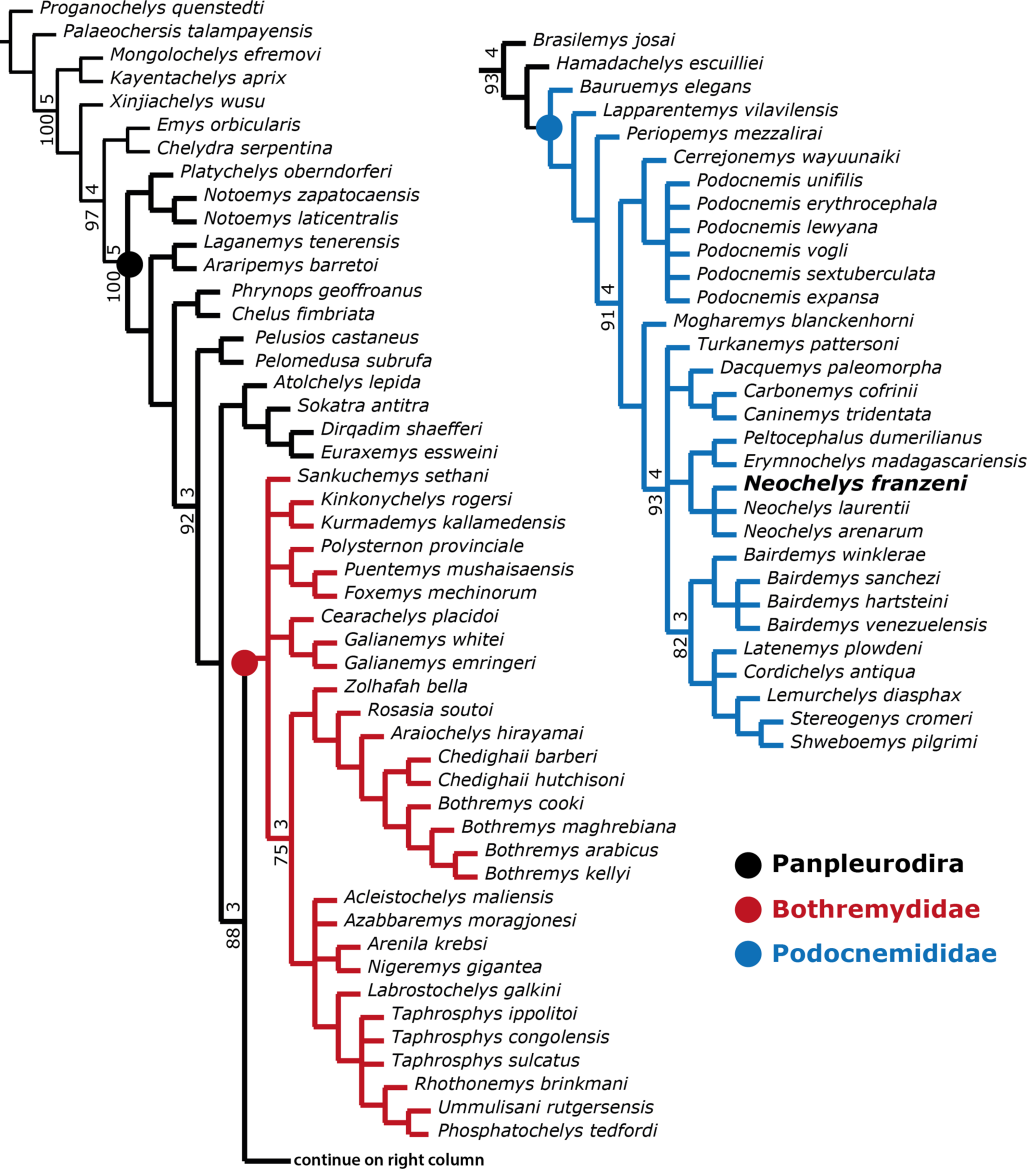
Phylogenetic trees for Pleurodira including *Neochelys franzeni*. Strict consensus of 60 retained trees in TNT, using New Technology Search, TBR, for 1,000 replicates, Tree length = 581. Bootstrap values (bottom of the branches), and Bremer support values (top of the branches).

A first parsimony run in PAUP including all taxa scored in the matrix and all morphological characters (1–187) (Supplemental Information 2) was stopped after two days of running and remaining in replicate 1, the procedure was repeated for several days more without obtain any increase in the number of replicates. TNT, which is faster than PAUP in the analysis of large volume of data (Goloboff, Farris & Nixon, 2008) was used for the initial (all taxa, all morphological characters run), obtaining a strict consensus, showing a large polytomy for all ingroup taxa (Supplemental Information 4, plate 7). A second analysis was run in TNT excluding taxa with significant amount of missing data (approx. 65%, principally shell-only taxa) which correspond with the dubious taxa mentioned by Gaffney et al. (2011), plus *Albertwoodemys testudinum* Gaffney et al., 2011, the two species of *Elochelys;* “*Neochelys*” *fajumensis* Andrews, 1903 (*sensu* Gaffney et al., 2011), *Brontochelys gaffneyi* sensu Gaffney et al., 2011, *Podocnemis bassleri* Williams, 1956 and *Dortoka vasconica* Lapparent de Broin & Murelaga, 1996; however, *Cerrejonemys wayuunaiki* was retained, considering that it has a relatively low percentage of missing data represented by skull and shell characters. A strict consensus tree of 60 best trees, 581 tree length, shows a relatively well-resolved phylogeny of Pleurodira (Fig. 8), with some polytomies within Bothremydidae and Podocnemididae. *Atolchelys lepida* Romano et al., 2014 recently described, that bothremydid is found here as closer to Euraxemydidae, but this is probably a fluke due to the relatively large amount of missing data, for when it is included in an analysis of bothremydid taxa alone the result is identical to the one obtained by Romano et al. (2014), (see discussion below).

**Figure 9:**
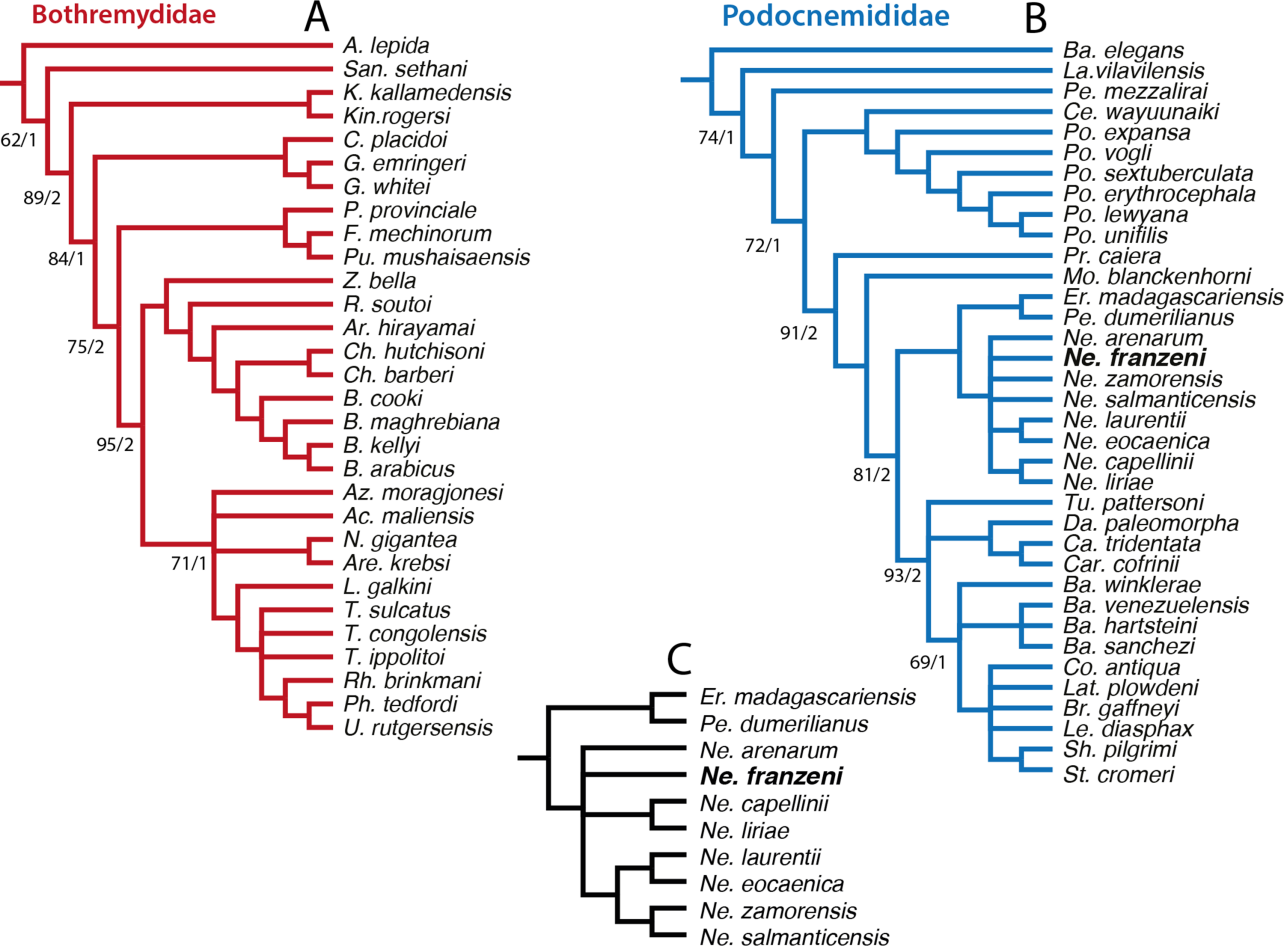
Phylogenetic trees for Bothremydidae and Podocnemididae including *Neochelys franzeni*. (A) Atrict consensus tree of 9 most parsimonious trees (TL = 454; CI = 0.551; RC = 0.448; and HI = 0.489), including only bothremydids. (B) Strict consensus tree of 17,572 most-parsimonious trees (TL = 3,176; CI = 0.706; RC = 0.439; HI = 0.304), including only podocnemidids (molecular plus morphologic data). (C) Adams consensus of the tree in (B), showing the potential relationships among Neochelys taxa. Bootstrap values (left of the slash), and Bremer support values (right of the slash).

In order to explore potentially better resolve clades, with fewer polytomies inside Bothremydidae and Podocnemididae clades, focused analyses were run in PAUP for each of these clades. The first included only Bothremydidae, and excluded all taxa basal to *Phosphatochelys tedfordi* in Fig. 8, and as for the other previous runs, molecular data was excluded. The strict consensus tree of 9 most parsimonious trees (Fig. 9A) (TL = 454; CI = 0.551; RC = 0.448; and HI = 0.489), shows agreement with trees obtained by previous studies (Gaffney, Tong & Meylan, 2006, Fig. 288; Romano et al., 2014, Fig. 2), at least for the major groups (Kurmademydini, Cearachelyini, Bothremydini, and Taphrosphynini). Compared to Gaffney, Tong & Meylan (2006 Fig. 1 and 288), Foxemydina (including *Puentemys mushaisaensis* Cadena, Bloch & Jaramillo, 2012) is found outside Bothremydodda; Romano et al. (2014) obtained a similar result, but with Foxemydina in a polytomy with Bothremydini and Taphrosphyini. *Sankuchemys sethnai* (Singh et al., 1998) as also obtained by Romano et al. (2014) is placed outside Kurmademydini. The second focused analysis—of Podocnemididae—included all taxa from Fig. 8 (except Bothremydidae). For that, molecular data were added to the parsimony analysis, in order to improve resolution between extant species. The strict consensus tree of 17,572 most-parsimonious trees (Fig. 9B) (TL = 3,176; CI = 0.706; RC = 0.439; HI = 0.304) with the present, enlarged morphological character list shows the same topology obtained in Cadena et al. (2012), Fig. 7B) for extant species of *Podocnemis* and *Cerrejonemys wayuunaiki* (Cadena, Bloch & Jaramillo, 2010). *Neochelys* (exclusive of “*N*.” *fajumensis*) is inferred to be the sister taxon of the clade *Erymnochelys madagascariensis* + *Peltocephalus dumerilianus*, which is in contrast to Gaffney et al. (2011), Fig. 98), who found it to be closely related to Stereogenyina. *Carbonemys cofrinii* Cadena et al. (2012) is found here to be the sister taxon of *Caninemys tridentata* Meylan, Gaffney & Campos (2009). Unresolved polytomies remain inside Stereogenyina, for example *Bairdemys* spp. and the clade formed by *Brontochelys gaffneyi* Gaffney et al., 2011, *Lemurchelys diasphax* Gaffney et al., 2011, *Latenemys plowdeni* Gaffney et al., 2011 and *Cordichelys antiqua* Andrews, 1903 (*sensu* Gaffney et al., 2011). Missing data (the shell of only one of the four species of *Bairdemys* spp., for instance, is known) as well as the exclusion of species specific characters in the analysis could be consider as a potential reason for these polytomies. Despite of the polytomies found inside Stereogenyina, the clade *Shweboemys pilgrimi* + *Stereogenys cromeri* was also found here, as in Gaffney et al. (2011). Adams consensus was obtained in order to explore the potential relationships among *Neochelys* taxa (Fig. 9C), showing for example that *N. laurentii, N. eocaenica, N. zamorensis*, and *N. salmanticensis* form a clade, and that *N. franzeni* and *N. arenarum* are in polytomy at the base of *Neochelys* clade.

## Discussion

### *Neochelys franzeni* ontogeny

The recovery of several specimens of *N. franzeni* of different size allowed the study of the variability or stability of morphological characters in the three different ontogenetic stages (hatchings-juveniles, juveniles, and adults), based on size and degree of ossification between sutures. The diagnostic characters of the species are constant through ontogeny, although the median ridge (keel) on the neural series is less marked in adult stages, something that is also observed in specimens of extant *Podocnemis sextuberculata*. The relative sizes of the carapacial and plastral bones and scutes show no ontogenetic trend; nevertheless, differences do occur, especially at the contact areas between scutes, e.g., in the length of extragulars in two specimens of similar ontogenetic stage (specimens HLMD-Me 14981 and 15576): in the former the extragulars touch the entoplastron and in the latter they are restricted to the epiplastron. This type of difference has been used by previous authors to distinguish between species of *Neochelys* (see Schleich, 1993, Pérez-García & Lapparent de Broin, 2013; Pérez-García & Lapparent de Broin, 2015), however I suggest that they should be only considered as strong apomorphic characters when a large sample size is available to test their invariability.

### Rediagnosis of *Neochelys franzeni*

In the original diagnosis of *N. franzeni* from Schleich (1993), eight autapomorphic features were listed, including size (around 15 cm), which is excluded from the new revised diagnosis because the size range is higher as shown here. A gular scute touching the pectorals is also excluded because it is variable among specimens, and it is also found in *N. arenarum* (see Broin, 1977, Fig. 12). The abdominofemoral sulcus straight or slightly convex at the inguinal notch level, is another highly variable morphological feature among specimens of *N. franzeni*, and was also excluded. Schleich (1993) also mentioned a rounded and flat anterior plastral lobe as apomorphic of *N. franzeni*, a condition that is also found in *N. laurenti* and *N. salmanticensis*. Other features were corroborated, retained, and more precisely given in the revised diagnosis.

### Diagnosis of *Neochelys*

Recently Pérez-García & Lapparent de Broin (2015) revised the diagnosis of *Neochelys*, giving an extensive list of skull and shell characters, however most of them are highly variable among podocnemidids, and do not follow the purpose of a diagnosis, which is to clearly show how a new or revised taxon differs from others (Cifelli & Kielan-Jaworowska, 2005). Here I focused only on the three exclusive apomorphies of *Neochelys*, including those supporting its inclusion inside the clade Podocnemididae, obtained using the “describe tree” tool of PAUP, applied to the tree shown in Fig. 9B. For similarities or differences between *Neochelys* and the other podocnemidids, see Pérez-García & Lapparent de Broin (2015). The first apomorphy of *Neochelys* is the shape of the interparietal scute (Character 19), which is heart-shaped, slightly more elongate that in *Bauremys elegans* (Suárez, 1969). A subrectangular cheek emargination (Character 25), less than half of the height of the meatus quadrati, is intermediate between the moderately advanced state in *Podocnemis* spp. and the very shallow state seen in *Erymnochelys madagascariensis* and *Peltocephalus dumerilianus* (see Supplemental Information 4, plate 3). The second and third apomorphic characters are the absence of lateral depressions before the upper beak and the narrow and prominent snout (Characters 4 & 5) (Supplemental Information 4, plates 1 & 3). The combination of these three derived features is unique to *Neochelys* among other podocnemidids, some of which also lack a lateral depressions before the upper beak but do not have a prominent snout (*Podocnemis* spp.), or have a prominent snout but also a deep lateral depression before the upper beak (*E. madagascariensis* and *P. dumerilianus*). In terms of the shell, only one character constitutes an indisputable shared and unique synapomorphy of all the eight species of *Neochelys*: the presence of a pectorohumeral sulcus located well anterior of the epiplastron-hyoplastron suture, especially at the medial margin of epiplastra (Character 184). The lack of abundant shared synapomorphies among the species of *Neochelys* could be explained by rapid evolutionary modification to shared ancestral characters, or also by a potential overestimate of past species diversity due to the necessity of authors to name new species, even when the small differences between taxa can fall inside the range of intraspecific variation, as in some of the examples given above. Besides, considering that the geographical distribution of *Neochelys* is restricted to Europe and that its geological time range is exclusively Eocene, it is also possible that a lower number of species existed (fewer than the ones described in the literature) with extensive geographical ranges, and that the small or poorly marked morphological differences between specimens from different fossil localities mentioned by previous studies could fit inside “subspecies” level, or just represent bias caused by the low sample size or the highly fragmented state of some of them.

### *Neochelys franzeni* taphonomy

Taphonomic aspects of Messel Pit turtles were recently discussed by Joyce et al. (2012) with regard to the couples of *Allaeochelys crassesculpta*. They concluded that the couples were copulating in the habitable surface waters and then sank into deeper toxic levels of the stratified volcanic maar lake. In the case of *N. franzeni*, none of the specimens so far known has been found as couples, and isolated individuals represent a wide ontogenetic range, suggesting potentially different causes of death, such as senescence, starvation, or even rapid chemical changes in the lake water. Post-mortem events in *N. franzeni* specimens include disarticulation and displacement principally of neck, tail, and limb elements; displacements are very low to absent in skull and shell elements. Crushing and slight deformation is also present, greatly affecting hatchlings and juveniles, for instance in HLMD-Me 15375, whose skeleton is almost completely crushed. Potential preservation of “blood vessels” and bone cells (principally osteocytes) is being investigated not only for *N. franzeni*, but also other turtles and fossil vertebrates from Messel Pit, considering that recent studies have shown the high occurrence of osteocytes preservation in fossil turtles (Cadena & Schweitzer, 2012; Cadena & Schweitzer, 2014).

## Supplemental Information

10.7717/peerj.1221/supp-1 Supplemental Information 1 Messel Pit location and stratigraphy, and *Neochelys franzeni* detailed and additional photographs Figure 1A, Messel Pit Fossil Site, UNESCO World Heritage. View from the visitor’s platform direction north. Fig. 1B, stratigraphic horizon of Middle Messel Formation, showing the occurrences of *N. franzeni*. Taken and modified from (Tütken, 2014) and (Hesse & Habersetzer, 1993). Plate 1, *N. franzeni* SMF ME 1091 (holotype), interparietal scute, humerus, and tibia. Plate 2, *N. franzeni* SMF ME 715, lateral and ventral views skull and vertebral scute 1. Plate 3, *N. franzeni* HLMD-Me 14981, vertebral scute 3, epiplastra, and xiphiplastra. Plate 4, *N. franzeni* HLMD-Me 14981, dorsal and ventral views skull. Plate 5, *N. franzeni* HLMD-Me 15576, neurals, left mesoplastron and epiplastra. Plate 6, *N. franzeni* HLMD-Me 15576, dorsal view skull, ventral view lower jaw, right pes, and left femur-fibula. Plate 7, *N. franzeni* HLMD-Me 15375, dorsal view skull and dorsal and ventral views complete specimen. Plate 8, *N. franzeni* SNR 202/617, dorsal view skull. Abbreviations: An, anal scute; ang, angular; ar, articular; co, costal bone; ct, cavum tympani; de, dentary; ent, entoplastron; epi, epiplastron; Ex, extragular; Fe, femoral scute; fe, femur; fi, fibula; fic; foramen intermandibularis caudalis; fpc, foramen posterius chorda tympani; fr, frontal; fsm, foramen supramaxillae; Gu, gular; Hu, humeral scute; hyd, hyoid apparatus, ceratobrachial 2; hyo, hyoplastron; hyp, hypoplastron; IP, interparietal scute; ju, jugal; Ma, marginal scute; mes, mesoplastron; mx, maxilla; ne, neural bone; nu, nuchal; p, peripheral; pa, parietal; pf, prefrontal; Pl, pleural scute; pl, palatine; pm, premaxilla; po, postorbital; pt, pterygoid; qu, quadrate; Rfib, right fibula; sq, squamosal; SO, supraoccipital scute; ti, tibia; Ve, vertebral scute; xip, xiphiplastron.

10.7717/peerj.1221/supp-2 Supplemental Information 2 Character list and characters figures List of 187 morphological characters used in the phylogenetical analysis presented in this study. Also, characters exclude and changes in scores from previous studies, as well as composite plates with figures for each of the characters. Individual .eps files for each character can be download on Dryad (LINK).

10.7717/peerj.1221/supp-3 Supplemental Information 3 NEXUS file character-taxon matrix

10.7717/peerj.1221/supp-4 Supplemental Information 4 Skulls, carapace outline, vertebral scute 1 shape, and anterior plastral composition of fossil and extant podocnemidids. List of specimens examined Photographs or figures are not to original scale. Plate 1. Podocnemididae skulls in dorsal view. Plate 2. Podocnemididae skulls in ventral view. Plate 3. Podocnemididae skulls in left lateral view. Plate 4. Carapace outline of *Neochelys* species and some extant podocnemidids. Plate 5. Vertebral scute 1 of *Neochelys* species and some extant podocnemidids. Plate 6. Anterior plastral bone and scutes for some *Neochelys* species and some extant podocnemidids. Plate 7. Strict consensus tree when all taxa and all morphological characters from character-taxon matrix are included in a new technology search, TNT. List 1. List of specimens of extant species directly examined.
