# Supplementary material for: A global phylogeny of Pelomedusoides turtles with new material of Neochelys franzeni Schleich, 1993 (Testudines, Podocnemididae) from the middle Eocene, Messel Pit, of Germany
Source: PeerJ. 2015 Aug 27;3:e1221. doi: 10.7717/peerj.1221 (PMC4556147; doi:10.7717/peerj.1221)
Supplement: Supplemental Information 1 — Figure 1A, Messel Pit Fossil Site, UNESCO World Heritage. View from the visitor’s platform direction north. Fig. 1B, stratigraphic horizon of Middle Messel Formation, showing the occurrences of N. franzeni. Taken and modified from (Tütken, 2014) and (Hesse & Habersetzer, 1993). Plate 1, N. franzeni SMF ME 1091 (holotype), interparietal scute, humerus, and tibia. Plate 2, N. franzeni SMF ME 715, lateral and ventral views skull and vertebral scute 1. Plate 3, N. franzeni HLMD-Me 14981, vertebral scute 3, epiplastra, and xiphiplastra. Plate 4, N. franzeni HLMD-Me 14981, dorsal and ventral views skull. Plate 5, N. franzeni HLMD-Me 15576, neurals, left mesoplastron and epiplastra. Plate 6, N. franzeni HLMD-Me 15576, dorsal view skull, ventral view lower jaw, right pes, and left femur-fibula. Plate 7, N. franzeni HLMD-Me 15375, dorsal view skull and dorsal and ventral views complete specimen. Plate 8, N. franzeni SNR 202/617, dorsal view skull. Abbreviations: An, anal scute; ang, angular; ar, articular; co, costal bone; ct, cavum tympani; de, dentary; ent, entoplastron; epi, epiplastron; Ex, extragular; Fe, femoral scute; fe, femur; fi, fibula; fic; foramen intermandibularis caudalis; fpc, foramen posterius chorda tympani; fr, frontal; fsm, foramen supramaxillae; Gu, gular; Hu, humeral scute; hyd, hyoid apparatus, ceratobrachial 2; hyo, hyoplastron; hyp, hypoplastron; IP, interparietal scute; ju, jugal; Ma, marginal scute; mes, mesoplastron; mx, maxilla; ne, neural bone; nu, nuchal; p, peripheral; pa, parietal; pf, prefrontal; Pl, pleural scute; pl, palatine; pm, premaxilla; po, postorbital; pt, pterygoid; qu, quadrate; Rfib, right fibula; sq, squamosal; SO, supraoccipital scute; ti, tibia; Ve, vertebral scute; xip, xiphiplastron. [file peerj-03-1221-s001.pdf]

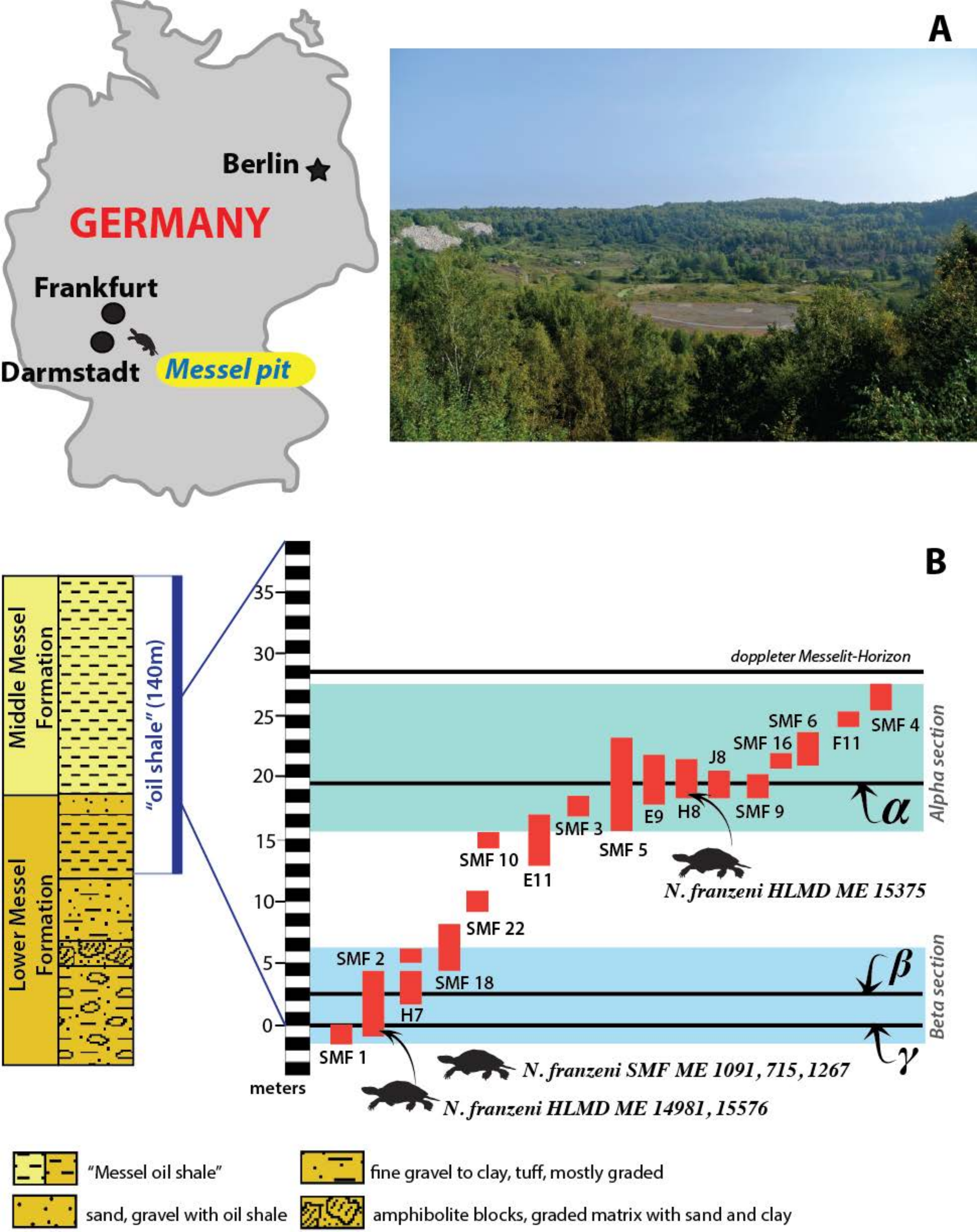
A
Berlin
GERMANY
Frankfurt
Darmstadt
Messel pit
B
Middle Messel Formation
Lower Messel Formation
"oil shale" (140m)
35
30
25
20
15
10
5
0
meters
doppleter Messelit-Horizon
Alpha section
Beta section
α
β
γ
SMF 1
SMF 2
H7
SMF 18
SMF 22
SMF 10
E11
SMF 3
SMF 5
E9
H8
J8
SMF 9
SMF 16
SMF 6
F11
SMF 4
N. franzeni HLMD ME 15375
N. franzeni SMF ME 1091, 715, 1267
N. franzeni HLMD ME 14981, 15576
"Messel oil shale"
fine gravel to clay, tuff, mostly graded
sand, gravel with oil shale
amphibolite blocks, graded matrix with sand and clay

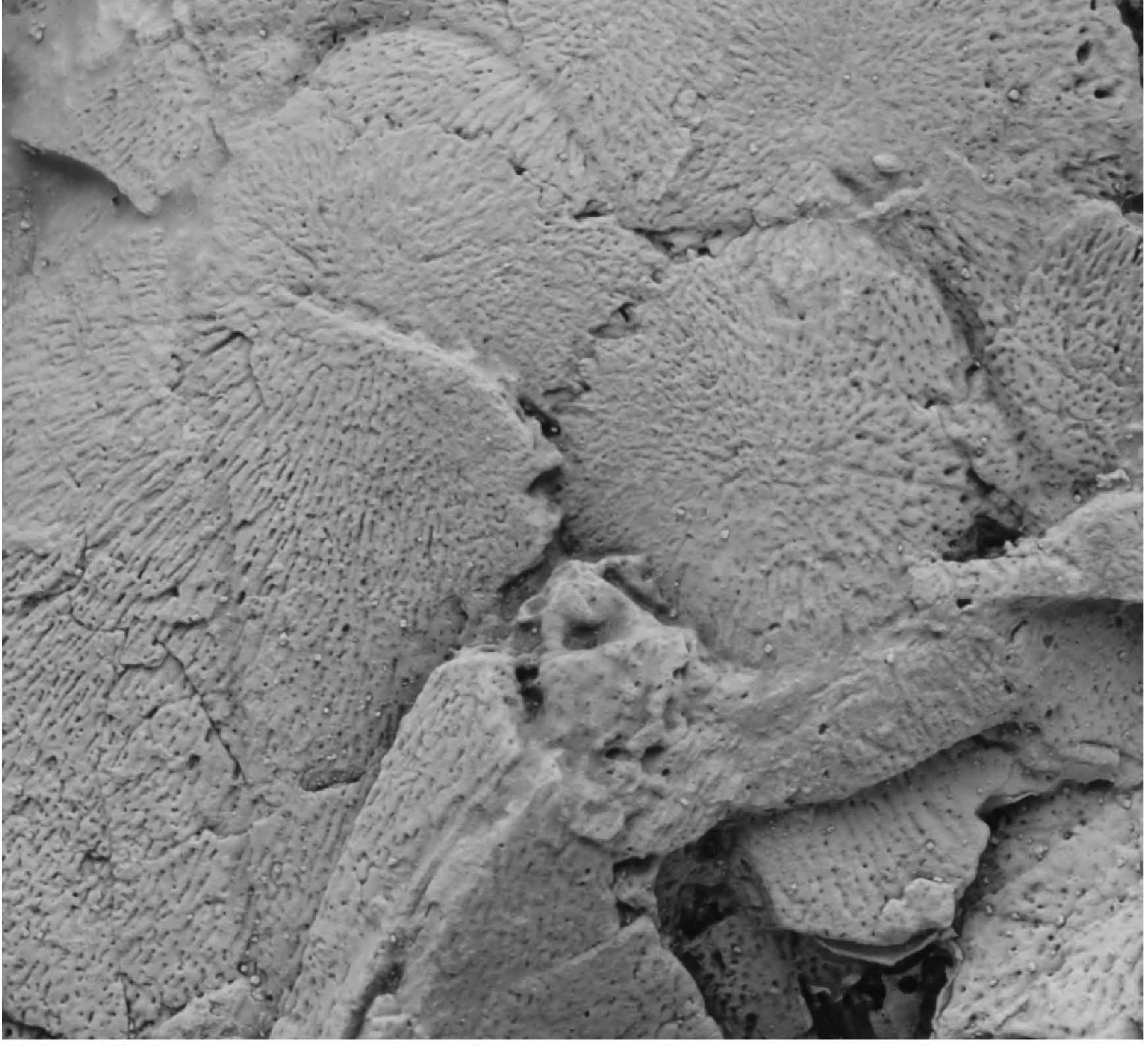

Skull interparietal scute/bone sculpture

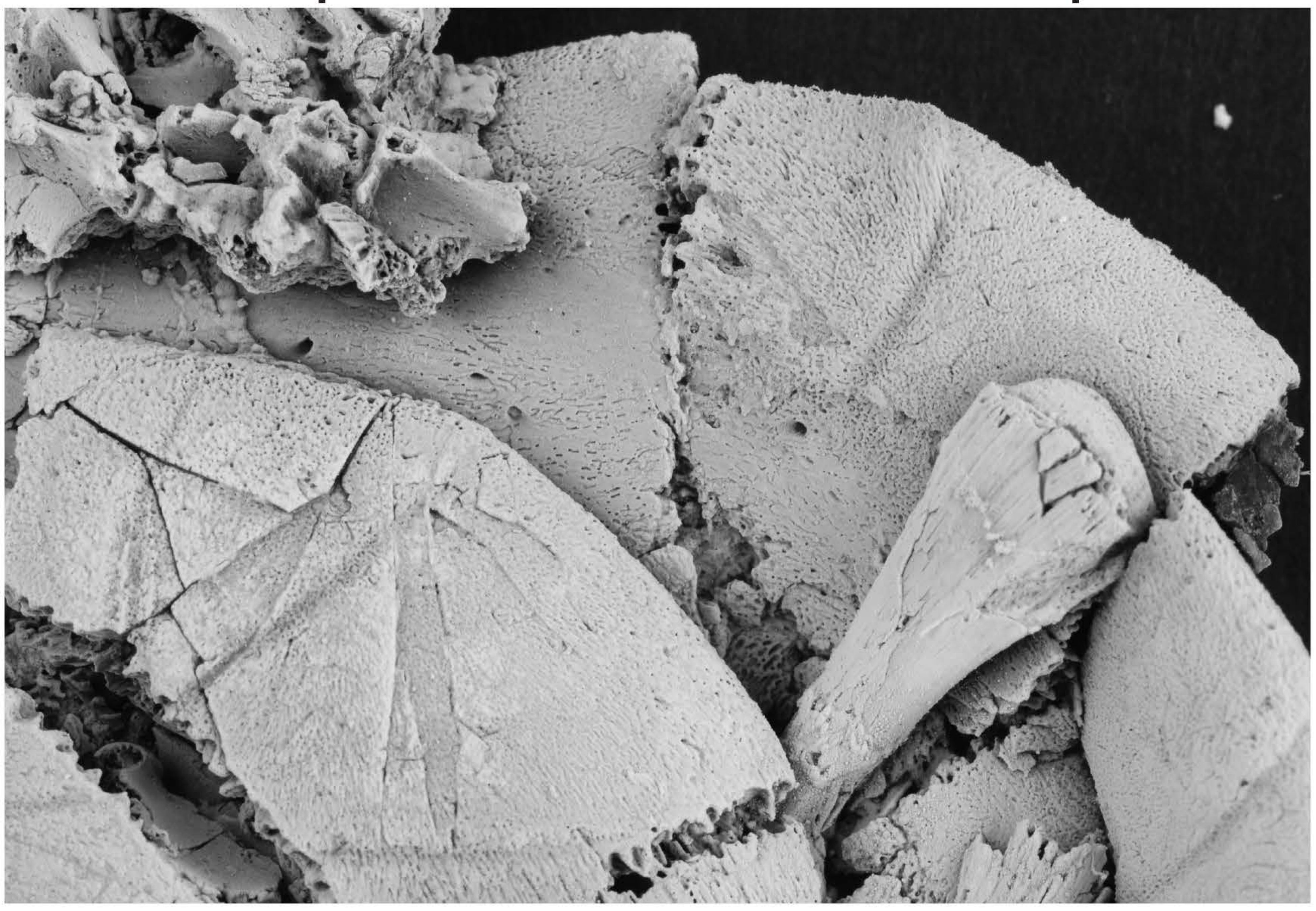

Left epiplastron, humerus, cervicals

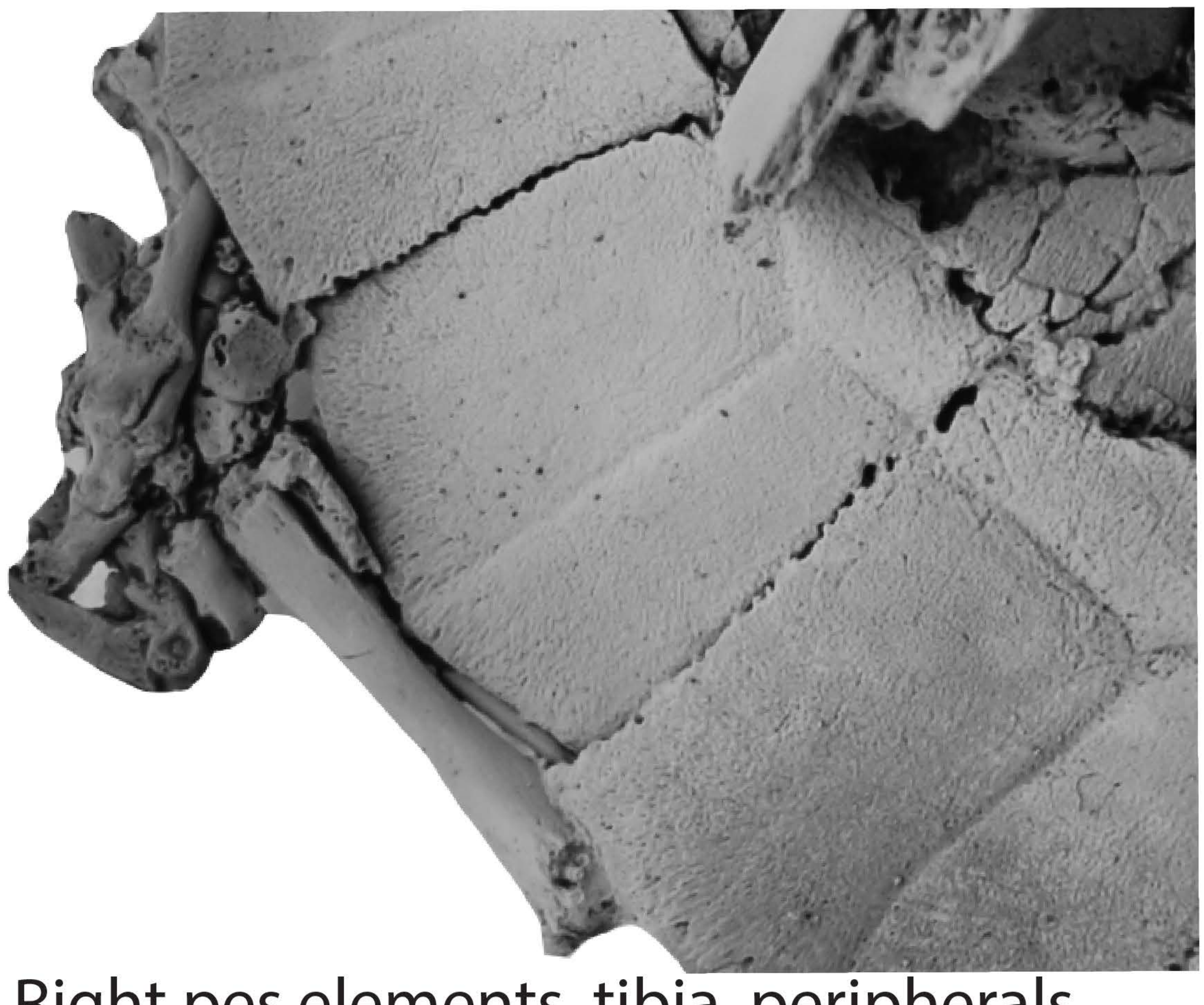

Right pes elements, tibia, peripherals

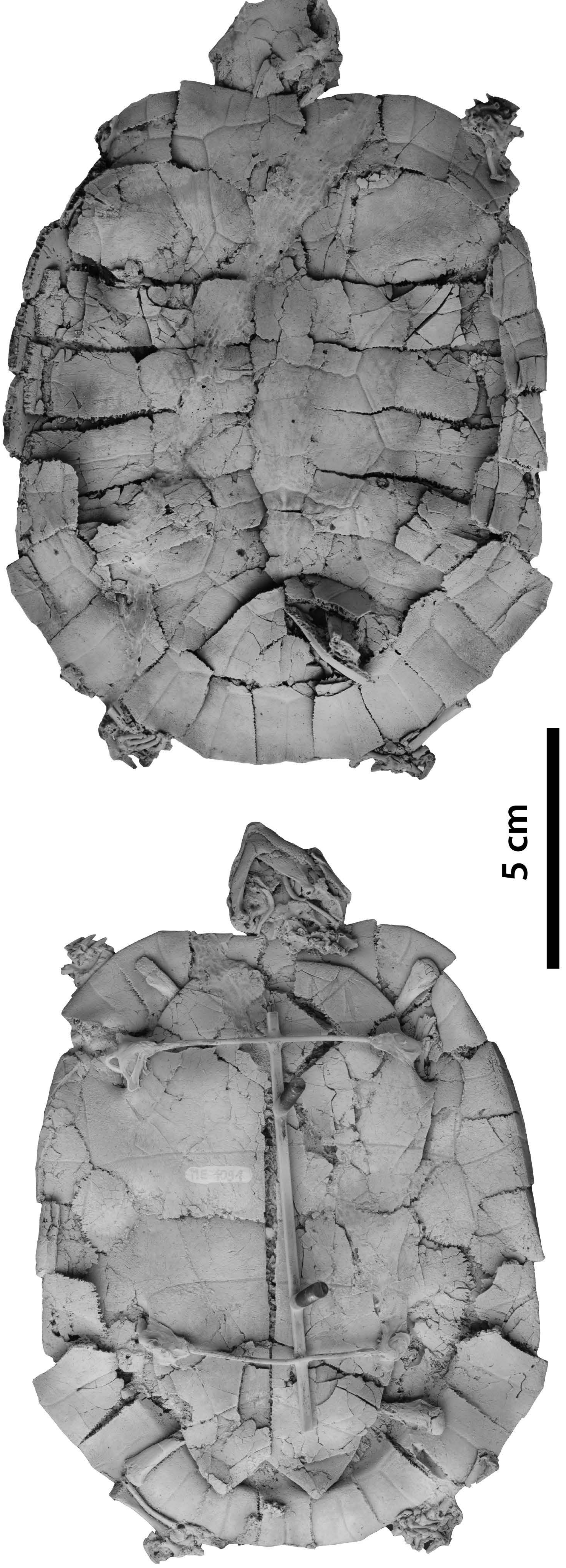

Plate 1. ***Neochelys franzeni*** **SMF ME 1091 (Holotype)**

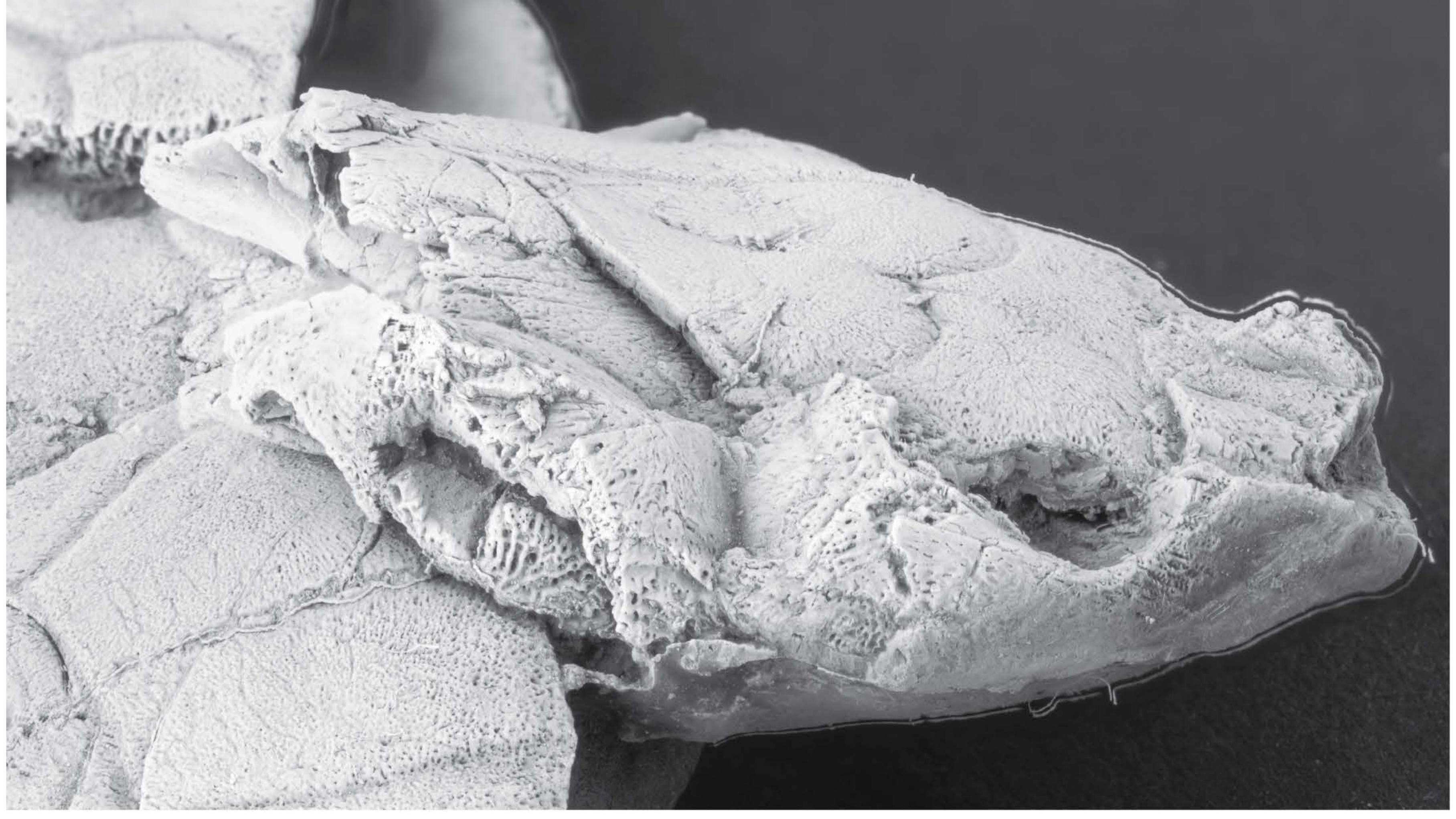

Skull lateral (right) view

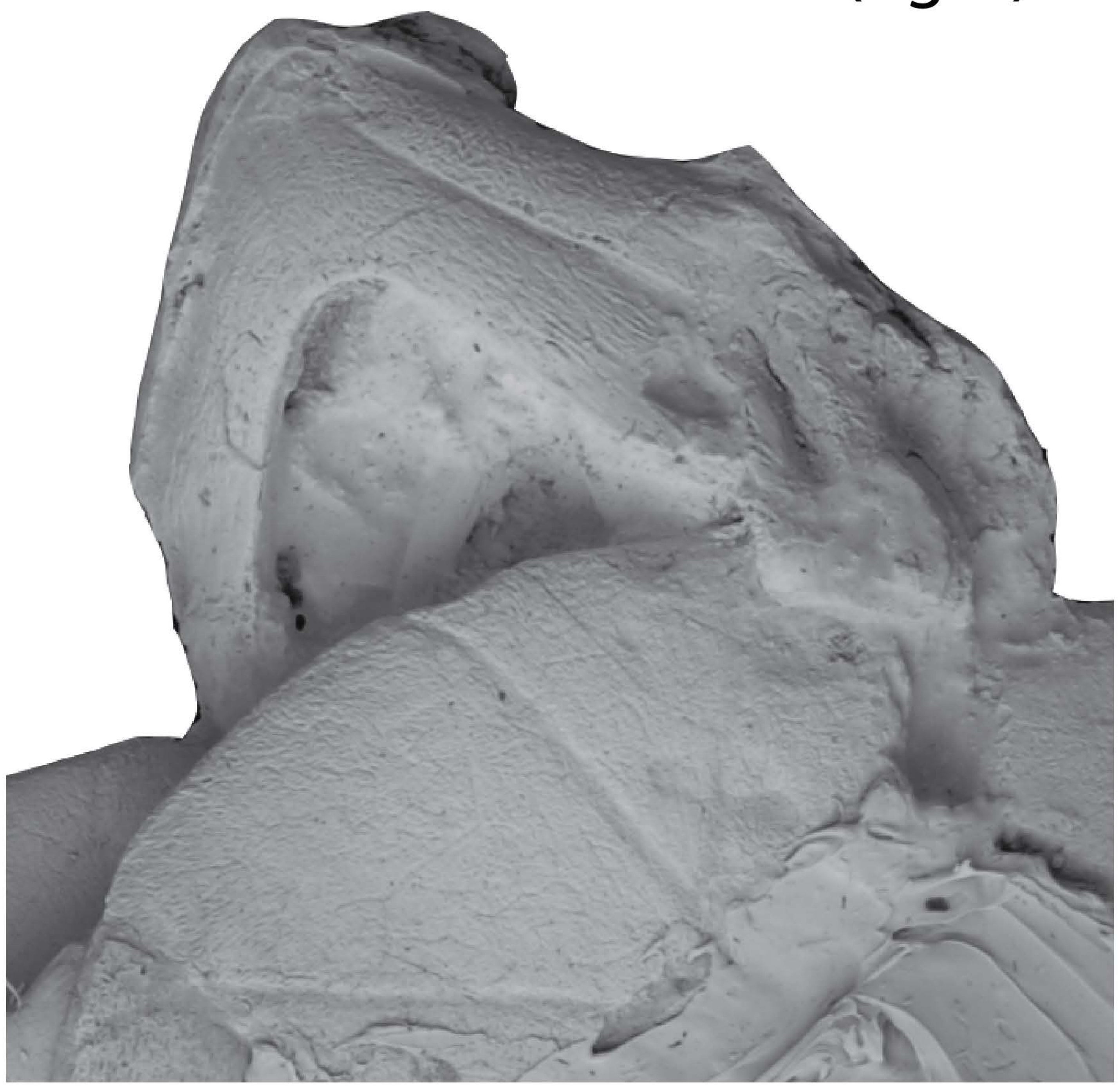

Skull ventral view

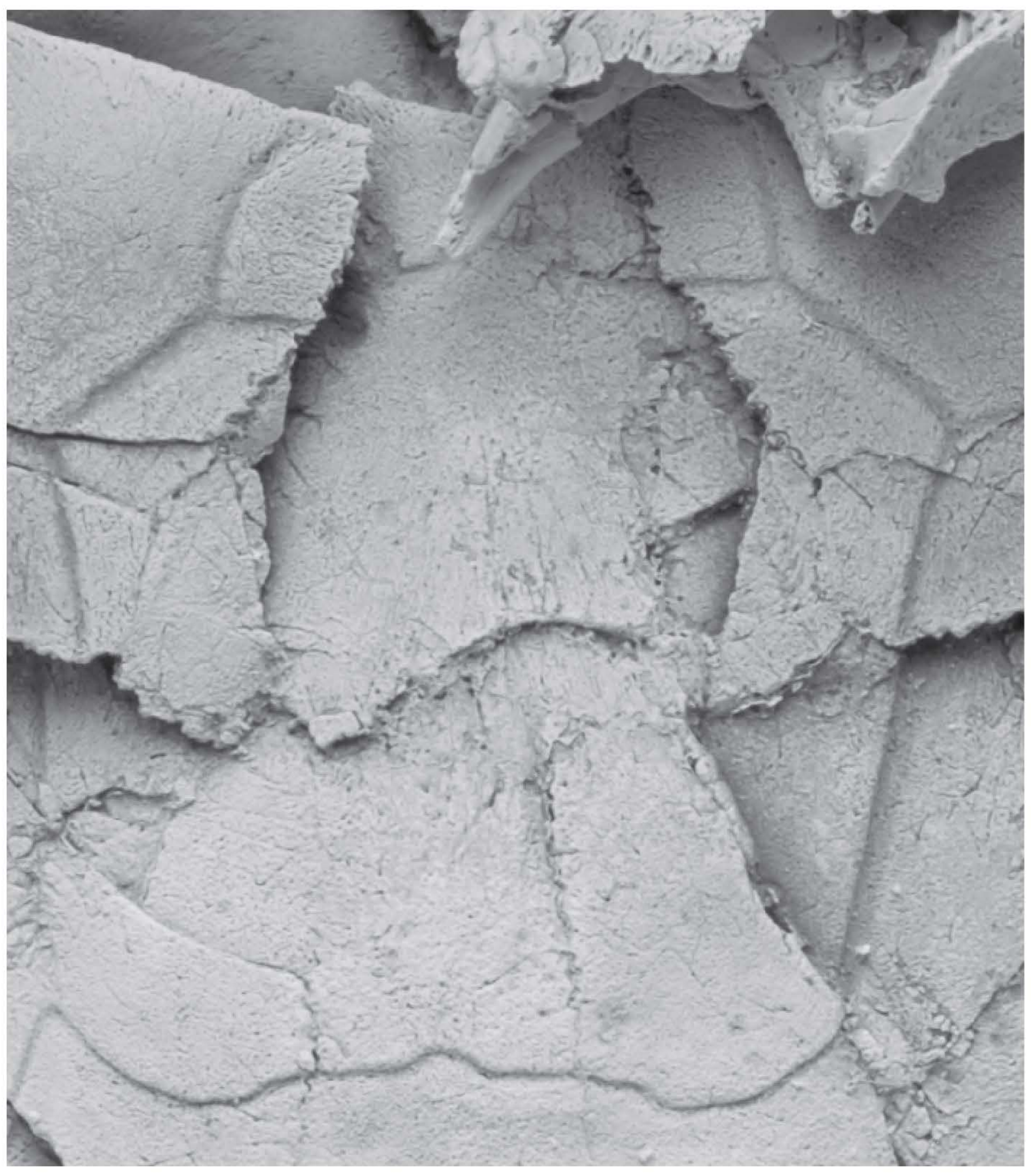

Nuchal/vertebral scute 1

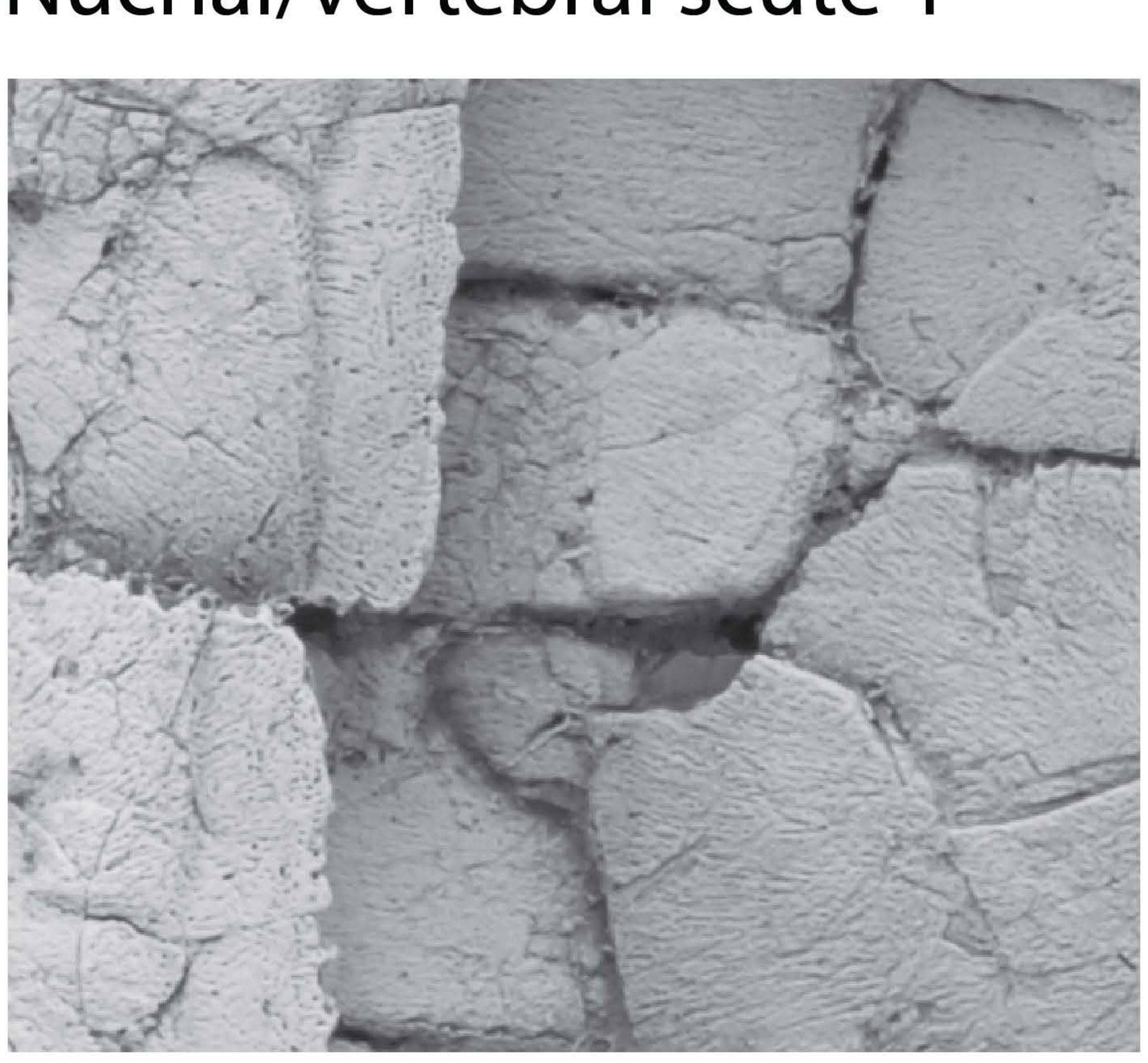

Peirpheral/costals dorsal scultpture

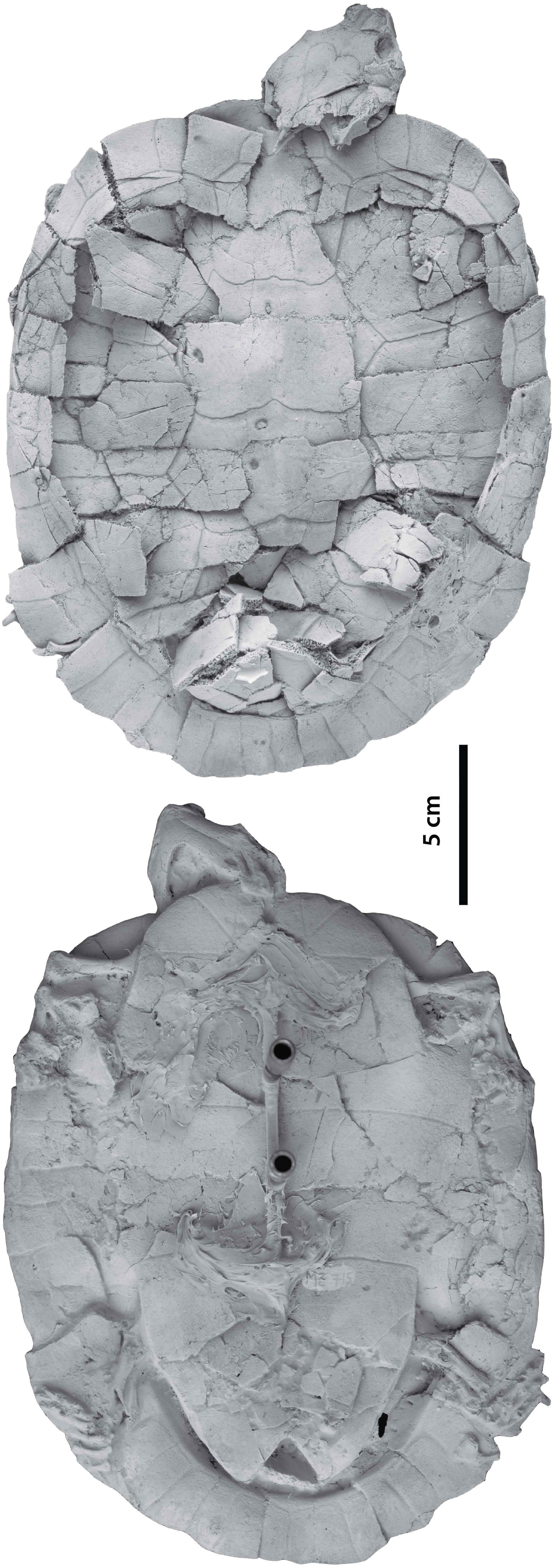

Plate 2. *Neochelys franzeni* SMF ME 715

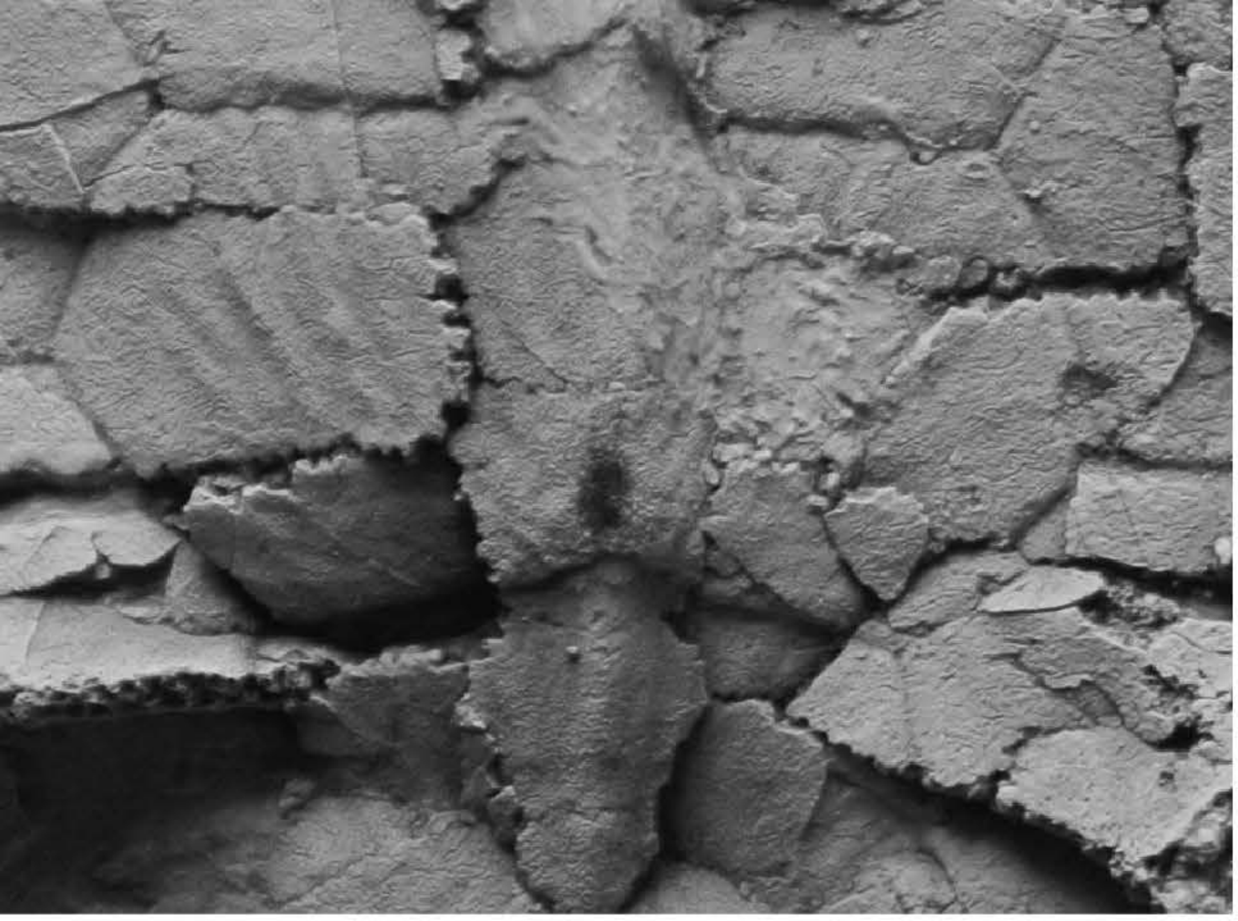

Vertebral 3, radial striations

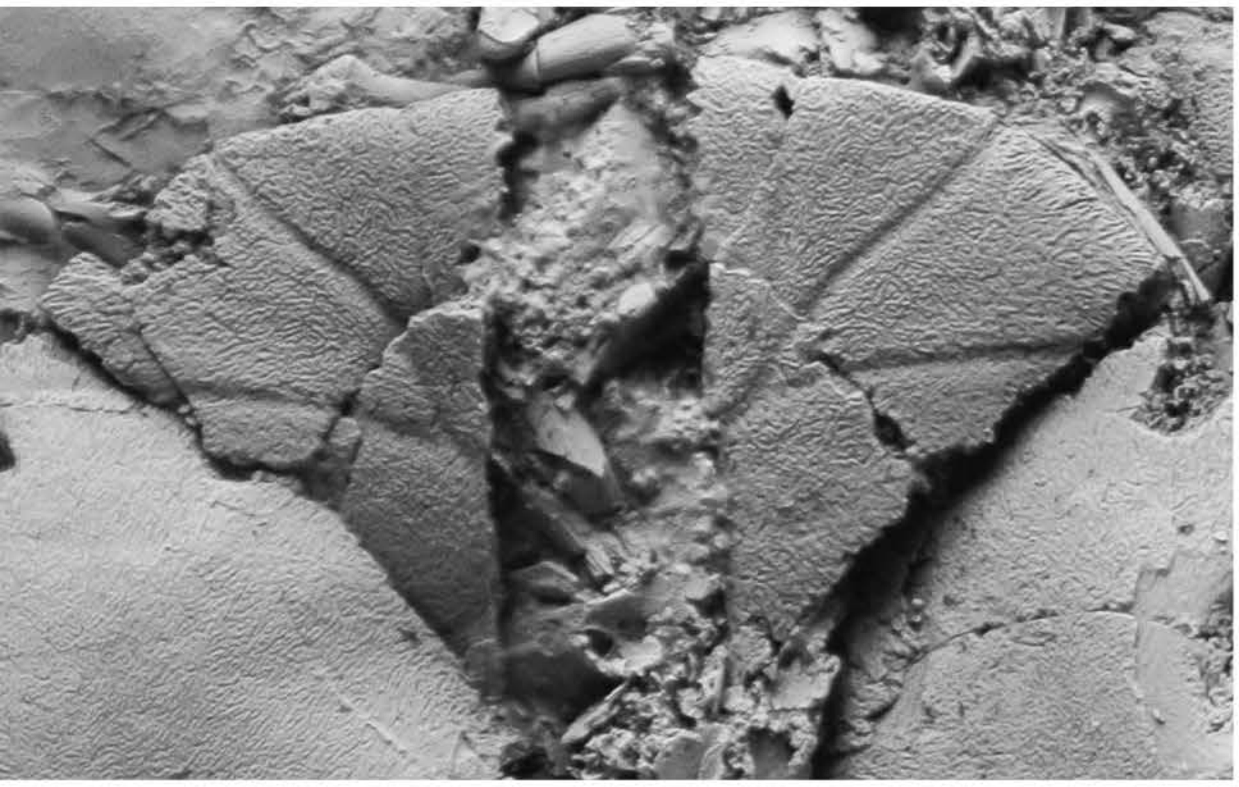

Epiplastra

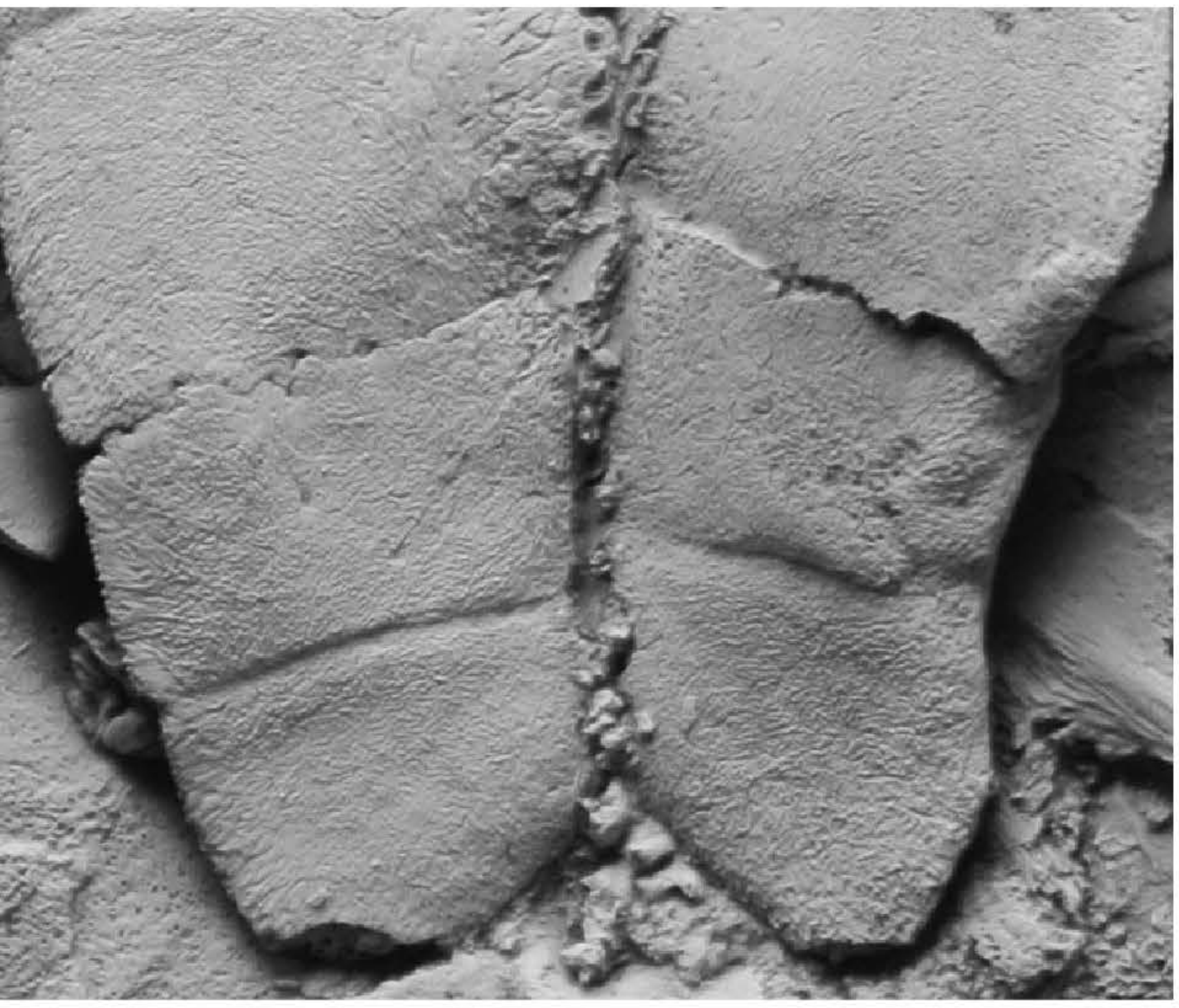

Xiphiplastra

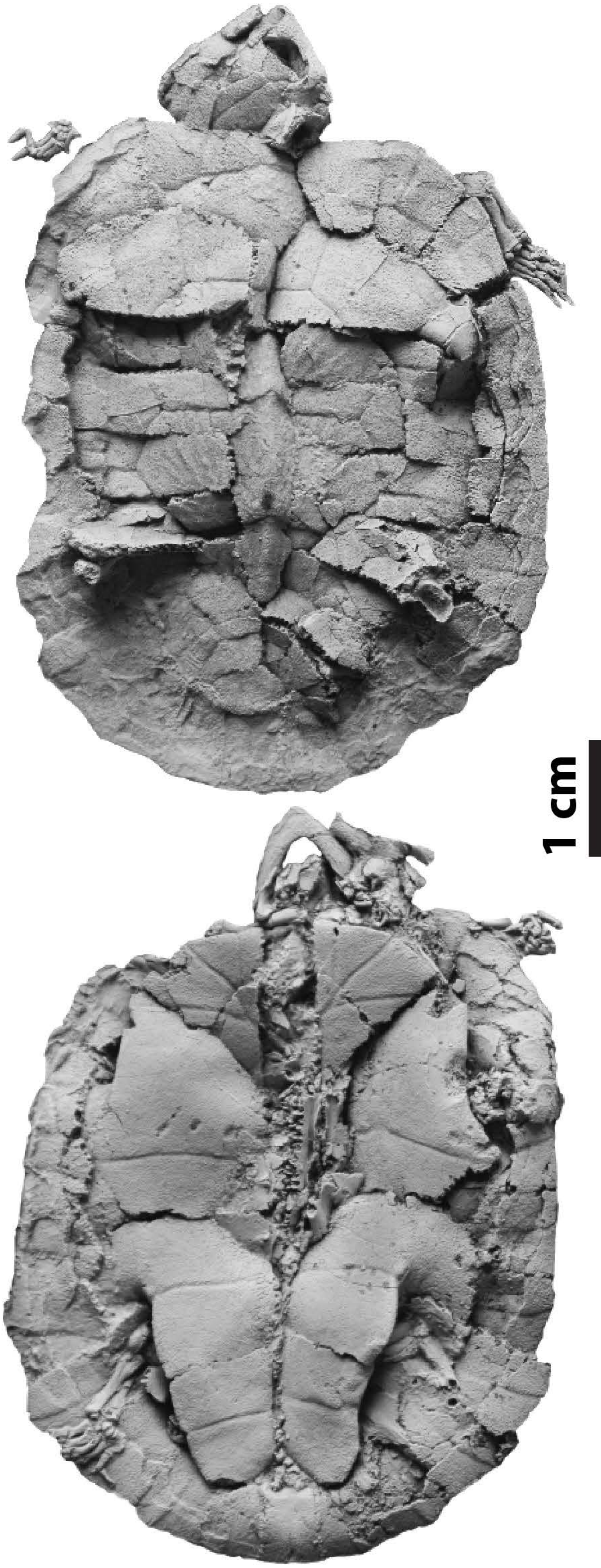

Plate 3. ***Neochelys franzeni*** **HLMD ME 14981**

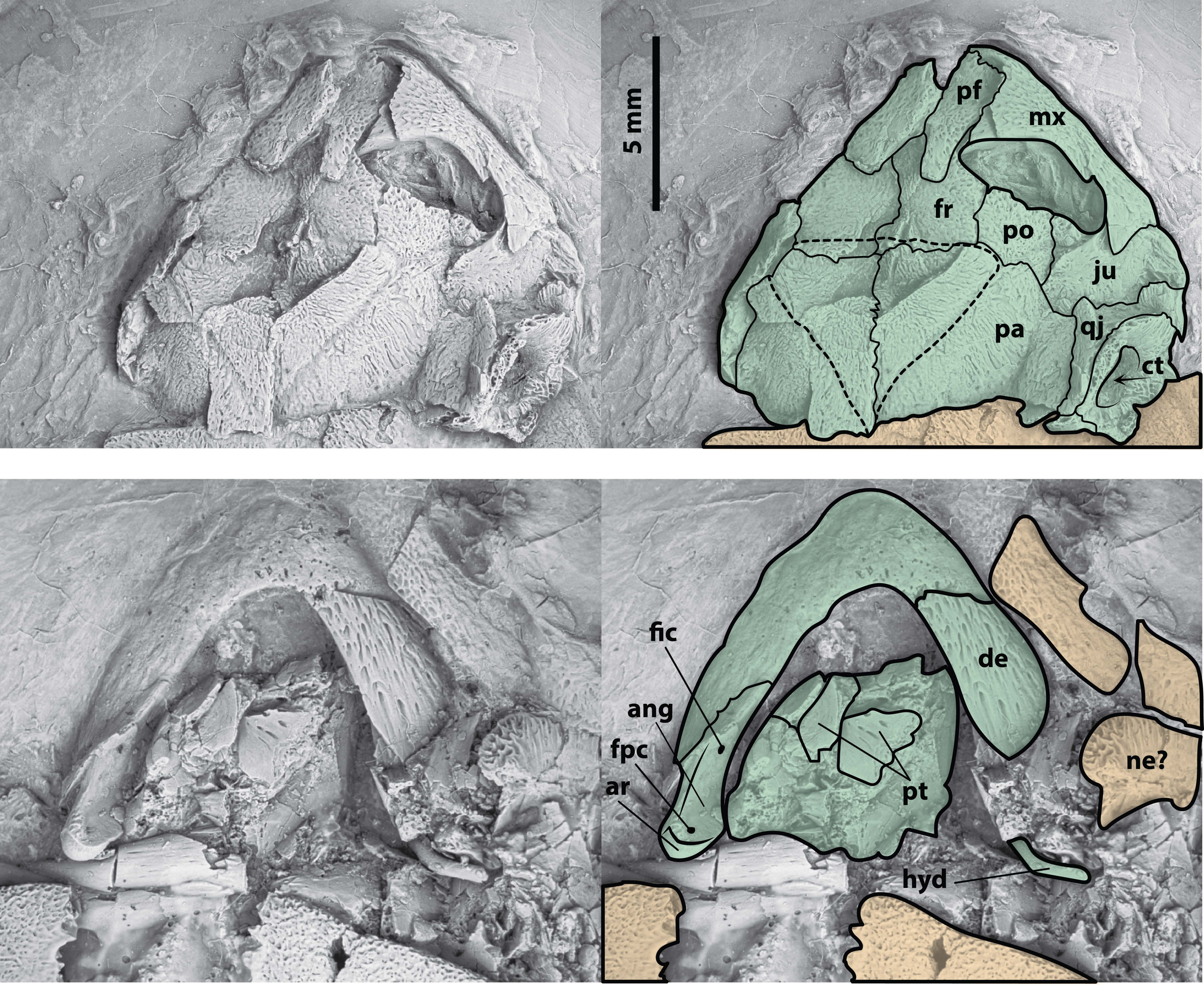

Plate 4. *Neochelys franzeni* HLMD ME 14981 skull, dorsal(top), ventral (bottom)

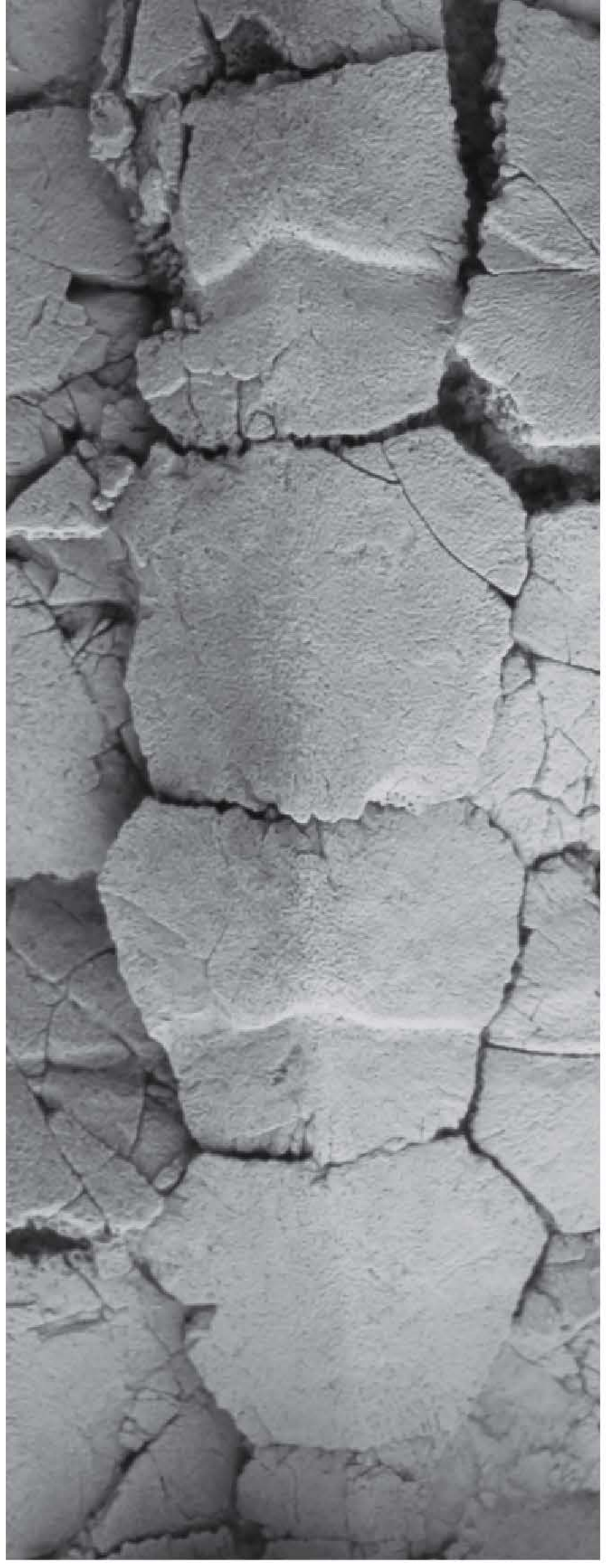

Neurals and the medial ridge

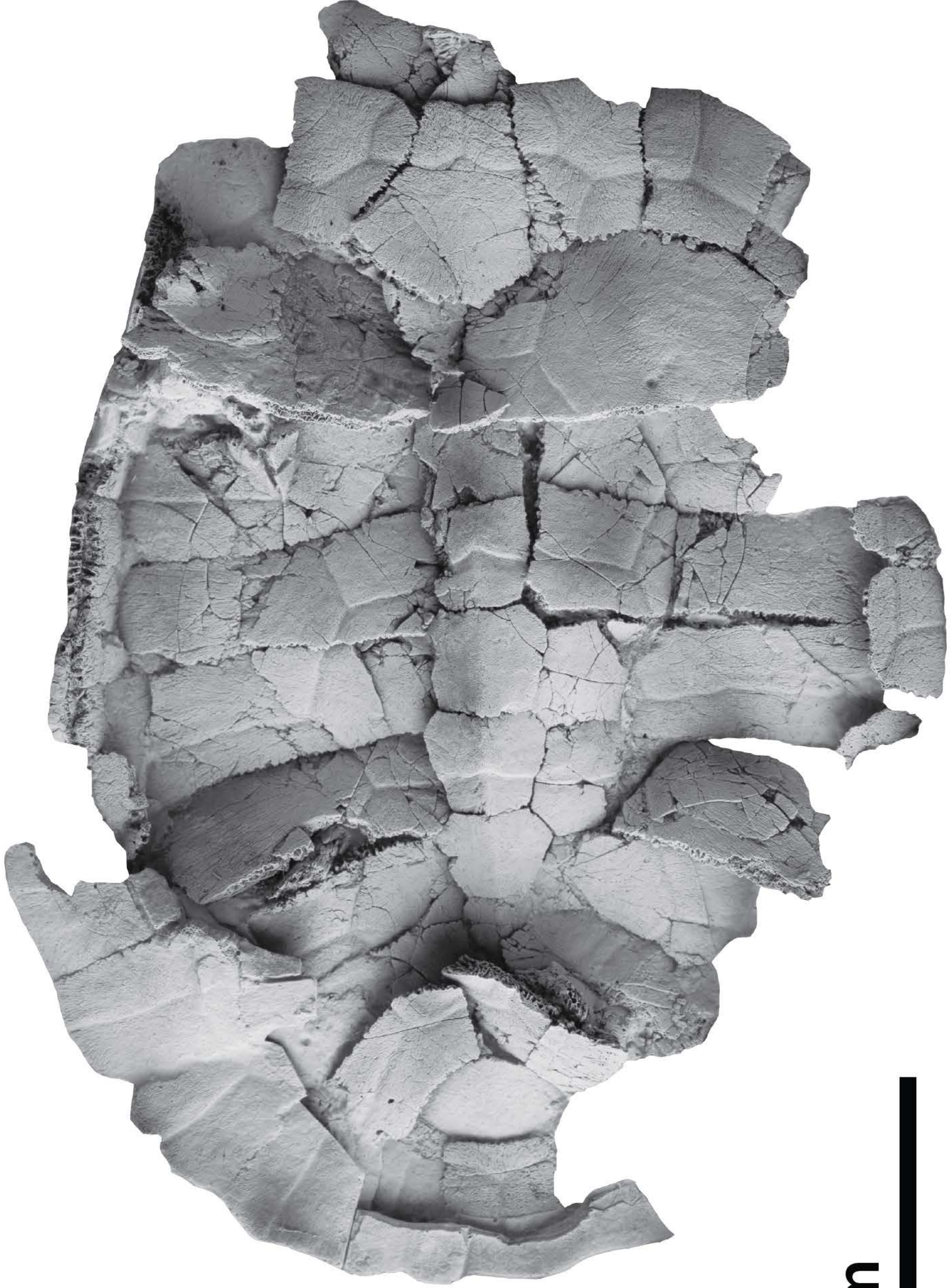

5 cm

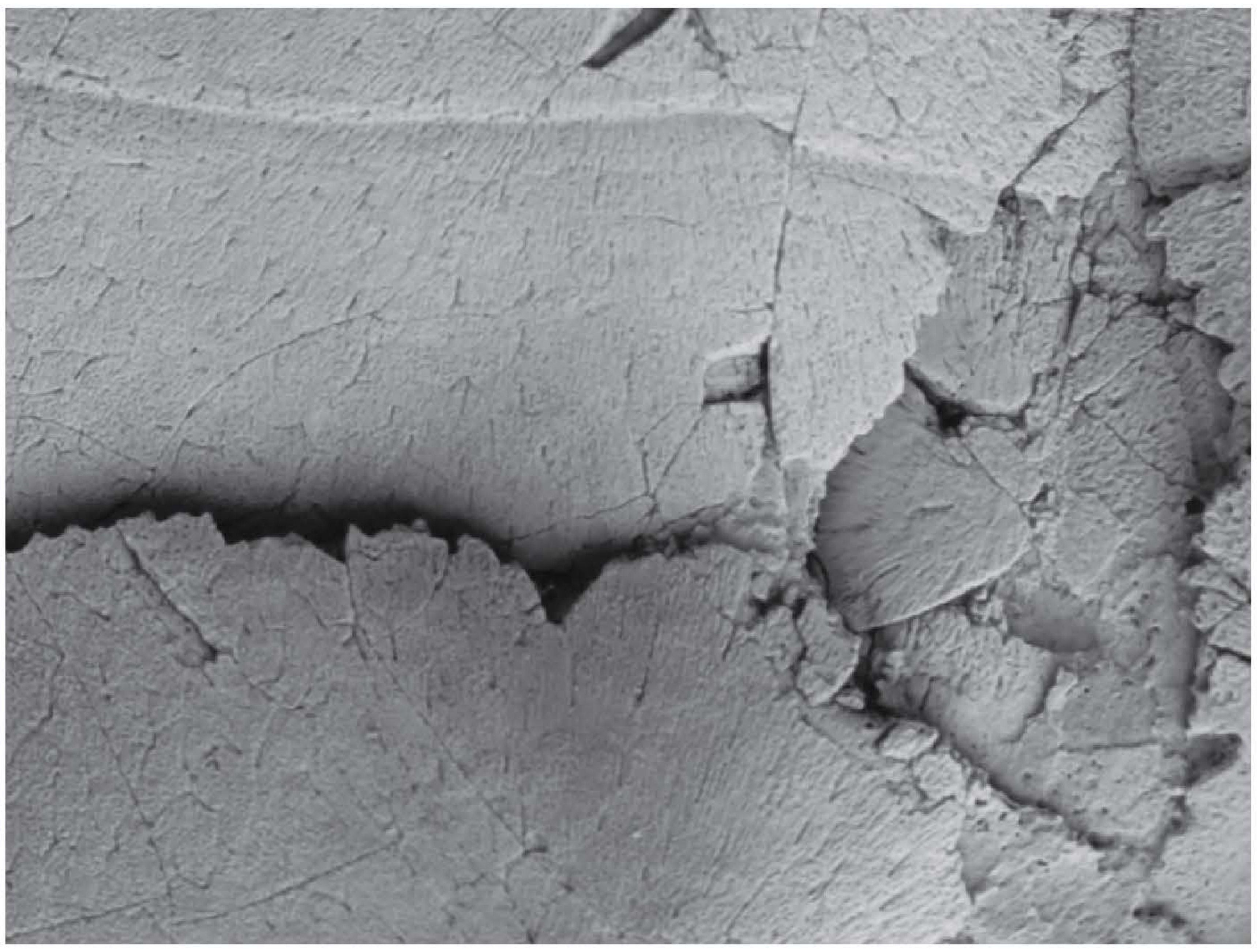

Left mesoplastron, pec-abd sulcus

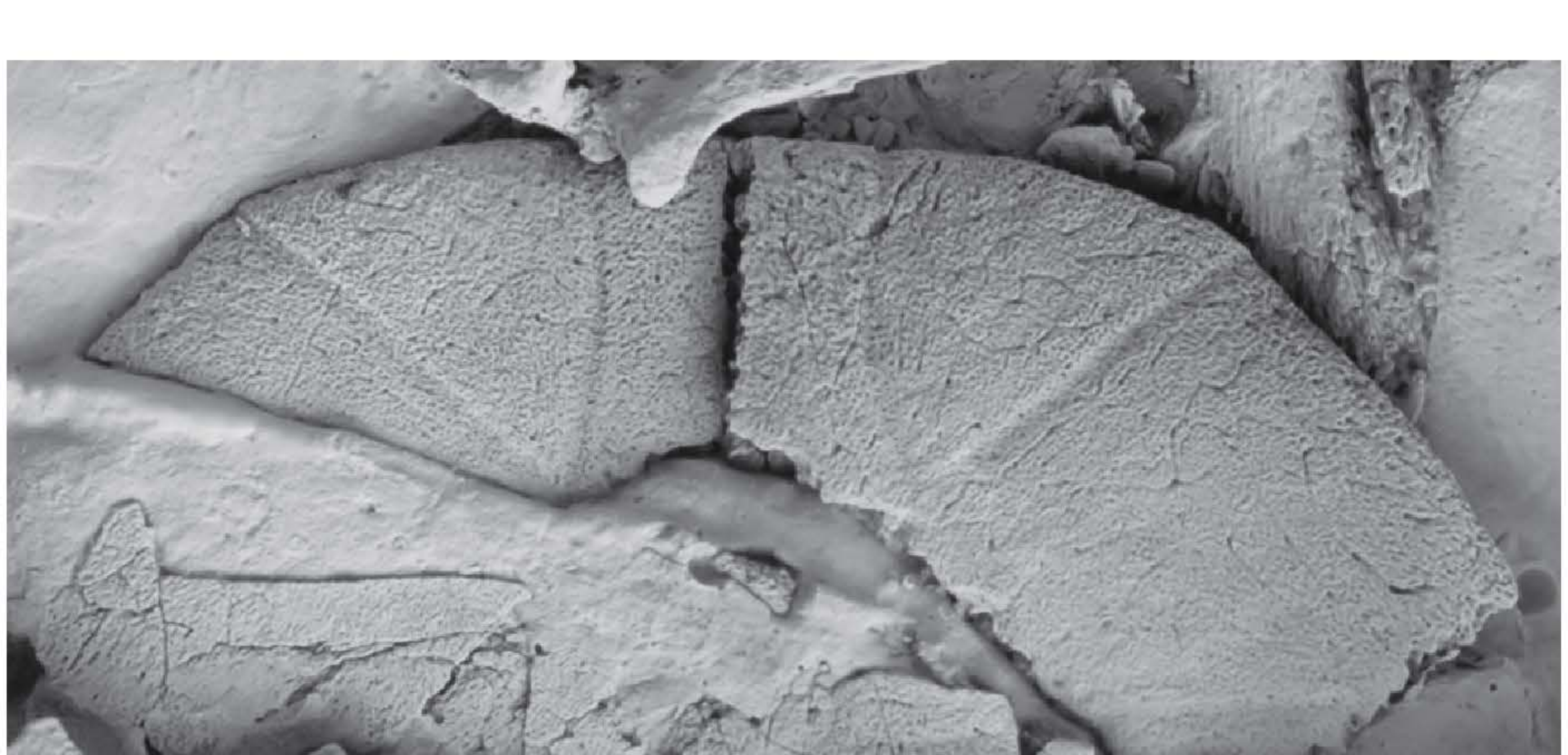

Epiplastra

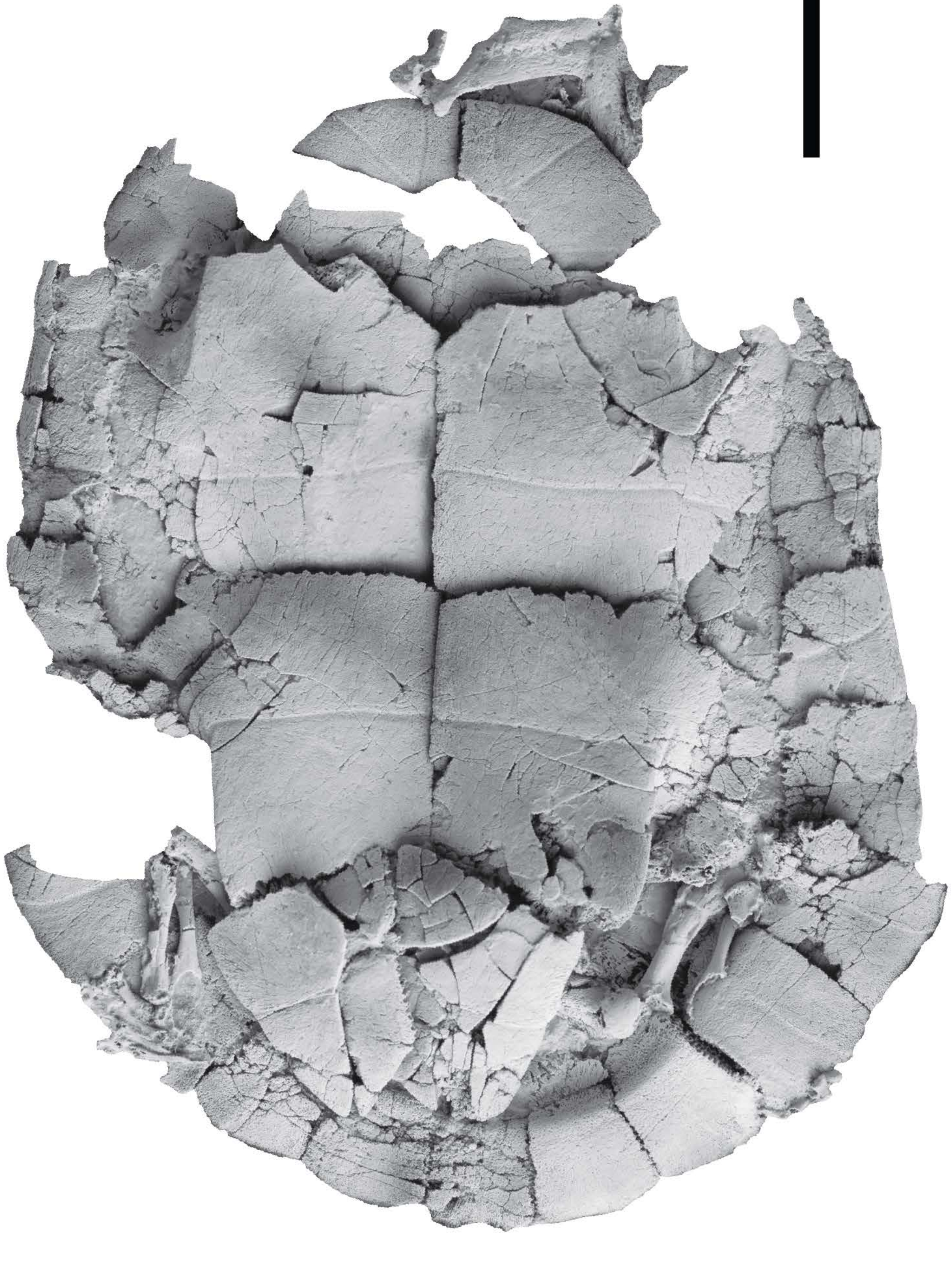

Plate 5. *Neochelys franzeni* HLMD ME 115576

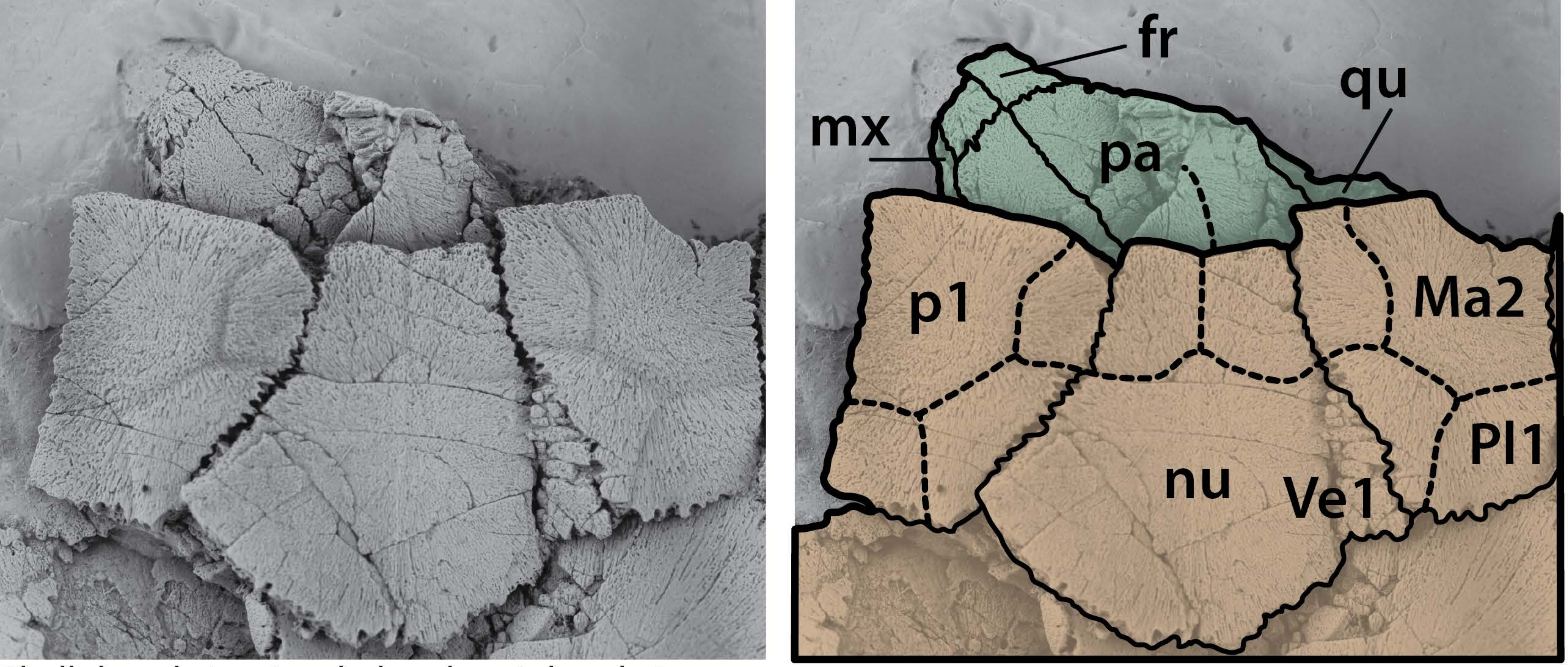

Skull dorsal view/nuchal and peripherals 1

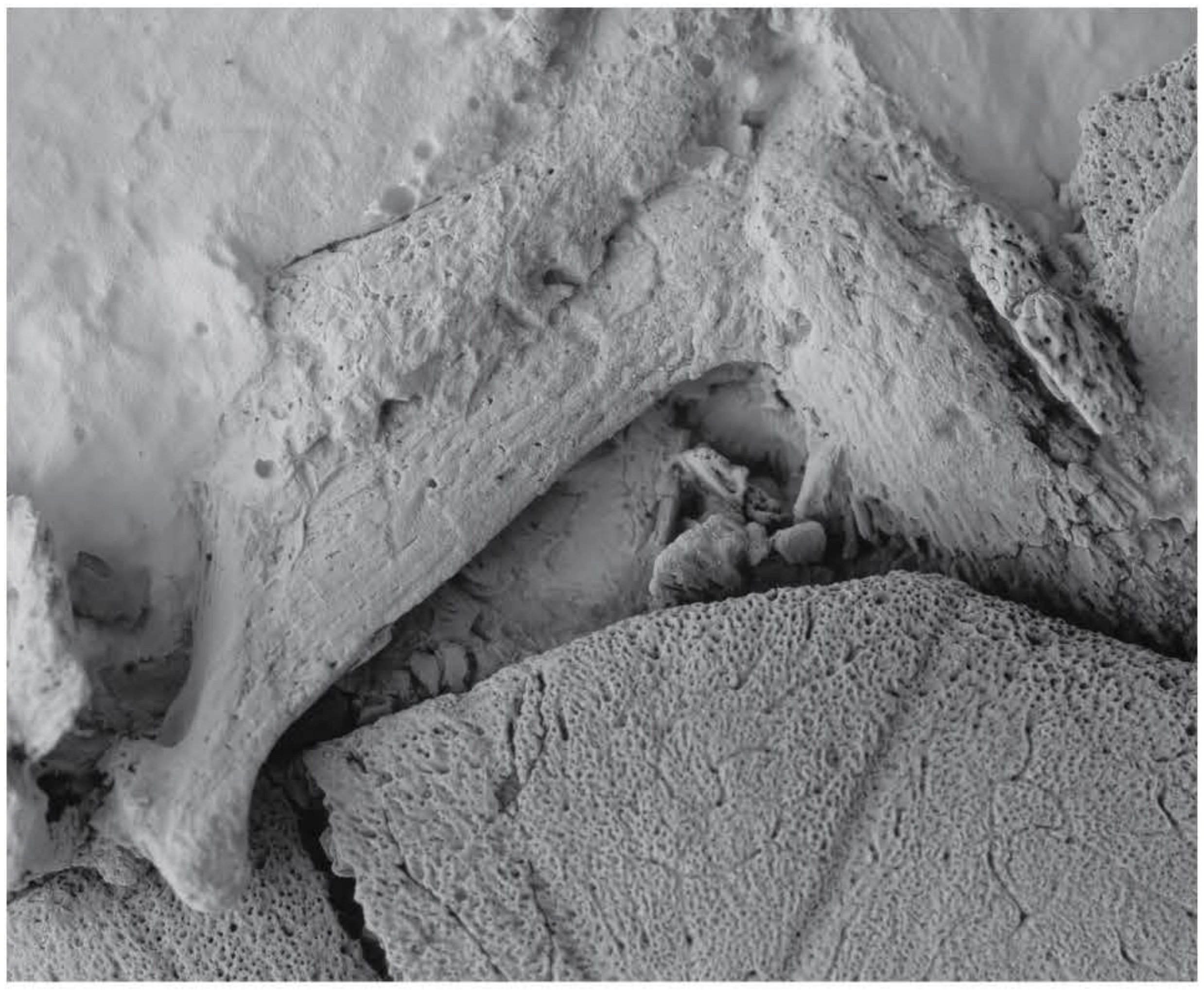

Lower jaw

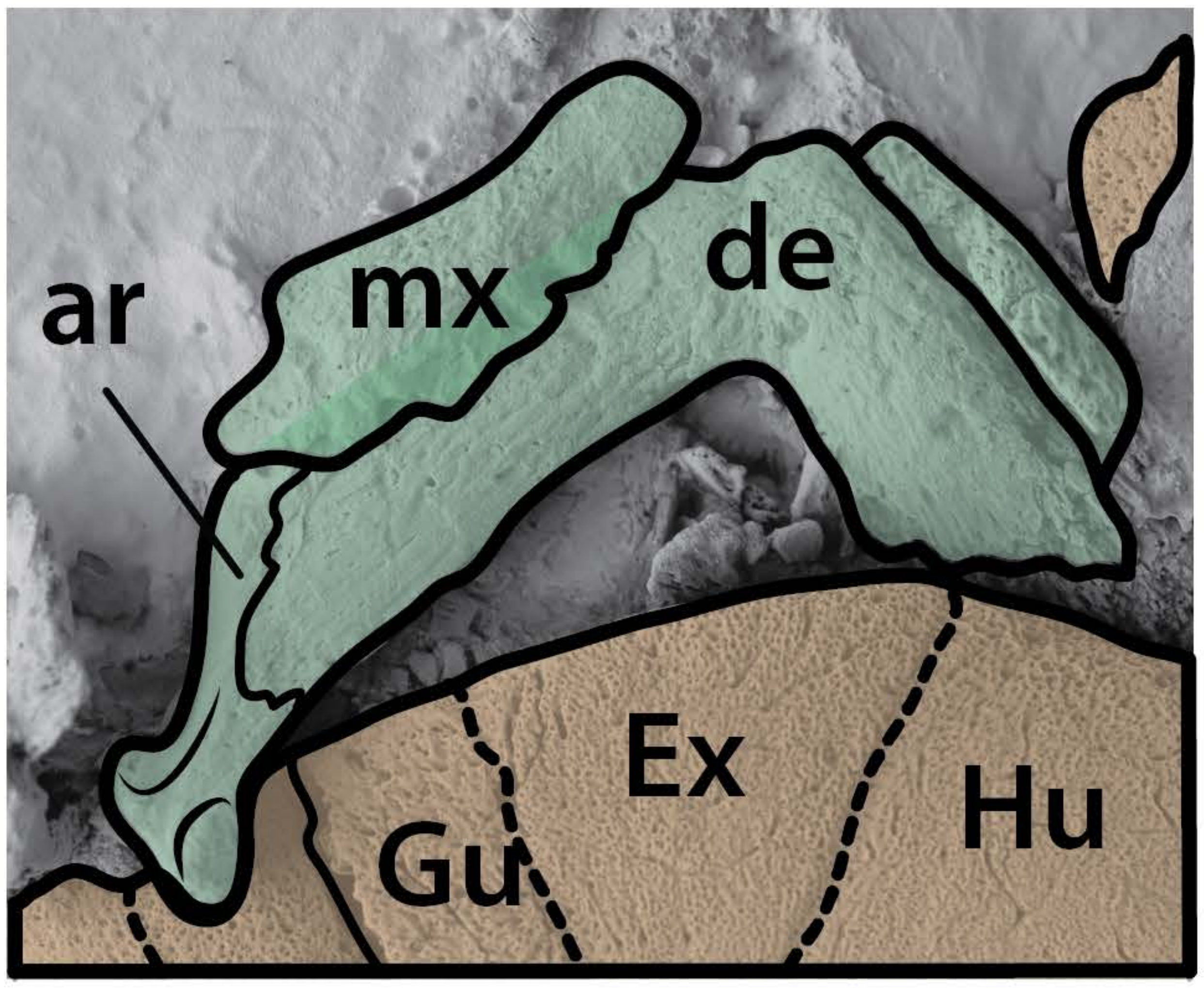

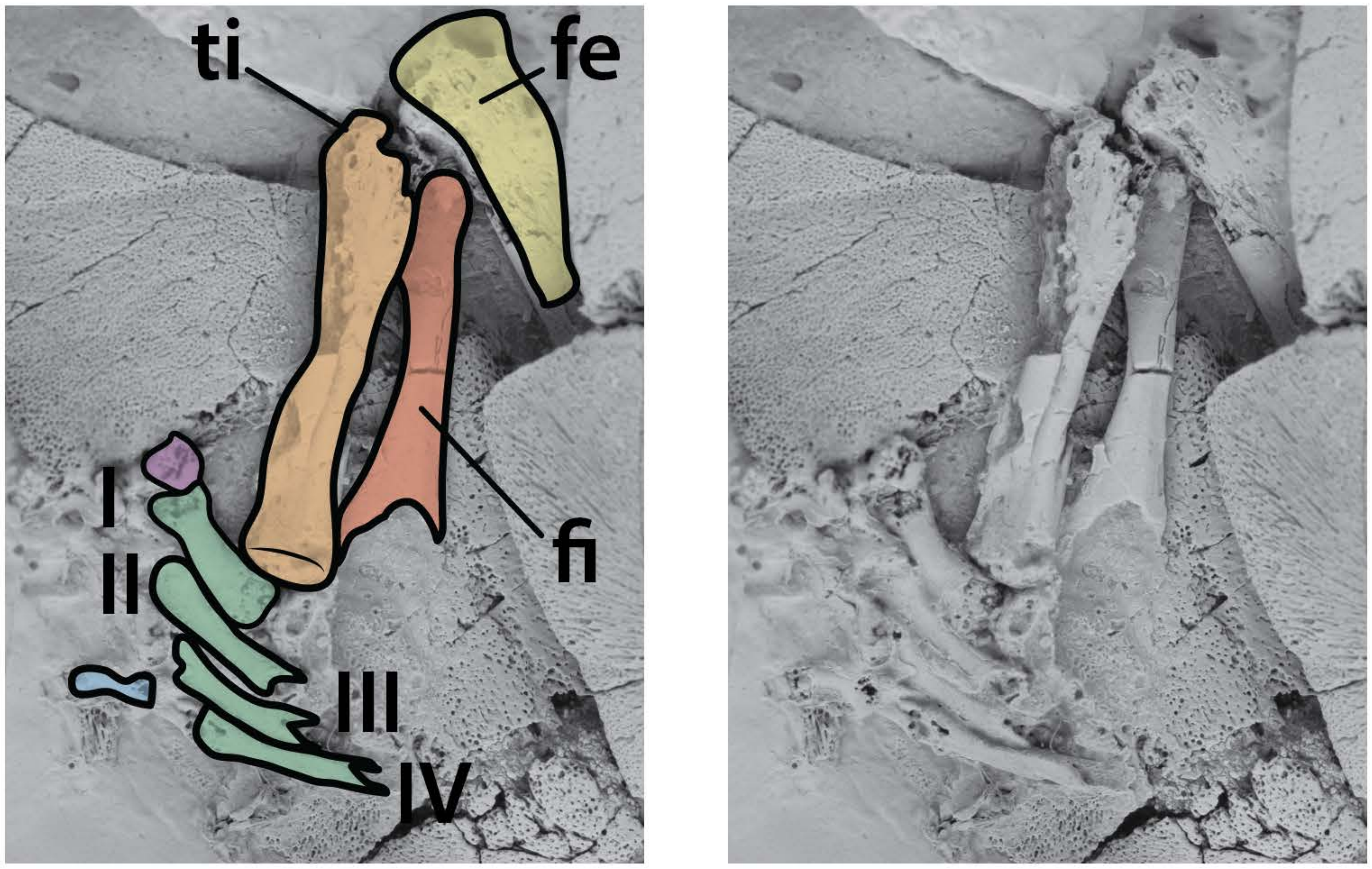

Right pes elements(top)-left femur-fibula (bottom)

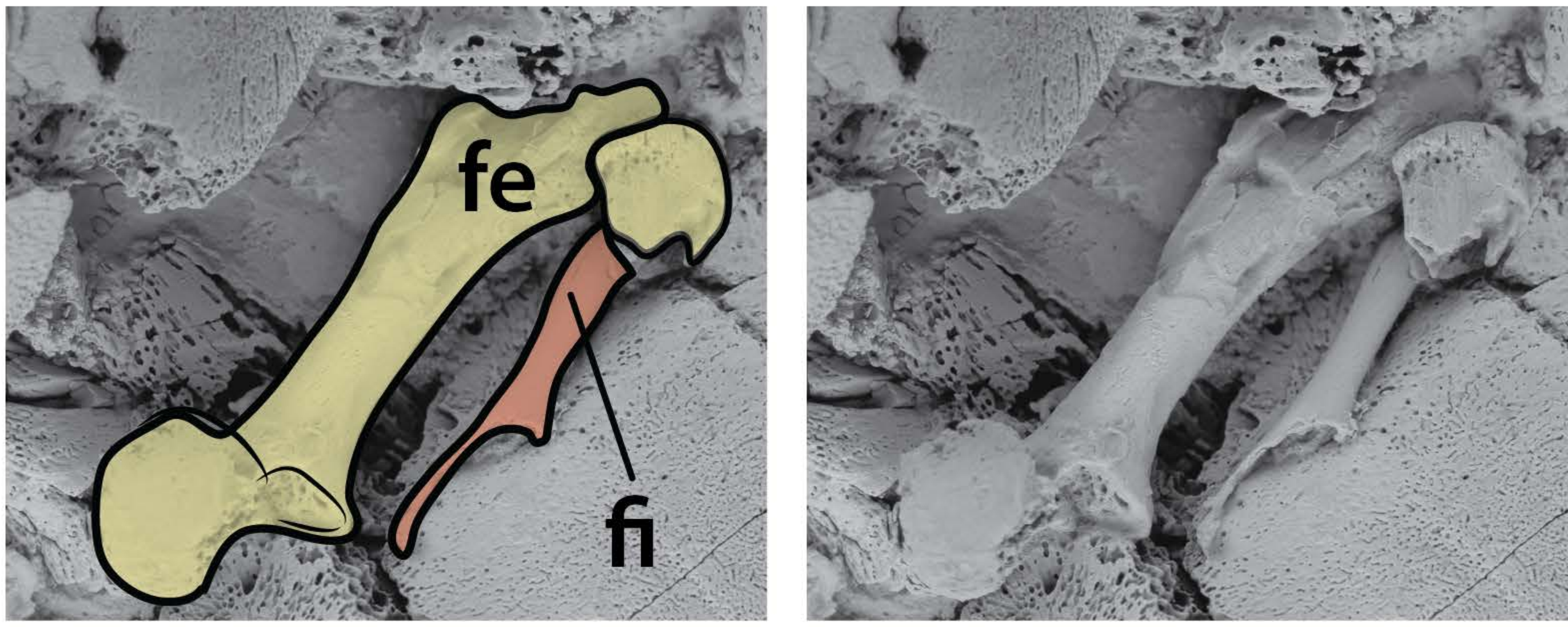

Plate 6. *Neochelys franzeni* HLMD ME 15576

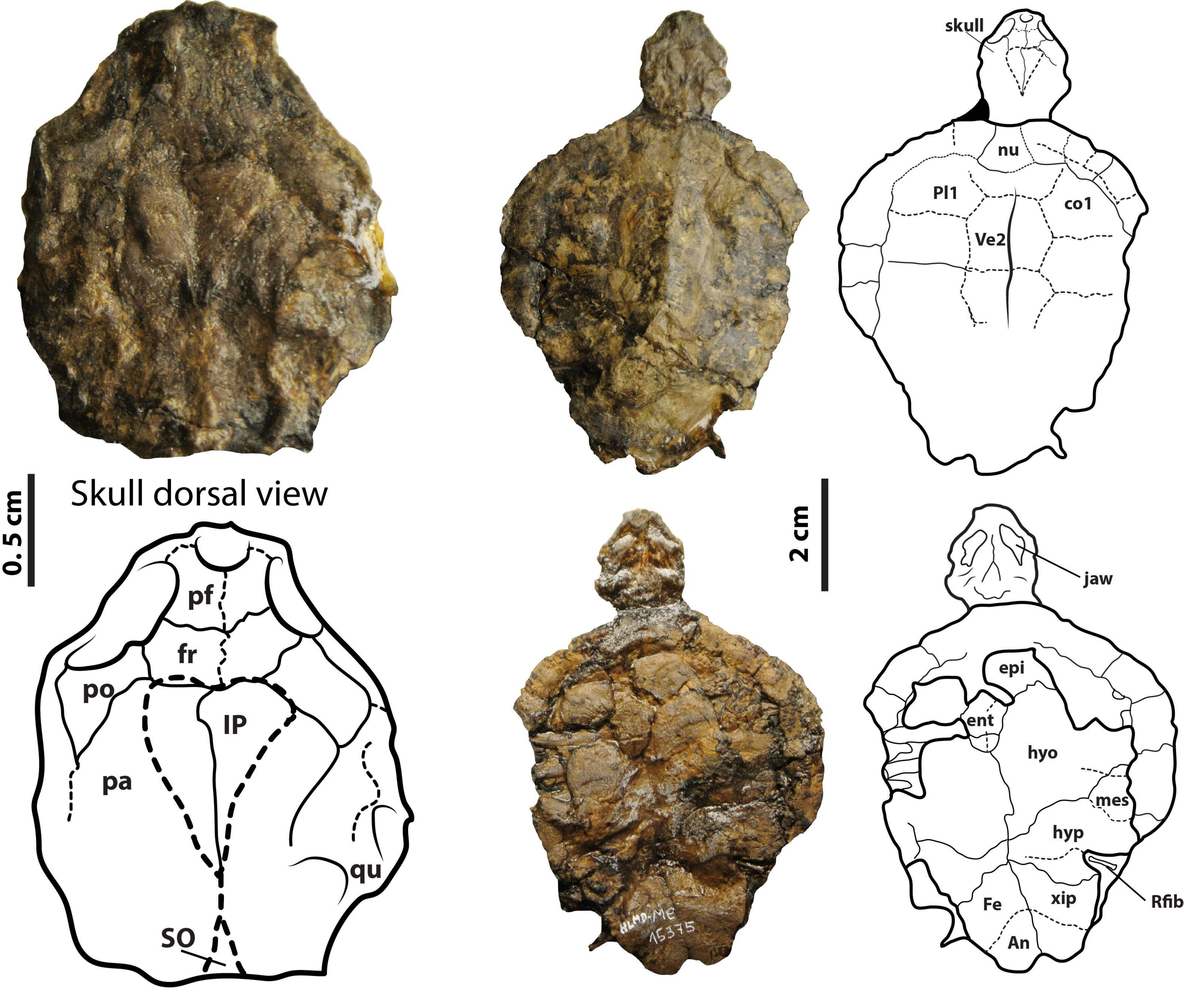

Plate 7. ***Neochelys franzeni*** **HLMD ME 15375**

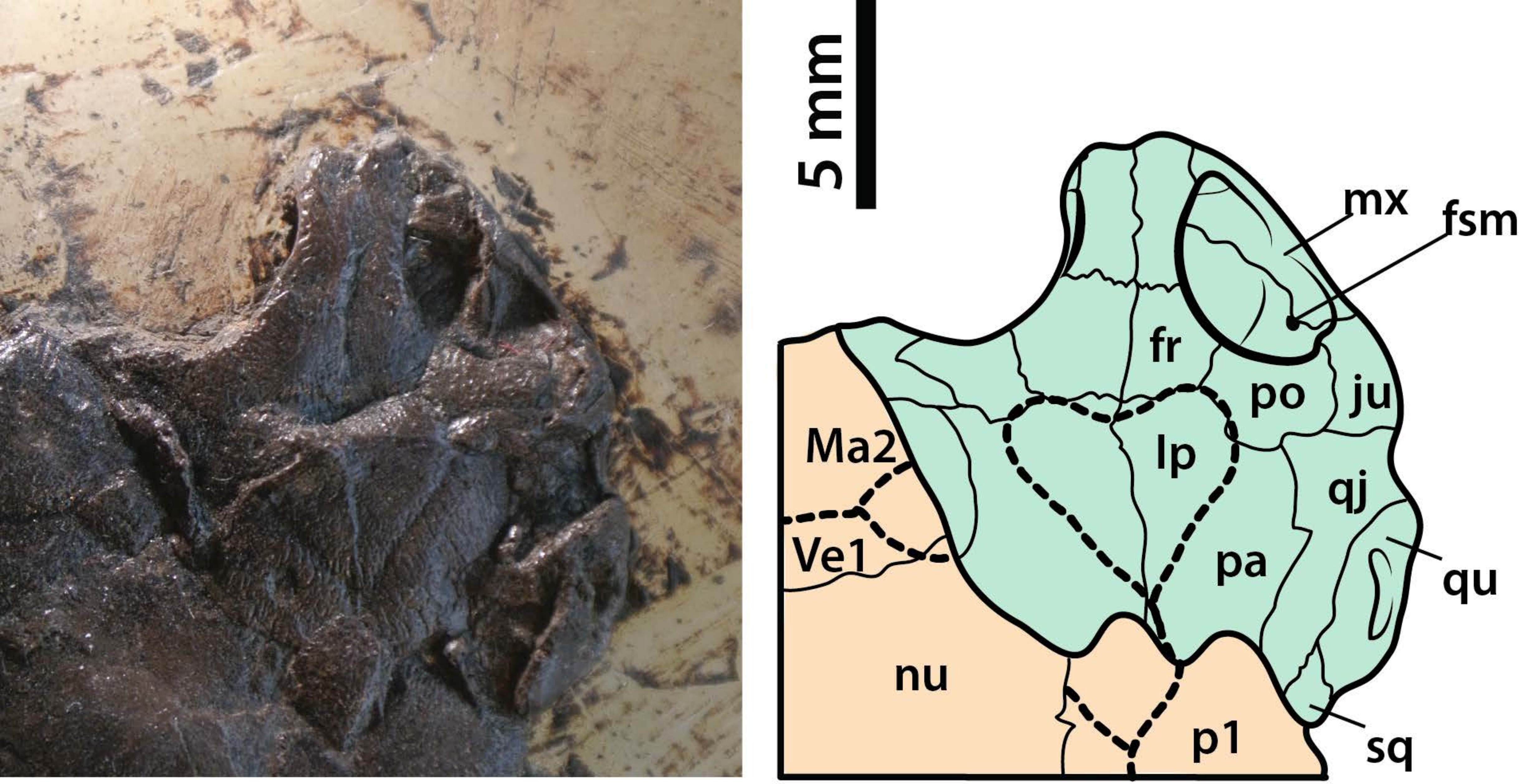

Plate 8. *Neochelys franzeni* SNR 202/617
