# Supplementary material for: A global phylogeny of Pelomedusoides turtles with new material of Neochelys franzeni Schleich, 1993 (Testudines, Podocnemididae) from the middle Eocene, Messel Pit, of Germany
Source: PeerJ. 2015 Aug 27;3:e1221. doi: 10.7717/peerj.1221 (PMC4556147; doi:10.7717/peerj.1221)
Supplement: Supplemental Information 4 — Photographs or figures are not to original scale. Plate 1. Podocnemididae skulls in dorsal view. Plate 2. Podocnemididae skulls in ventral view. Plate 3. Podocnemididae skulls in left lateral view. Plate 4. Carapace outline of Neochelys species and some extant podocnemidids. Plate 5. Vertebral scute 1 of Neochelys species and some extant podocnemidids. Plate 6. Anterior plastral bone and scutes for some Neochelys species and some extant podocnemidids. Plate 7. Strict consensus tree when all taxa and all morphological characters from character-taxon matrix are included in a new technology search, TNT. List 1. List of specimens of extant species directly examined. [file peerj-03-1221-s004.pdf]

Plate 1. Podocnemididae- skulls dorsal view

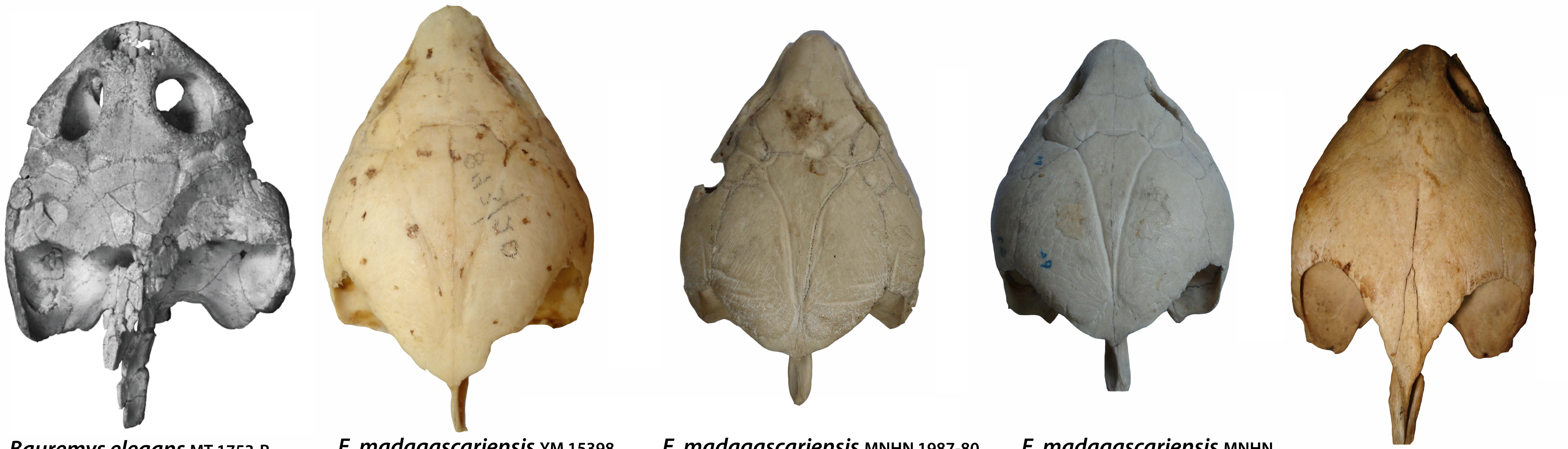

Bauremys elegans MT 1753-R

E. madagascariensis YM 15398

E. madagascariensis MNHN 1987-80

E. madagascariensis MNHN

Podocenemis expansa ICN

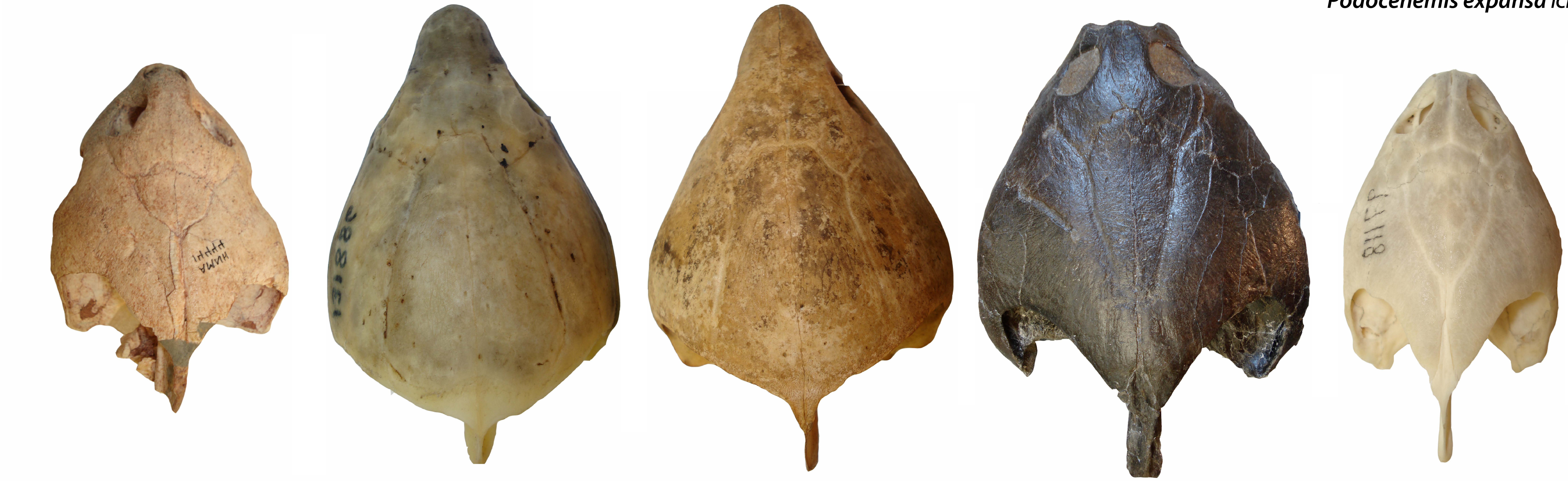

Lapparentemys vilavilensis AMNH 14444

Pe. dumerilianus NFWFL 336

Pe. dumerilianus AMNH 131886

Podocnemis bassleri AMNH 1662

Podocnemis unifilis AMNH 97118

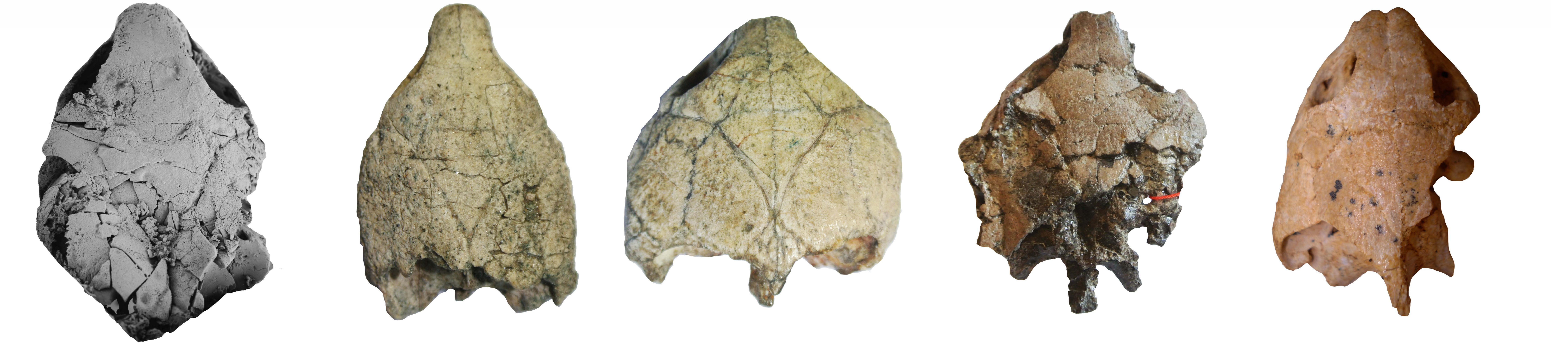

Neochelys franzeni SMF ME 1267

Neochelys arenarum MNHN RI 7

Neochelys arenarum MNHN RI 6

Neochelys laurenti MNHN SPP29

"Neochelys" fajumensis DPC 3146

Plate 2. Podocnemidididae- skulls ventral view

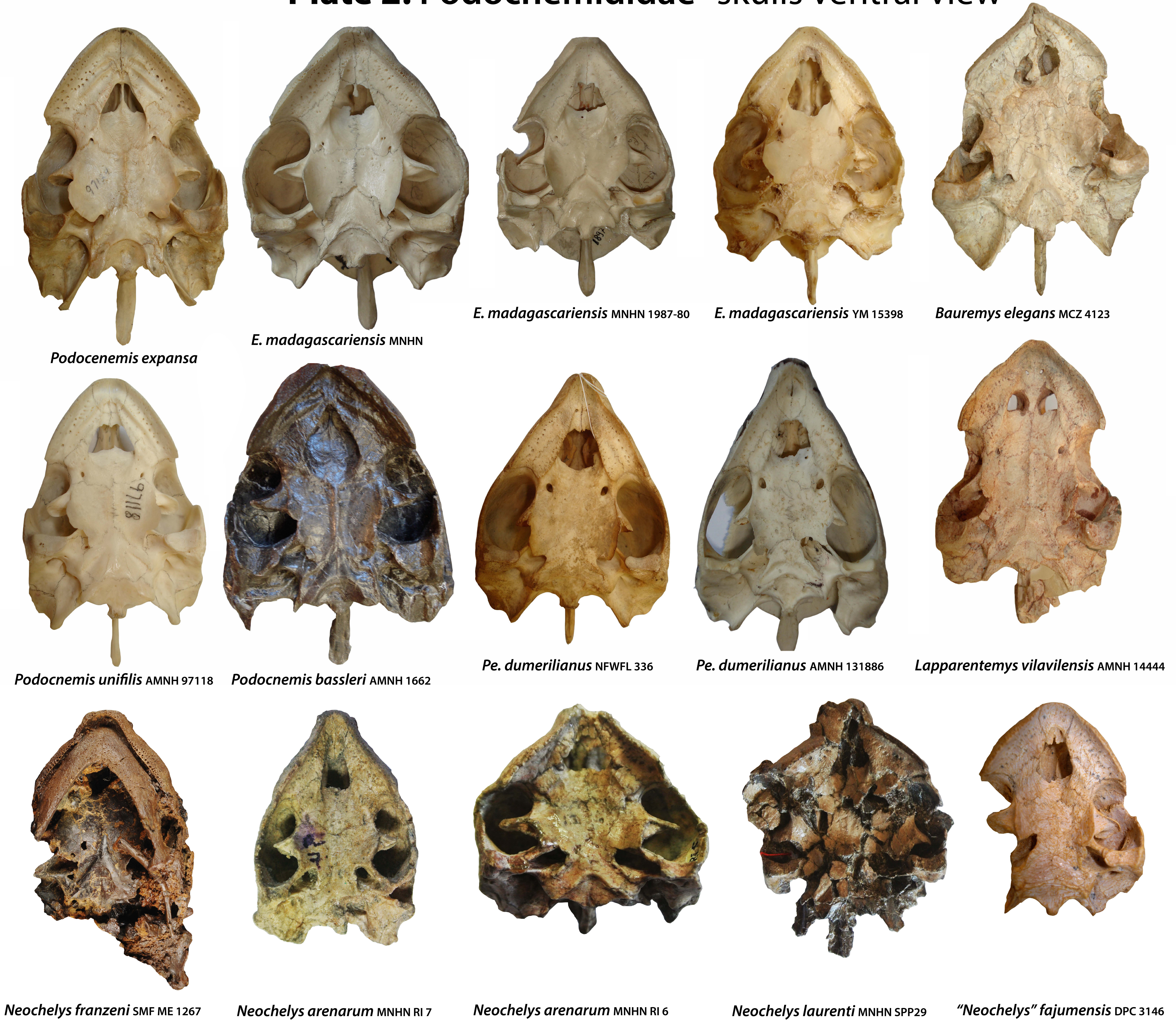

Plate 3. Podocnemididae- skulls left lateral view

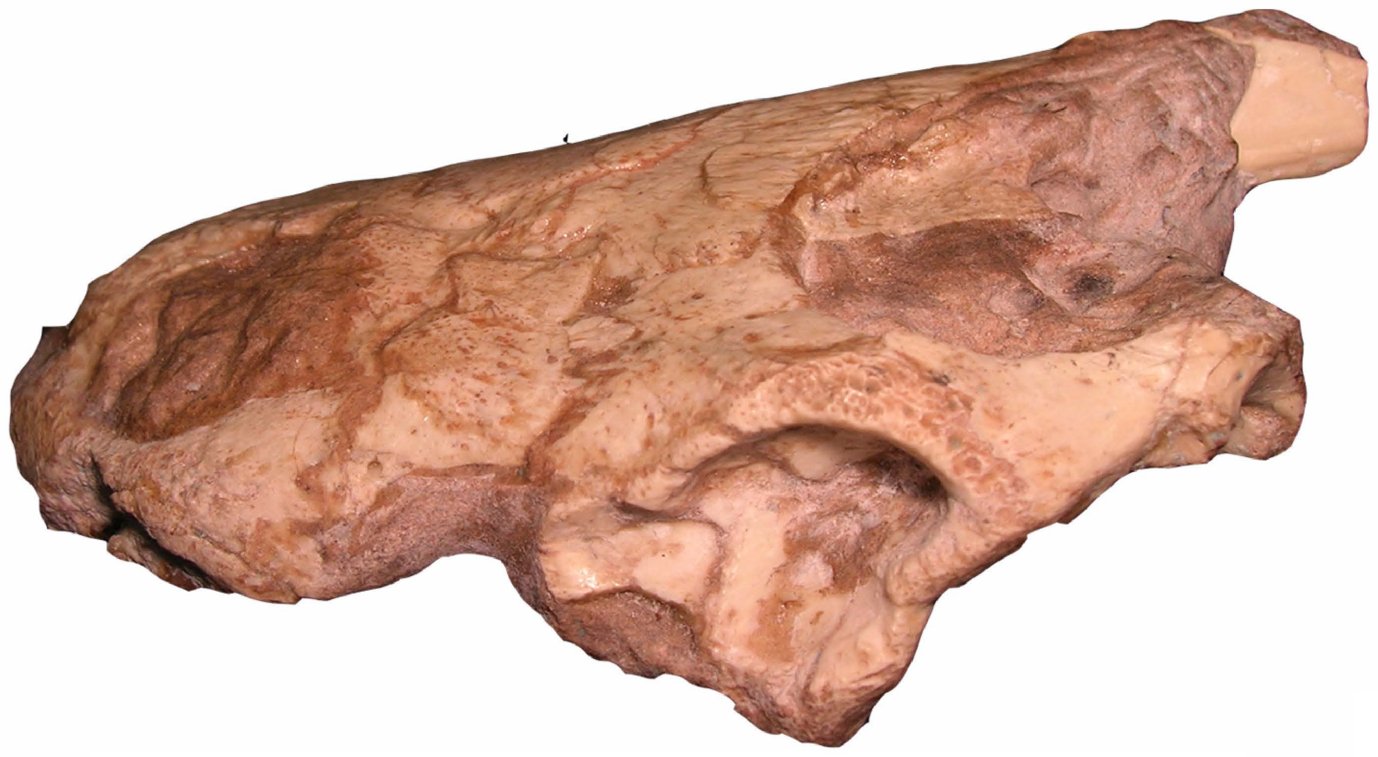
Bauremys elegans 4322

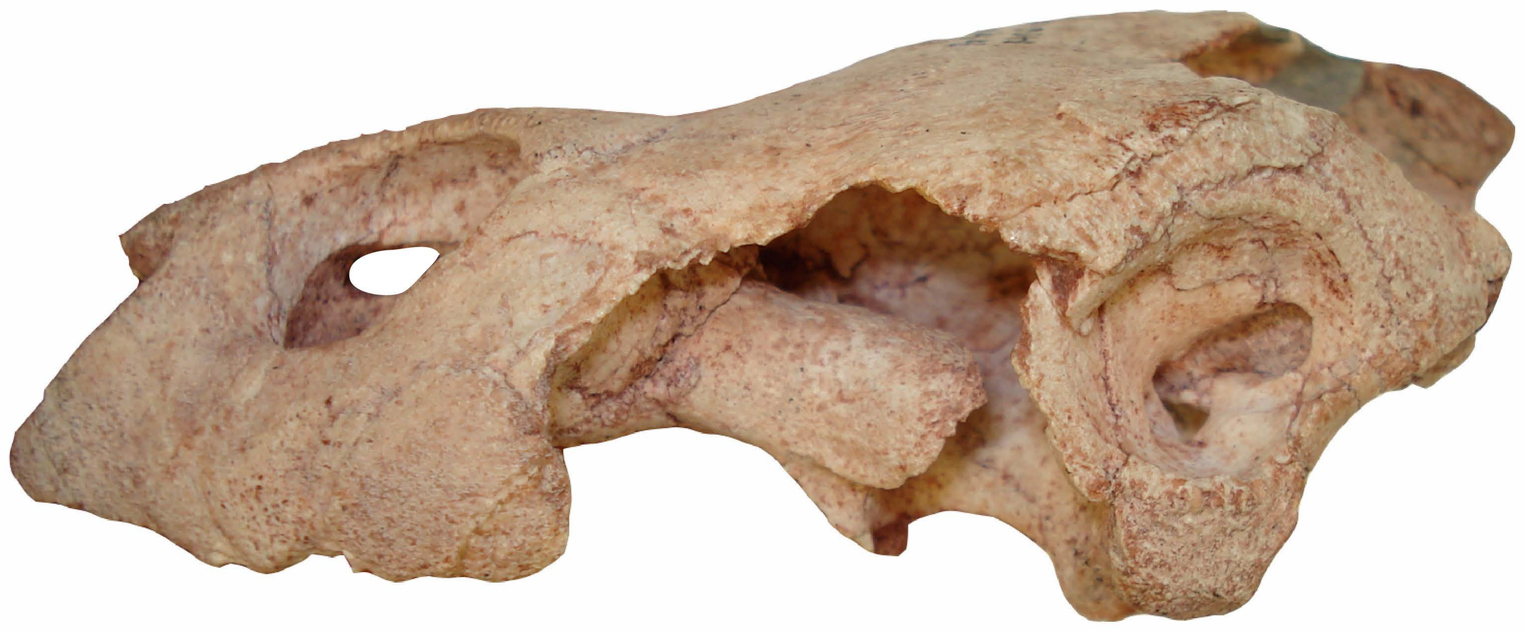
Lapparentemys vilavilensis AMNH 14444

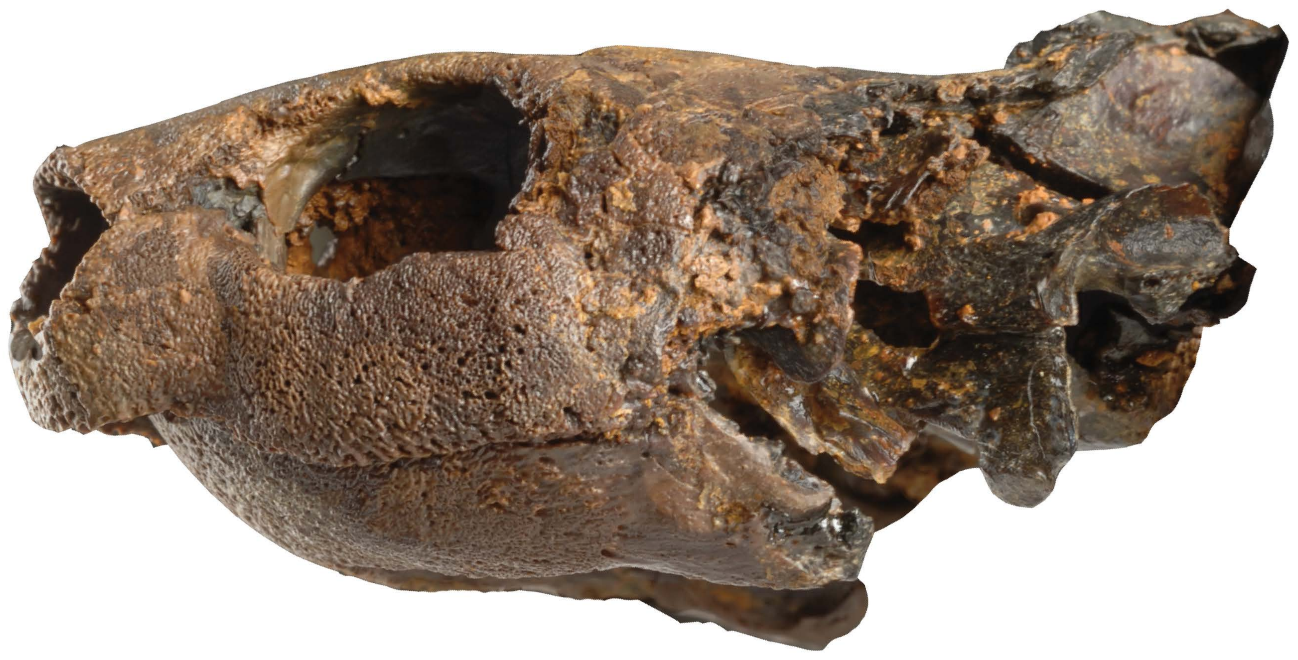
Neochelys franzeni SMF ME 1267

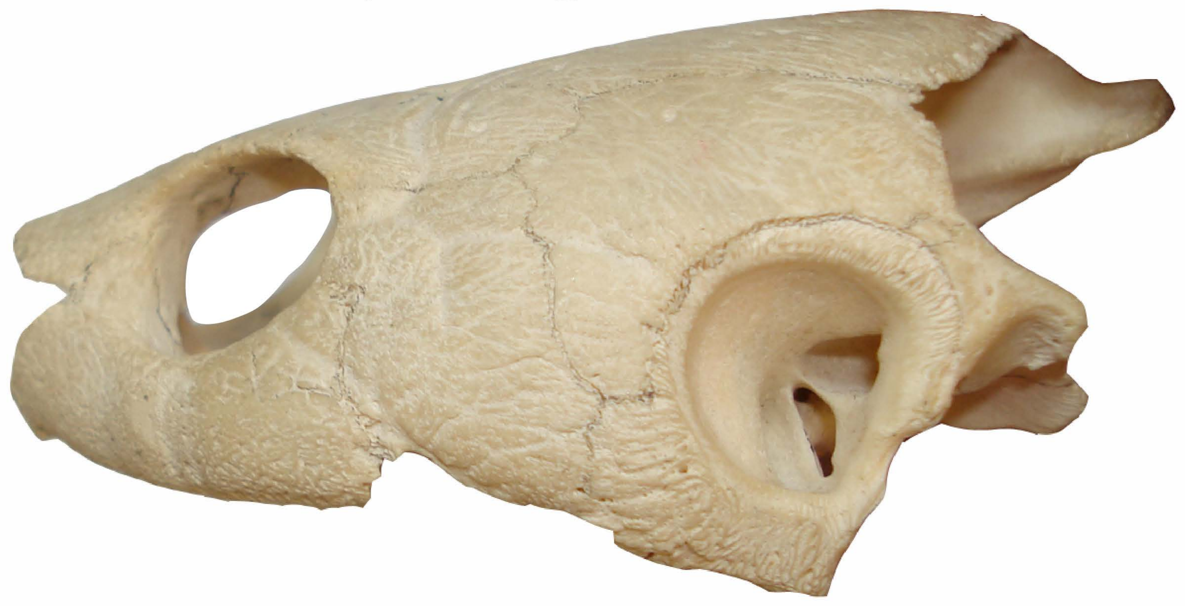
E. madagascariensis YM 15398

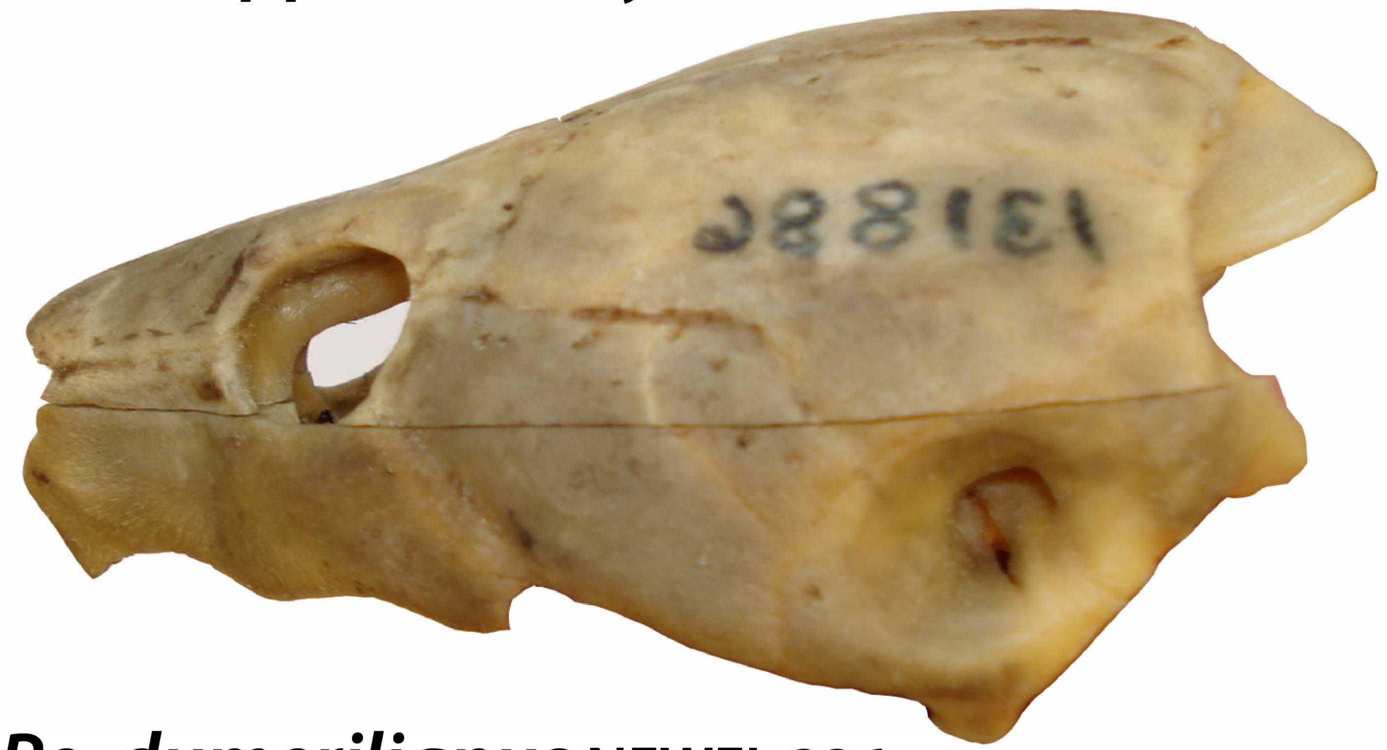

Pe. dumerilianus NFWFL 336

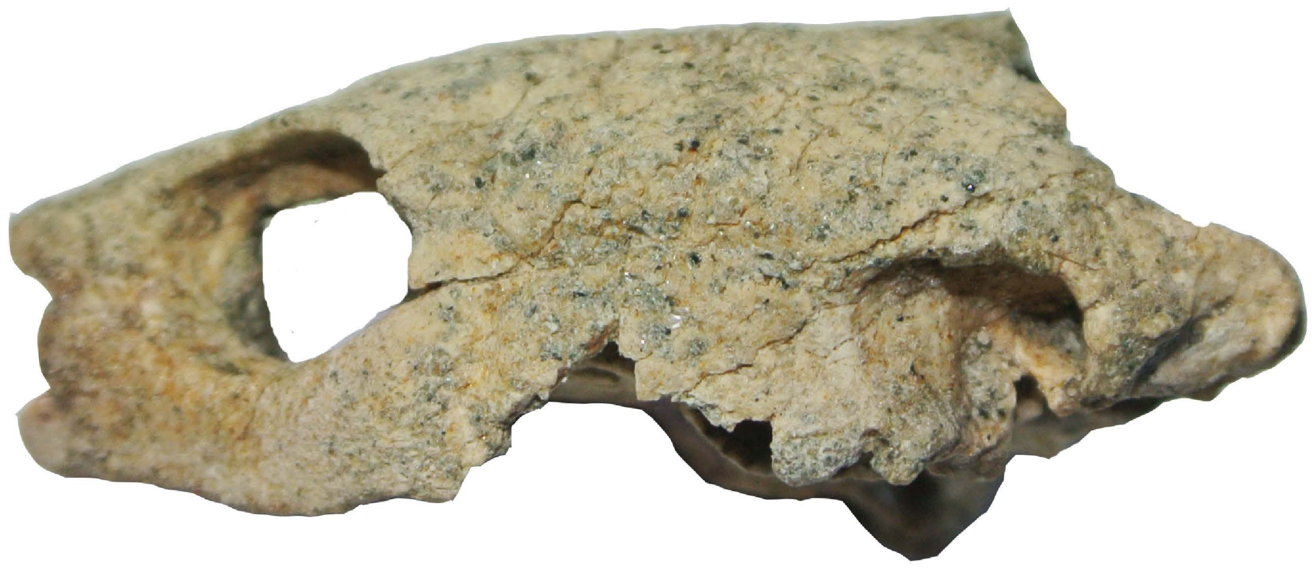
Neochelys arenarum MNHN RI 7

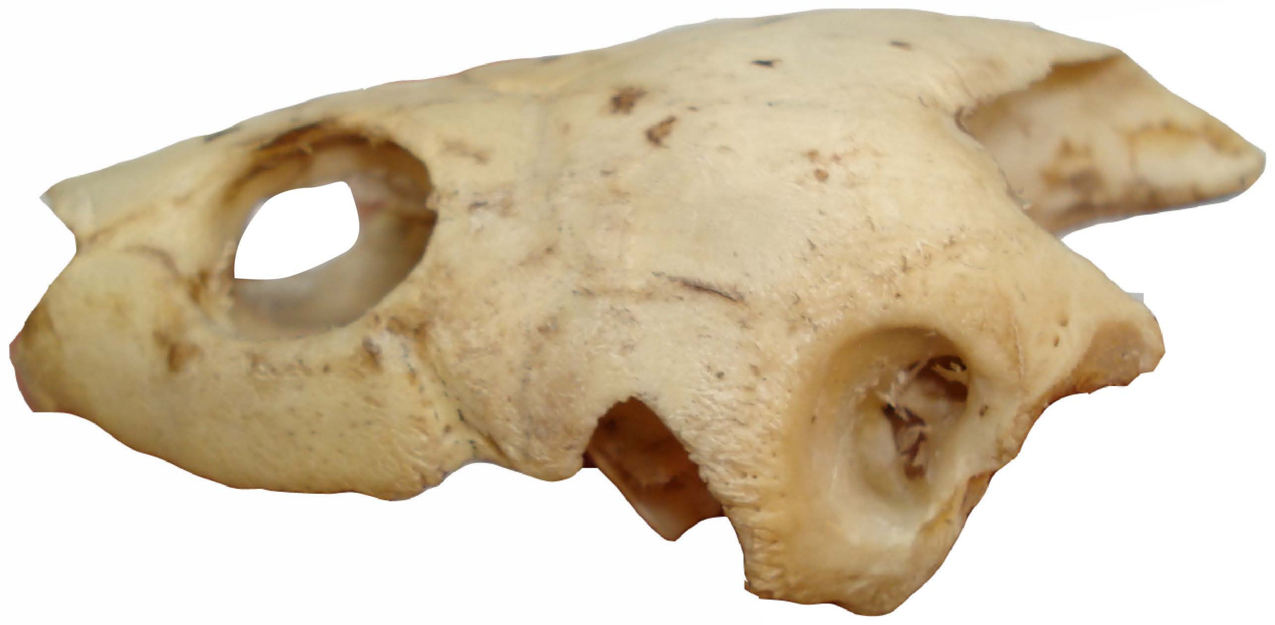
E. madagascariensis MNHN 1987-80

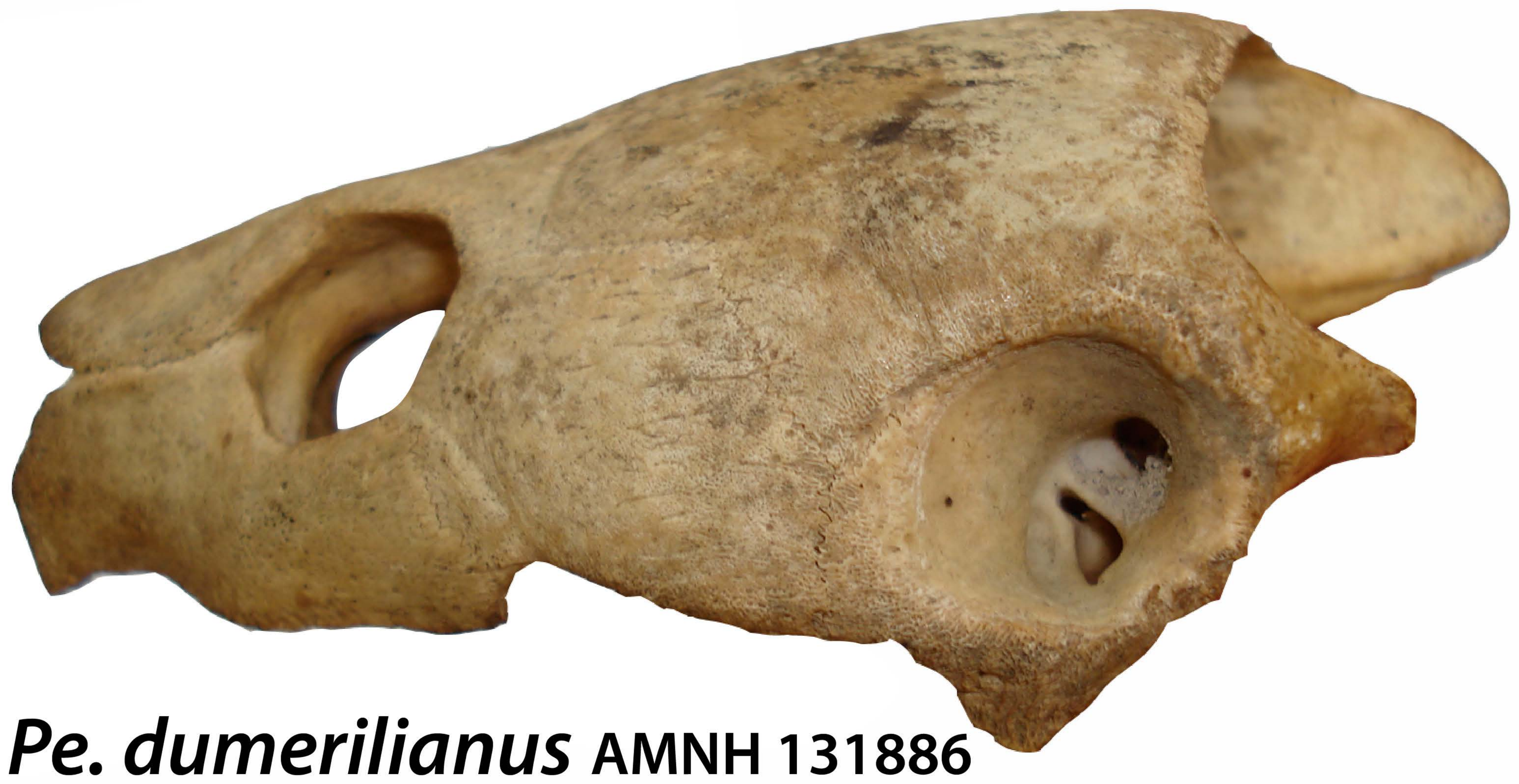
Pe. dumerilianus AMNH 131886

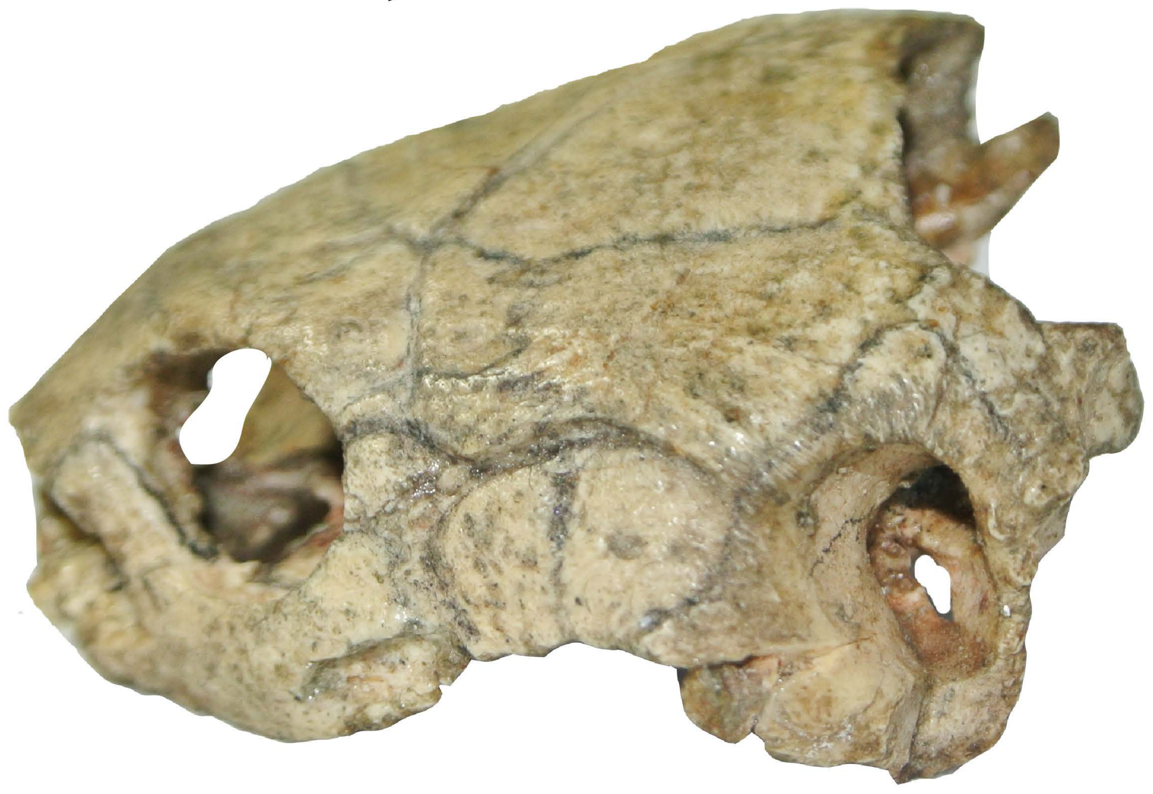
Neochelys arenarum MNHN RI 6

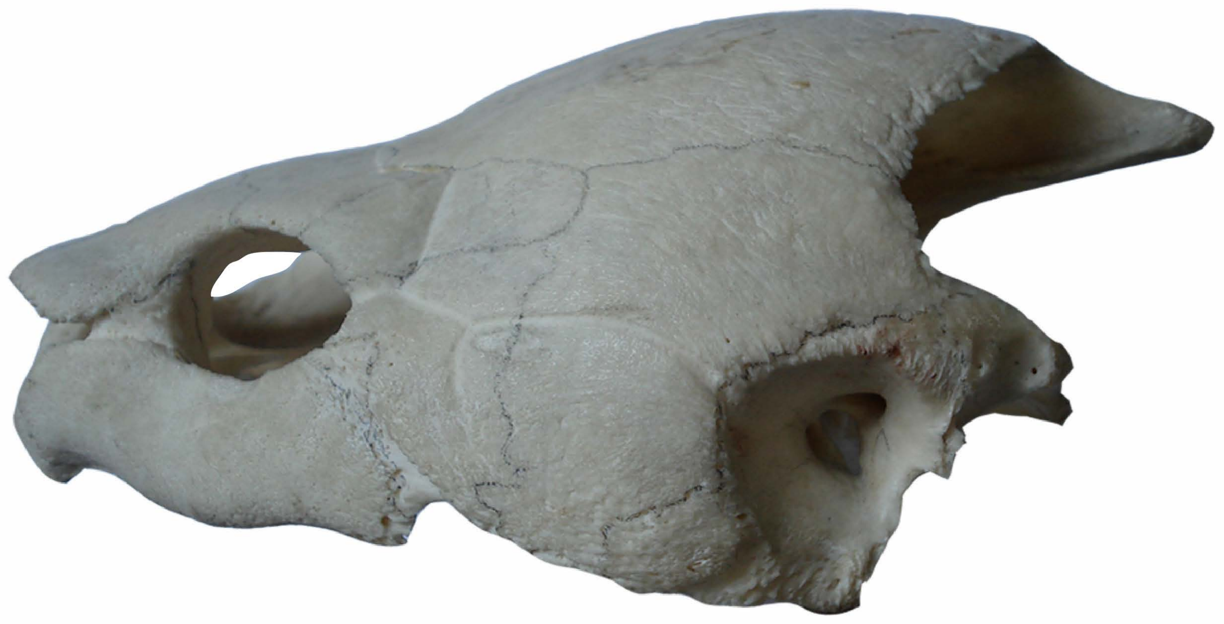
E. madagascariensis MNHN

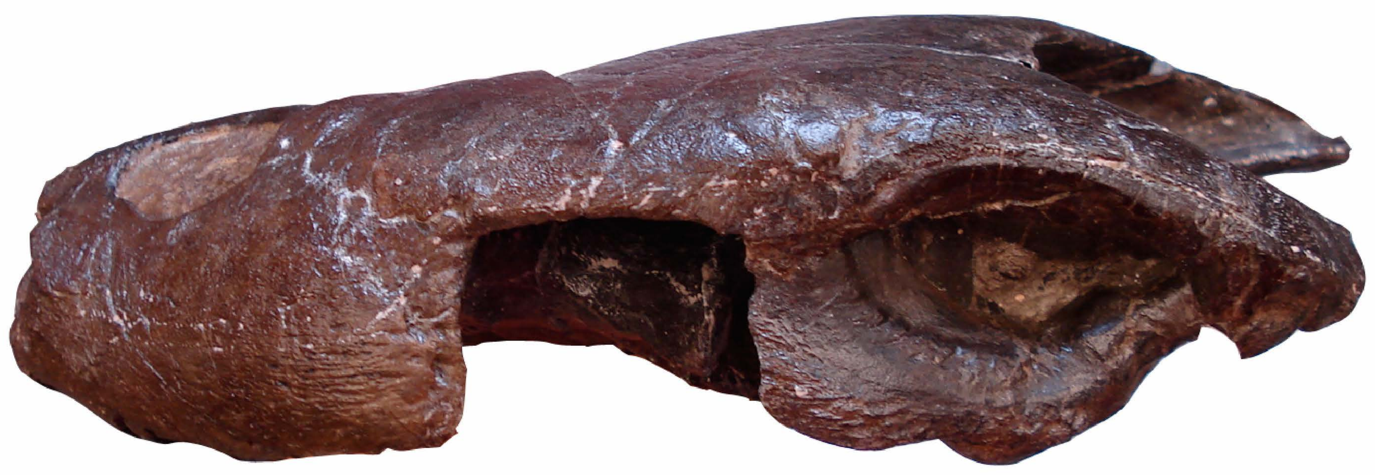
Podocnemis bassleri AMNH 1662

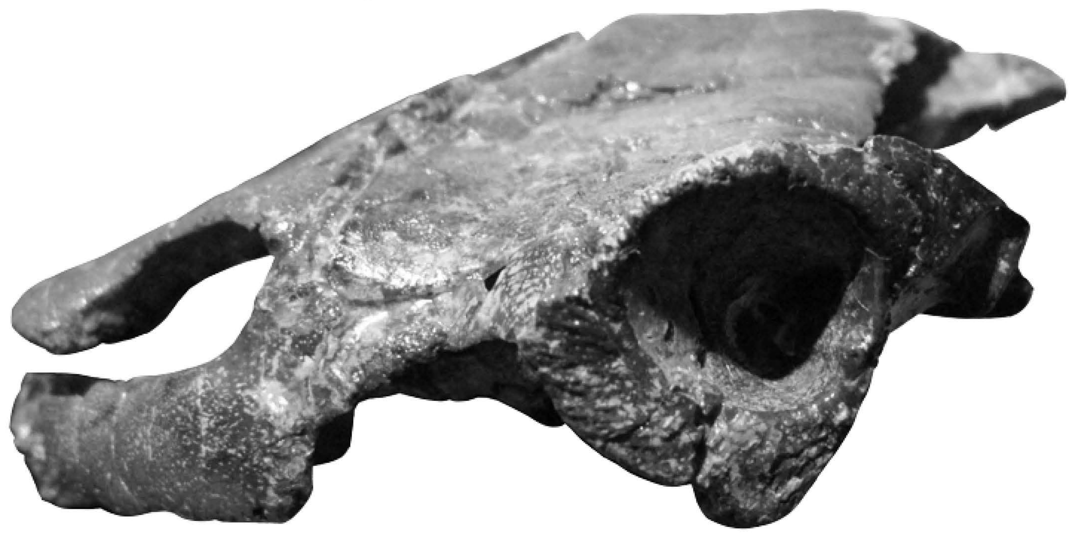
Neochelys laurenti MDE T12

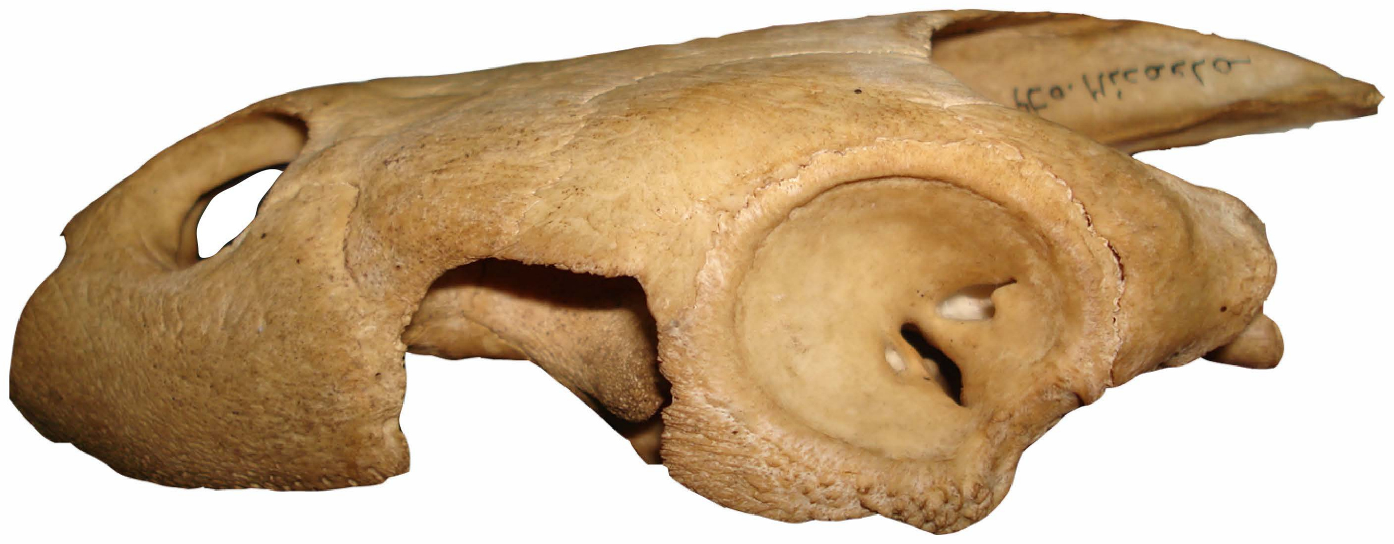
Podocenemis expansa ICN

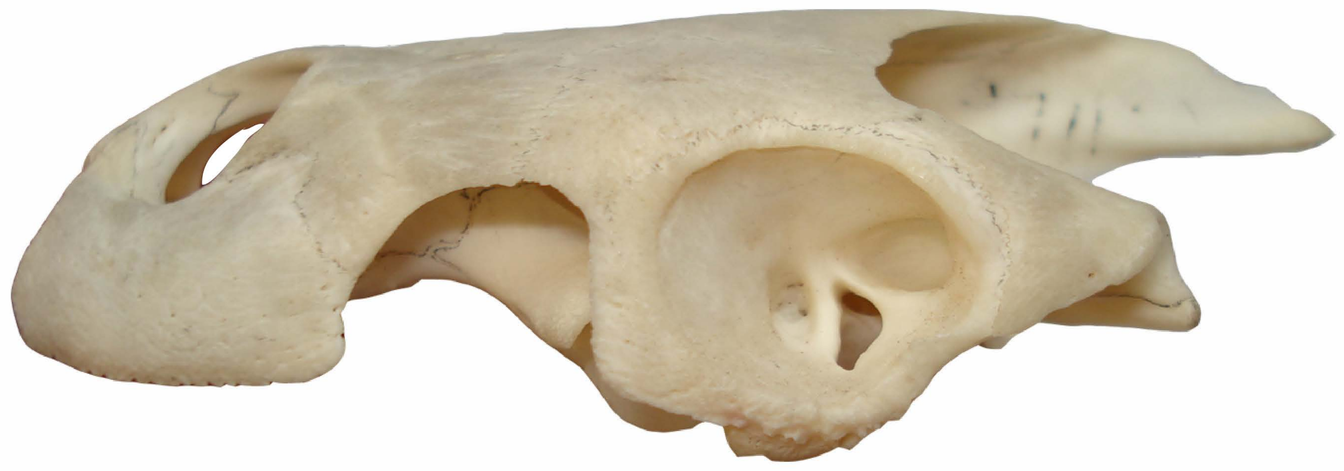
Podocnemis unifilis AMNH 97118

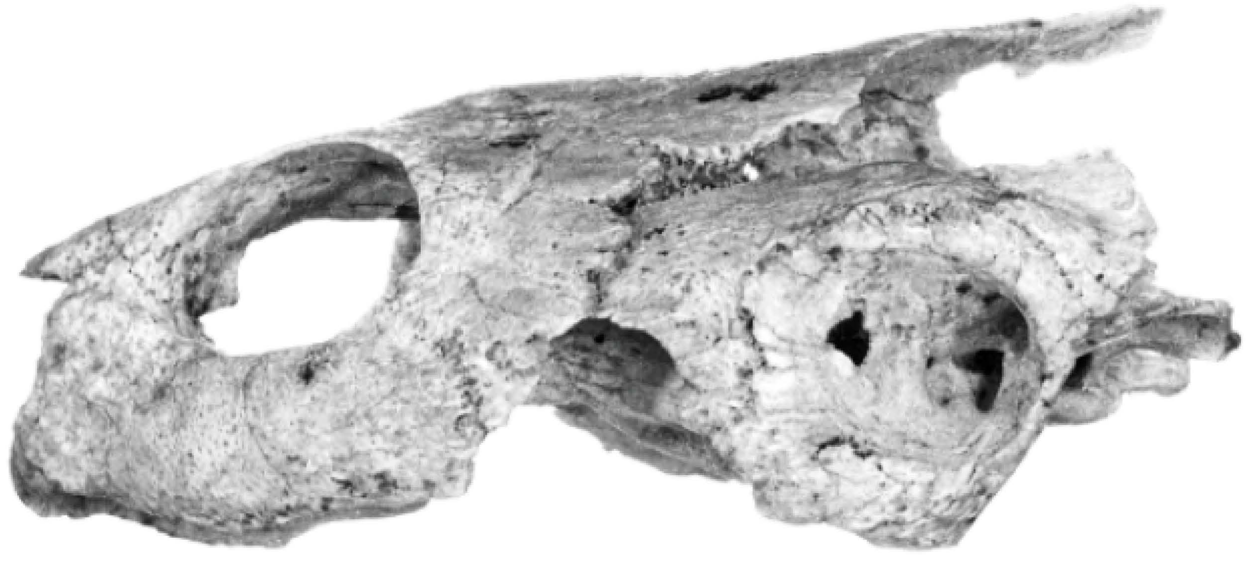
"Neochelys" fajumensis DPC 3146

Plate 4. Carapace outline

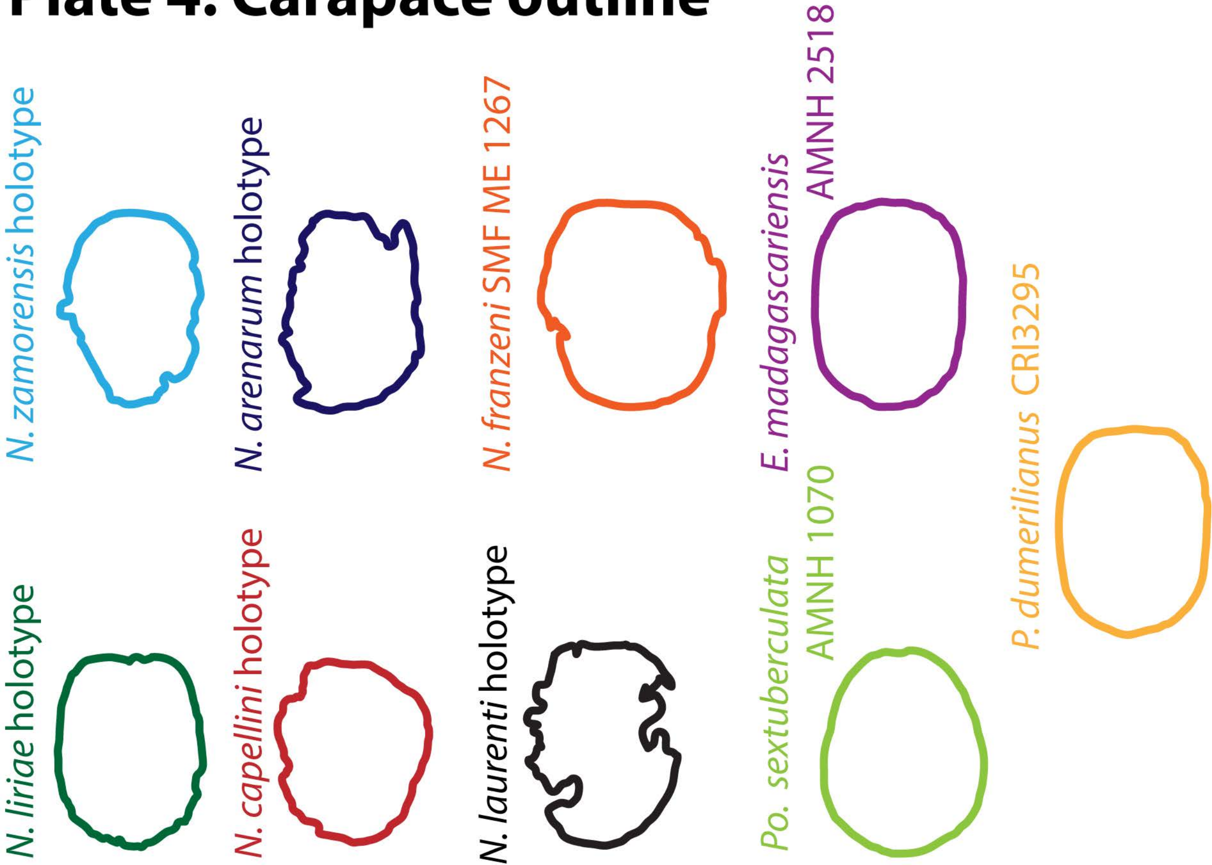

Plate 5. Vertebral scute 1 shape

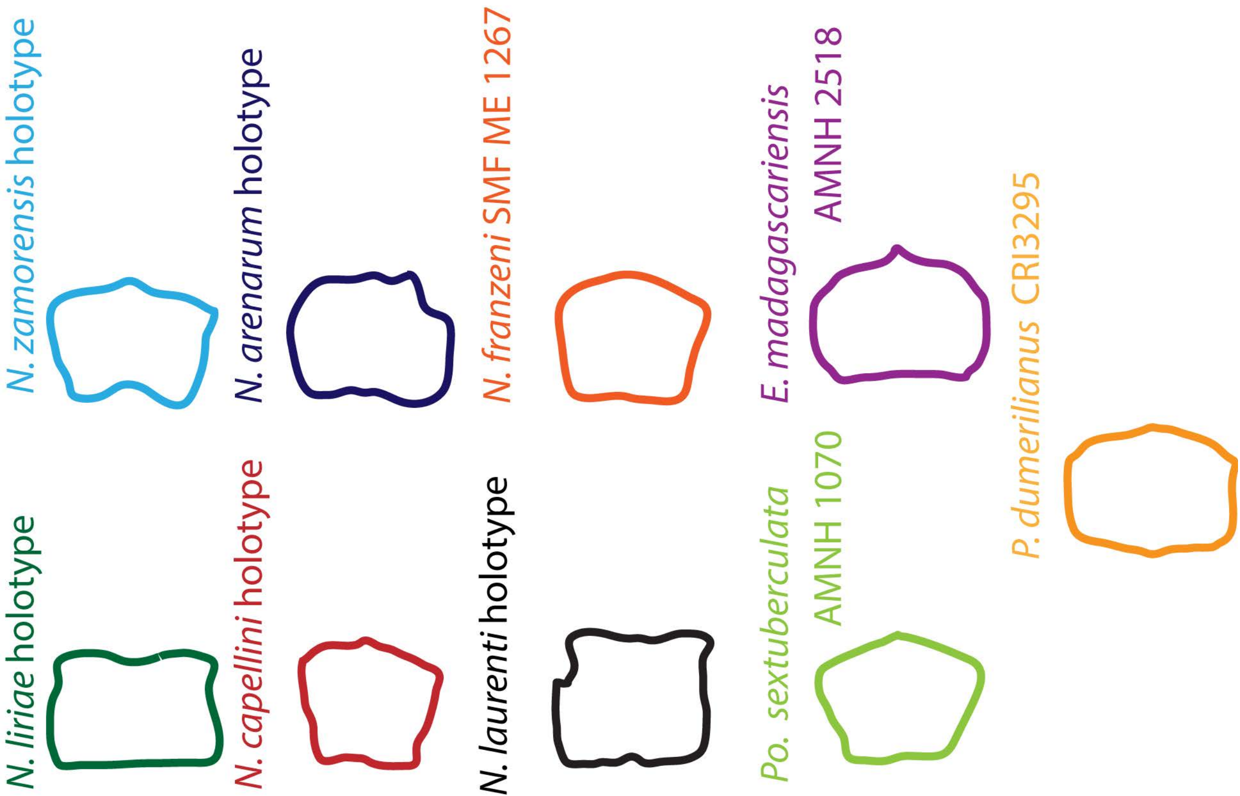

Plate 6. Anterior plastron (bones/scutes)

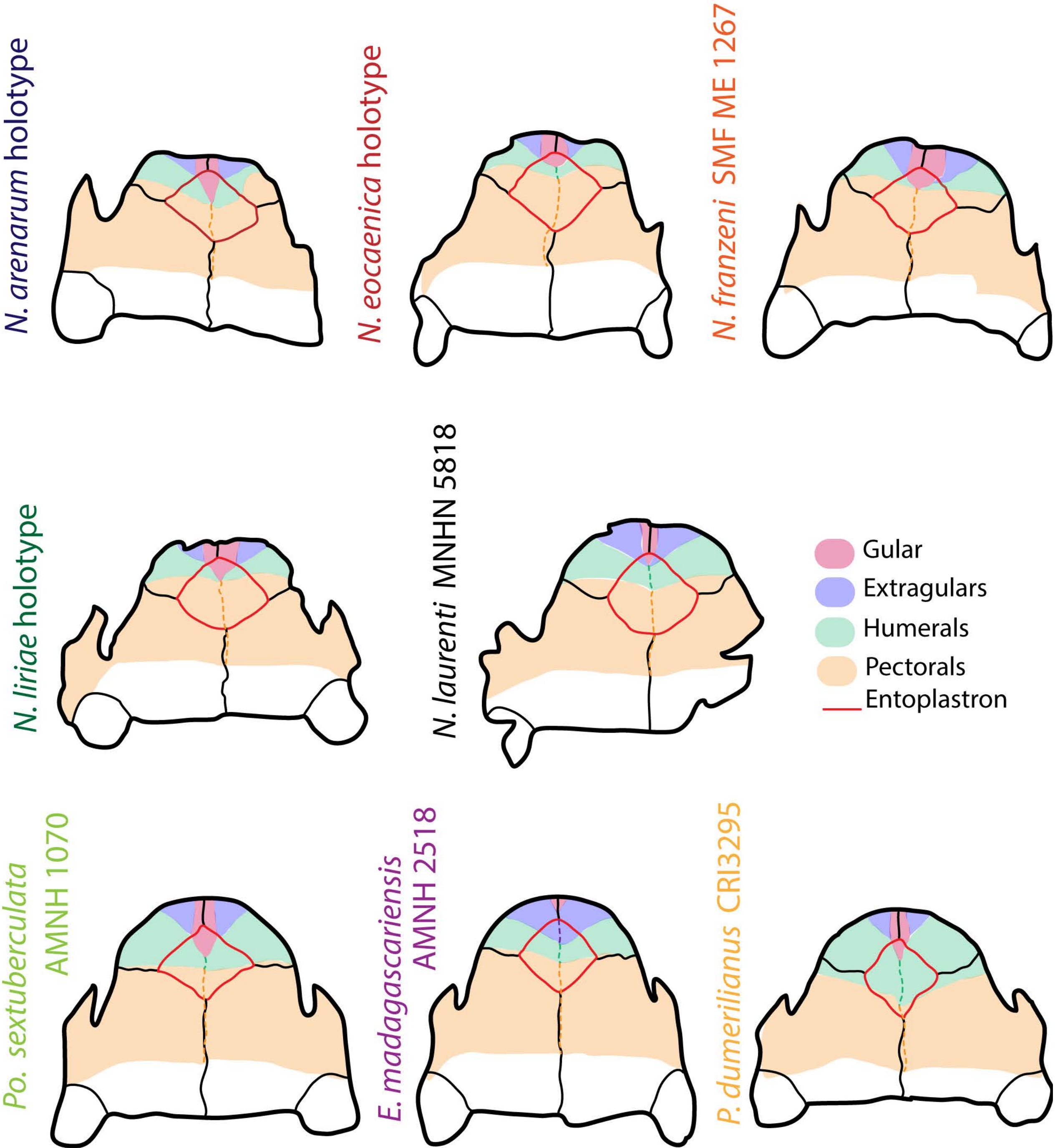

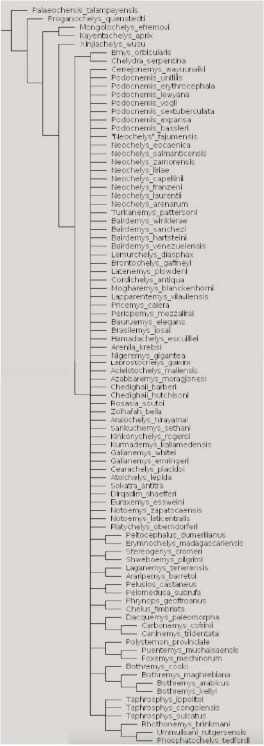

Plate 7. Strict consensus all taxa, all morphological characteres

List 1. Extant and some fossil specimens directly examined.

Institutional abbreviations:

AMNH	American Museum of Natural History, New York, USA
CRI	Chelonian Research Institute, Oviedo, Florida, USA
ICN	Instituto de Ciencias Naturales, Universidad Nacional de Colombia, Bogotá, Colombia
IGM	Museo José Royo y Gómez, Servicio Geológico Colombiano, Bogotá, Colombia
MNHN	Muséum National d'Histoire Naturelle, Paris, France
NFWFL	National Fish and Wildlife Foundation, Herpetological collection, University of Florida, Gainesville, USA
NMW	Naturhistoriches Museum Wien, Viena, Austria
UF	Herpetological Collection, University of Florida, Gainesville, Florida, USA
UCMP	University of California Museum of Paleontology, Berkeley, California, USA
USNM	Smithsonian National Museum of Natural History, Maryland, USA

**Species**	Institution/Collection	Collection number	
Neochelys arenarum	MNHN	RI6	Fossil
Neochelys arenarum	MNHN	RI7	Fossil
Neochelys arenarum	MNHN	RI4	Fossil
Neochelys arenarum	MNHN	RI45	Fossil
Neochelys laurenti	MNHN	FSPP29	Fossil
Neochelys laurenti	MNHN	FSPP28	Fossil
Neochelys laurenti	MNHN	5819	Fossil
Neochelys laurenti	MNHN	5818	Fossil
Neochelys laurenti	MNHN	FSPP30	Fossil
Neochelys liriae	MNHN	EBA515	Fossil
Podocnemis bassleri	AMNH	1662	Fossil
Podocnemis medemi	IGM	1863	Fossil
Podocnemis pritchardi	UCMP	63782	Fossil
Podocnemis expansa	AMNH	58098	
Podocnemis expansa	AMNH	62947	
Podocnemis expansa	AMNH	97124	
Podocnemis expansa	ICN	6319	
Podocnemis expansa	ICN	7341	
Podocnemis expansa	ICN	8921	
Podocnemis expansa	UF	118589	
Podocnemis expansa	USNM	29476	

Podocnemis expansa	USNM	65113
Podocnemis expansa	NMW	1824
Podocnemis expansa	NMW	137
Podocnemis expansa	NMW	1852
Podocnemis expansa	NMW	34528
Podocnemis expansa	NMW	35550
Podocnemis expansa	NMW	39252
Podocnemis erythrocephala	MNHN	1463
Podocnemis erythrocephala	ICN	7339
Podocnemis erythrocephala	UF	57921
Podocnemis erythrocephala	USNM	35474
Podocnemis erythrocephala	CRI	1194
Podocnemis erythrocephala	CRI	1922
Podocnemis erythrocephala	CRI	2757
Podocnemis erythrocephala	CRI	4068
Podocnemis erythrocephala	CRI	6023
Podocnemis erythrocephala	CRI	8207
Podocnemis erythrocephala	CRI	8208
Podocnemis sextuberculata	AMNH	111068
Podocnemis sextuberculata	AMNH	111069
Podocnemis sextuberculata	AMNH	111070
Podocnemis sextuberculata	USNM	65109
Podocnemis sextuberculata	USNM	65110
Podocnemis sextuberculata	CRI	2830
Podocnemis sextuberculata	CRI	5500
Podocnemis sextuberculata	CRI	6543
Podocnemis lewyana	ICN	6207
Podocnemis lewyana	ICN	6203
Podocnemis lewyana	ICN	7653
Podocnemis lewyana	ICN	7068
Podocnemis lewyana	ICN	5769
Podocnemis lewyana	ICN	1715
Podocnemis lewyana	ICN	1699
Podocnemis lewyana	ICN	1672
Podocnemis lewyana	MNHN	283
Podocnemis lewyana	MNHN	286
Podocnemis lewyana	CRI	3230
Podocnemis unifilis	NMW	1829
Podocnemis unifilis	AMNH	58195
Podocnemis unifilis	AMNH	97118
Podocnemis unifilis	ICN	1701
Podocnemis unifilis	ICN	1721
Podocnemis unifilis	ICN	6455

Podocnemis unifilis	USNM	313861
Podocnemis unifilis	CRI	2776
Podocnemis unifilis	CRI	2778
Podocnemis unifilis	CRI	4370
Podocnemis unifilis	CRI	11120
Podocnemis vogli	MNHN	5074
Podocnemis vogli	ICN	1682
Podocnemis vogli	UF	39060
Podocnemis vogli	UF	39100
Podocnemis vogli	USNM	266206
Podocnemis vogli	ICN	1686
Pelocephalus dumerilianus	CRI	7524
Pelocephalus dumerilianus	CRI	3344
Pelocephalus dumerilianus	CRI	3295
Pelocephalus dumerilianus	CRI	1348
Pelocephalus dumerilianus	CRI	1344
Pelocephalus dumerilianus	CRI	1343
Pelocephalus dumerilianus	USNM	257688
Pelocephalus dumerilianus	USNM	257687
Pelocephalus dumerilianus	NFWFL	339
Pelocephalus dumerilianus	NFWFL	337
Pelocephalus dumerilianus	NFWFL	336
Pelocephalus dumerilianus	ICN	7631
Pelocephalus dumerilianus	ICN	7324
Pelocephalus dumerilianus	AMNH	131886
Erymnochelys madagascariensis	AMNH	843120
Erymnochelys madagascariensis	AMNH	2518
Erymnochelys madagascariensis	MNHN	80
Erymnochelys madagascariensis	AMNH	116
Erymnochelys madagascariensis	AMNH	843124
Erymnochelys madagascariensis	AMNH	843125
Erymnochelys madagascariensis	NMW	139
Erymnochelys madagascariensis	NMW	140
Erymnochelys madagascariensis	NMW	141
Erymnochelys madagascariensis	NMW	142
Erymnochelys madagascariensis	NMW	146
Erymnochelys madagascariensis	NMW	147
Erymnochelys madagascariensis	NMW	422
Erymnochelys madagascariensis	NMW	1811
Erymnochelys madagascariensis	NMW	1812
Erymnochelys madagascariensis	NMW	1813
Erymnochelys madagascariensis	NMW	1814
Erymnochelys madagascariensis	NMW	1815

Erymnochelys madagascariensis	NMW	1816
Erymnochelys madagascariensis	NMW	1821
Erymnochelys madagascariensis	NMW	1822
Erymnochelys madagascariensis	NMW	1839
Erymnochelys madagascariensis	NMW	1840
