# Supplementary material for: A global phylogeny of Pelomedusoides turtles with new material of Neochelys franzeni Schleich, 1993 (Testudines, Podocnemididae) from the middle Eocene, Messel Pit, of Germany
Source: PeerJ. 2015 Aug 27;3:e1221. doi: 10.7717/peerj.1221 (PMC4556147; doi:10.7717/peerj.1221)
Supplement: Supplemental Information 2 — List of 187 morphological characters used in the phylogenetical analysis presented in this study. Also, characters exclude and changes in scores from previous studies, as well as composite plates with figures for each of the characters. Individual .eps files for each character can be download on Dryad (LINK). [file peerj-03-1221-s002.pdf]

Supplemental Information 2. An illustrated guide of morphological characters for turtles of the clade Pleurodira (Testudines: Pan-Pleurodira) turtles.

Introduction

The body plan of turtles can be considered as a unique masterpiece in vertebrate evolution, by its particular anatomy and physiology (see (Hirasawa et al. 2013; Hirasawa et al. 2014; Lyson et al. 2014) references therein). The turtle skeleton is formed by a cranium (skull, lower jaw, and hyoid apparatus) composed of approximately 46 bones and 282 axial skeletal bones (including limbs, neck, tail, pelvic and pectoral girdles, carapace and plastron)(Pritchard 2008). This complex anatomical configuration and all its possible variations in shape and number of bones are related not only to lineages diversity, but also to environmental, climatic, and geographical adaptations through out their almost 229 Ma of evolution; making the reconstruction of turtles evolutionary history and their phylogenetic relationships a challenging labor for biologists and paleontologists. Labor that can be even more tedious if different authors work with different morphological terminology and definitions to refer to the same structure or features. A good example of this was recently discussed by (Rabi et al. 2013), pointing out how through years, different authors have used different names to refer to the same carotid arteria foramina, creating not only confusion and misunderstanding, but also erroneous scoring in phylogenetic studies.

In order to set up a common and practical morphological terminology, not only for researchers in the field of turtle morphology, taxonomy, and phylogenetics, but also for students and future turtle researchers. I present here an illustrated guide of morphological characters with importance for phylogenetic studies of Pan-Pleurodira, principally Bothremydidae and Podocnemididae clades, based on the compilation, modification, and discussion of previous studies (see Session 1 below for references and abbreviations), as well as new insights from direct examination of a good number of specimens (See Supp. Inf. 3, list 1). Panpleurodires or side-necked turtles includes a highly diverse group of extant and extinct turtles, currently restricted to freshwater environments of the southern hemisphere (South America, Africa, Madagascar, and Australia), but globally spread during the Cretaceous and most of the Cenozoic (Cadena & Joyce 2015; Cadena et al. 2012a; Gaffney et al. 2011;2006).

Guide organization

This guide is organized in two main sessions. The first one is a list of 187 morphological characters, with their corresponding definitions, abbreviated reference(s) from where they were taken, and remarks if they were reworded, reorganized, combined or any other additional information or discussion. The second session corresponds to the graphical component of this guide, for which each character is figured in 36 composite plates, including all its possible character states, using a configured template in Adobe Illustrator CS6 (Fig. 1, session 2 of this appendix), see also (Supp. Inf. 4, for compressed file containing all .eps files for each character, facilitating modification of states or addition of new characters by future studies)

Session 1. Characters list, description, and discussions.

Characters described here have been taken, modified or added from previous global turtles or Pan-Pleurodira only studies or character lists (Bardet et al. 2013; Cadena 2010; Cadena et al. 2012a, 2012b; Cadena et al. 2013; Cadena and Parham, 2015; Gaffney et al. 2006, 2011; Hirayama 1994,1998; Joyce 2007; Joyce et al. 2011; Kear & Lee 2006; Lapparent de Broin 2000; Parham & Pyenson 2010; Pérez-García & Lapparent de Broin 2013; Sterli 2008; Sterli & de la Fuente 2013). Characters excluded from previous studies are found at the end of the session, principally characters that represent exclusive autapomorphies or represent redundant characters, reasons for exclusion are presented there too. See also Supp. Inf. 2 for the NEXUS file containing the character-taxon matrix for Pleurodira using the character list described below, as well as the changing in scoring presented at the end of this session.

Abbreviations for references used in the character list.

BR (Bardet et al., 2013)
CA1 (Cadena et al., 2010)
CA2 (Cadena et al., 2012a)
CA3 (Cadena et al., 2012b)
CA4 (Cadena et al., 2013)
GF1 (Gaffney et al., 2006)
GF2 (Gaffney et al., 2011)
HY1 (Hirayama, 1994)
HY2 (Hirayama, 1998)
JY1 (Joyce, 2007)
JY2 (Joyce et al., 2011)
KL (Kear and Lee, 2006)
LA (Lapparent de Broin, 2000)
PH (Parham and Pyenson, 2010)
PE (Pérez-García and Lapparent de Broin, 2013)
ST (Sterli, 2008)
STF (Sterli and de la Fuente 2013)

Skull

1. Nasals: 0 = present; 1 = absent. **GF1 (ch 1); JY1 & STF (ch 1, Nasal A). HY2, KL, BR (ch 2).**
2. Nasals, medial contact of nasals: 0 = nasals contact one another medially along their entire length; 1 = medial contact of nasals partially or fully hindered by long anterior frontal process. **JY1 & STF (ch 2).**
3. Prefrontals, medial contact of prefrontals on the dorsal skull surface: 0 = absent; 1 = present, absence of contact between the nasal or aperture narium externa and the frontal. **GF1 (ch 4); JY1 & STF (ch 4, Prefrontal A), HY2, KL, BR (ch 3).**
4. Prefrontals, preorbital skull broad: 0 = narrow, equal or less than 90°

approximately; 1 = very broad, major than 90º. **GF1 (ch 5).** *Remarks:* values of angles were added to the states.

5. Prefrontals, anterior overhang onto aperture narium externa: 0 = overhang partially to completely covering the aperture, formed by the nasals; 1 = formed by prefrontals, covering a small portion of the posterior region of the aperture, ending in acute medial tip; 2 = formed by prefrontals, completely covering the aperture, ending in a straight to convex edge. **CA2 (ch 5); GF1 (ch 6).** *Remarks:* character reworded.
6. Prefrontals, prefrontal-palatine contact: 0 = present; 1 = absent. **GF1 (ch 7); JY1 & STF (ch 6, Prefrontal C).** *Remarks:* this character is visible in dorsal or lateral views when orbital cavities are well preserved and free of rock matrix or without strong crushing or deformation effect.
7. Prefrontals, prefrontal-vomer contact: 0 = absent; 1 = present. **GF1 (ch 8); JY1 & STF (ch 5, Prefrontal B).** *Remarks:* this character is visible in sagittal cut of the skull, limiting the scoring in many fossils.
8. Prefrontals, prefrontal-parietal contact: 0 = absent; 1 = present. **GF1 (ch 10).**
9. Prefrontals, interorbital groove at medial contact between prefrontals: 0 = absent; 1 = present. **CA1 (ch 9); GF2 (ch 3);** and **LA (without number).**
10. Prefrontals, prefrontal-frontal anterodorsal shape in lateral view: 0 = flat to slightly convex; 1 = strongly convex. **GF2 (ch 4).**
11. Frontals-prefrontals, direction of the orbits in dorsal view of the skull: 0 = laterally facing, with a very narrow to almost complete absent dorsal exposure of the maxilla and jugal; 1 = dorsolateral facing, with portions of the maxilla and jugal dorsally exposed. **GF1 (ch 11).**
12. Frontals, foramen interorbitale: 0 = high, circular to oval in shape; 1 = low, much wider than height. **GF1 (ch 12).**
13. Parietal, parietal-quadratojugal contact: 0 = absent; 1 = short; 2 = long. **GF1 (ch 13).**
14. Parietal, temporal emargination in dorsal and lateral views: 0 = absent, complete roofing of the fossa temporalis, with straight to slightly concave posterior margin of parietals, squamosal, and supratemporal bones, keeping a very low posterior skull, compared to the orbital region, observed in lateral view; 1 = intermediate, partial roofing of the fossa temporalis, with concave to straight posterior margin of parietals and squamosal bones, covering partially the otic chamber, slightly higher posterior portion of the skull, compare to the orbital region; 2 = extremely reduced, almost complete roofing of the fossa temporalis, with slightly convex posterior margins of parietals and squamosals, and much higher posterior portion of the skull compare to the orbital region. **GF1 (ch 14); CA2 (ch 6).** *Remarks:* this character is redefined here considering (Lapparent de Broin 2000)(secondary roofing of the fossa temporalis) and (Gaffney et al. 2006) (temporal emargination); to do so I added to the states defined in (Cadena et al. 2012) the variations in height of the posterior portion of the skull and switch states 1 and 2 of (Gaffney et al. 2006)(ch 14).
15. Parietals, parietal-squamosal contact: 0 = present, upper temporal emargination absent or poorly developed; 1 = absent, upper temporal emargination well developed. **GF1 (ch 15); JY1 (ch 11) & STF (ch 12) (Parietal A); HY2, KL,**

BR (ch 7).

16. Parietals, parietal-pterygoid contact with the processus trochlearis pterygoidei: 0 = absent, restricted to the processus inferior parietalis; 1 = present, ventral process of the parietal reaches pterygoid on lateral side of sulcus palatinopterygoideus, very close to the base of processus trochlearis pterygoidei. **GF1 (ch 16).** *Remarks:* as pointed out by (Gaffney et al. 2006) this is a character very difficult to observe in fossils, depending of preservation, but CT images can help on its observation.
17. Parietals, sulcus palatinopterygoides: 0 = absent; 1 = present and high; 2 = present and low due to thick parietal and postorbital roof. **GF1 (ch 17).**
18. Parietals, enters orbital margin in dorsal view: 0 = no; 1 = yes. **GF1 (ch 18).**
19. Parietals, interparietal scute shape: 0 = absent; 1 = equilateral triangle; 2 = elongated triangle with straight to convex anterior margin; 3 = slightly elongated heart-like shape; 4 = parallel lateral sides, reaching the posterior margin of the parietals; 5 = broad posteriorly, reaching the posterior margin of the parietals; 6 = equilateral triangle elongated, with anterior margin posterior to the frontal-parietal sutural contact. **GF2 (ch 8).** *Remarks:* many fossil turtles do not preserved the sulci of scutes, see discussion about this character in (Lapparent de Broin et al. 2014) (suppl. Mat. 1 p. 16). In pleurodires, particularly in podocnemidids the sulci indicating the shape of the interparietal scute are usually well preserved. I added two extra states (state 3 and 6), which are particular shapes present in some podocnemidids. Testudines with very complex skull scutes pattern as for example *Chelus fimbriata* are code as not applicable (-) in the matrix.
20. Parietals, parietal scutes meeting medially: 0 = present; 1 = absent. **New character.**
21. Jugals, jugal retracted from orbital margin: 0 = jugals enters orbital margin; 1 = jugal slightly retracted; 2 = jugal widely retracted. **GF1 (ch 20).**
22. Jugals, jugal narrow dorsoventrally: 0 = absent, large exposure on the cheek; 1 = present, exposure in the cheek is long horizontally and short vertically. **GF1 (ch 21).**
23. Jugals, jugal-quadrate contact: 0 = absent; 1 = present. **GF1 (ch 22); HY2, KL & BR (ch 9).**
24. Jugals, exposure on triturating surface in ventral view of the skull: 0 = absent; 1 = large. **GF1 (ch 23).**
25. Jugals, cheek emargination: 0 = almost absent, forming a straight line dominated by the ventral edge of jugal; 1 = very advanced, exposing completely the fossa temporalis; 2 = moderate, reaching almost the level of the most dorsal margin of the orbit; 3 = slightly moderate, reaching the half level of the orbit, with an advance ventral descending contribution of jugal and quadratojugal; 4 = very shallow, complete ventral descending of jugal, resulting in a contact between this bone and the quadrate; 5 = very shallow, formed by a posterior projection of the maxilla, resulting in a contact between this bone and the maxilla, occasionally separated by a very narrow fissure. Also the jugal has a contact with the quadrate, but it is exclude it from the most ventral margin of the cheek emargination. **CA2 (ch 18); GF2 (ch 11); GF1 (ch 39); JY1 (ch 15) & STF (ch 21) (Jugal B).** *Remarks:* character combined and redefined from (Cadena et al. 2012) and (Gaffney et al. 2011) state characters have been reordered. Character 39 (maxilla-

quadrate contact) of (Gaffney et al. 2006) is clearly better defined as a state of the cheek emargination character. The condition of a narrow fissure separating the maxilla from the quadrate (State 2, GF1 (ch 39), considered by (Gaffney et al. 2006) as unique for *Phosphatochelys* and *Ummulisani* it is actually variable in *Phosphatochelys* as the same authors show in (fig 199 E), a specimen lacking the fissure and having a contact between the maxilla and quadrate. I include state 2 of GF1 (ch 39) in the definition of state 5 here.

26. Jugals, jugal-parietal contact: 0 = absent; 1 = present. **CA2 (ch 10); GF2 (ch 10).**
27. Squamosals, posterior projection: 0 = absent; 1 = present. **GF1 (ch 24); STF (ch 28, Squamosal D)**.
28. Squamosals, posteroventral vertical flange: 0 = absent; 1 = present. **GF1 (ch 25).**
29. Squamosals, lateral tubercle: 0 = absent; 1 = present. **GF1 (ch 26), GF2 (ch 13).**
30. Postorbitals, fossa orbitalis posterior enlargement: 0 = absent, orbital ring almost same size as the medial margin of the fossa orbitalis 1 = present, much larger than the orbital ring. **GF1 (ch 27).**
31. Postorbitals, septum orbitotemporale: 0 = absent; 1 = present, thick septum; 2 = present, but laterally thin creating a partially open septum. **GF1 (ch 28).**
32. Postorbitals, length versus width: 0 = slightly longer that wide; 1 = three times or more longer than wide; 2 = small, almost equal length vs. width. **GF1 (ch 29).** *Remarks:* (Gaffney et al. 2006) defined this character in terms of size, however the states were defined only respect to the length of the postorbital, then in (Gaffney et al. 2011) the states were defined in terms of size of the postorbital versus size of the orbit, here I precise better the states defining them comparatively with the width too.
33. Premaxillae, protrude anteriorly beyond labial ridge: 0 = no, or slightly forming a vertical to slightly incline wall; 1 = yes, projects anteriorly, specially visible in ventral view. **GF1 (ch 30).**
34. Premaxillae, midline depression: 0 = absent, shallow, or indistinct; 1 = distinct and wide, open U-like shaped; 2 = distinct but narrow, V-like shaped. **GF1 (ch 31).**
35. Premaxillae, dorsal sulcus: 0 = absent, smooth dorsal surface of the premaxilla; 1 = present, sulcus parallel to margin of the aperture narium externa. **GF1 (ch 33).**
36. Premaxillae, premaxilla-apertura narium interna contact: 0 = absent, usually because of a presence of vomer or a medial contact between maxillae in ventral view; 1 = present. **GF2 (ch 14); JY1 (ch 25) & STF (ch 35) (Premaxilla D).**
37. Maxillae, triturating surface: 0 = relatively narrow, parallel sides; 1 = triangular, wider posteriorly than anteriorly; 2 = triangular, very wide posteriorly. **GF1 (ch 34).**
38. Maxillae, triturating surface pits: 0 = absent; 1 = present. **GF1 (ch 35).**
39. Maxillae, accessory ridge on triturating surface: 0 = absent; 1 = present, one ridge; 2 = present, two ridges. **GF1 (ch 36); GF2 (ch 20); STF (ch 41, Maxilla E).** *Remarks:* combined character from GF1 (ch 36) presence or absence of the ridge and GF2 (ch 20) number of ridges presence.
40. Maxillae, maxilla-quadratojugal contact: 0 = absent, quadratojugal present; 1 = present; 2 = absent, quadratojugal absent. **GF1 (ch 38); JY1 (ch 17) & STF (ch 23) (Quadratojugal B).**

41. Maxillae, orbitonarial bar width: 0 = roughly equal to or slightly less than diameter of orbit; 1 = wider than the orbit; 2 = more than twice orbital diameter; 3 = very narrow, much less than diameter of orbit. **GF1 (ch 40).**

42. Maxillae, ventral rim of the orbit: 0 = very distinct margin; 1 = rim absent, gradually continuous slope. **GF1 (ch 42).**

43. Maxillae-premaxillae, medial expansion of triturating surface: 0 = absent, wide concavity at the midline; 1 = present, forming the medial maxillary ridge; 2 = secondary palate with midline cleft. **GF2 (ch 16).**

44. Maxillae-palatines, secondary palate length: 0 = secondary palate absent; 1 = relatively short (palate length/skull length less than 0.6), posterior edge of second palate does not reach the basisphenoid level; 2 = long (palate length/skull length more than 0.7), second palate reaches the basisphenoid level. **GF2 (ch 17).** *Remarks:* I added an additional state for the complete absence of the secondary palate, and also added to the definition of states 1 and 2 the ending level of the secondary palate versus the basisphenoid.

45. Maxillae, labial ridge height and width: 0 = high and narrow; 1 = low and thick. **GF2 (ch 19).**

46. Vomer, vomer bone: 0 = present, paired; 1 = present; 2 = absent. **GF1 (ch 45); JY1 (ch 26) & STF (ch 42) (Vomer).**

47. Vomer, central bar: 0 = sutured at both ends; 1 = very thin, attached anteriorly only or with a just touching posterior contact**. GF1 (ch 47).** *Remarks:* state 1 was modified to include the occasional occurrence of the vomer in *Podocnemis vogli*, which exhibits similar condition as in *Azabbaremys moragjonesi.*

48. Palatines, foramen palatinum posterius: 0 = in floor of the orbit; 1 = behind the orbit, in floor of sulcus palatinopterygoideus. **GF1 (ch 48).**

49. Palatines, dorsally arched palate: 0 = absent, palate low to almost flat; 1 = present, extremely arched. **GF1 (ch 49).**

50. Palatines, palatine contribution to upper triturating surface: 0 = absent or narrow generally forming a very small triangle; 1 = moderate, large triangular shape; 2 = large, palatine contributes with more than 50% of the width of the posterolateral edge of the triturating surface and it is long anteriorly projected. **GF1 (ch 50) GF2 (ch 24).**

51. Palatines, medial edges of palatal cleft: 0 = cleft absence; 1 = medial edges curved; 2 = medial edges parallel. **GF2 (ch 23).** *Remarks*: I switched states 1 and 2, because what this character is showing is the medial advance of second palate.

52. Palatines, dorsal process of palatine contacts parietal in septum orbitotemporale: 0 = no, 1 = yes. **GF2 (ch 25).** *Remarks*: very well preserved skulls or broken at the lateral temporal region are required to observe this character

53. Palatines, dorsal process of palatine contacts frontal in septum orbitotemporale: 0 = no; 1 = yes. **GF2 (ch 26).** *Remarks*: character visible in anterior view, looking inside the orbits.

54. Palatines, palatine-basisphenoid contact: 0 = no; 1 = yes, completely separating pterygoids. **GF2 (ch 28).**

55. Quadrates, antrum postoticum: 0 = absent, open incisura columellae auris; 1 = present, small; 2 = absent, closed incisura columellae auris; 3 = present, moderate to large. **GF1 (ch 51); Quadrate E), STF (ch 53, Antrum postoticum A).**

56. Quadrates, cavum tympani, arrangement between the quadrate, squamosal, opisthotic, stapes and Eustachian tube: 0 = no posterior bone restrictions, cavum tympani absent; 1 = cavum tympani well defined but open posteriorly, incisura columella auris posteroventrally open without bone restrictions; 2 = cavum tympani slightly open posteriorly, Eustachian tube and stapes separated by bone or a very narrow fissure; 3 = cavum tympani with just a very narrow posterior aperture, Eustachian tube and stapes enclosed by bone, incisura columella auris circular to slightly oval in shape posteriorly directed (seen in lateral view); 4 = cavum tympani completely closed with posterior acute tip, Eustachian tube and stapes enclosed by bone, incisura columella auris oval elongated in shape and ventrally directed (seen in lateral view). **CA2 (ch 15); GF1 (ch 52 & ch 53); JY2 (ch 37) & STF (ch 53) (Quadrate F).** *Remarks:* an additional state for meiolaniforms, where the Eustachian tube and stapes are enclosed by bone, with the contribution of the squamosal defined by (Sterli & de la Fuente 2013) is excluded here for being uninformative for the scope of Pan-Pleurodiran evolution.

57. Quadrates, ventral process of the sulcus Eustachii: 0 = absent; 1 = present. **GF1 (ch 54).** *Remarks:* this character is visible in lateral and even better in posterolateral view of the skull.

58. Quadrates, groove on incisura columella auris ridge: 0 = absent; 1 = present. **GF1 (ch 55).**

59. Quadrates, fossa precolumellaris: 0 = very small to absent; 1 = present, but very shallow; 2 = present, deep and well defined. **GF1 (ch 56).**

60. Quadrates, medial process contacting braincase elements and underlying cranioquadrate space: 0 = absent; 1 = present. **GF1 (ch 58).**

61. Quadrates, quadrate-basioccipital contact: 0 = absent; 1 = present. **GF1 (ch 59).**

62. Quadrates, condylus mandibularis position: 0 = near or in line with the basioccipital-basisphenoid suture; 1 = distinctly anterior to plane of basioccipital-basisphenoid suture; 2 = distinctly posterior to condylus occipitalis. **GF1 (ch 60).**

63. Quadrates, condylus mandibularis shape: 0 = much wider than long, with anterior and posterior edges straight to concave making it shorter at midline; 1 = slightly wider than long, in a "kidney bean" shape, with anterior edge straight to concave and posterior edge convex. **CA1 (ch 31).**

64. Quadrates, ventral process: 0 =absent to very short; 1 = moderate, condylus separate from the cavum tympani ventral margin level; 2 = long, condylus mandibularis considerably separate from the cavum timpani region. **CA1 (ch 18).**

65. Pterygoids, cavum pterygoidei: 0 = absent; 1 = present, shallow to moderately deep; 2 = very deep, delimited posteriorly by a well defined ridge of the medial process of the quadrate. **CA2 (ch 26); GF1 (ch 68 & ch 69); GF2 (ch 35); STF (ch 69, Pterygoid K)**. *Remarks:* (Gaffney et al. 2006) considered two separate characters for the concavity formed by the pterygoid, quadrate, basisphenoid and prootic, which is associated with the pterygoideus muscle and some of the main arteries entering the braincase, including the carotid. The two characters are: fossa pterygoidea (ch 68) and cavum pterygoidei (ch 69) pointing out that these two concavities are not homologous as was considered previously by (Lapparent de Broin 2000) (podocnemidoid fossa) and that were independently acquired by bothremydids (fossa pterygoidea) and podocnemidids plus *Brasilemys,* and

Hamadachelys (cavum pterygoidei). In (Gaffney et al. 2011) the difference between the fossa pterygoidea and cavum pterygoidei is expressed with an additional character (character 36), which deals with the anterior opening of this concavity (absent for the fossa, and present with several states for the cavum). Considering that the purpose of this study is to define a general character list for all Pan-Pleurodirans, these are my considerations about the fossa pterygoidea versus cavum pterygoidei. First of at all the definition of both characters is literally ambiguous in (Gaffney et al. 2006) because the words cavum and fossa mean in Latin exactly the same "hole" or "cavity". Secondly, the bones involved, position in the skull, as well as the function of the concavity are identical for both bothremydids and podocnemidoids, making the split of the character not well supported anatomically speaking, and finally variations in the roofing of the cavity, and size of the anterior opening are related to the degree of evolution of the character and can be expressed in a second character as I define it here.

66. Pterygoids, cavum pterygoidei flooring and anterior opening: 0 = uncovered by the pterygoid or basisphenoid, anteriorly closed by the pterygoid bone; 1 = slightly covered anteromedially by the basisphenoid and pterygoid, anterior opening small; 2 = partially to fully covered by the pterygoid flange, anterior opening moderate: 3 = partially to fully covered by the pterygoid flange, anterior opening large, foramen cavernosum opens in roof of cavum pterygoidei. **GF2 (ch 35 and ch 36).** *Remarks:* this character is a combination of character 35 and 36 from (Gaffney et al. 2011).
67. Pterygoids, pterygoid flange: 0 = absent; 1 = partially developed; 2 = well developed, covering the cavum pterygoidei and projected ventrally. **CA1 (ch 28); GF2 (ch 37).**
68. Pterygoids, processus trochlearis pterygoidei: 0 = absent; 1 = present. **GF1 (ch 70); GF2 (ch 38); JY1 (ch 42) & STF (ch 63) (Pterygoid E).**
69. Pterygoids, processus trochlearis pterygoidei lateral margin: 0 = very inclined compared with the horizontal axis of the processus; 1 = low inclination compared with the horizontal axis of the processus. **GF2 (ch 38).** *Remarks:* (Gaffney et al. 2011) combined this character in a single one together with the presence/absence of processus trochlearis pterygoidei. Splitting the character in two, as I do here, keeps separate and without several states one of the principal synapomorphies of Pan-Pleurodirans.
70. Pterygoids, trigeminal ridge: 0 = absent; 1 = thin, creating a shallow canal along the lateral margin of the pterygoid and medial of the quadrate. **GF1 (ch 72).** *Remarks:* a ridge a long the quadrate process of the pterygoid is present in many turtles, but the trigeminal ridge defined by (Gaffney et al. 2006) present in the subgroup Taphrosphyina (Bothremydidae) is very thin and I added to the definition that it is accompanied by a shallow canal along the quadrate and basisphenoid, reason because it is very thin.
71. Pterygoids, foramen posterius canalis carotici interni (fpcci): 0 = absent; 1 = present. **New character.** *Remarks*: I split character 74 from (Gaffney et al. 2006) in two here, presence/absence and position of the foramen (character 72, this study).
72. Pterygoids, position of the foramen posterius canalis carotici interni (fpcci): 0 = in

basisphenoid or basisphenoid-pterygoid suture; 1 = in prootic or close to prootic-basisphenoid suture; 2 = in quadrate or quadrate-pterygoid suture; 3 = in pterygoid-basisphenoid-quadrate suture. **GF1 (ch 74); GF2 (ch 49); JY1 (ch 56, Canalis Caroticum A); STF (ch 100, Canalis Caroticum G).** *Remarks:* the terminology of the carotid foramina has been recently redefined by (Rabi et al. 2013) pointing out that the fpcci is absent in basal turtles and paracryptodires, and the foramen present at the basisphenoid of these turtles it is actually the foramen posterius canalis carotici cerebralis, this makes that the original states defined for this character in (Gaffney et al. 2006) need to be update as I described above.

73. Pterygoids, posterior margin of pterygoid forms part of foramen posterius canalis carotici interni: 0 = no, pterygoid does not enter/touch the foramen; 1 = yes, pterygoid do enter/touch the anterior margin of foramen. **GF1 (ch 75).**
74. Pterygoids, foramen posterius canalis carotici palatinum (fpccp) (previously known as foramen caroticum laterale): 0 = present; 1 = absent. **GF1 (ch 76).** *Remarks:* see (Rabi et al. 2013) for the definition of the foramina related to carotid arteries. For crown-Testudines this character can only be visible on the dorsal surface of the pterygoid, limiting the number of fossil specimens that can be scored.
75. Pterygoids, midline contact: 0 = midline contact of pterygoids relatively long; 1 = midline contact very short. **GF1 (ch 77); JY1 (ch 44) & STF (ch 65) (Pterygoid G).**
76. Pterygoids, processus pterygoideus externus: 0 = absent; 1 = present. **GF1 (ch 72); JY1 (ch 67) (Pterygoid I).**
77. Epipterygoid, epipterygoid bone between the parietal and pterygoid: 0 = present; 1 = absent. **GF1 (ch 78); JY2 (ch 37) & STF (ch 57) (Epipterygoid A).**
78. Supraoccipital, supraoccipital-quadrate contact: 0 = absent; 1 = present. **GF1 (ch 79).**
79. Supraoccipital, crista supraoccipitalis: 0 = very short to absent; 1 = moderate to very long. **GF1 (ch 80), GF2 (ch 40); JY1 (ch 46) & STF (ch 72) (Supraoccipital A); HY2, KL, & BR (ch 28).** *Remarks:* I consider the crista supraoccipitalis in three separate characters combined and redefined from previous studies. The first one deals with the presence or absence of the crista (Character 78). The second deals with the presence or absence of the horizontal plate (Character 79), a synapomorphic character for Podocnemidae sensu (Cadena et al. 2012) following also (Gaffney et al. 2011) who also pointed out that the horizontal plate of the crista supraoccipitalis is lost in *Erymnochelys* and *Peltocephalus*, however after the direct examination of important number of specimens for these two species (see supplemental information 5), I conclude that the absence of the horizontal plate is actually atypical condition for both species. The last character of the crista supraoccipitalis is related with the shape of the lateral expansion of the horizontal plate (Character 80).
80. Supraoccipital, crista supraoccipitalis horizontal plate along its ventral edge and posterior ending level: 0 = horizontal plate absent, crista ending before the most posterior tip of squamosals; 1 = horizontal plate present, crista goes beyond the most posterior end of squamosals; 2 = horizontal plate present, crista goes beyond the most posterior end of squamosals but dorsally covered by the posterolateral

expansion of parietals (very shallow temporal emargination). **GF1 (ch 40).** *Remarks:* see remarks Character 78. The third state considers the condition in taxa with very shallow to almost absent temporal emargination, as for example *Dacquemis paleomorpha* see (Gaffney et al. 2002) (fig 2).

81. Supraoccipital, crista supraoccipitalis lateral expansion of the horizontal plate: 0 = uniform width from the anterior to the posterior aspect, ending in an acute to rounded tip in dorsal view; 1 = short, wider posteroventrally than anteroventrally, ending in a bulbous-like shape in dorsal view. **CA1 (ch 12); STF (ch 74, Supraoccipital C).** *Remarks:* see remarks Character 79.
82. Supraoccipital, dorsal exposure on skull roof: 0 = absent; 1 = present, small; 2 = present, large. **GF2 (ch 39); JY2 (ch 49) & STF (ch 73) (Supraoccipital B).**
83. Exoccipitals, foramen jugulare posterius: 0 = not formed in bone; 1 = formed by bone and open or partially closed; 2 = completely closed by bone. **GF1 (ch 82).**
84. Exoccipitals, condylus occipitalis: 0 = formed by the basioccipital plus both exoccipitals; 1 = exoccipitals only, medial contact between the exoccipitals in ventral view. **GF1 (ch 84).**
85. Exoccipitals, exoccipital-quadrate contact: 0 = absent; 1 = extensive, prootic absent; 2 = narrow, prootic present; 3 = narrow, prootic absent. **GF1 (ch 85).**
86. Exoccipitals, ventral process: 0 = absent to very short allowing a contact between the basioccipital and quadrate in ventral view of the skull; 1 = present, exoccipital reaches the basisphenoid, avoiding a basioccipital-quadrate contact in ventral view of the skull. **GF1 (ch 86).** *Remarks:* (Gaffney et al. 2011) pointed out that the ventral process of the exoccipitals is a unique condition for euraxemydids, however some bothremydids have also a ventral process of the exoccipital; for example *Labrostochelys*. The difference or better the exclusive condition of euraxemydids is that the ventral process is anteriorly extended avoiding a basioccipital-quadrate contact, condition that was added to the states here.
87. Exoccipitals, foramina nervi hypoglossi: 0 = separated on occipital surface; 1 = combined and recessed below occipital surface, almost same size of foramen jugulare posterius. **GF2 (ch 43).**
88. Basioccipital, basioccipital size ratio (length/width maximum values): 0 = ≥0.60; 1 = ≤0.59. **GF1 (ch 87), GF2 (ch 44).**
89. Basioccipital, basioccipital-opisthotic contact: 0 = absent; 1 = present. **GF1 (ch 89).**
90. Basioccipital, tubera position: 0 = absent or vey incipient located closer to medial axis of basioccipital; 1 = laterally positioned. **GF2 (ch 45).** *Remarks*: character reworded.
91. Basioccipital, horizontal occipital shelf: 0 = absent to very narrow; 1 = present, wide. **GF2 (ch 46)**. *Remarks*: character reworded.
92. Prootics, hyomandibular branch of facial nerve lies in its own canal: 0 = no; 1 = yes. **GF1 (ch 90).** *Remarks:* according to (Gaffney et al. 2006) this is a synapomorphy of Pan-Pleurodira, and a character that can only be observe in fragmentary prootics, well preserved skull with open otic chamber, or CT images.
93. Prootics, foramen stapedio-temporale (fst) orientation: 0 = more dorsally oriented on roof of otic chamber; 1 = more anteroventrally oriented, located at the anterior surface of the otic chamber. **GF1 (ch 92); STF (ch 91, Stapedial Artery C).**

94. Prootics, foramen stapedio-temporale and foramen nervi trigemini: 0 = separated by most of prootic; 1 = separated by narrow bar of prootic. **GF1 (ch 93).**
95. Prootics, ventral exposure: 0 = most of the prootic exposed ventrally; 1 = prootic about half covered by quadrate and basisphenoid; 2 = nearly all of the prootic completely covered by the quadrate, basisphenoid, and pterygoid. **GF1 (ch 94).**
96. Prootics, processus trochlearis oticum: 0 = absent: 1 = present. **GF1 (ch 96).**
97. Opisthotics, processus interfenestralis exposure: 0 = exposed ventrally; 1 = covered by bone. **GF1 (ch 99); ST (ch 56); STF (ch 82, Opisthotic D).**
98. Opisthotics, fenestra postotica closed medially: 0 = absent; 1 = present, closed medially by opisthotic-quadrate contact. **GF1 (ch 100).**
99. Opisthotics, fenestra postotica short horizontal slit: 0 = absent, more open dorsoventrally; 1 = present, short horizontal slit. **GF1 (ch 101).**
100. Opisthotics: processus paroccipitalis: 0 = projects posteriorly beyond squamosal; 1 = smaller, does not project beyond squamosal. **GF1 (ch 102).**
101. Opisthotics, thin horizontal flange on posterior margin: 0 = absent; 1 = present, just dorsal to fenestra postotica. **GF1 (ch 103).**
102. Basisphenoid, basisphenoid-quadrate contact: 0 = absent, separated by prootic or pterygoid; 1 = present, narrow occurring at the widest region of the basisphenoid; 2 = present, wide. **GF1 (ch 104), STF (ch 56, Quadrate I).** *Remarks:* after direct examination of several specimens of Pelomedusidae (see Supplementary material 1), I conclude that the most common condition is a narrow contact between these bones, taking this into account I change the order of the states 1 and 2 in contrast to (Gaffney et al. 2006) making the acquisition of a contact between basisphenoid-quadrate gradational (absent, narrow, wide).
103. Basisphenoid, ventral outline: 0 = elongated, blade-like shape, not sutured to pterygoids; 1 = triangular in shape; 2 = pentagonal in shape; 3 = elongated, blade-like shape but strongly sutured to pterygoids; 4 = V-shaped pointing anteriorly. **GF1 (ch 106).**
104. Basisphenoid, processus clinoideus: 0 = present with abducent canal; 1 = absent, canal is a groove. **GF1 (ch 107).**
105. Basisphenoid, sella turcica/dorsum sella: 0 = deep, well-defined margins: 1 = very shallow, low margins. **GF1 (ch 110).**

Lower jaw

106. Splenial, splenial bone: 0 = present: 1 = absent. **GF1 (ch 113); JY1 (ch 59, Splenial A); HY2 (ch 45); KL & BR (ch 44).**
107. Dentaries, lingual ridge in lateral view: 0 = same height or lower than the labial ridge; 1 = higher than the labial ridge. **GF1 (ch 114); HY2 (ch 43); PH (ch 7); KL & BR (ch 42).** *Remarks:* reworded from (Gaffney et al. 2006) to include the condition in outgroups of having a lingual ridge of same height of lower than labial.
108. Dentaries, dentary pits: 0 = absent; 1 = present. **GF1 (ch 115).**
109. Dentaries, U-shaped lingual ridge on symphysis: 0 = absent, lingual ridge ends in V-shaped on symphysis; 1 = present, narrow U-shaped; 2 = present, wide U-shaped. **GF1 (ch 116).** *Remarks:* extra state added for wide U-shaped taxa.

110. Dentaries, medial contact of dentaries (symphysis): 0 = fused; 1 = sutured. **GF1 (ch 117); JY1 (ch 58) & STF (ch 120) (Dentary A).**

111. Dentaries, triturating surface width: 0 = narrow, almost same width at symphysis and posteriorly; 1 = much wider posteriorly than on symphysis; 2 = very wide, anterior and posteriorly. **GF1 (ch 118); HY2, KL, & BR (ch 39).** *Remarks:* a third state was added for very wide triturating surfaces.

112. Dentaries, widely exposed on lateral surface: 0 = yes, wide posterior exposure; 1 = no, covered posteriorly by surangular. **GF1 (ch 119).**

113. Dentaries, accessory ridge: 0 = absent; 1 = present, parallel to lingual and labial ridges; 2 = present, located along the symphysis. **CA1 (ch 39).** *Remarks:* a third state was added for differentiate the position of the ridge.

114. Dentaries, medial margin shape and angle between rami: 0 = V-shaped, ≤90º angle between rami; 1 = U-shaped, obtuse, >90º angle between rami; 2 = U-shaped with a medial tip due to the posteromedial participation of the coronoid in the triturating surface, >90º angle between rami. **CA1 (ch 34).** Remarks: character modified from (Cadena 2010) including not only the angle between rami, but also the shape that these form at symphysis, expressed by the medial margin of dentaries.

115. Surangulars, foramen nervi auriculotemporalis: 0 = absent; 1 = present. **GF1 (ch 120).**

116. Coronoids, wide lateral exposure: 0 = no; 1 = yes. **GF1 (ch 121).**

117. Prearticulars, prearticular-angular contact and the anteroventral opening of the fossa Meckelii: 0 = very long contact closing most of the anteroventral opening of the fossa Meckelii; 1 = short contact, long anteroventral exposure of the fossa Meckelii. **GF1 (ch 122).** *Remarks:* I reworded this character in terms not only of the opening of the fossa Meckelii, but also of the prearticular-angular contact.

118. Articulars, processus retroarticularis: 0 = long and posteriorly projected; 1 = very short to absent; 2 = short, projected posterolaterally; 3 = short to moderately long, projected posteroventrally**. CA1 (ch 36); GF1 (ch 123).** *Remarks:* character combined and reworded from (Cadena 2010) and (Gaffney et al. 2006).

119. Articulars, foramen chorda tympani enclosed in processus retroarticularis: 0 = no; 1 = yes. **GF2 (ch 56).**

Cervical vertebrae

120. Cervical vertebrae, cervical ribs: 0 = present: 1 = absent. **GF1 (ch 124); JY1 (ch 101) & STF (ch 182) (Cervical Rib A).**

121. Cervical vertebrae, cervical postzygapophyses: 0 = all separate; 1 = some fused. **GF1 (ch 125); STF (ch 198) (Cervical Vertebra J).**

122. Cervical vertebrae, posterodorsal extension of postzygapophyses on neural spine: 0 = present; 1 = absent. **GF1 (ch 126).** *Remarks:* character reworded.

123. Cervical vertebrae, cervical centra formation: 0 = amphicoelous, platycoelous; 1 = formed central articulation. **GF1 (ch127); JY1 (ch 105) & STF (ch 187) (Cervical Articulation A); HY2 (ch 49);** and **Kl & BR (ch 48).** *Remarks:* the original definition by (Gaffney et al. 2006) deals with two features in the same character, one is the formation of a cervical centra articulation and the second

with the shape of this. I split the character in two, the formation of the cervical centra (character 123) and the second the shape of this (character 124), which is Character 58 of (Gaffney et al. 2011).

124. Cervical vertebrae, cervicals (4-6) cervical centra shape: 0 = oval to circular in shape; 1 = heterocoelic or "saddle-shaped"; 2 = wider than high, with two ventrolateral keels. **GF2 (ch 58).** *Remarks:* considering that there is considerable variation in the shape of the centra from anterior to posterior cervical vertebrae in some taxa (see (Lapparent de Broin 2000) and (Cadena 2010)), I restrict this character to cervicals 4-6.

125. Cervical vertebrae, cervical 2 biconvex: 0 = no; 1 = yes. **GF1 (ch 128); GF2 (ch 59).**

126. Cervical vertebrae, posterior cervicals with strongly developed ventral keels: 0 = absent or slightly developed in all vertebrae; 1 = present, more developed on posterior vertebrae. **CA2 (ch 37); JY1 (ch 103) & STF (ch 184) (Cervical Vertebra B); HY2 (ch 48);** and **KL & Br (ch 47).**

Caudal vertebrae

127. Caudal vertebrae, caudal articulation pattern: 0 = platycoelous, amphicoelous; 1 = formed centra varies, opisthocoelous and procoelous; 2 = all centra procoelous. **GF1 (ch 129); JY1 (ch 119) & STF (ch 205) (Caudal B); HY2, KL & BR (ch 58 and 59).**

Pelvic-Shoulder girdles

128. Pelvic girdles, tenth thoracic centrum: 0 = not incorporated into sacrum; 1 = incorporated into sacrum. **GF1 (ch 132).**

129. Pelvic girdles, pelvis-shell attachment: 0 = pelvis-shell attachment by ligaments; 1 = pelvis attached by strong sutural contact of the ischium and pubis with the plastron, and ilium with the carapace. **GF1 (ch 133); ST (ch 138); JY1 (ch 134) & STF (ch 221) (Pelvis A).**

130. Pelvic girdles, dorsal part of ilium: 0 = inclined with anterior and posterior processes; 1 = columnar expanded mediolaterally. **GF1 (ch 134).**

131. Pelvic girdles, acetabulum versus distal ilium portion distance in lateral view: 0 = distal ilium and acetabulum relatively far from midline, creating a very wide pelvis; 1 = ilium and acetabulum close to midline. **GF1 (ch 135).** *Remarks*: character reworded.

132. Shoulder girdles, coracoid dorsolongitudinal ridge: 0 = absent; 1 = present. **CA1 (ch 44).**

133. Humeri, shoulder on lateral side of head: 0 = present; 1 = absent. **GF1 (ch 137).**

Carapace

134. Carapace, geometry of the outline in terms of maximum width and length: 0 = maximum length equals or slightly longer than the maximum width which located right at the half of the carapace 1 = maximum length longer than the maximum width which is located anterior to the half of the carapace; 2 = maximum length

much longer than the maximum width which is located at the very posterior level of the carapace; 3 = maximum length longer than the maximum width which is located posteriorly close to the carapace half. **PE (ch 1).** *Remarks:* the geometry of the outline of the carapace can be very variable character, however the most important geometric patterns can be group in terms of the maximum length and width and the location of the maximum width in contrast to the half of the carapace as defined here, observing the carapace in dorsal view.

135. Nuchal, nuchal bone geometry: 0 = two or more times wider than length; 1 = width greater than length but less than two times; 2 = width approximately equals length; 3 = width approximately equals length, maximum width at least twice the anterior edge width; 4 = width less than length; 5 = nuchal bone greatly emarginated, anterior edge width equals maximum width. **GF1 (ch 139); PE (ch 3 and 4); STF (ch 128) (Nuchal C).** *Remarks:* as pointed out by (Gaffney et al. 2006) this is a very variable character within species. Here I define the character in terms of geometry of the nuchal, placing it inside squares or rectangles in order to visualize easier the different potential patterns. It is also important to notice that although the character is defined for observation in dorsal view of the carapace, the ideal observation will be in anterodorsal view, considering that most turtles have a relatively dome shell. Having this into account potential differences could occur in the geometry of the nuchal if dorsal versus anterodorsal views are compared. However, considering that almost all illustrated specimens in literature have a dorsal view and lacks the anterodorsal, I suggest using only the dorsal view for this character.

136. Nuchal, nuchal embayment: 0 = absent, 1 = present. **GF1 (ch 154).**

137. Neurals, neural series completeness: 0 = neural series reaches suprapygal; 1 = reaches costals 8; 2 = reaches costals 7; 3 = reaches costals 6; 4 = neurals absent or discontinuous. **GF1 (ch 141); JY1 (ch 68) & STF (ch 134) (Costal B).**

138. Neurals, neural series pattern: 0 = irregular, width relatively even; 1 = irregular, neurals 2 and 4 four-sided, alternating in width; 2 = regular mostly hexagonal, coffin-shaped. **GF1 (ch 145).** *Remarks:* I switched states 0 and 1, considering *Kayentachelys aprix* condition as known primitive condition.

139. Neurals, neurals number: 0 = more than eight; 1 = eight; 2 = seven; 3 = 6 or less, but at least one neural dorsally exposed. **GF1 (ch 146); HY2**, **KL & BR (ch 85 & ch 87)** and **PH (ch 33).** *Remarks:* character reworded.

140. Neurals, arrangement between neurals 1, 2, 3 and costals 1, 2, 3 and 4: 0 = neural 1 contacts costals 1 and 2, neural 2 (four-sided) only contacts costal 2; 1 = neural 1 contacts costal 1 and 2, or tip contact with costal 2, neural 2 (four-sided) only contacts costal 2, neural 3 contacts costal 2, 3, and 4; 2 = neural 1 (four-sided) contacts costal 1 and 2, neural 2 contacts costal 2 and 3, neural 3 (four-sided) only contacts costal 3. 3 = neural 1 (four-sided) only contacts costal 1, neural 3 contacts costal 2. **CA2 (ch 43); GF1 (ch 144); JY1 (ch 64) & STF (ch 129) (Neural A).** *Remarks:* I combined the four-sided neural character of Gaffney et al. (2006) with the arrangement of the three most anterior neurals and costals defined by (Cadena et al. 2012).

141. Neurals, neural and costal bones with keels or knobs: 0 = present; 1 = only neurals 1-5; 2 = absent, smooth surface. **GF2 (ch 65); JY1 (ch 61) & STF (ch**

122) (Carapace B) and **HY2, KL, & BR (ch 84).** *Remarks:* I modified the character adding a third state of keels not only on neurals but also on costals.

142. Costals, iliac scar position: 0 = absent or very incipient scar on costals 7 and 8; 1 = on costals 7, 8, pygal, and peripheral 1; 2 = on costal 8 and suprapygal; 3 = on costals 7, 8, and suprapygal; 4 = restricted to costals 7 and 8. **GF1 (ch 142).** *Remarks:* I added to states, one representing the condition of *Platychelys oberndorferi* important to understand the evolution of the pelvic girdle in Pan-Pleurodira and state 2 for those taxa with iliac scar restricted to costal 8 and suprapygal.
143. Costals, costal 1 maximum length versus costal 2 maximum length: 0 = costal 1 shorter than the length of costal 2; 1 = costal 1 equals or slightly longer than twice the length of costal 2; 2 = costal 1 more than twice longer than the length of costal 2. **GF1 (ch 143).** *Remarks:* states were modified and split in three to cover all possible relationships with the length of costals 1 and 2.
144. Costals, arrangement between costal 1 and axillary process (buttress): 0 = axillary process of hyoplastron restricted to peripherals; 1 = short axillary process entering into costal 1; 2 = long axillary process reaching costal 2 or at the contact between costal 1 and 2; 3 = long axillary process reaching almost the central point of costal 1, accompanied by a very strong and well defined costal rib. **GF1 (ch 148).** *Remarks:* I added to the definition of this character the length and position of the axillary process onto costal 1. The shape of the axillary process or its scar is even more variable than its entrance position from peripherals and length, so I avoid the use of the shape, however, some of them can be apomorphic or generic useful characters (see (Lapparent de Broin & Wermer 1998); (Cadena et al. 2012b) (character 176).
145. Costals, inguinal process (buttress): 0 = absent or very short touching most posterolateral portion of costal 5; 1 = inguinal process of hypoplastron long on costal 5; 2 = inguinal process long and restricted to costal 4. **GF1 (ch 150)**. *Remarks:* character reworded and I added a third state considering the condition in *Chelus* spp. see (Cadena et al. 2008).
146. Peripherals, axillary process extent on ventral surface of peripherals: 0 = reaches peripheral 2; 1 = reaches anteromedial or medial margin of peripheral 3; 2 = reaches posteromedial margin of peripheral 3; 3 = restricted to peripheral 4. **GF1 (ch 149).** *Remarks:* I split state 2 of (Gaffney et al. 2006) in states 2 and 3 here. There are taxa with hyoplastron only reaching the peripheral 4 and this should be considered as a different state and not just a variant of posteromedial peripheral 3 contact.
147. Peripherals, length of contact between peripheral 1 and costal 1 in dorsal view: 0 = anterior margin of peripheral 1 less than twice the length of the contact between peripheral 1 and costal 1; 1 = anterior margin of peripheral 1 is twice or more the length of peripheral 1 and costal 1 contact; 2 = contact between peripheral 1 and costal 1 absent due to a large nuchal. **GF1 (ch147).** *Remarks:* I switched the states order, considering that the most primitive condition for this character can be obtained from *Kayentachelys aprix* and *Notoemys* spp. (Gaffney et al. 2006) defined a fourth state (no contact due to small nuchal) to include the condition in *Araripemys barretoi*, however this is incorrect, because there is a contact between

the small triangular in shape peripheral 1 and costal 1, so state 3 was removed for this character.

148. Peripherals, peripheral 1 anterior margin width versus lateral margin length: 0 = lateral margin longer than the anterior margin; 1 = anterior margin width longer than lateral margin; 2 = anterior equals to lateral margin length. **PE (ch 2).** *Remarks:* (Pérez-García & Lapparent de Broin 2013) did not provide details of where the length/width should be measurement in the peripheral 1, here I redefined the character in terms of the anterior margin width and lateral margin length in dorsal view of the carapace.

149. Suprapygal, shape of suprapygal in contact with the pygal: 0 = pentagonal to trapezoidal in shape; 1 = almost triangular tapering anteriorly. **New character.**

150. Cervical, cervical scute(s): 0 = present; 1 = absent. **GF1 (ch 138); JY1 (ch 70) & STF (ch 138) (Cervical A).**

151. Marginals, marginal scute 1 anterior margin width versus lateral margin length: 0 = lateral margin longer or equal than the anterior margin; 1 = anterior margin width longer than lateral margin. **PE (ch 6).** *Remarks:* in contrast to the states in character 149 where I separate equals from longer or wider conditions, in the case of marginals 1, they never are much longer than wide, so I combine the case where they are slightly longer than wide with equal width-length condition.

152. Marginals, marginal scute 1 proportion overlapping the anterior margin/portion of peripheral bone 1: 0 = between 30 to 60%; 1 = less than 30%; 2 = more than 60%. **PE (ch 8).** *Remarks:* character redefined in terms of the proportion of peripheral 1 covered by marginal 1 at the anterior margin, also adding a third state where the marginal 1 overlaps more that 60% of peripheral 1.

153. Supramarginal scutes: 0 = present; 1 = absent. **GF1 (ch 151).**

154. Vertebrals, vertebral scutes 2-4 width versus pleurals 2 and 3 width: 0 = vertebral 2-4 wider than pleurals 2 and 3; 1 = vertebrals 2-4 slightly squared, equal to or narrower than pleurals 2 and 3. **CA3 (ch 178); GF1 (ch 152); JY1 (ch 73) & STF (ch 141) (Vertebral B).** *Remarks:* I restrict this character to vertebrals 2-4 and pleurals 2 and 3, other anterior or posterior vertebrals and pleurals have wide variability, most of the cases variations that constitute apomorphies for some taxa.

155. Vertebrals, vertebral scute 1 reaches anterior margin of carapace: 0 = no, medial contact between marginals 1 or cervicals present; 1 = yes. **GF1 (ch 153).**

156. Vertebrals, vertebral scute 1 shape of the anterolateral margins: 0 = lateral margins parallel to midline axis of carapace and anterior margin straight at the medial level; 1 = lateral margins anteriorly divergent, anterior margin medially convergent in acute tip or convex; 2 = lyre-shaped vertebral 1, lateral margins divergent at the most anterior corner, anterior margin with a medial notch. **PE (ch 9).** *Remarks:* I redefined this character in terms of the lateral and anterior margins of vertebral 1.

157. Vertebrals, vertebral scute 1 overlap onto anterior peripherals and nuchal: 0 = overlapping nuchal and peripherals 1; 1 = overlapping nuchal, peripherals 1 and 2; 2 = overlapping only nuchal. **CA3 (ch 177).**

158. Vertebrals, lateral position of the sulcus between vertebral scutes 3 and 4 in taxa with five vertebrals: 0 = sulcus positioned on costal 6; 1 = sulcus positioned on costal 5. **JY1 (ch 74) & STF (142) (Vertebral C).** *Remarks:* I modified the

definition of the character, instead of using the position of the sulcus on neurals, I use the position on costals 5 or 6, doing this taxa with completely absent of neurals can be scored for the character too.

159. Vertebrals, most posterior vertebral scute overlapping peripheral 10: 0 = no, overlapping only peripheral 11 and pygal, or restricted to suprapygal; 1 = yes, overlapping pygal, peripheral 10 and 11. **New character.** *Remarks*: this character resembles character 9 (width of vertebral 5) of (Pérez-García & Lapparent de Broin 2013) because when the vertebral 5 or 6 is the widest generally reaches peripheral 10, I consider that with the new definition the character can be more useful in cases where not all vertebrals are preserved or is difficult to establish clear difference in the width of them.

160. Thoracic rib, thoracic rib 1 versus 2: 0 = thoracic rib 1 large and separated from thoracic rib 2; 1 = thoracic rib 1 reduced, almost same size of the medial portion of thoracic rib 2, both separated by an oval elongated space; 2 = thoracic rib 1 shorter than the medial exposed portion of thoracic rib 2, very small oval space between them. **GF1 (ch155).** *Remarks:* (Gaffney et al. 2006) separated this character in two states, however after examination of important number of extant pleurodires (see Supplementary material 1), I conclude that state 1 of (Gaffney et al. 2006) can be split in two distinct conditions, related not only to the size of thoracic rib 1 versus thoracic rib 2, but also in terms of the separation between them.

161. Thoracic rib, articulation facet on thoracic rib 1: 0 = absent; 1 = present, facet or tubercle on anterior margin of thoracic rib 1. **GF1 (ch 157).**

162. Thoracic vertebrae, shape of thoracic vertebrae: 0 = cylindrical, longer than wide, keeled ventrally; 1 = smooth and flat ventrally, hexagonal in shape with central lateral notch**. New character.**

163. Costovertebral tunnel, costovertebral tunnel formed by thoracic ribs and vertebrae: 0 = present, anteriorly and posteriorly only; 1 = present, wide for the entire length; 1 = absent. **GF1 (ch 156).**

Plastron

164. Mesoplastra, medial contact between mesoplastra: 0 = present; 1 = absent, mesoplastron laterally restricted, wider than long and reduced in size; 2 = absent, mesoplastron laterally restricted, roughly equidimensional; 3 = mesoplastron absent. **GF1 (ch 158); JY1 (ch 85) & STF (ch 160) (Mesoplastron A)** and **AN (ch 122).** *Remarks:* character reworded.

165. Entoplastron, shape and participation of the entoplastron in the anterior plastral lobe margin: 0 = pentagonal in shape, anterior margin of entoplastron included in the anterior margin of plastron (anterior entoplastron process); 1 = four-sided diamond or rhombus-shaped, with not contribution to the anterior margin of the plastron, due to medial contact of epiplastra; 2 = triangular in shape, with not contribution to the anterior margin of the plastron, due to medial contact of epiplastra; 3 = entoplastron absent. **CA4 (ch 75); GF1 (ch159 and ch 160); JY1 (ch 78) & STF (ch 149 and 153) (Entoplastron A and E).** *Remarks:* the shape of the entoplastron is in part conditioned by the presence or not of a medial

contact between epiplastra, I suggest to consider these two characters in a single one as defined here.

166. Entoplastron, posterior end of the entoplastron reaching the axillary notch level of plastron: 0 = absent, entoplastron located very anterior to the axillary notch level; 1 = present. **CA4 (ch 179).** *Remarks:* character restricted to turtles lacking long posterior entoplastron process (most basal Testudines).

167. Epiplastra, anteriorly projected tuberosities of the epiplastron: 0 = present, two or more; 1 = absent. **CA4 (ch 77); GF1 (ch 163)**. *Remarks:* (Gaffney et al. 2006) defined this character as gular scute projections, however the tuberosities (projections) are part of the anterior portion of the epiplastron and not of the scutes. This character is better defined as part of the epiplastra, as I suggest here.

168. Epiplastra, anterior plastral lobe margin shape: 0 = dentate straight to slightly convex (trapezoidal); 1 = smooth, highly convex; 2 = smooth, acute tip. **CA4 (ch 77); GF1 (ch 164); PE (ch 11).** *Remarks:* character 77 of (Cadena et al. 2013) is split in character 167 and character 168 (this study), also character 164 of (Gaffney et al. 2006) is split in character 167 and character 168 (this study), the logic behind this changes is that the anterior plastral lobe have three well defined features: presence or absence of tuberosities (character 166, this study); its shape more clearly defined by its anterior margin (character 167, this study); and the proportion between its length and its width (character 168, this study).

169. Epiplastra, anterior plastral lobe width/length ratio: 0 = maximum width at the hyoplastron notch (base of the anterior plastral lobe) almost twice the maximum length; 1 = maximum width slightly longer than the maximum length; 2 = maximum width almost three times the maximum length. **GF1 (ch 164).** *Remarks:* character redefined.

170. Epiplastra, anterior plastral lobe reaches carapace anterior margin: 0 = yes, same level of beyond the anterior margin of carapace; 1 = no, well posterior to the anterior margin of carapace. **GF1 (ch 173).**

171. Hyoplastra, central fontanelle at the hyo-hypoplastron suture: 0 = absent; 1 = present. **CA4 (ch 79).** *Remarks:* character can have ontogenetic variability (fontanelle present in hatching/juveniles for some taxa). So avoid using it if there is evidence of specimens in early ontogenetic stages having a central fontanelle.

172. Hyoplastra, plastral bridge length at the axillary and inguinal notches: 0 = long; 1 = very short. **CA1 (ch 50).**

173. Hyoplastra, axillary musk duct location: 0 = musk duct are absent; 1 = located at the most anterior tip of the sutural contact between hyoplastron and peripherals (in buttress), before axillary notch; 2 = present in bridge. **GF2 (ch 69).** *Remarks:* character reworded and extra state added for cases where the duct(s) are absent.

174. Hyoplastra, number of musk ducts in bridge: 0 = one pair; 1 = three pairs; 2 = four or more pairs, with or without an extra par in buttress. **GF2 (ch 70).** *Remarks:* character reworded.

175. Xiphiplastra, anal notch: 0 = absent; 1 = present. **GF1 (ch 174); JY1 (ch 87) & STF (163) (Xiphiplastron A).**

176. Xiphiplastra, size and shape of ischial scar: 0 = large linear or V-shaped; 1 = small, oval to circular. **GF1 (ch 168).** *Remarks:* I removed state 0 of (Gaffney et al. 2006) for this character considering that is redundant with character 129 (this

study).

177. Xiphiplastra, ischial scar position related to the anal notch of xiphiplastron: 0 = the most posterior tip of the scar reaches the level of the anal notch; 1 = the most posterior tip is located anteriorly, before the anal notch level. **CA3 (ch 180).**

178. Gular, gular scutes: 0 = two (one pair); 1 = one gular scute. **GF1 (ch 162); JY1 (ch 91) & STF (ch 167) (Gular A).** *Remarks:* intergular for (Gaffney et al. 2006) and other authors.

179. Humerals, medial contact between humeral scutes: 0 = present; 1 = absent, even just a touching tips. **CA2 (ch 46); GF1 (ch 170); PE (ch 14).** *Remarks:* the arrangement between gular, extragulars (gulars for (Gaffney et al. 2006)and other authors) and humeral scutes can be extremely variable to generic and specific level, specially in terms of the size or the length/width of contacts between scutes as (Pérez-García & Lapparent de Broin 2013) described in detail for *Neochelys* spp. Here I consider four characters (179-182) that describe the most common arrangements between these anterior plastral scutes, excluding autapomorphic configurations for single species.

180. Extragulars, medial contact of extragular scutes: 0 = absent; 1 = present, restricting the posterior advance of the gular; 2 = extragulars absent. **CA2 (ch 46); GF1 (ch 170); PE (ch 14).** *Remarks:* see character 179.

181. Extragulars, covering most or at least a small portion of entoplastron: 0 = no; 1 = yes. **CA2 (ch 46); GF1 (ch 170); PE (ch 14).** *Remarks:* see character 179.

182. Extragulars, anterior margin width: 0 = almost the same width as the anterior margin of gular; 1 = wider than the anterior margin of gular; 2 = much wider than the anterior margin of gular, which is triangular in shape; 3 = much narrower than the anterior margin of gular. **CA2 (ch 46); GF1 (ch 170); PE (ch 14).** *Remarks:* see remarks character 179 .

183. Pectorals, pectoral scute on entoplastron: 0 = absent; 1 = present. **GF1 (ch 165); STF (ch 174) (Humeral B).**

184. Pectorals, pectoral scute on epiplastron: 0 = absent; 1 = present, crossing the lateral margin of epiplastron-hyoplastron suture; 2 = present, very anterior to the epiplastron-hyoplastron suture. **GF1 (ch 166)**. *Remarks*: a third state was added to this character.

185. Pectorals, pectoral scute on mesoplastron: 0 = present; 1 = absent. **GF1 (ch 167).**

186. Inframarginals, inframarginal scutes on bridge: 0 = present; 1 = absent. **GF1 (ch 171); JY1 (ch 100, Inframarginal A) & STF (ch 179, 180, and 181) (Inframarginals, A, B and C).** *Remarks:* primitive condition as for *Kayentachelys aprix.*

187. Abdominals, length abdominals versus femorals scutes at the midline of plastron: 0 = abdominals longer or almost same length than femorals: 1 = femorals twice the length of abdominals; 2 = abdominal scutes do not meet at the midline, due to the presence of a central fontanelle. **GF1 (ch 172); JY1 (ch 98) & STF (ch 177) (Abdominal A).** *Remarks*: (Gaffney et al. 2006) defined this character in terms of the length of abdominals versus anal scutes, in my opinion a better definition is in terms of abdominals versus femorals, avoiding high variations caused by the depth of the anal notch of xiphiplastra.

Characters excluded or combined from previous studies

From GF1

Character 2. Lacrimal: 0 = present; 1 = absent. *Reason to be removed from the list:* this is an uninformative character for Pan-Pleurodiran turtles, useful to differentiate stem-testudines.

Character 3. Lacrimal: lacrimal foramen: 0 = present; 1 = absent. *Reason to be removed from the list:* this is an uninformative character for Pan-Pleurodiran turtles, useful to differentiate stem-testudines.

Character 9. Prefrontals-vomer, fissura ethmoidalis: 0 = very wide; 1 = narrower. *Reason to be removed from the list:* this is an uninformative character for Pan-Pleurodiran turtles, polymorphic in cryptodires.

Character 19. Supratemporal, supratemporal bone: 0 = present; 1 = absent. *Reason to be removed from the list:* this is an uninformative character for Pan-Pleurodiran turtles, useful to differentiate stem-testudines.

Character 36. Premaxilla, midline dorsal process: 0 = present, meeting nasals; 1 = absent, or low; 2 = present, at least partially separating nares. *Reason to be removed from the list:* apomorphic for *Proganochelys quenstedti* among Testudinata. Stages 1 and 2 are very close in all pleurodires and very subjective for separation.

Character 46. Vomer, vomerine teeth: 0 = present; 1 = absent. *Reason to be removed from the list:* this is an uninformative character for Pan-Pleurodiran turtles, useful to differentiate stem-testudines.

Character 53. Quadrate, stapes contained in bony canal: 0 = stapes not completely contained in bone; 1 = stapes completely enclosed by bony incisor columellae auris. *Reason to be removed from the list*: character fused in character 56 (states 1 and 3).

Character 61. Quadrate, fully formed cavum tympani: 0 = no; 1 = yes. *Reason to be removed from the list:* I combined this character with the character 57 (cavum tympani, arrangement between the quadrate, squamosal, opisthotic, stapes and Eustachian tube), because the formation of the cavum tympani is related to the closing of the quadrate and the incisura columella auris.

Character 62. Quadrate, cavum tympani with acute posterior edge: 0 = no; 1 = acute edge, also enclosing stapes. *Reason to be removed from the list:* I combined this character with the character 57 (cavum tympani, arrangement between the quadrate, squamosal, opisthotic, stapes and Eustachian tube), state 3.

Character 63. Quadrate, middle ear with complete lateral wall: 0 = no; 1 = yes. *Reason to be removed from the list:* this is an uninformative character for Pan-Pleurodiran turtles,

useful to differentiate stem-testudines.

Character 64. Quadrate, cavum tympani curved dorsally: 0 = no; 1 = yes. *Reason to be removed from the list:* this is an uninformative character for Pan-Pleurodiran turtles, useful to differentiate stem-testudines.

Character 65. Quadrate, covers opisthotic laterally: 0 = no; 1 = yes. *Reason to be removed from the list:* this is an uninformative character for Pan-Pleurodiran turtles, useful to differentiate stem-testudines.

Character 66. Quadrate, pocket for stapes articulation: 0 = no; 1 = yes. *Reason to be removed from the list:* this is an uninformative character for Pan-Pleurodiran turtles, useful to differentiate stem-testudines.

Character 67. Quadrate, cranioquadrate space: 0 = relatively open; 1 = well defined canal. *Reason to be removed from the list:* this is an uninformative character for Pan-Pleurodiran turtles, useful to differentiate stem-testudines.

Character 81. Supraoccipital, wide occipital plate: 0 = wide occipital plate with posteriorly concave depression; 1 = narrower occiput. *Reason to be removed from the list:* this is an uninformative character for Pan-Pleurodiran turtles, useful to differentiate stem-testudines.

Character 83. Exoccipital, recessus scalae tympani: 0 = not formed in bone; 1 = formed by bone, also forming fenestra perilymphatica. *Reason to be removed from the list:* this is an uninformative character for Pan-Pleurodiran turtles, useful to differentiate stem-testudines.

Character 88. Basioccipital, basioccipital thick: 0 = basioccipital and basisphenoid relatively thick in cross section; 1 = thinner. *Reason to be removed from the list:* this is an uninformative character for Pan-Pleurodiran turtles, useful to differentiate stem-testudines.

Character 91. Prootic, foramen stapedio-temporale: 0 = not a canal; 1 = foramen and canal. *Reason to be removed from the list:* this is an uninformative character for Pan-Pleurodiran turtles, useful to differentiate stem-testudines.

Character 97. Prootic, plane of fenestra ovalis: 0 = inclined from the vertical; 1 = very close to vertical. *Reason to be removed from the list:* this is an uninformative character for Pan-Pleurodiran turtles, useful to differentiate stem-testudines.

Character 98. Opisthotic, processus interfenestralis expanded ventrally: 0 = narrow ventrally; 1 = expanded ventrally. *Reason to be removed from the list:* this is an uninformative character for Pan-Pleurodiran turtles, useful to differentiate stem-testudines.

Character 105. Basisphenoid, interpterygoid vacuity: 0 = large and open; 1 = small or absent. *Reason to be removed from the list:* this is an uninformative character for Pan-Pleurodiran turtles, useful to differentiate stem-testudines.

Character 108. Basisphenoid, skull akinetic: 0 = no, basipterygoid articulation present; 1 = yes, basipterygoid articulation sutured. *Reason to be removed from the list:* this is an uninformative character for Pan-Pleurodiran turtles, useful to differentiate stem-testudines.

Character 109. Basisphenoid, cultriform process: 0 = rod-like, thin; 1 = broad, flat, covered ventrally. *Reason to be removed from the list:* this is an uninformative character for Pan-Pleurodiran turtles, useful to differentiate stem-testudines.

Character 111. Basisphenoid, ventral tubercle: 0 = single tubercle formed by basisphenoid and basioccipital; 1 = paired tubercles; 2 = tubercles absent. *Reason to be removed from the list:* this is an uninformative character for Pan-Pleurodiran turtles, useful to differentiate stem-testudines.

Character 112. Columella auris, footplate: 0 = absent, stapes thick; 1 = wide footplate present, stapes thin. *Reason to be removed from the list:* this is an uninformative character for Pan-Pleurodiran turtles, useful to differentiate stem-testudines.

Character 130. Shoulder girdle, coracoid foramen: 0 = present; 1 = absent. *Reason to be removed from the list:* this is an uninformative character for Pan-Pleurodiran turtles, useful to differentiate stem-testudines.

Character 131. Shoulder girdle, coracoid shape: 0 = coracoid a flat plate; 1 = coracoid columnar. *Reason to be removed from the list:* this is an uninformative character for Pan-Pleurodiran turtles, useful to differentiate stem-testudines.

Character 136. Pelvic girdle, thyroid fenestra: 0 = separate; 1 = confluent. *Reason to be removed from the list:* the confluent condition is present in all pleurodires and also outgroups considered in this study. This character is useful to differentiate stem-testudines.

Character 140. Carapace, pygal notch: 0 = present; 1 = absent. *Reason to be removed from the list:* this is an uninformative character for Pan-Pleurodiran turtles, useful to differentiate stem-testudines. *Platychelys oberndorferi* has a very shallow pygal notch, this can be also the case in some cryptodires, however it is not so deep and well defined as in basal stem-testudines.

Character 161. Plastron, dorsal epiplastral process: 0 = large, reaching or nearly reaching carapace; 1 = small or absent. *Reason to be removed from the list:* this is an uninformative character for Pan-Pleurodiran turtles, useful to differentiate stem-testudines.

Character 169. Plastron, posterior lobe wider than pelvis: 0 = no, pelvis visible in ventral view; 1 = yes, pelvis hidden by wide posterior lobe. *Reason to be removed from the list:* this is an uninformative character for Pan-Pleurodiran turtles, useful to differentiate stem-testudines.

Character 175. Shell, surface texture: 0 = surface rough with radiating ridges diverging from posteromedial part (growth center) of vertebral and pleural scale areas; 1 = weakly granulated polygons; 2 = strongly granulated polygons; 3 = fine, striated ridges; 4 = smooth; 5 = numerous small pits; 6 = texture as in *Dortoka. Reason to be removed from the list:* I agree with the senior author of (Gaffney et al. 2006) in removed this character because the difficulty of identify objectively and consistently the surface texture of shell over a wide range of taxa, and I suggest its used only as part of diagnosis of taxa.

From GF2

Character 15. Premaxillae: pinched snout: 0 = absent; 1 = concave outline near premaxilla-maxilla contact, snout not elongated; 2 = concave outline posterior to premaxilla-maxilla contact, snout elongated. *Reason to be removed from the list:* the pinched snout is a feature resulting of the anterior projection of the prefrontals over the aperture narium and also of their dorsal inflation, this character is better defined by the way that the prefrontals cover the apertura narium externa (character 5, this study).

Character 18. Maxillae: triturating surface convexity: 0 = absent or shallow; 1 = deep. *Reason to be removed from the list:* apomorphic for two species of *Bairdemys* genus.

Character 21. Maxillae, meet broadly on midline: 0 = no; 1 = yes. *Reason to be removed from the list:* extremely homoplastic as was pointed out by (Gaffney et al. 2011)).

Character 27. Palatines, orbitalis posterior pocket in septum orbitotemporale: 0 = absent; 1 = present. *Reason to be removed from the list:* the best specimen figured by (Gaffney et al. 2011) for observation of this character is in my opinion *Mogharemys blanckenhorni* (Pag 121, Fig 50), however, the called "posterior pocket" is not really a well defined feature at the fossa orbitalis, and not remarkable different from the shape of the anterior surface of the septum orbitotemporale of extant podocnemidids. I keep this character out because it is not very well defined or illustrated by (Gaffney et al. 2011) CT images of stereogynis could help to test the validity of this character and to reach a better definition.

Character 49. Prootic, foramen posterius canalis carotici interni: 0 = in prootic; 1 = in basisphenoid with cavum pterygoidei: 2 = variably in pterygoid, quadrate basisphenoid; 3 = pterygoid and basisphenoid. *Reason to be removed from the list:* character included in the redefined character 73 this study.

Character 67. Carapace, costal 2 anterior edge thickened near buttress: 0 = no; 1 = yes. *Reason to be removed from the list:* the definition of the character is poor in terms not only of lacking figures or photographs illustrating the thickened, but also of how to

differentiate from the average thickness of the costal bones.

From CA1

Character 37. Surangular, well extended anteriorly: 0 = absent; 1 = present. *Reason to be removed from the list:* after examination of more specimens of extant podocnemidids, I conclude that there is strong variability of this character in the degrees of the anterior extension of surangular bone.

Character 40. Dentary, narrow and elongated ridge, located in the medial margin on the ventral surface: 0 = absent; 1 = present. *Reason to be removed from the list:* this is an autapomorphic character of *Cerrejonemys wayuunaiki* among Pan-Pleurodira.

Character 49. Carapace, lateral thickness of the shell: 0 = ≤20 mm; 1 =≥20 mm. *Reason to be removed from the list:* this is an autapomorphic character of *Cerrejonemys wayuunaiki* among Pan-Pleurodira.

From CA2

Character 9. Prefrontal, prefrontal-postorbital contact: 0 = absent; 1 = present. *Reason to be removed from the list:* this is an autapomorphic character of *Carbonemys cofrinii* among Pan-Pleurodira.

From PE1

Character 7. Overlap of the marginal 1 on the latero-anterior margin of the nuchal: 0 = marginal 1 overlapping more than one third of the latero-anterior margin; 1 = marginal 1 overlapping less than one third of the latero-anterior margin. *Reason to be removed from the list:* this becomes a redundant character with character 152 of this study, and it is variable at generic and specific level, depending of the inclination of the posterior margin of marginal 1.

Character 12. Ratio between the length of the entoplastron and its distance with the pectoro-abdominal sulcus: 0 = entoplastral length longer than two times that distance; 1 = entoplastral length less than two times that distance. *Reason to be removed from the list:* the second state is scored for three species of *Neochelys*: *N. eoceanica, N. zamoraensis, N. salmanticensis* however from the figure and photos provide in (Broin 1977) and (Jiménez-Fuentes 1968; Jiménez-Fuentes 1992) it is clear that the differences are not significant and can fall easily inside ontogenetic or intraspecific variations.

Character 13. Lateral margins of the posterior plastral lobe: 0 = rounded; 1 = straight. *Reason to be removed from the list:* this is a highly variable character in individuals of same species of extant podocnemidids, and apomorphic for *Neochelys zamoraensis* as

scored in Table 2 of (Pérez-García & Lapparent de Broin 2013).

Character 15. Length of the dorsal shell: 0 = near 25 cm; 1 = greater than 35 cm. *Reason to be removed from the list:* I avoid the use of this character that can be related to ontogenetic stages, sexual dimorphisms variations, and an affected by a poorly documented number of individuals for some species.

Changes in scoring

From GF1 and Romano et al (2014)

Character 8 (Character 7 this study)

Araripemys barretoi, Laganemys tenerensis	from (0) to (?)
Pelomedusa subrufa, Pelusios castaneus, Dirqadim schaefferi	from (0) to (-)

Reason for the change: if the vomer is absent or not preserved, this character should be code as not applicable (-) for those lacking the vomer, and (?) for those with dubious presence/absence or contact with the prefrontal.

Character 11 (Character 11 this study)

Sokatra antitra, Pelomedusa subrufa, Pelusios castaneus Cearachelys placidoi, Galianemys emringeri, Galianemys whitei, Kurmademys kallamedensis, Kinkonychelys rogersi, Foxemys mechinorum, Polysternon provincial, Araiochelys hirayamai, Zolhafah bella, Rosasia soutoi, Taphrosphys sulcatus, Taphrosphys congolensis, Taphrosphys ippolitoi, Azabbaremys moragjonesi, Acleistochelys maliensis, *Labrostochelys galkini, Phosphatochelys tedfordi, Ummulisani rutgersensis, Rhothonemys brinkmani, Nigeremys gigantean, Arenila krebsi* from (0) to (1)

Reason for the change: the condition for all these taxa, following the redefinition of character states made here, fit better in (1) than in (0).

Character 16 (Character 16 this study)

Kayentachelys aprix	from (0) to (?)

Reason for the change: as the specimen is preserved is not completely clear if the contact exists or not.

Character 39 (Character 25 this study)

Phosphatochelys tedfordi, Ummulisani rutgersensis	from (2) to (5)

Reason for the change: a contact between maxilla and quadrate with very shallow cheek emargination is present for both taxa. See also the character remarks for discussion about states changes.

Character 27 (Character 30 this study)

Laganemys tenerensis	from (0) to (?)

Reason for the change: the orbits are still filled with matrix, avoiding the observation of this character.

Character 35 (Character 38 this study)

Atolchelys lepida	from (1) to (0)

Reason for the change: photographs and figures presented for this taxon by (Romano et al. 2014) show absence of deep and rounded pits as those exhibit by other bothremydids.

Character 45 (Character 46 this study)

Laganemys tenerensis	from (?) to (1)

Reason for the change: vomer is present in this taxon, see (Sereno & ElSahfie 2013)

Character 76 (Character 74 this study)

Laganemys tenerensis	from (0) to (?)
Atolchelys lepida	from (1) to (?)

Reason for the change: both species lack of skulls enough prepared/preserved to be able to score this character. In the case of *L. tenerensis*, CT data was produced, however is not available for public access and corroboration.

Character 77 (Character 75 this study)

Ummulisani rutgersensis	from (0/1) to (?)
Rhothonemys brinkmani	from (0) to (?)
Bothremys kellyi	from (1) to (?)

Reason for the change: all these taxa do not preserve completely the pterygoids-basisphenoid area.

Character 78 (Character 77 this study)

Atolchelys lepida	from (1) to (?)

Reason for the change: not enough prepared/preserved to be able to score this character.

Character 79 (Character 78 this study)

Brasilemys josai from (?) to (0)

Reason for the change: character can be scored following (Lapparent de Broin 2000)

Character 106 (Character 103 this study)

Atolchelys lepida from (4) to (1)

Reason for the change: the shape of basisphenoid is as in state 1.

Character 107 (Character 104 this study)

Atolchelys lepida from (0) to (?)

Reason for the change: character is not visible as the specimen is preserved/prepared.
Hamadachelys escuilliei from (?) to (0)

Reason for the change: can be scored from the CT images available on Digimorph.

Character 110 (Character 105 this study)

Atolchelys lepida, Araripemys barretoi, Laganemys tenerensis from (0) to (?)

Reason for the change: character is not visible as the specimens are preserved/prepared, or just by the information provided by the respective references

Character 116 (Character 109 this study)

Hamadachelys escuilliei from (0) to (1)

Reason for the change: U-shaped (1) is the condition for this taxon, see (Gaffney et al. 2006) (Fig. 251).

Character 117 (Character 110 this study)

Hamadachelys escuilliei from (0) to (?)

Reason for the change: condition unknown as specimen preserved.

Session 2. Characters figures.

Figures used in this guide, were taken, modified, or redraw in different degree from previous studies as follow:

Cadena & Joyce 2015
Characters: 134,135,138,139,140,147,148,149,151,152,153,159,164,165
DigiMorph 2015
Characters: 16,17,31,35,66,74,93,94,96,104,105,129
Gaffney 1979
Characters: 48,68,77,92,124,125
Gaffney 1990
Characters: 120,121,130,131,132,133
Gaffney & Wood 2002
Characters: 19,20,64,81
Gaffney et al. 2002
Characters: 80,82
Gaffney et al. 2011
Characters: 14,15,18,19,20,21,22,23,24,25,26,27,28,29,32,33,36,39, 40,42,43,44,45,46,52,53,54,56,60,61,63,64,78,81,82,84,87,90,95,102 111,113,114,135,183
Gaffney et al. 2006
Characters: 4,5,8,11,12,13,17,19,21,22,23,24,25,26,27,28,29,30,31, 32,33,34,35,36,37,38,39,40,41,42,43,46,47,49,50,55,56,57,58,59,62, 65,66,67,68,69,70,71,72,73,75,76,78,78,82,83,84,85,86,88,89,90,94, 95,97,98,99,100,101,102,103,104,105,107,108,109,110,111,112,115, 116,117,118,135,137,139,140,147,148,157,158,166,169,170,175,176 177,187
Joyce 2007
Characters: 1,2,3,4,5,6,7,8,9,10,11,12,13,14,15,25,46,55,56,60,61,63 71,76,83,85,103,106,122,123,126,127,134,136,137,138,139,140,143 149,150,151,152,153,154,155,156,157,158,159,164,166,167,168,169 170,171,172,175,178,183,184,185,186,187
Lapparent de Broin 2000
Characters: 85
Meylan 1996
Characters: 135,136,140,155,165,168,171,187
Pérez-García and Lapparent de Broin 2013
Characters: 156
This study
Characters: 65,66,67,69,91,113, 114,118,119,124,125,128,129,141,142, 144,145,146,160,161,162,163,173,174,179,180,181,182

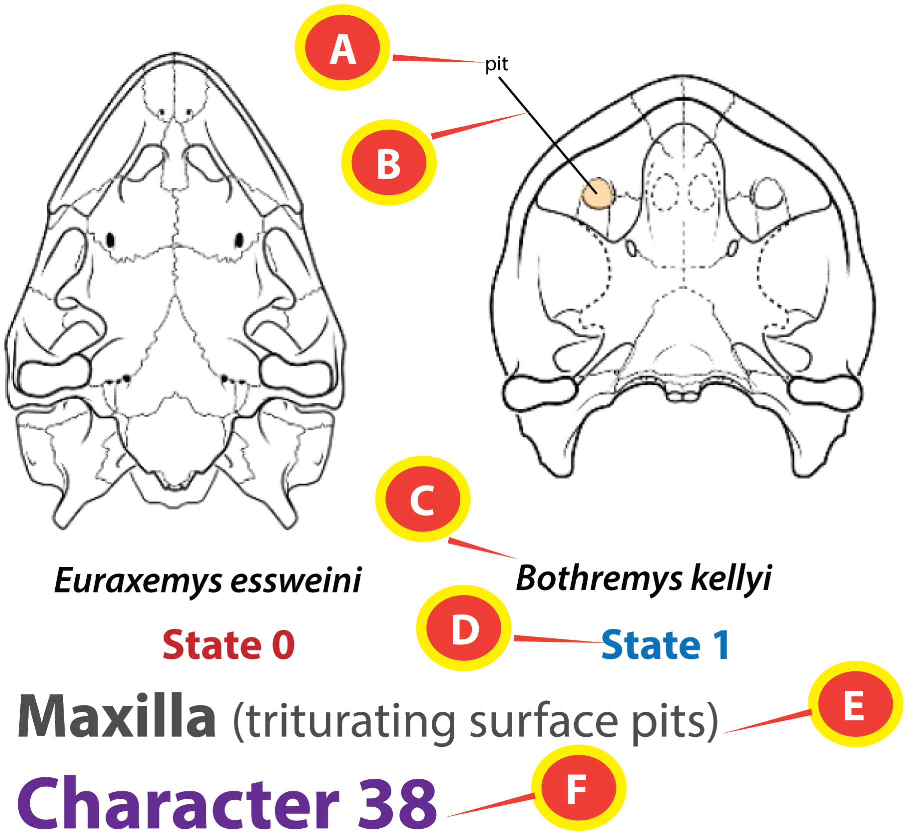

Figure 1. Example of character figure with all the settings used in Adobe Illustrator CS6, page setup: 170 mm width, 225 mm height, .eps file extension. **A.** Bone or feature: Myriad Pro, Regular, 10 pt, black (Color number: 010101). **B.** Line: 1 pt, basic, uniform, black (010101). **C.** Species name: Myriad Pro, Semibold, 18pt, black (010101). **D.** Character state: Myriad Pro, bold, 21 pt, (State 0 (C1282D), State 1 (1071B9), State 2 (F79321), State 3 (24B573), State 4 (93298D), State 5 (ED2279)). **E.** Character name Myriad Pro, bold, 30pt, grey (4D4D4E) and character definition: Myriad Pro, regular, 24pt, grey (4D4D4E). **F.** Character number: Myriad Pro, bold, 36pt, purple (652C90).

References

Bardet N, Jalil N-E, Lapparent de Broin F, Germain D, Lambert O, Amaghzaz M. 2013. A giant chelonioid turtle from the late cretaceous of Morocco with a suction feeding apparatus unique among tetrapods. PLoS ONE **8:**e63586 DOI 10.1371/journal.pone.0063586.

Broin FD. 1977. Contribution à l'étude des Chéloniens. Chéloniens con-tinentaux du Crétacé et du Tertiaire de France. *Mem Mus Nac HistNat* **38:**1-366.

Cadena EA, J. I, Bloch., C. A, Jaramillo. 2010. New podocnemidid turtle (Testudines: Pleurodira) from the Middle-Upper Paleocene of South America. *Journal of Vertebrate Paleontology* **30:**367-382.

Cadena EA, Ksepka DT, Jaramillo CA, and Bloch JI. 2012a. New pelomedusoid turtles (Testudines, Panpleurodira) from the late Palaeocene Cerrejón Formation of Colombia and implications for phylogeny and body size evolution. *Journal of Systematics Palaeontology* **10:**313-331.

Cadena EA, Bloch JI, and Jaramillo CA. 2012b. New bothremydid turtle (Testudines, Pleurodira) from the Paleocene of North-Eastern Colombia. *Journal of Paleontology* **86:**689-699.

Cadena EA, Jaramillo CA, and Bloch JI. 2013. New material of the Platychelyid Turtle *Notoemys zapatocaensis* from the Early Cretaceous of Colombia; Implications for under-standing Pleurodira evolution. In: Brinkman DB, Holroyd PA, and Gardner JD, eds. *Morphology and Evolution of Turtles*: Springer, Dordrecht, 120-140.

Cadena EA, and Joyce WG. 2015. A Review of the Fossil Record of Turtles of the Clades Platychelyidae and Dortokidae. *Bulletin of the Peabody Museum of Natural History* **56**:3-20.

Cadena EA and Parham J. 2015. Oldest known marine turtle? An Early Cretaceous protostegid from Colombia. PaleoBios.

Digimorph, 2015. Available at http://www.digimorph.org2014).

Gaffney ES. 1979. Comparative cranial morphology of recent and fossil turtles. *Bulletin of the American museum of Natural History* **164:**1-313.

Gaffney ES. 1990. The comparative osteology of the Triassic turtle *Proganochelys*. *Bulletin of the American museum of Natural History* **194:**1-263.

Gaffney ES, DeBlieux D, Simons E, Sánchez-Villagra MR, and Meylan P. 2002. Redescription of the skull of *Dacquemys Williams*, 1954, a podocnemidid side-necked turtle from the Late Eocene of Egypt. *American Museum Novitates* **3372**:1-16.

Gaffney ES, Meylan PA, Wood RC, Simons E, and Campos DD. 2011. Evolution of the Side-Necked Turtles: The Family Podocnemididae. *Bulletin of the American museum of Natural History*:1-237.

Gaffney ES, Tong HY, and Meylan PA. 2006. Evolution of the side-necked turtles: The families Bothremydidae, Euraxemydidae, and Araripemydidae. *Bulletin of the American museum of Natural History*:1-237.

Gaffney ES and Wood RC. 2002. *Bairdemys*, a new side-necked turtle (Pelomedusoides: Podocnemididae) from the Miocene of the Caribbean. *American Museum Novitates* **3359**:1-28.

Hirasawa T, Nagashima H, and Kuratani S. 2013. The endoskeletal origin of the turtle carapace. *Nature communications* **4:**2107 doi: 10.1038/ncomms3107

Hirasawa T, Pascual-Anaya J, Kamezaki N, Taniguchi M, Mine K, and Kuratani S. 2014. The Evolutionary Origin of the Turtle Shell and Its Dependence on the Axial Arrest of the Embryonic Rib Cage. *Journal of Experimental Zoology* (Mol Dev Evol) 9999B:1-14.

Hirayama R. 1994. Phylogenetic systematics of chelonioid sea turtles. *The Island Arc* **3:**270-284.

Hirayama R. 1998. Oldest known sea turtle. *Nature* **392**:705-708.

Jiménez-Fuentes E. 1968. *Stereogenys salmanticensis* nov. sp., quelonioeocénico del Valle del Duero. *Studia Geologica Salmantensia* **24**:191-203.

Jiménez-Fuentes E. 1992. Quelonios fósiles de Castilla y Leon. In: Jiménez-Fuentes E, and Llovera C, eds. Vertebrados fósiles de Castilla y León, Salamanca: Junta de Castilla y León, 177-195.

Joyce WG. 2007. Phylogenetic relationships of Mesozoic Turtles. *Bulletin of Peabody Museum of Natural History* **48:**3-102.

Joyce WG, chapman SD, Moody RT, and Walker CA. 2011. The skull of the solemydid turtle *Helochelydra nopcsai* from the Early Cretaceous of the Isle of Wight (UK) and a review of Solemydidae. *Palaeontology* **212:**75-97.

Kear BP, and Lee MSY. 2006. A primitive protostegid from Australia and early sea turtle evolution. *Biology Letters* **2:**116-119.

Lapparent de Broin F. 2000. The oldest pre-Podocnemidid turtle (Chelonii, Pleurodira), from the Early Cretaceous, Ceara State, Brasil, and its environment. *Threeballs del Museu of Geologia de Barcelona* **9**:43-95.

Lapparent de Broin F, Bardet N, Amaghzaz M, and Meslouh S. 2014. A strange new chelonioid turtle from the Latest CretaceousPhosphates of Morocco. *Comptes Rendus Palevol* **86**:75-97

Lapparent de Broin F, and Wermer C. 1998. New late Cretaceous turtles from the Western Desert, Egypt. *Annales de Paléontologie* **84:**131-214.

Lyson TR, Schachner ER, Botha-Brink J, Scheyer TM, Lambertz M, Bever GS, Rubidge BS, and de Queiroz K. 2014. Origin of the unique ventilatory apparatus of turtles. *Nature communications*.

Meylan PA. 1996. Skeletal morphology and relationships of the Early Cretaceous side-necked turtle, Araripemys barretoi (Testudines: Pelomedusoides: Araripemydidae), from the Santana Formation of Brazil. *Journal of Vertebrate Paleontology* **16**:20-33.

Parham JF, and Pyenson ND. 2010. New sea turtle from the Miocene of Peru and the iterative evolution of feeding ecomorphologies since the Cretaceous. *Journal of Vertebrate Paleontology* **84**:231-247.

Pérez-García A, and Lapparent de Broin F. 2013. A new species of *Neochelys* (Chelonii, Podocnemididae) from the Ypresian (Early Eocene) of the South of France. *Comptes Rendus Palevol* **12:**269-277.

Pritchard PCH. 2008. Evolution and structure of the Turtle shell. In: Wyneken J, Godfrey MH, and Bels V, eds. *Biology of Turtles*. Boca Raton: CRC Press, 45-85.

Rabi M, Zhou C-F, Wings O, Ge S, and Joyce WG. 2013. A new xinjiangchelyid turtle from the Middle Jurassic of Xinjiang, China and the evolution of the basipterygoid process in Mesozoic turtles. *BMC Evolutionary Biology* **13**:203-221.

Romano PSR, Gallo V, Renato RC, and Antonioli L. 2014. *Atolchelys lepida*, a new side-necked turtle from the Early Cretaceous of Brazil and the age of crown Pleurodira. *Biology Letters* 10:20140290. http://dx.doi.org/10.1098/rsbl.2014.0290

Sereno PC, and ElSahfie SJ. 2013. A New Long-Necked Turtle, *Laganemys tenerensis* (Pleurodira: Araripemydidae), from the Elrhaz Formation (Aptian–Albian) of Niger. In: Brinkman DB, Holroyd PA, and Gardner JD, eds. Morphology and evolution of turtles. Netherlands: Springer, 215-250.

Sterli J. 2008. A new, nearly complete stem turtle from the Jurassic of South America with implications for turtle evolution. *Biology Letters* **4:**286-289.

Sterli J, and de la Fuente MS. 2013. New evidence from the Palaeocene of Patagonia (Argentina) on the evolution and palaeo-biogeography of Meiolaniformes (Testudinata, new taxon name). *Journal of Systematic Palaeontology* **11:**835-852.

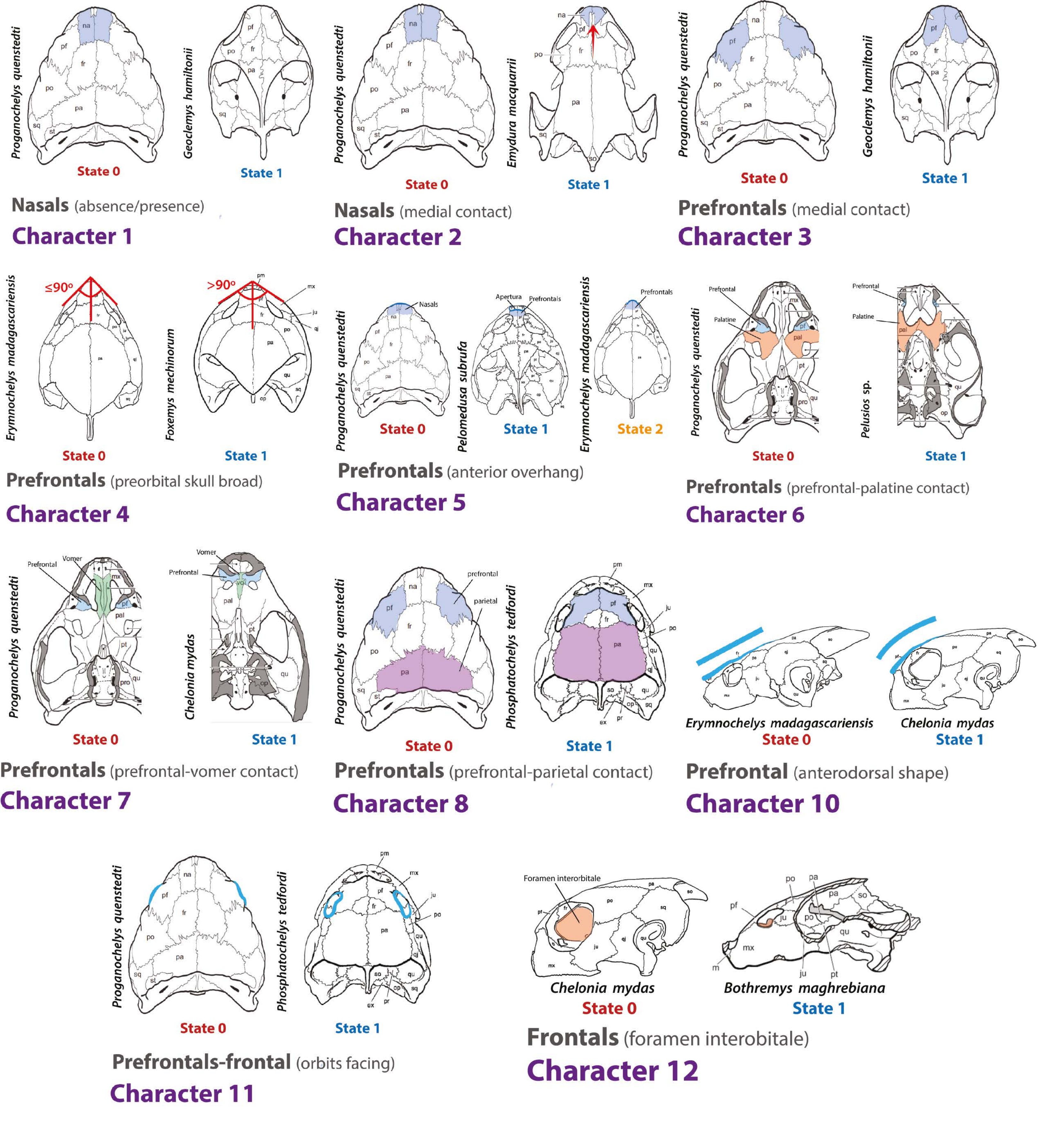

Plate 1. Characters 1 to 12

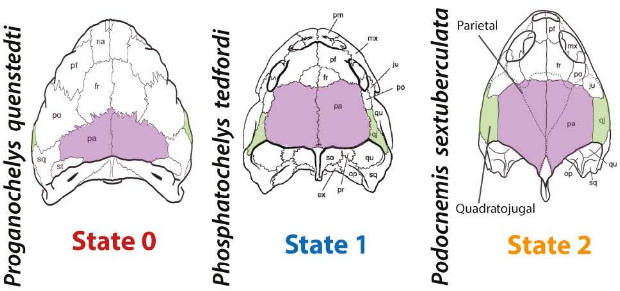

Parietals (parietal-quadratojugal contact)

Character 13

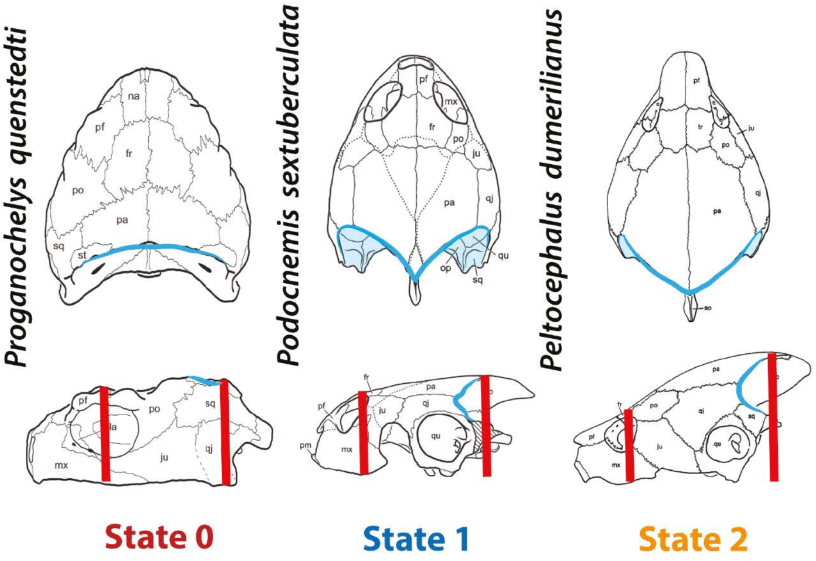

Parietals (temporal emargination)

Character 14

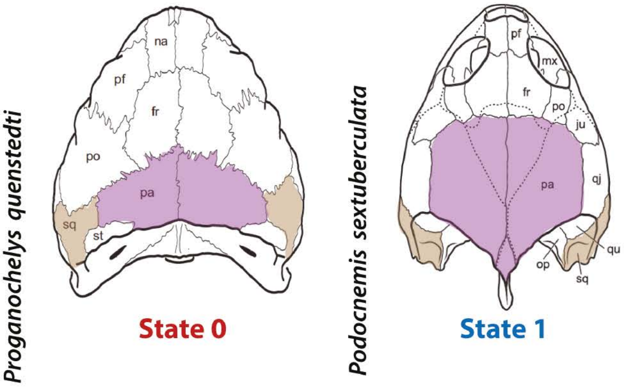

Parietals (parietal-squamosal contact)

Character 15

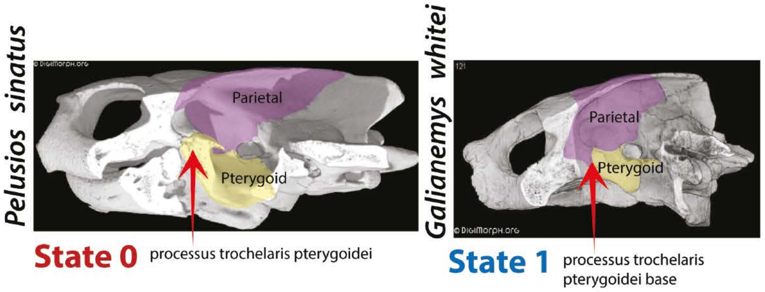

Parietals (parietal-pterygoid contact)

Character 16

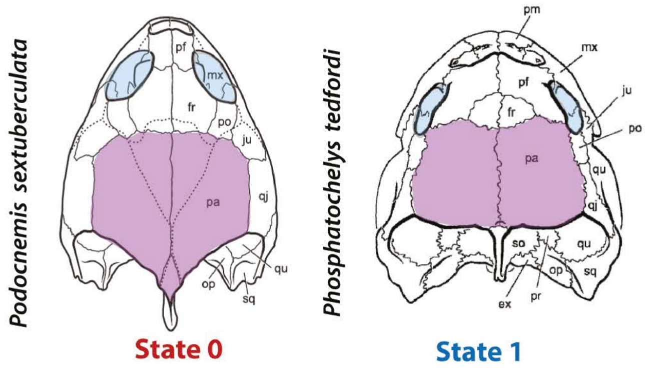

Parietals (parietals enters orbital margin)

Character 18

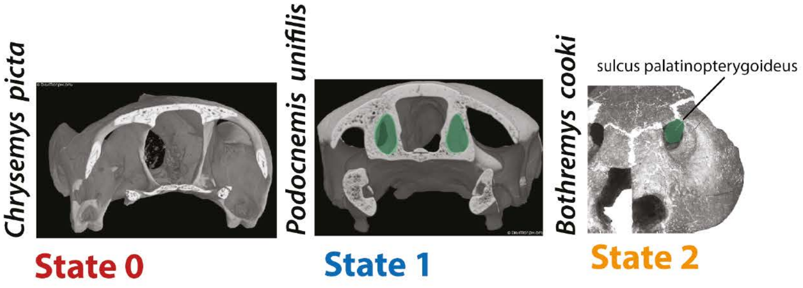

Parietals (sulcus palatinopterygoideus)

Character 17

Plate 2. Characters 13 to 18

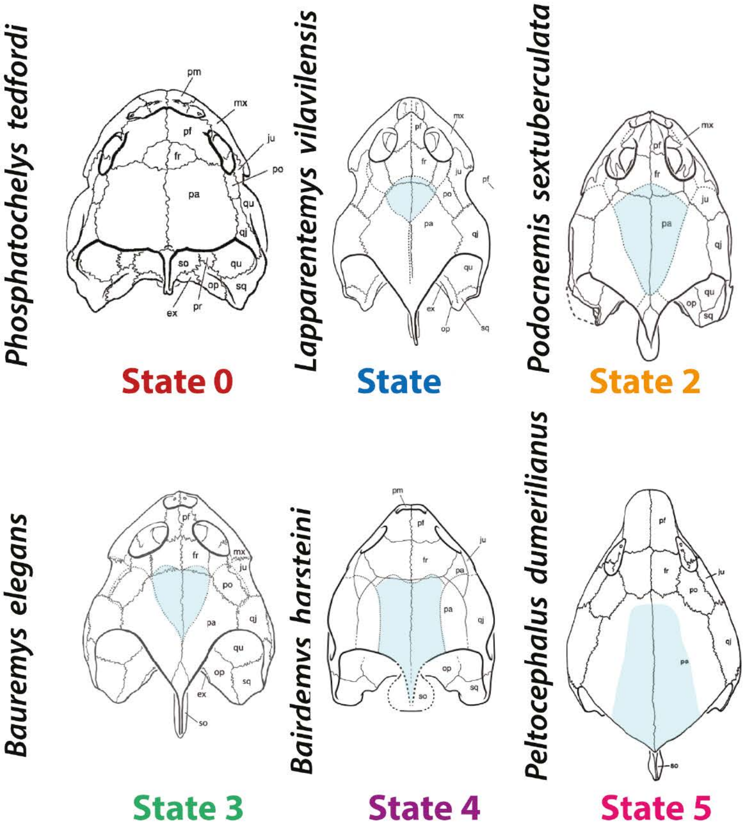

Parietals (interparietal scute shape)

Character 19

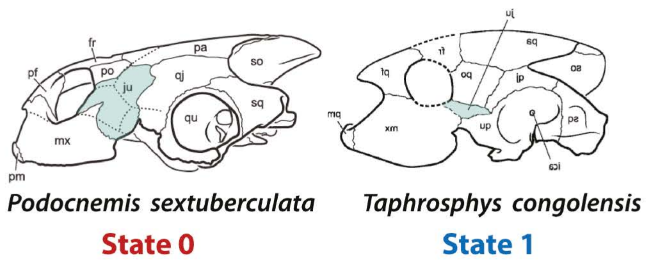

Jugals (jugal narrow dorsoventrally)

Character 22

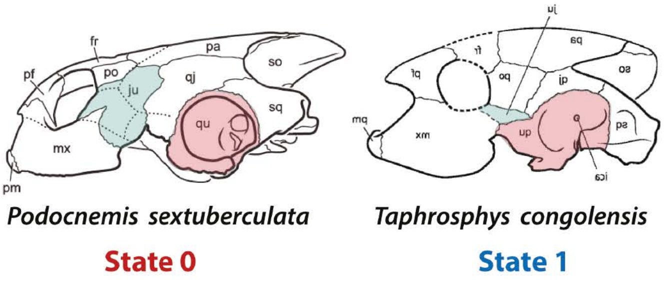

Jugals (jugal-quadrate contact)

Character 23

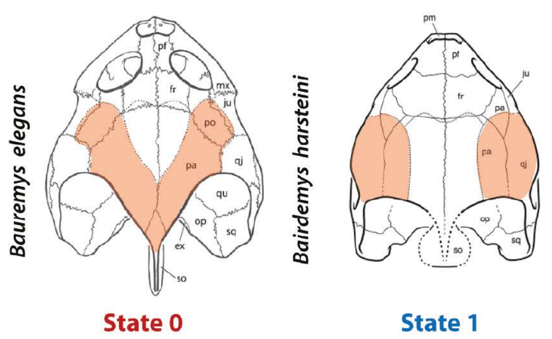

Parietals (parietal scutes contact)

Character 20

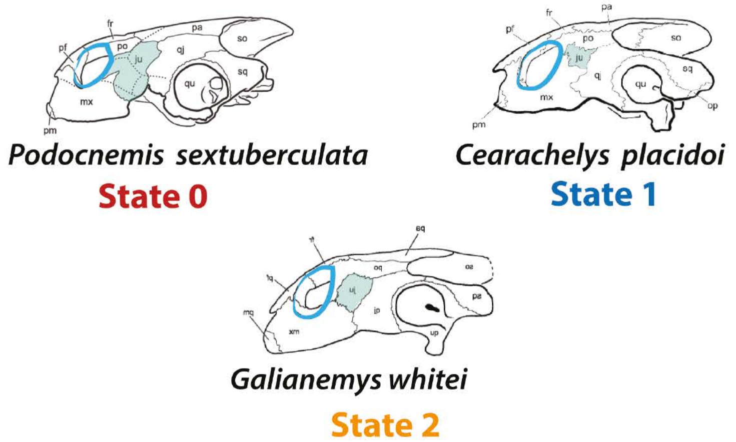

Jugals (jugals-orbital margin contact)

Character 21

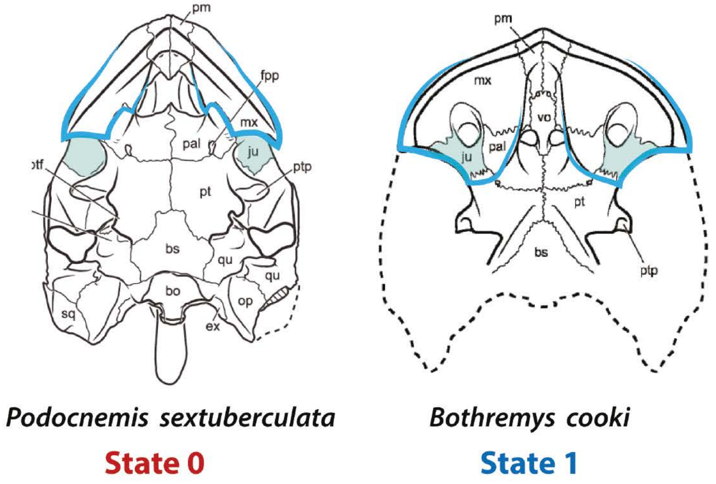

Jugals (exposure on triturating surface)

Character 24

Plate 3. Characters 19 to 24

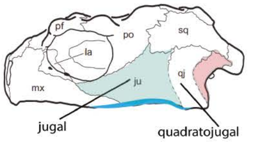

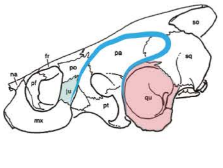

Proganochelys quenstedti — State 0

Emydura macquarrii — State 1

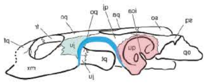

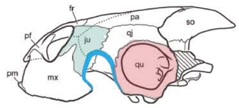

Araripemys barretoi — State 2

Podocnemis sextuberculata — State 3

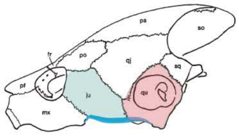

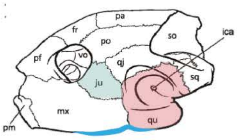

Erymnochelys madagascariensis — State 4

Azabbaremys moragjonesi — State 5

Jugals (cheek emargination)

Character 25

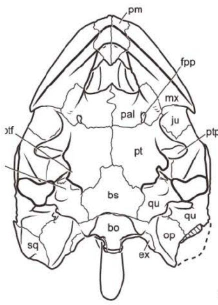

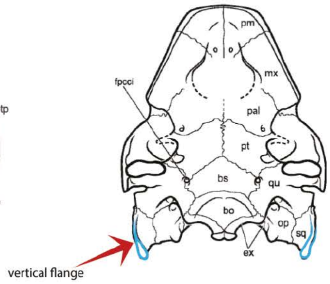

Podocnemis sextuberculata — State 0

Taphrophys ippolitoi — State 1

Squamosal (vertical flange)

Character 28

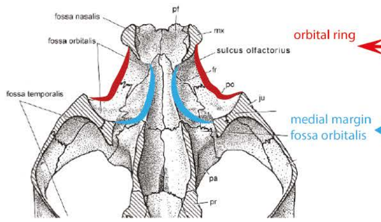

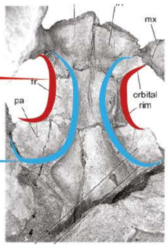

Pelusios castaneus — State 0

Bothremys cooki — State 1

Postorbital (fossa postorbitalis post. enlarg.)

Character 30

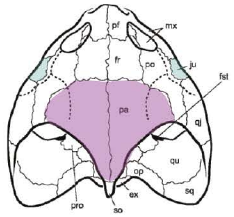

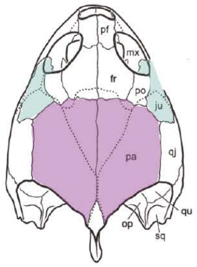

Galianemys emringeri — State 0

Podocnemis sextuberculata — State 1

Jugals (jugal-parietal contact)

Character 26

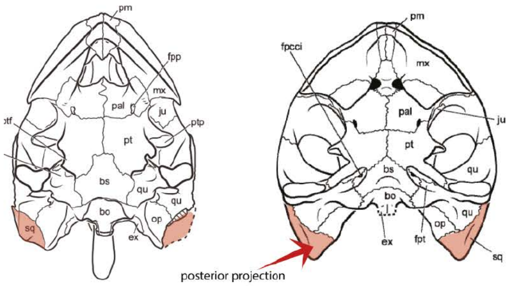

Podocnemis sextuberculata — State 0

Foxemys mechinorum — State 1

Squamosal (posterior projection)

Character 27

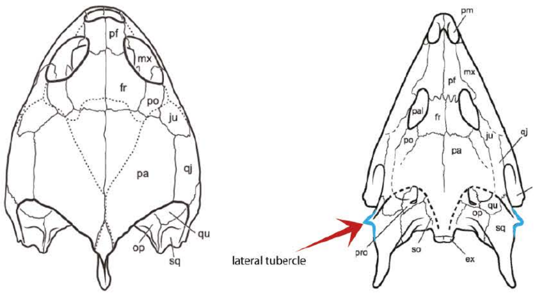

Podocnemis sextuberculata — State 0

Labrostochelys galkini — State 1

Squamosal (lateral tubercle)

Character 29

Plate 4. Characters 25 to 30

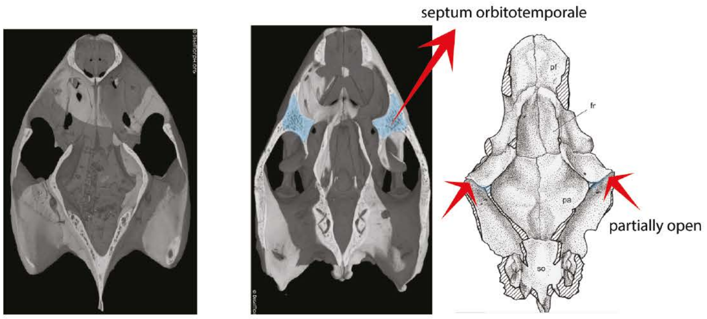

Chrysemys picta *Podocnemis unifilis* *Taphrosphys sulcatus*

State 0 State 1 State 2

Postorbital (septum orbitotemporale)

Character 31

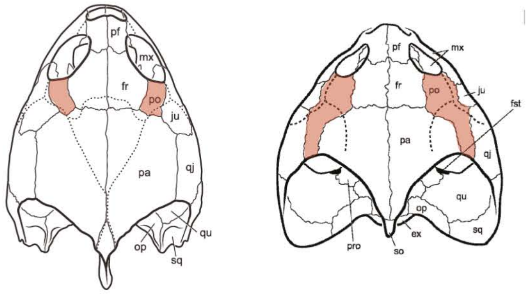

Podocnemis sextuberculata *Galianemys emringeri*

State 0 State 1

Postorbital (length vs width)

Character 32

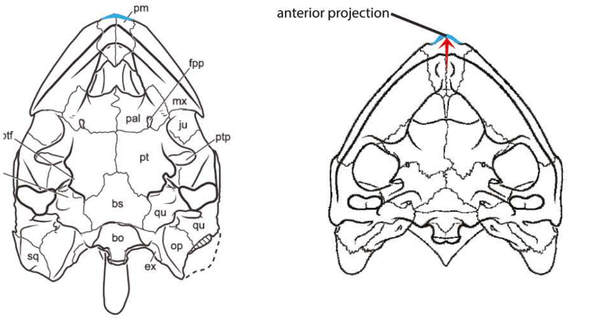

Podocnemis sextuberculata *Azabbaremys moragjonesi*

State 0 State 1

Premaxilla (anterior projection)

Character 33

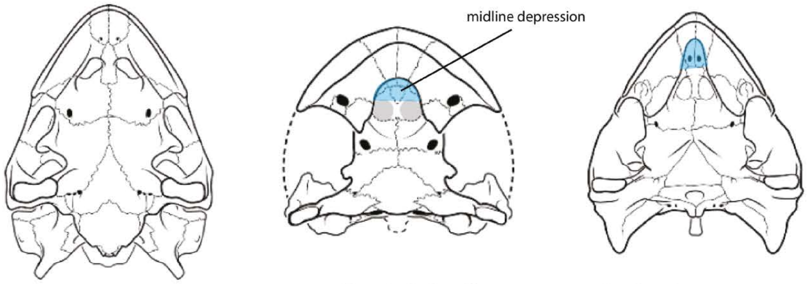

Euraxemys essweini *Zolhafah bella* *Araiochelys hirayamai*

State 0 State 1 State 2

Premaxilla (midline depression)

Character 34

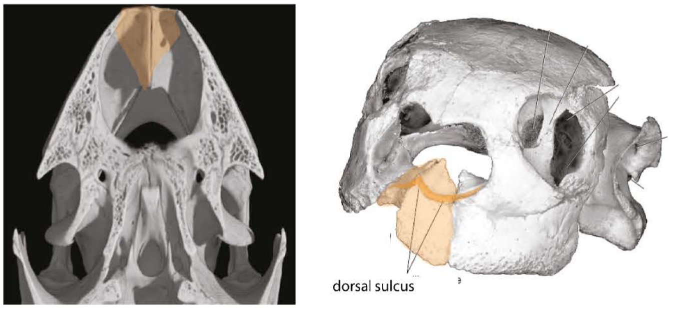

Podocnemis unifilis *Phosphatochelys tedfordi*

State 0 State 1

Premaxilla (dorsal sulcus)

Character 35

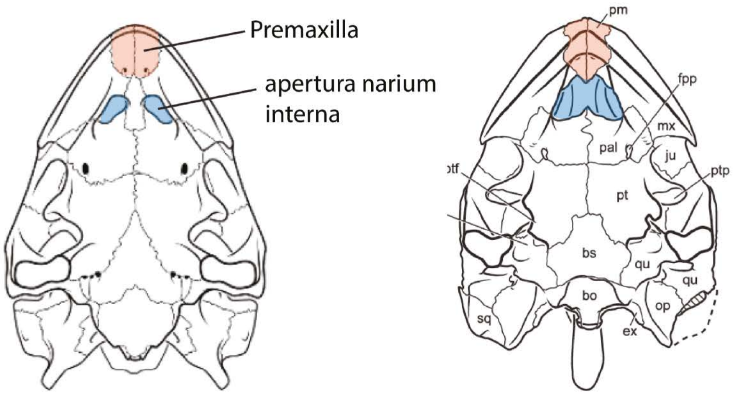

Euraxemys essweini *Podocnemis unifilis*

State 0 State 1

Premaxilla (apertura narium interna)

Character 36

Plate 5. Characters 31 to 36

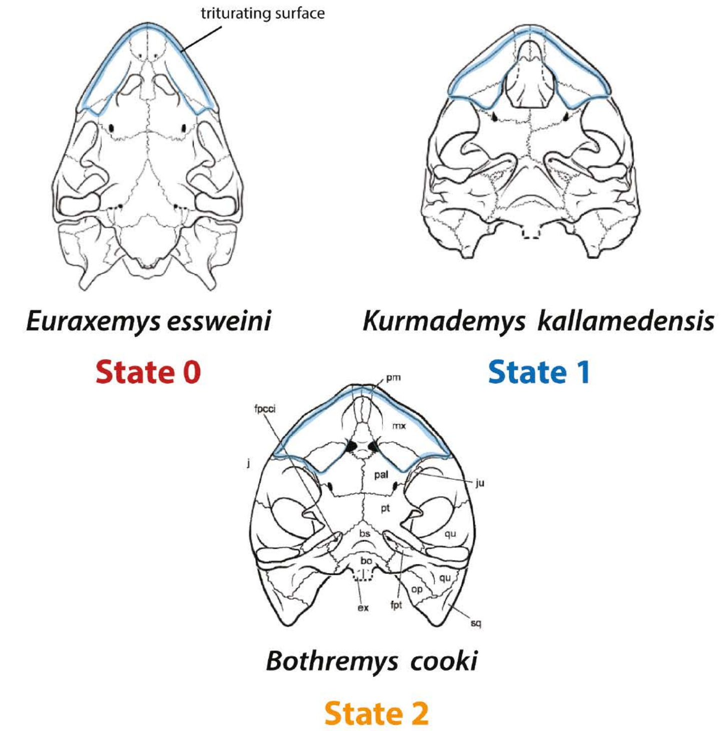

Maxilla (triturating surface)

Character 37

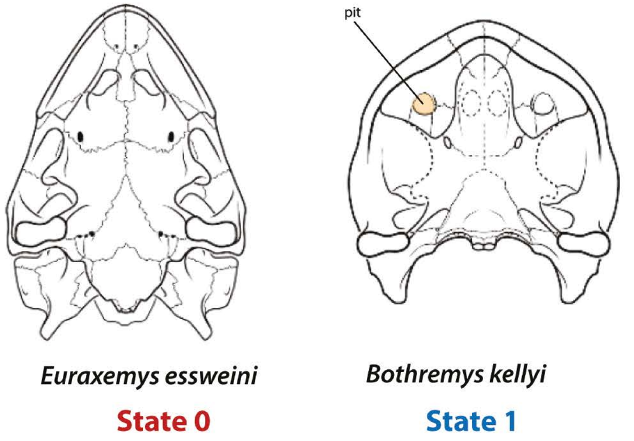

Maxilla (triturating surface pits)

Character 38

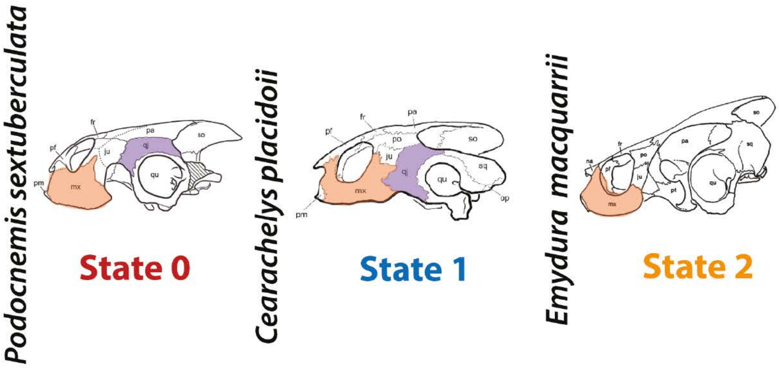

Maxilla (maxilla-quadratojugal contact)

Character 40

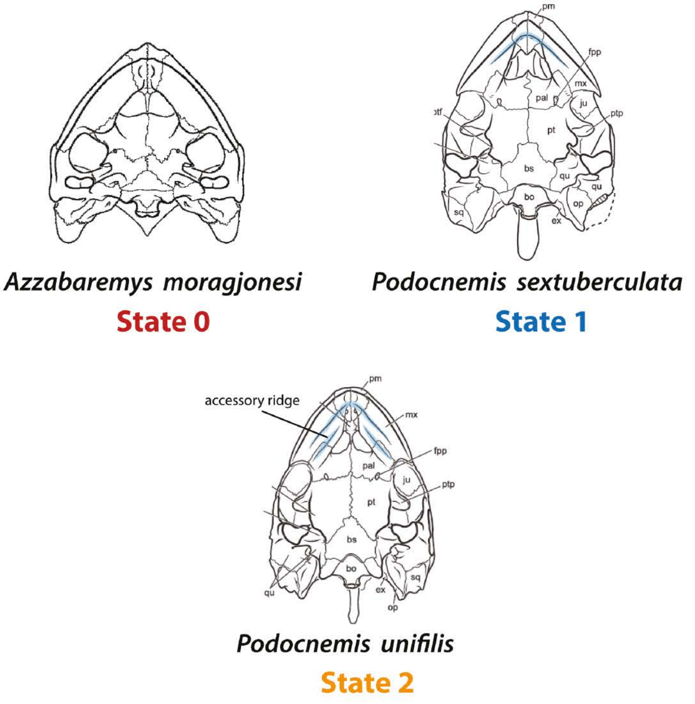

Maxilla (accessory ridge(s))

Character 39

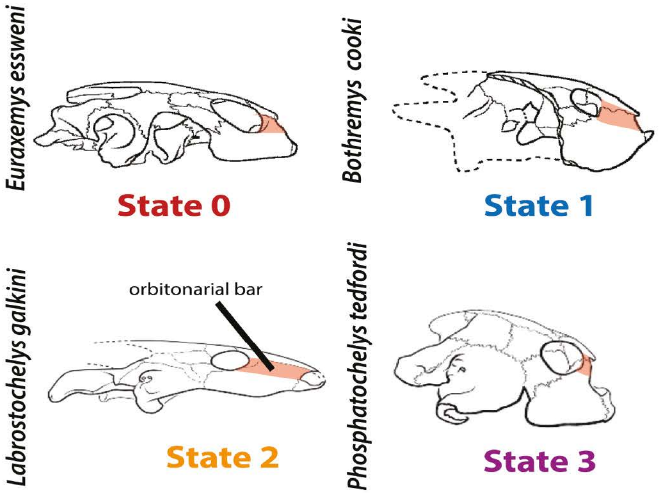

Maxilla (orbitonarial bar)

Character 41

Plate 6. Characters 37 to 41

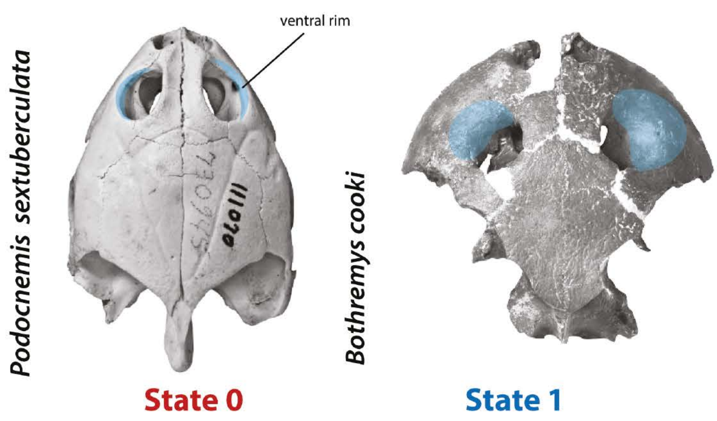

Maxilla (ventral rim of orbit)

Character 42

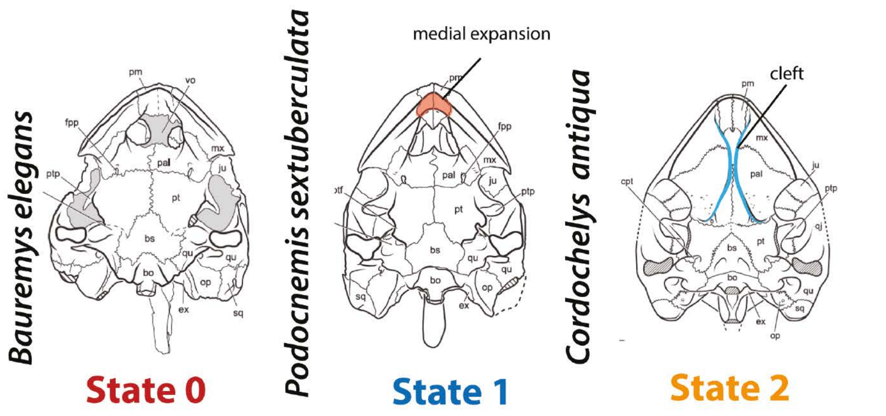

Maxilla (medial expansion of trit. surface)

Character 43

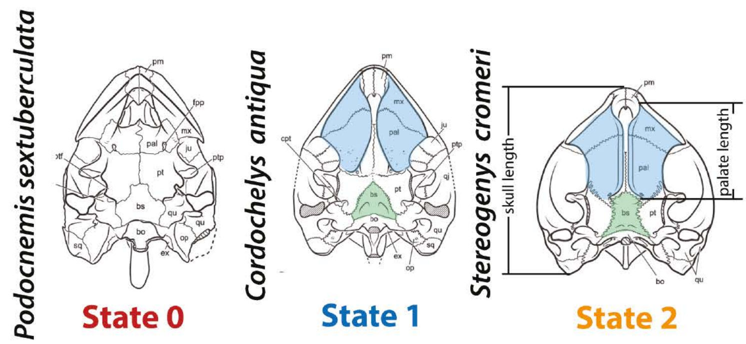

Maxilla-palatine (secondary pl. length)

Character 44

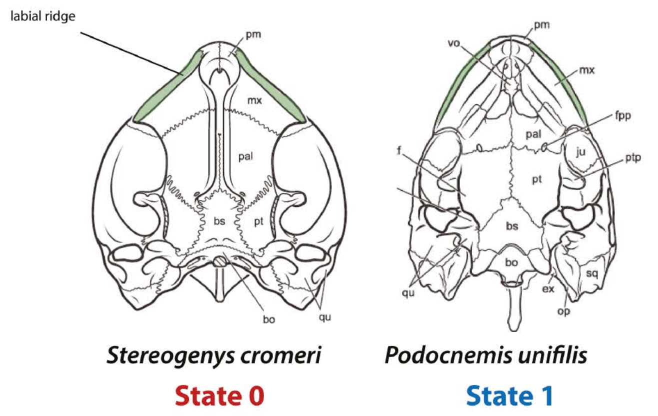

Maxilla (labial ridge height and width)

Character 45

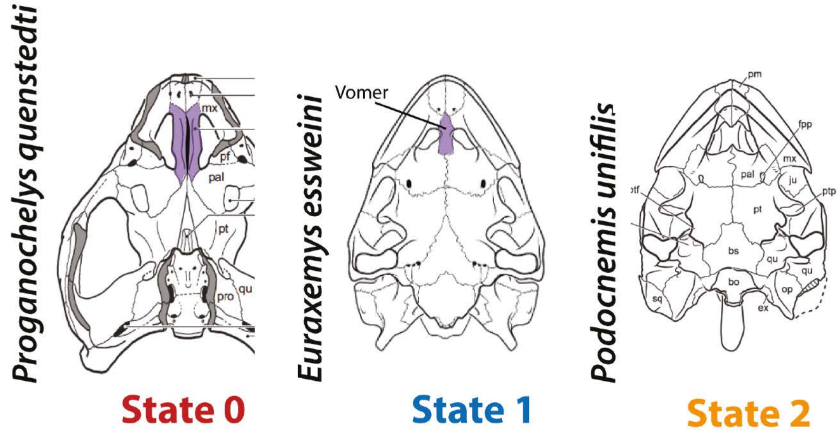

Vomer (number, presence/absence)

Character 46

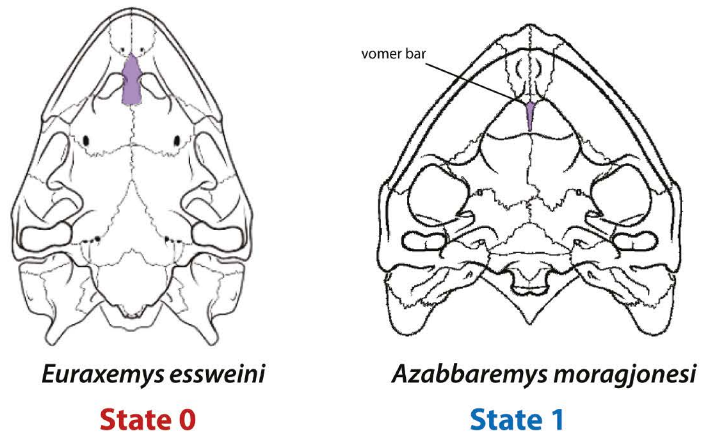

Vomer (central bar contacts)

Character 47

Plate 7. Characters 42 to 47

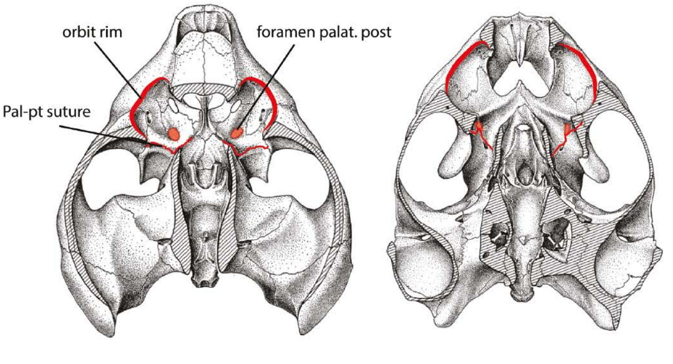

Eubaena cephalica — **State 0**

Pelusios sp. — **State 1**

Palatine (foramen palatinum posterius)

Character 48

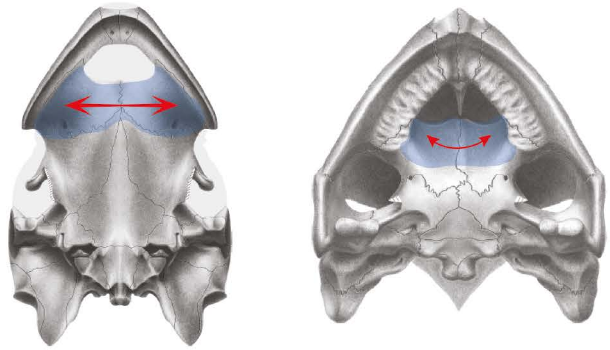

Araripemys barretoi — **State 0**

Azabbaremys moragjonesi — **State 1**

Palatine (dorsally arched palate)

Character 49

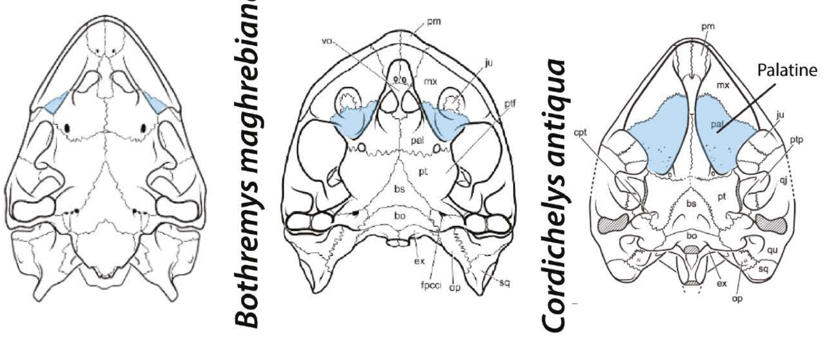

Euraxemys essweini — **State 0**

Bothremys maghrebiana — **State 1**

Cordichelys antiqua — **State 2**

Palatine (upper triturating sf. contribution)

Character 50

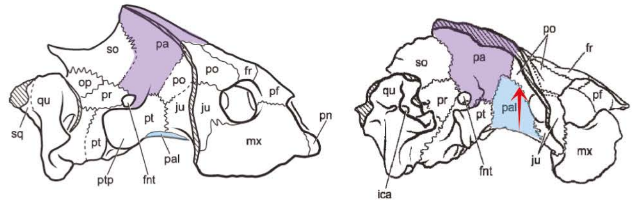

Lemurchelys diasphax — **State 0**

Stereogenys cromeri — **State 1**

Palatine (dorsal process contact parietal)

Character 52

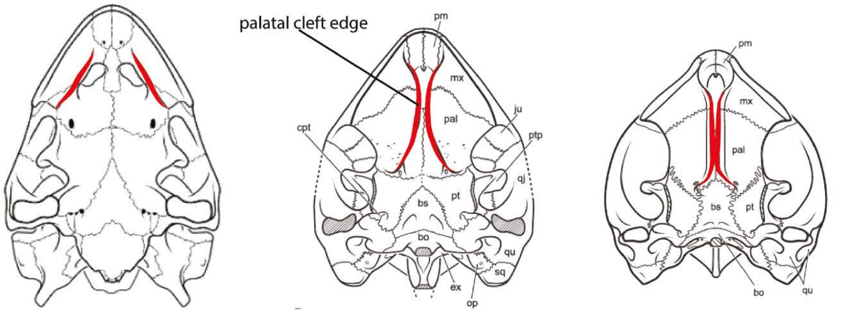

Euraxemys essweini — **State 0**

Cordichelys antiqua — **State 1**

Stereogenys cromeri — **State 2**

Palatine (medial edges of palatal cleft)

Character 51

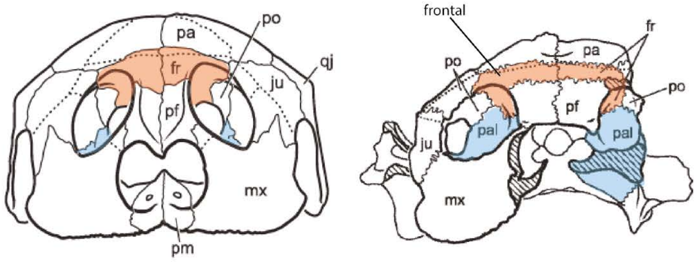

Podocnemis sextuberculata — **State 0**

Stereogenys cromeri — **State 1**

Palatine (dorsal process contacts frontal)

Character 53

Plate 8. Characters 48 to 53

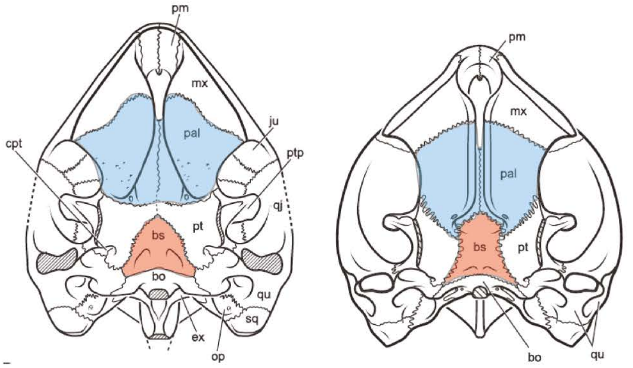

Cordichelys antiqua
State 0

Stereogenys cromeri
State 1

Palatine (palatine-basisphenoid contact)

Character 54

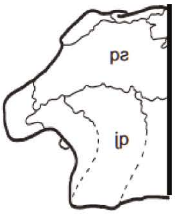

Proganochelys quenstedti
State 0

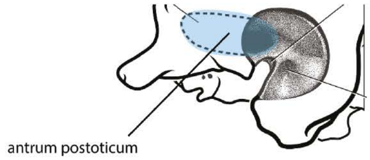

Euraxemys essweini
State 2

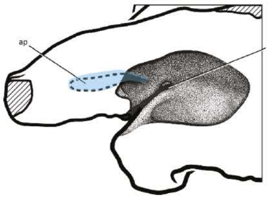

Foxemys mechinorum
State 1

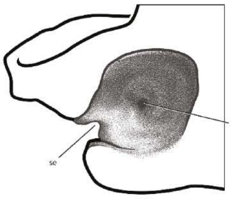

Azabbaremys moragjonesi
State 3

Quadrate (antrum postoticum)

Character 55

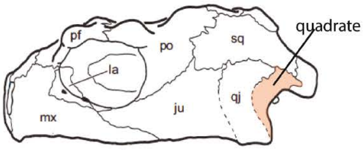

Proganochelys quenstedti
State 0

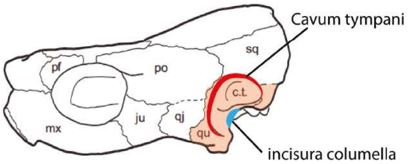

Kayentachelys aprix
State 1

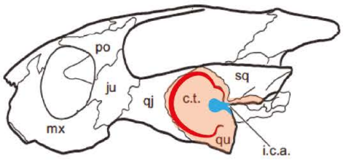

Geoclemmys hamiltonii
State 2

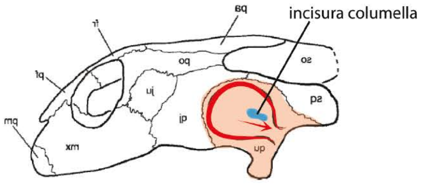

Galianemys whitei
State 3

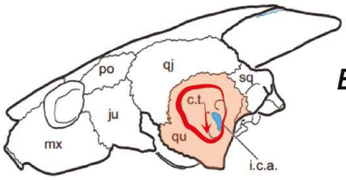

Erymnochelys madagascariensis
State 4

Quadrate (cavum tympani)

Character 56

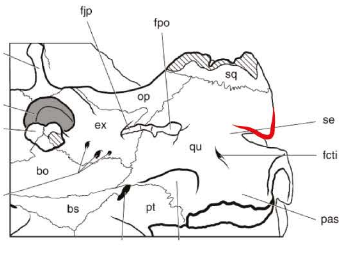

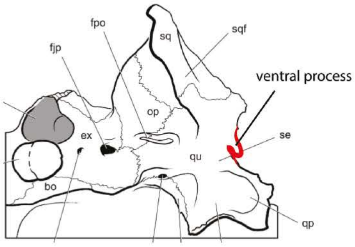

Galianemys whitei
State 0

Labrostochelys galkini
State 1

Quadrate (ventral process of s. Eustachi)

Character 57

Plate 9. Characters 54 to 57

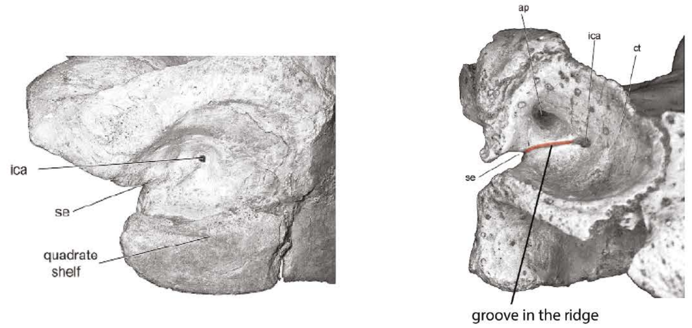

Azabbaremys moragjonesi — **State 0**

Galianemys whitei — **State 1**

Quadrate (groove on incisura columella)

Character 58

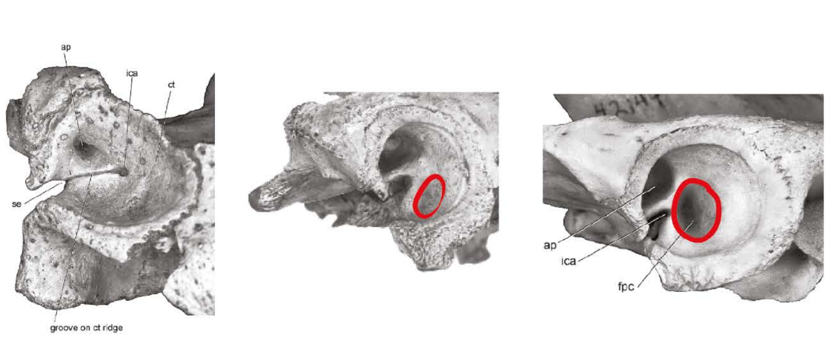

Galianemys whitei — **State 0**

Euraxemys essweni — **State 1**

Pelusios sinatus — **State 2**

Quadrate (fossa precolumellaris)

Character 59

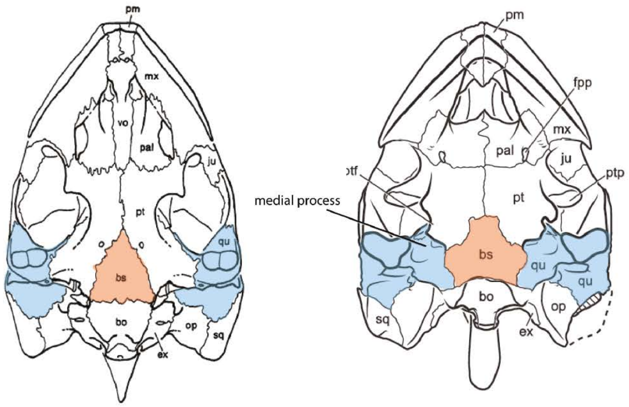

Emys orbicularis — **State 0**

Podocnemis sextuberculata — **State 1**

Quadrate (medial process)

Character 60

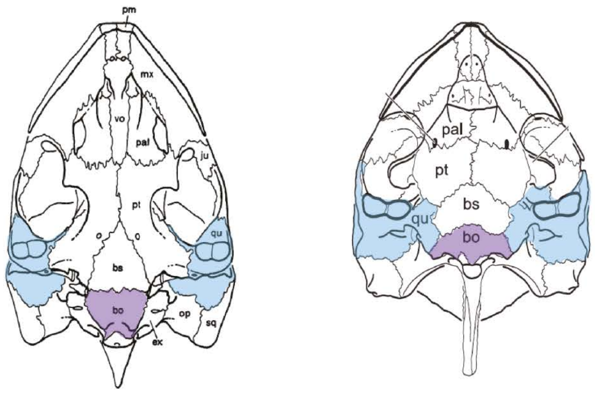

Emys orbicularis — **State 0**

Erymnochelys madagascariensis — **State 1**

Quadrate (quadrate-basioccipital contact)

Character 61

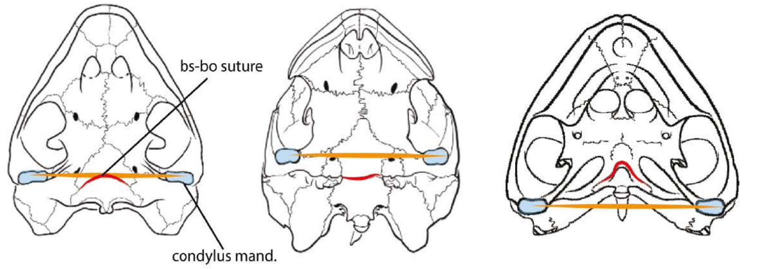

Galianemys whitei — **State 0**

Pelomesuda subrufa — **State 1**

Nigeremys gigantea — **State 2**

Quadrate (condylus mandibularis position)

Character 62

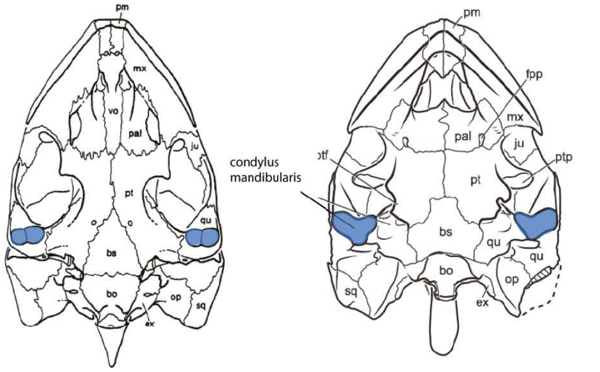

Emys orbicularis — **State 0**

Podocnemis sextuberculata — **State 1**

Quadrate (condylus mandibularis shape)

Character 63

Plate 10. Characters 58 to 63

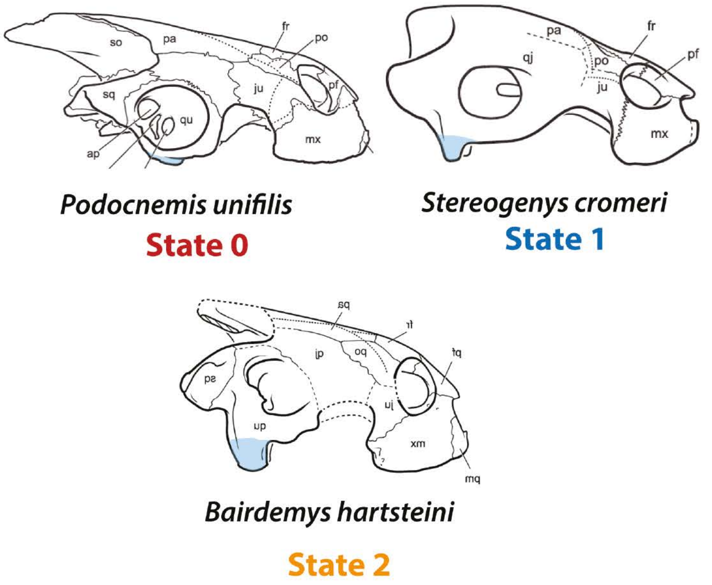

Quadrate (ventral process)

Character 64

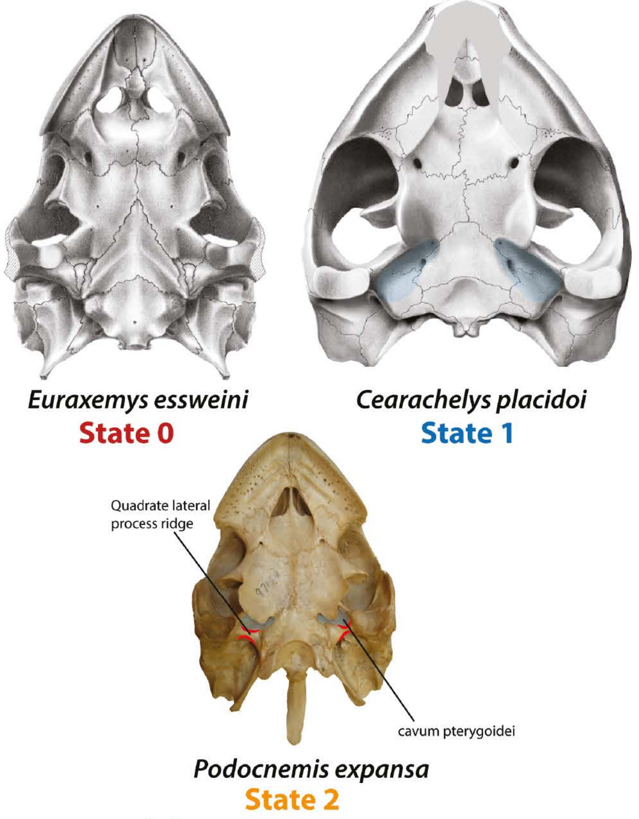

Pterygoid (cavum pterygoidei)

Character 65

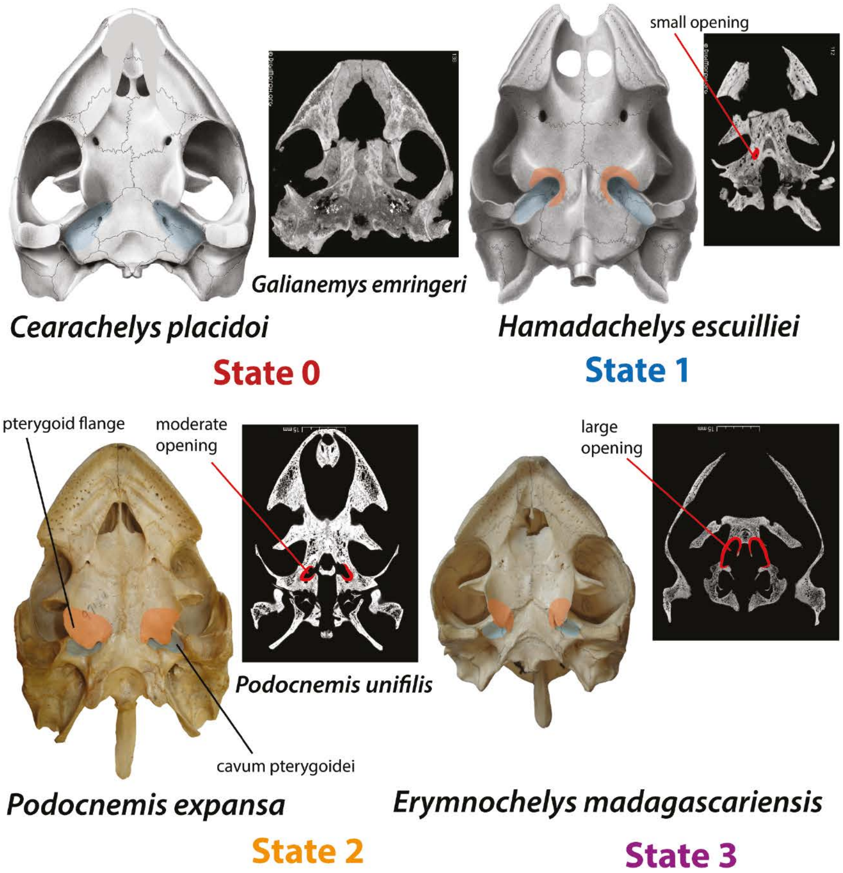

Pterygoid (flooring and anterior opening)

Character 66

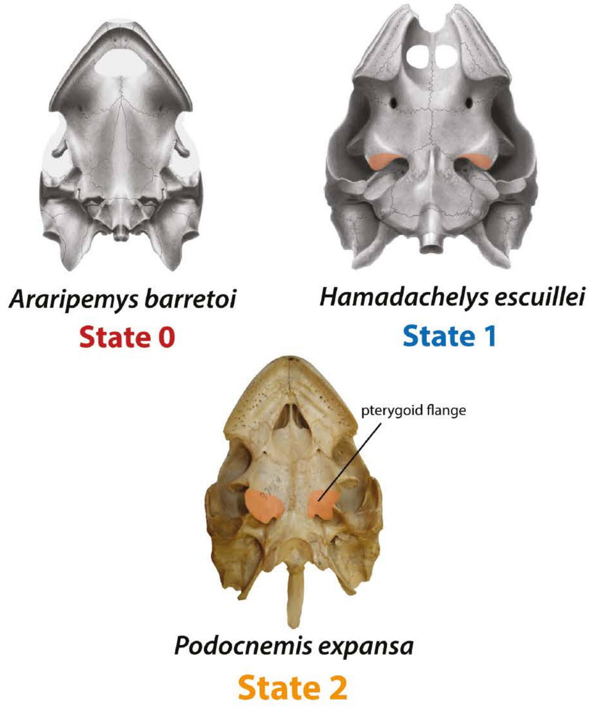

Pterygoid (pterygoid flange)

Character 67

Plate 11. Characters 64 to 67

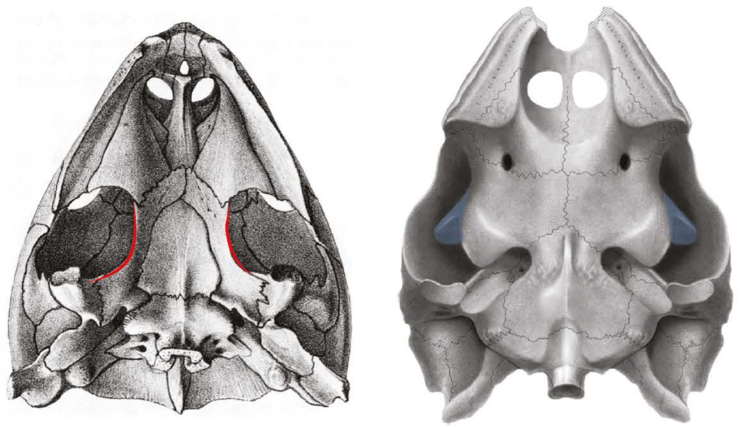

Dermochelys coriacea — State 0

Hamadachelys escuilliei — State 1

Pterygoid (proc. trochlearis pterygoidei)

Character 68

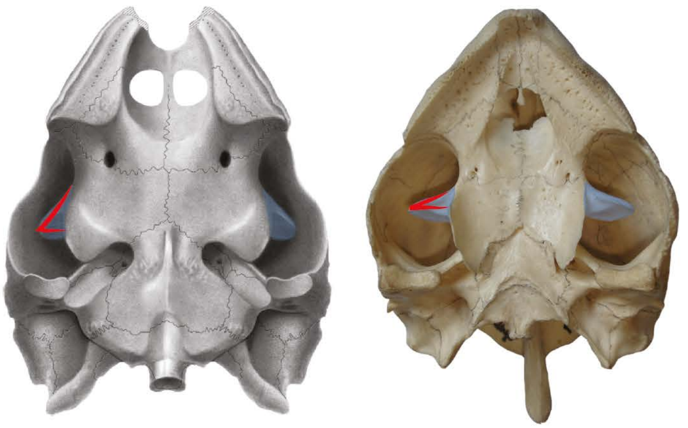

Hamadachelys escuilliei — State 0

Erymnochelys madagascariensis — State 1

Pterygoid (proc. trochlearis lateral margin)

Character 69

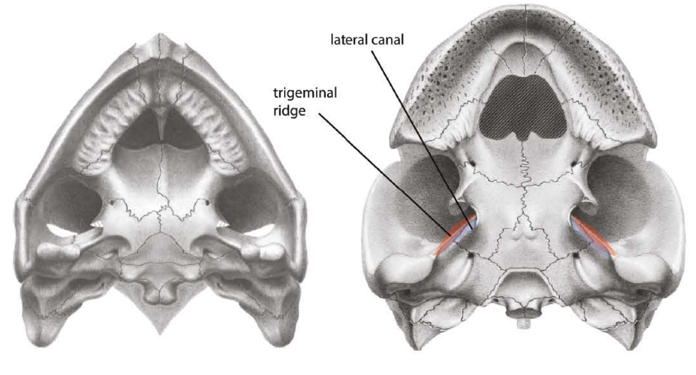

Azabbaremys moragjonesi — State 0

Phosphatochelys tedfordi — State 1

Pterygoid (trigeminal ridge and lat. canal)

Character 70

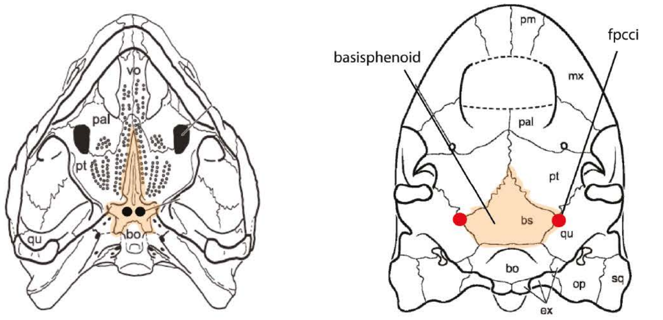

Proganochelys quenstedti — State 0

Taphrosphys congolensis — State 1

Pterygoid (for. post. can. cart. intr. (fpcci))

Character 71

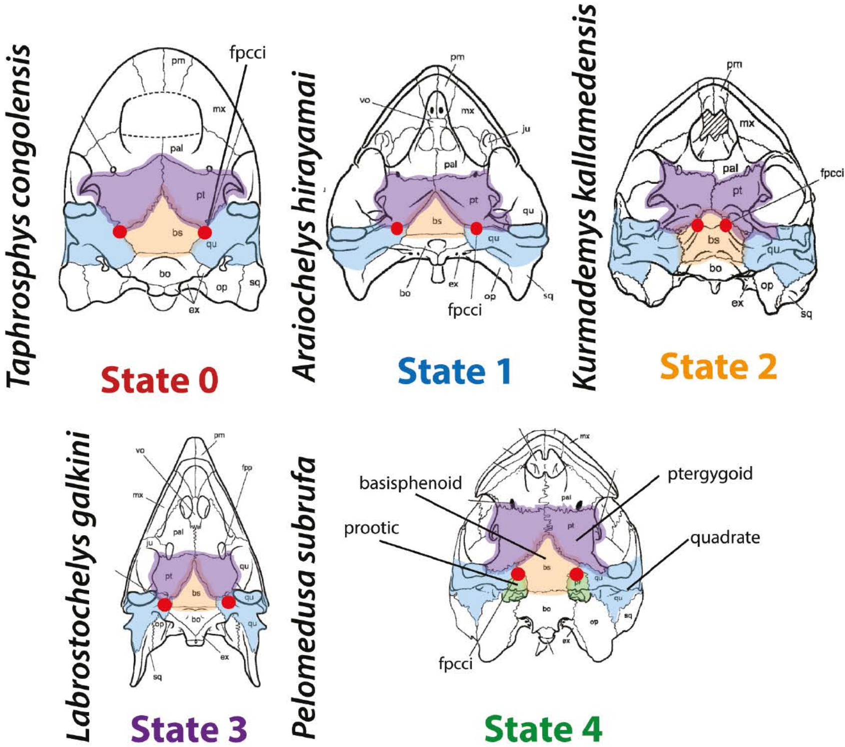

Pterygoid (fpcci position)

Character 72

Plate 12. Characters 68 to 72

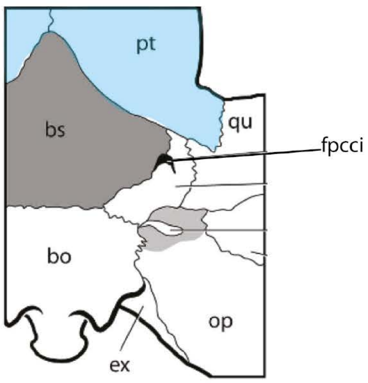

Pelusios sinatus
State 0

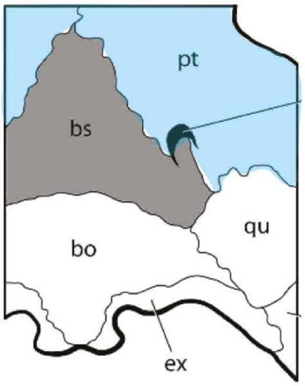

Galianemys whitei
State 1

Pterygoid (posterior margin and fpcci)

Character 73

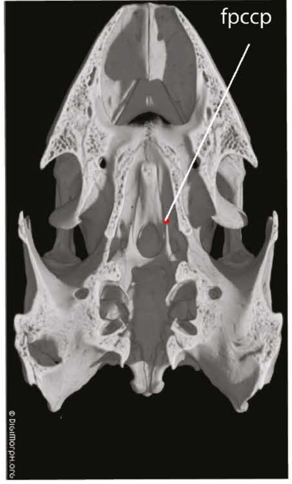

Podocnemis unifilis
State 0

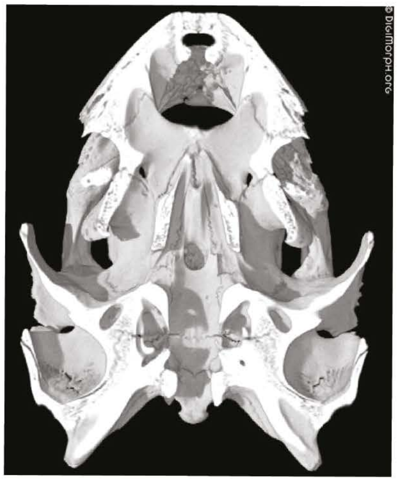

Pelusios sinatus
State 1

Pterygoid (for. post. can. car. pal. (fpccp))

Character 74

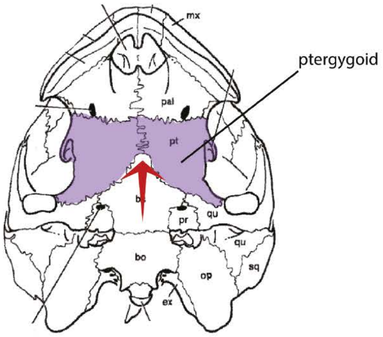

Pelomedusa subrufa
State 0

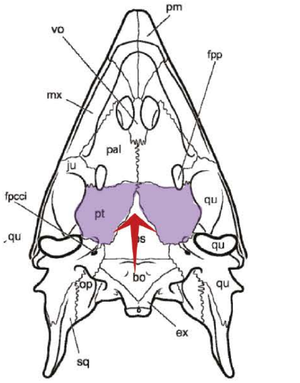

Labrostochelys galkini
State 1

Pterygoids (midline contact pterygoids)

Character 75

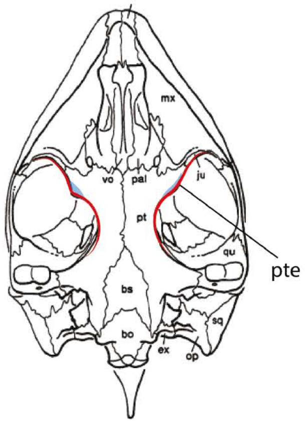

Kinosternon subrubrum
State 0

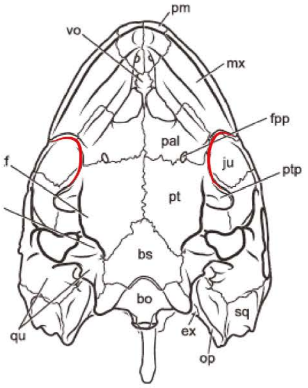

Podocnemis unifilis
State 1

Pterygoid (proc. pterygoideus ext. (pte))

Character 76

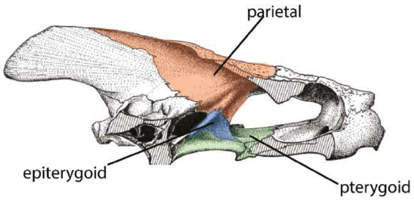

Chelydra serpentina
State 0

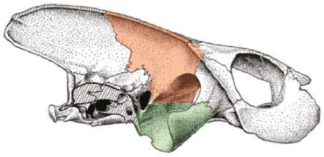

Emydura sp.
State 1

Epipterygoid (presence/absence)

Character 77

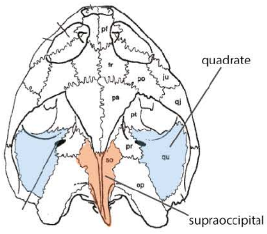

Pelomesuda subrufa
State 0

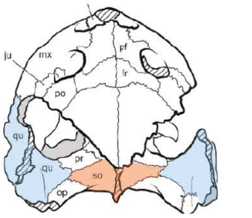

Bothremys maghrebiana
State 1

Supraoccipital (supraoccipital-quadrate)

Character 78

Plate 13. Characters 73 to 78

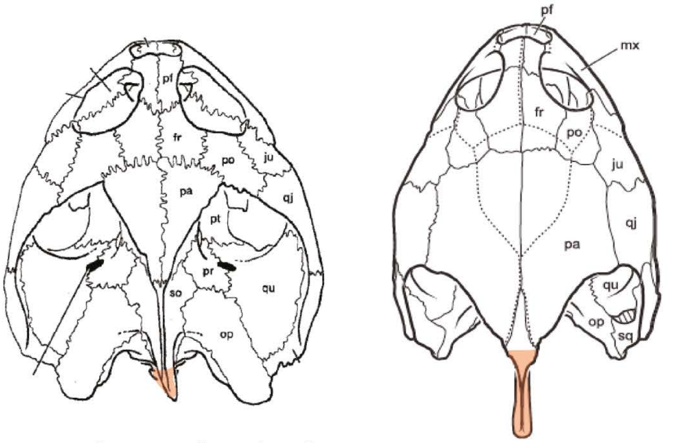

Pelomesuda subrufa
State 0

Podocnemis unifilis
State 1

Supraoccipital (crista suproccp. pres/abs)

Character 79

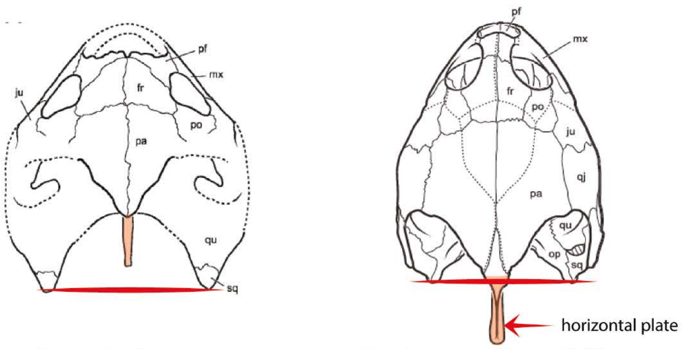

Arenila krebsi
State 0

Podocnemis unifilis
State 1

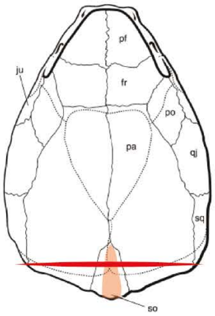

Dacquemis paleomorpha
State 2

Supraoccipital (cris. sup. horizontal plate)

Character 80

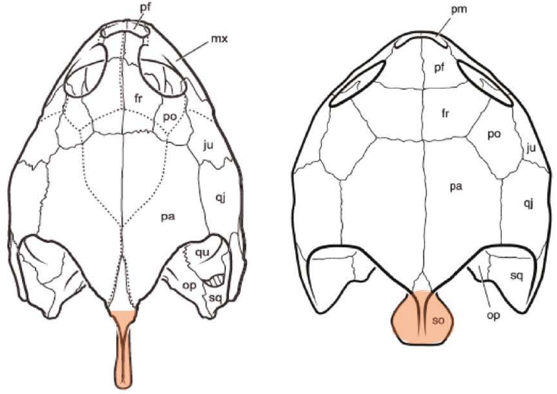

Podocnemis unifilis
State 0

Bairdemys venezuelensis
State 1

Supraoccipital (cris. sup. lat. expans)

Character 81

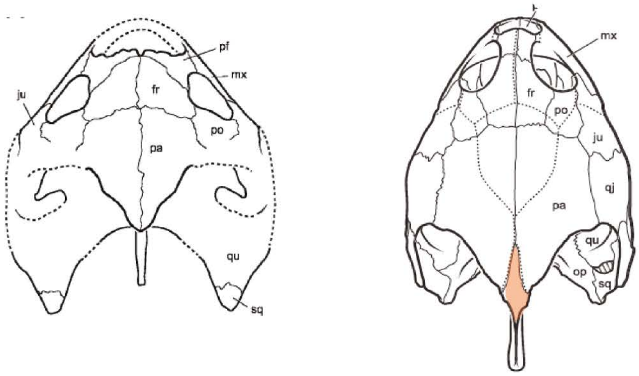

Arenila krebsi
State 0

Podocnemis unifilis
State 1

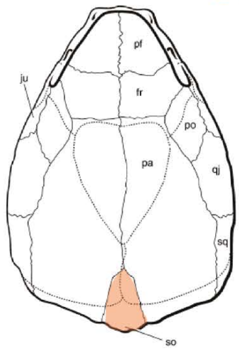

Dacquemis paleomorpha
State 2

Supraoccipital (dorsal exposure)

Character 82

Plate 14. Characters 79 to 82

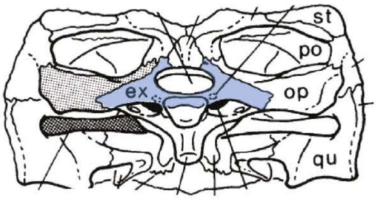

Proganochelys quenstedti
State 0

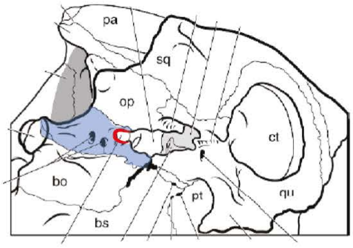

Euraxemys essweni
State 1

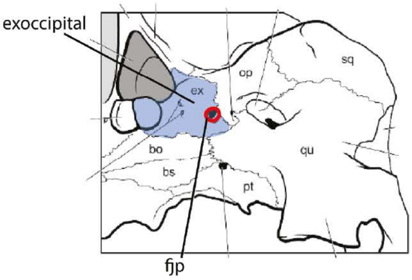

Azabbaremys moragjonesi
State 2

Exoccipital (for. jugulare posterius (fjp))

Character 83

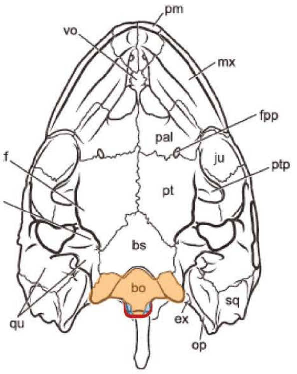

Podocnemis unifilis
State 0

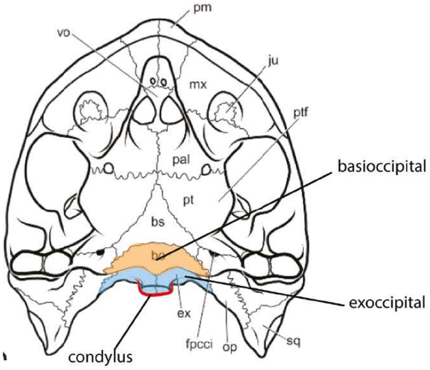

Bothremys maghrebiana
State 1

Exoccipital (condylus occipitalis bones)

Character 84

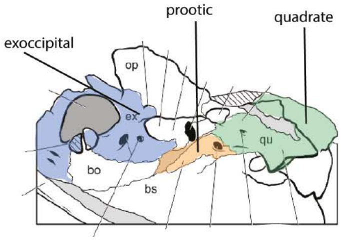

Araripemys barretoi
State 0

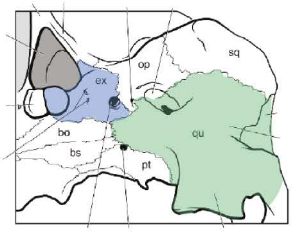

Azabbaremys moragjonesi
State 1

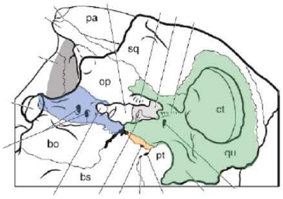

Euraxemys essweni
State 2

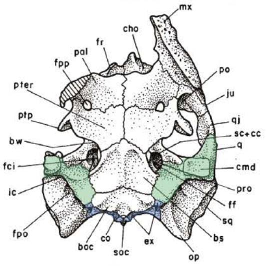

Brasilemys josai
State 3

Exoccipital (exoccipital-quadrate contact)

Character 85

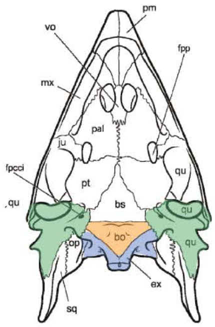

Labrostochelys galkini
State 0

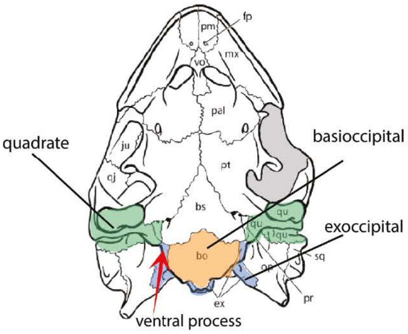

Euraxemys essweni
State 1

Exoccipital (ventral process)

Character 86

Plate 15. Characters 83 to 86

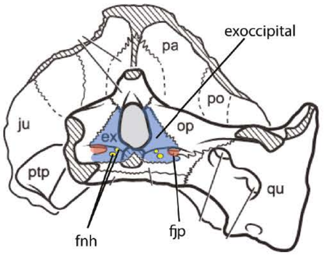

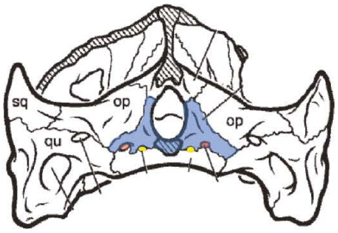

Lemurchelys diasphax
State 0

Latentemys plowdeni
State 1

Exoccipital (foramen nervi hypoglossi)

Character 87

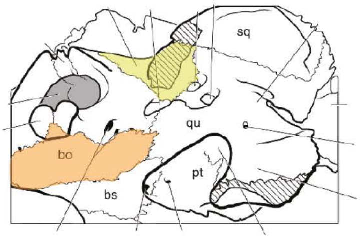

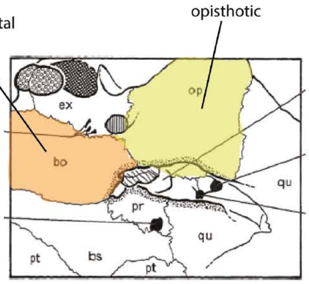

Galianemys emringeri
State 0

Pelomedusa subrufa
State 1

Basioccipital (basioccp.-opist. contact)

Character 89

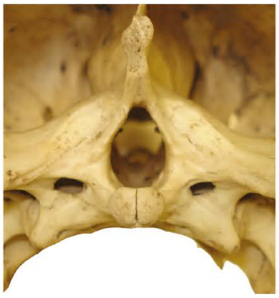

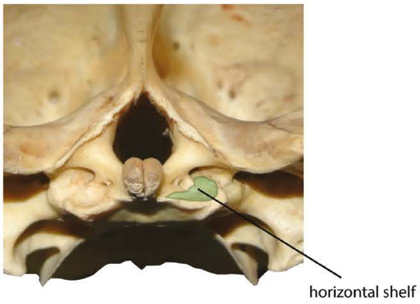

Peltocephalus dumerilianus
State 0

Erymnochelys madagascariensis
State 1

Basioccipital (horizontal shelf)

Character 91

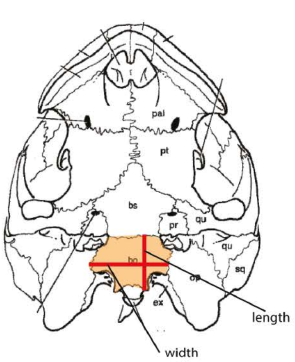

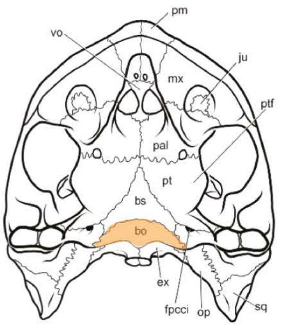

Pelomedusa subrufa
State 0

Bothremys maghrebiana
State 1

Basioccipital (length/width ratio)

Character 88

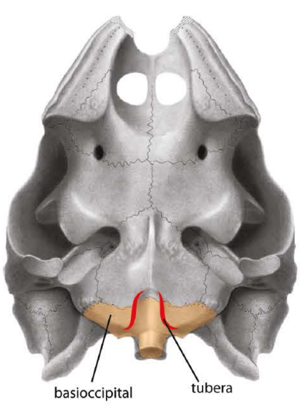

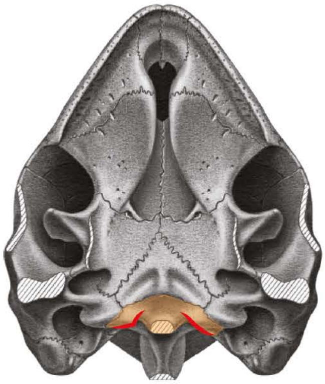

Hamadachelys escuilliei
State 0

Cordichelys antiqua
State 1

Basioccipital (tubera position)

Character 90

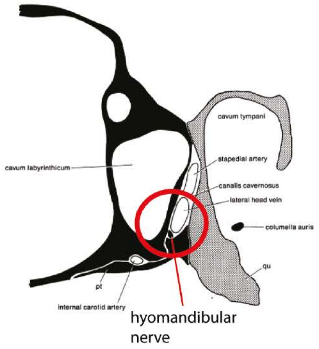

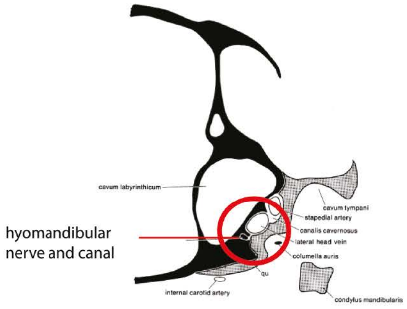

Chelydra serpetina
State 0

Podocnemis expansa
State 1

Prootic (hyomandibular nerve and canal)

Character 92

Plate 16. Characters 87 to 92

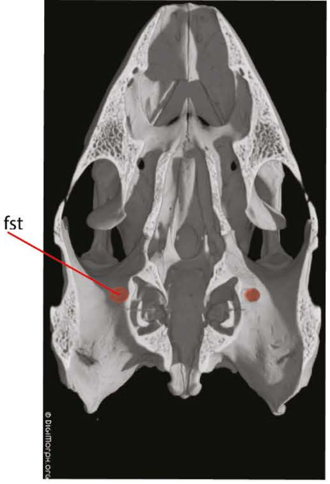

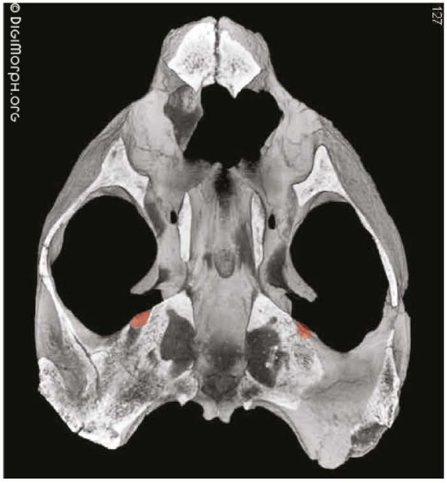

Podocnemis unifilis

State 0

Galianemys whitei

State 1

Prootic (for. stap.-temp. orientation)

Character 93

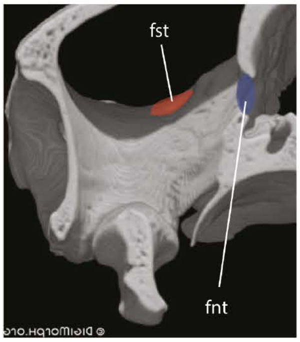

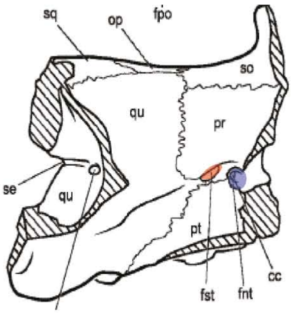

Podocnemis unifilis

State 0

Bothremys cooki

State 1

Prootic (for. stap.-temp.-for. nervi trigemini)

Character 94

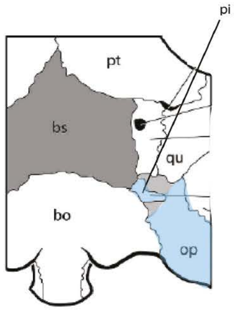

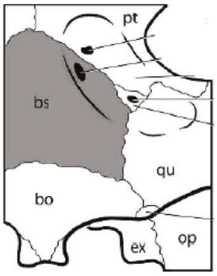

Emydura macquarri

State 0

Euraxemys essweni

State 1

Opisthotic (processus interfenestralis (pi))

Character 97

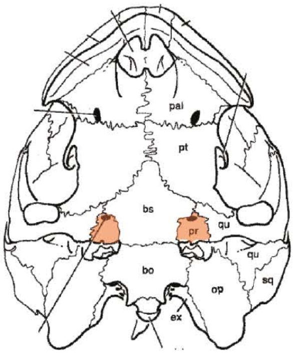

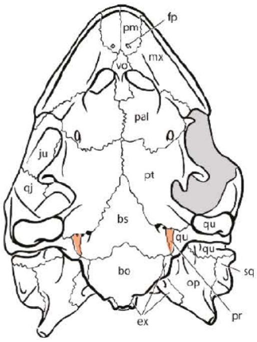

Pelomedusa subrufa

State 0

Euraxemys essweni

State 1

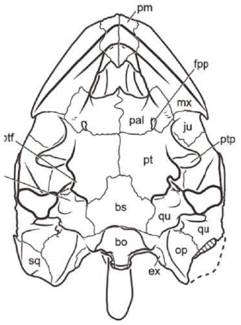

Podocnemis sextuberculata

State 2

Prootic (ventral exposure)

Character 95

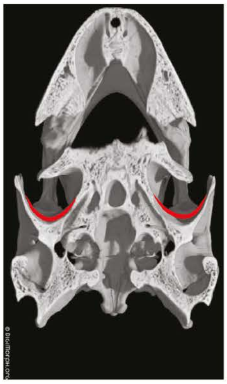

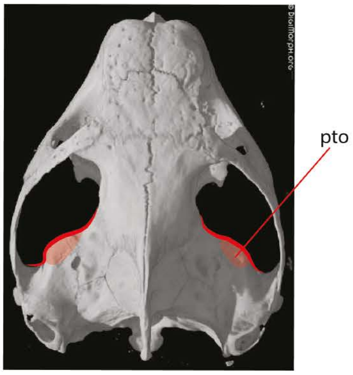

Podocnemis unifilis

State 0

Gopherus polyphemus

State 1

Prootic (processus trochlearis oticum (pto))

Character 96

Plate 17. Characters 93 to 97

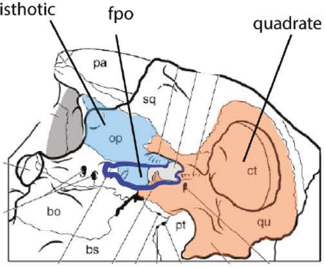

Euraxemys essweni

State 0

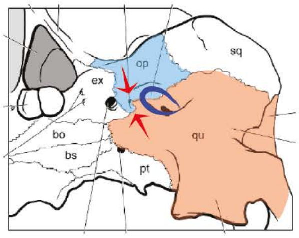

Azabbaremys moragjonesi

State 1

Opisthotic (fen. post. (fpo) closed medially)

Character 98

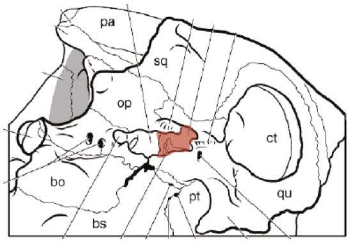

Euraxemys essweni

State 0

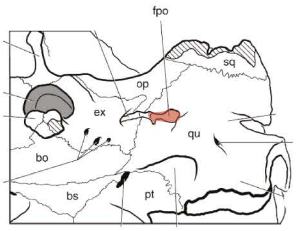

Galianemys whitei

State 1

Opisthotic (fenst. post. horizontal slit)

Character 99

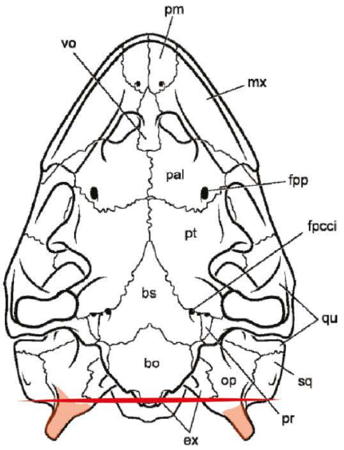

Euraxemys essweni

State 0

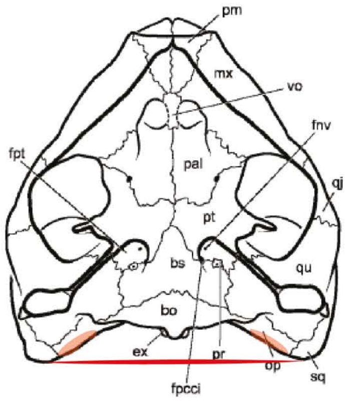

Galianemys emringeri

State 1

Opisthotic (processus paraoccipitalis)

Character 100

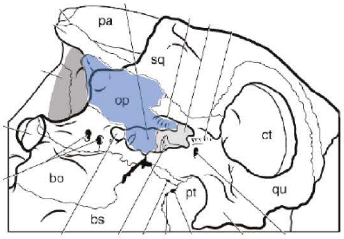

Euraxemys essweni

State 0

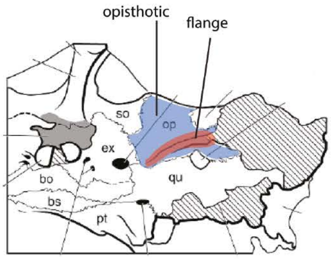

Chedighaii hutchisoni

State 1

Opisthotic (thin horizontal flange)

Character 101

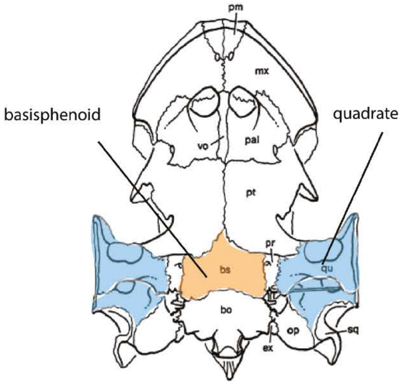

Emydura macquarrii

State 0

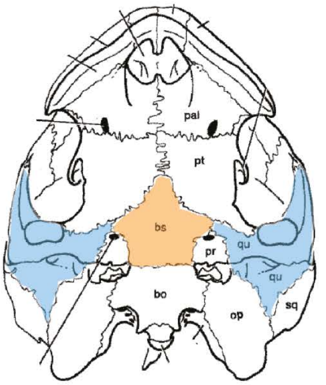

Pelomedusa subrufa

State 1

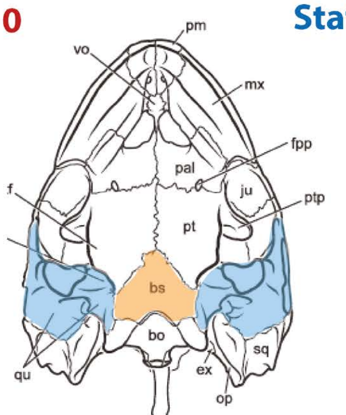

Podocnemis unifilis

State 2

Basiphenoid (basiph-quad. contact)

Character 102

Plate 18. Characters 98 to 102

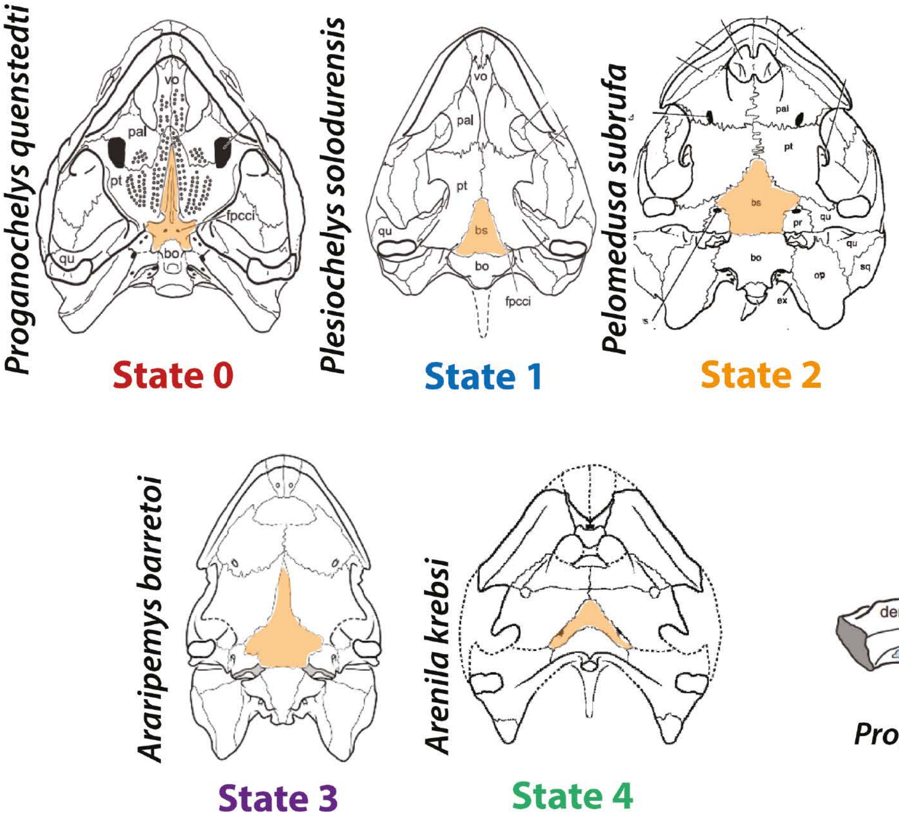

Basiphenoid (ventral outline)

Character 103

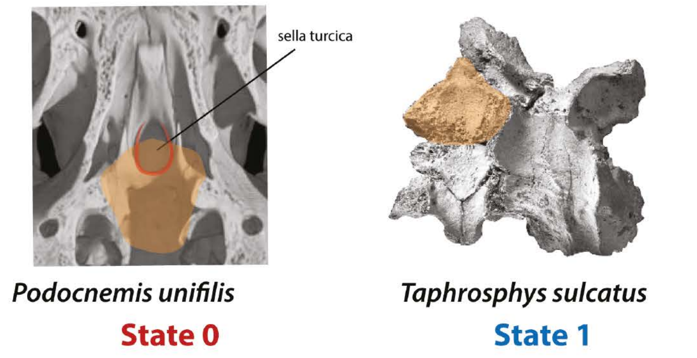

Basiphenoid (sella turcica/dorsum sella)

Character 105

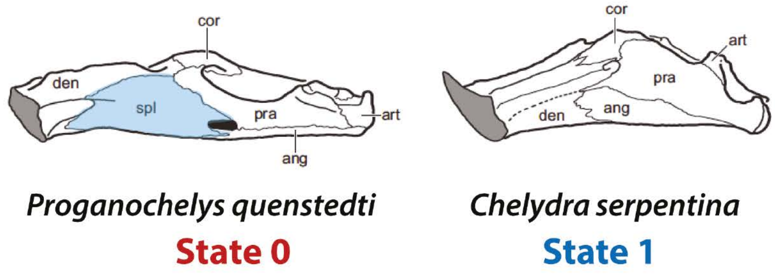

Splenial (splenial bone presence/absence)

Character 106

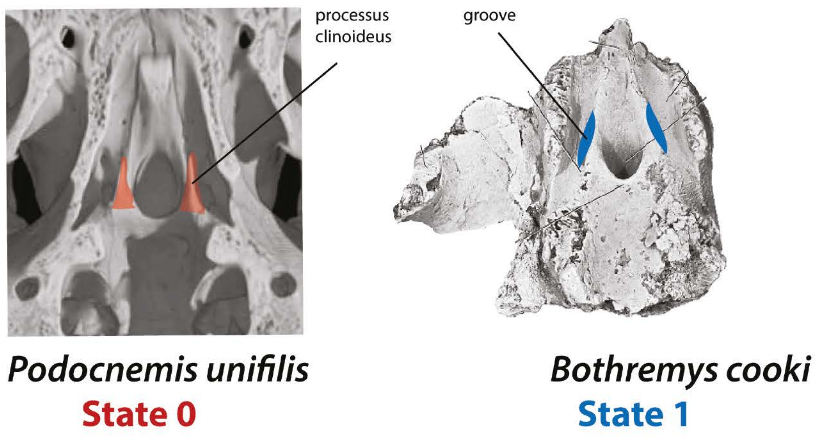

Basiphenoid (processus clinoideus)

Character 104

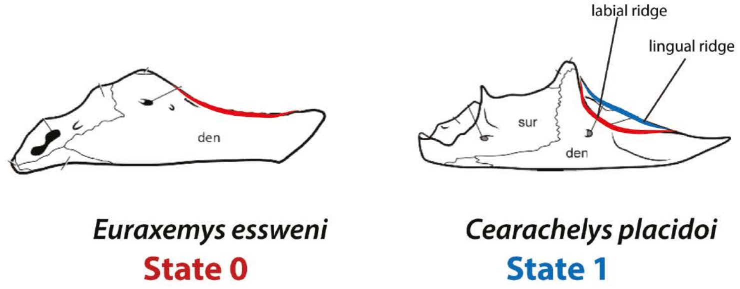

Dentary (lingual ridge)

Character 107

Plate 19. Characters 103 to 107

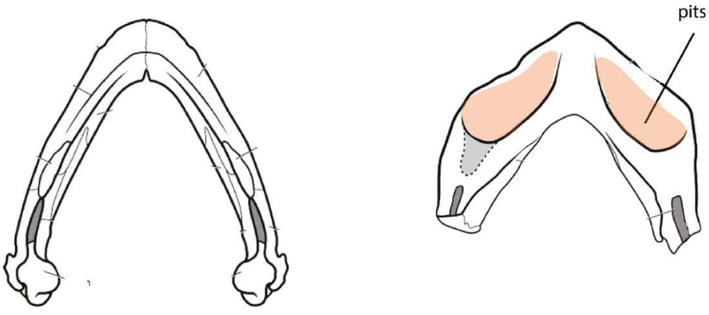

Euraxemys essweni
State 0

Bothremys cooki
State 1

Dentary (pits)

Character 108

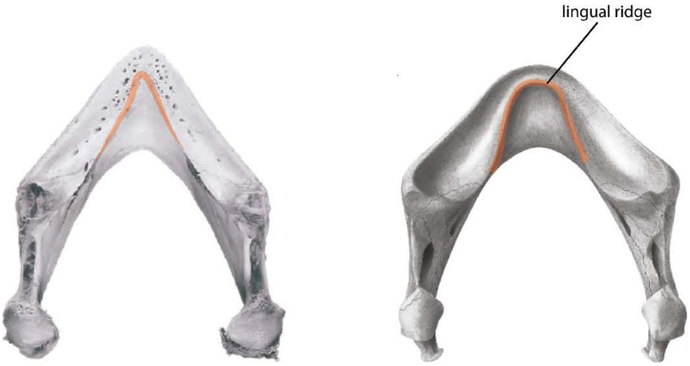

Erymnochelys madagascariensis
State 0

Kurmademys kallamedensis
State 1

Dentary (U-shaped ling. ridge at symphysis)

Character 109

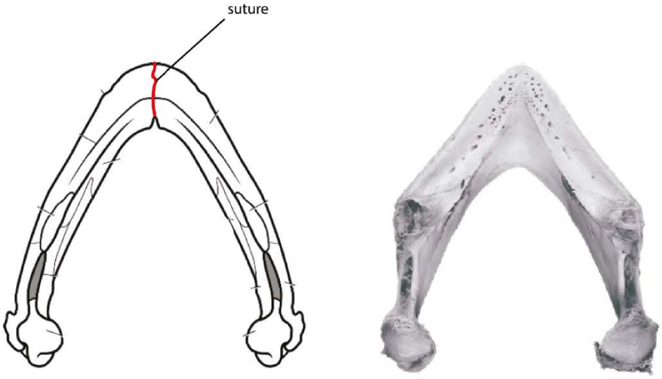

Euraxemys essweni
State 0

Erymnochelys madagascariensis
State 1

Dentary (fused/sutured symphysis)

Character 110

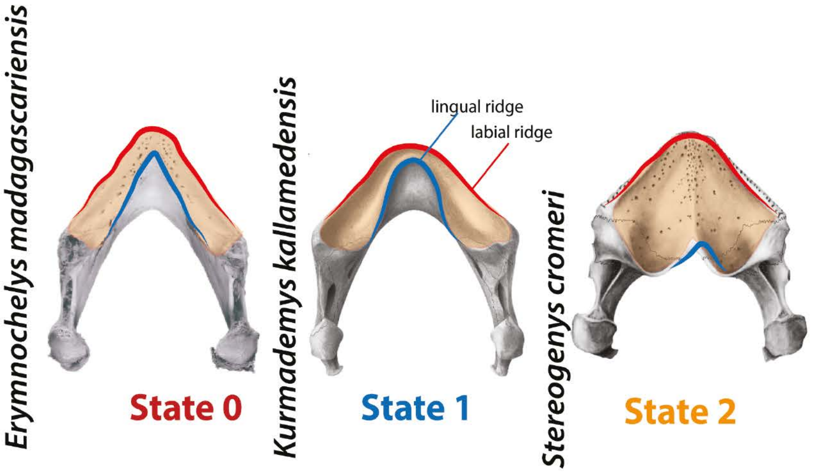

Dentary (triturating surface width)

Character 111

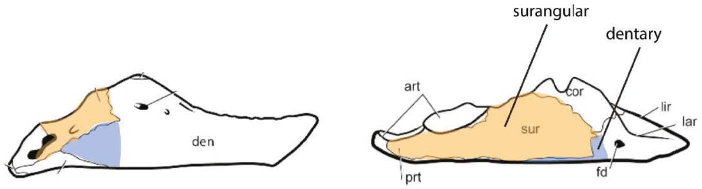

Euraxemys essweni
State 0

Bothremys maghrebiana
State 1

Dentary (posterolateral exposure)

Character 112

Plate 20. Characters 108 to 112

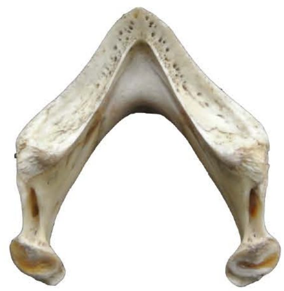

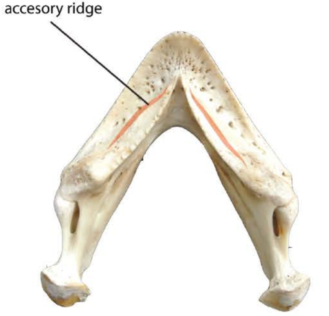

Erymnochelys madagascariensis
State 0

Podocnemis expansa
State 1

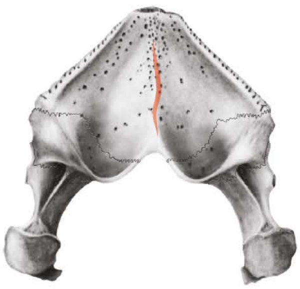

Stereogenys cromeri
State 2

Dentary (accesory ridge)

Character 113

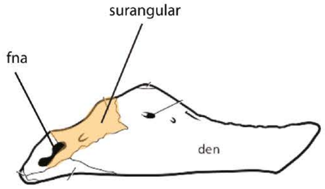

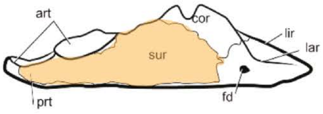

Euraxemys essweni
State 0

Bothremys maghrebiana
State 1

Surangular (for. ner. auri. (fna))

Character 115

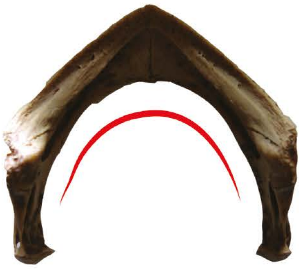

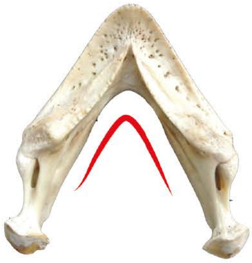

Pelomedusa subrufa
State 0

Podocnemis expansa
State 1

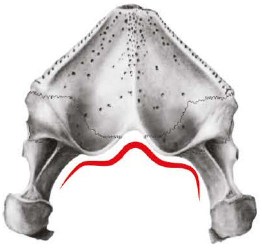

Stereogenys cromeri
State 2

Dentary (medial margin shape and angle)

Character 114

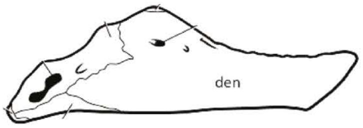

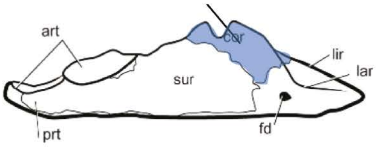

Euraxemys essweni
State 0

Bothremys maghrebiana
State 1

Coronoid (lateral exposure)

Character 116

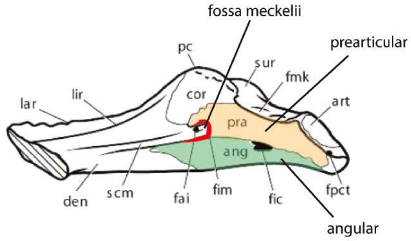

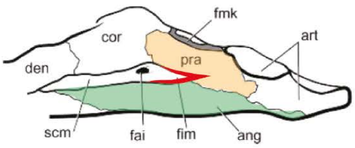

Euraxemys essweni
State 0

Bothremys maghrebiana
State 1

Prearticular (prearticular-angular contact)

Character 117

Plate 21. Characters 113 to 117

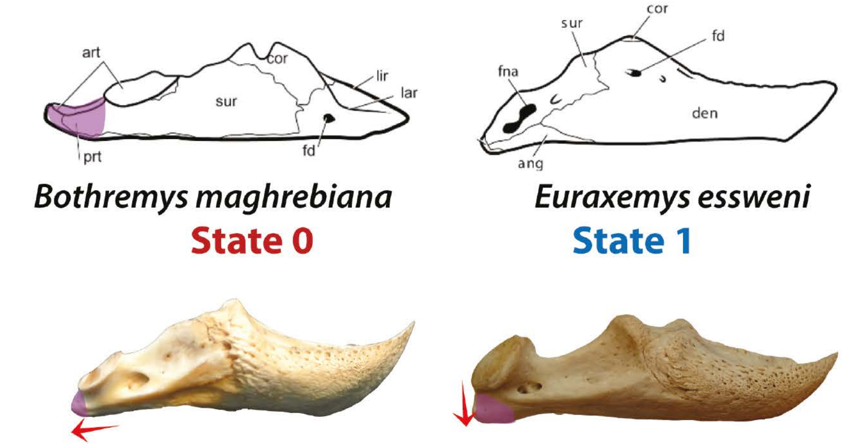

Bothremys maghrebiana — State 0

Euraxemys essweni — State 1

Erymnochelys madagascariensis — State 2

Podocnemis expansa — State 3

Articular (procesuss retroarticularis)

Character 118

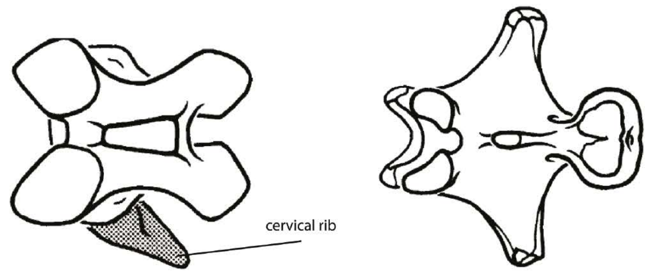

Proganochelys quenstedti — State 0

Podocnemis expansa — State 1

Cervicals (cervical ribs)

Character 120

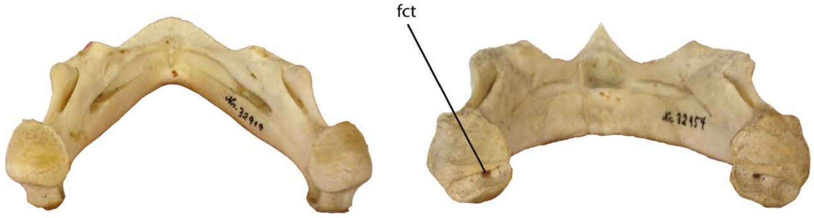

Podocnemis expansa — State 0

Peltocephalus dumerilianus — State 1

Articular (foramen chorda tympani (fct))

Character 119

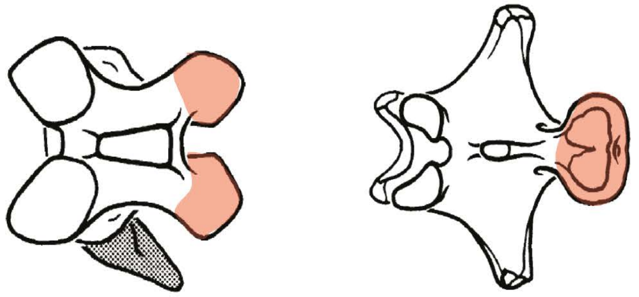

Proganochelys quenstedti — State 0

Podocnemis expansa — State 1

Cervicals (cervical postzygapophyses)

Character 121

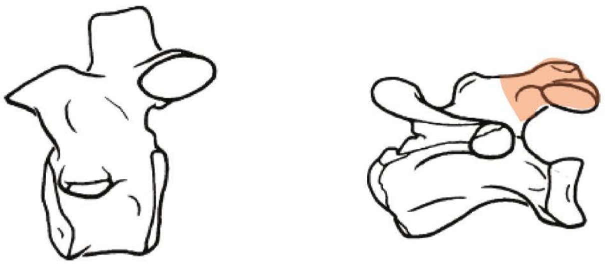

Proganochelys quenstedti — State 0

Podocnemis expansa — State 1

Cervicals (postzygapophyses post. ext.)

Character 122

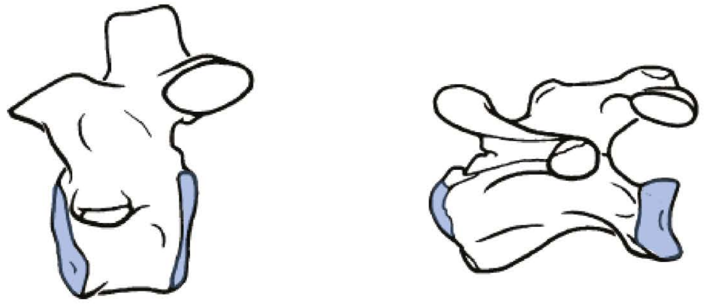

Proganochelys quenstedti — State 0

Podocnemis expansa — State 1

Cervicals (cervical centra)

Character 123

Plate 22. Characters 118 to 123

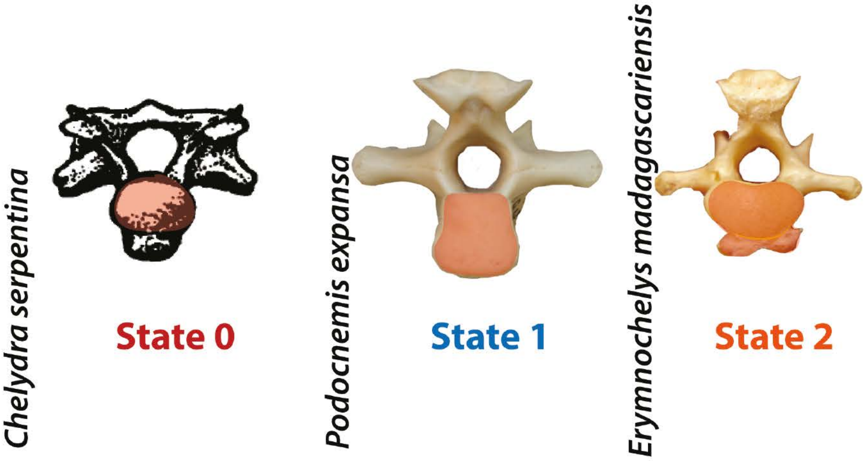

Cervicals (cervical centra shape)

Character 124

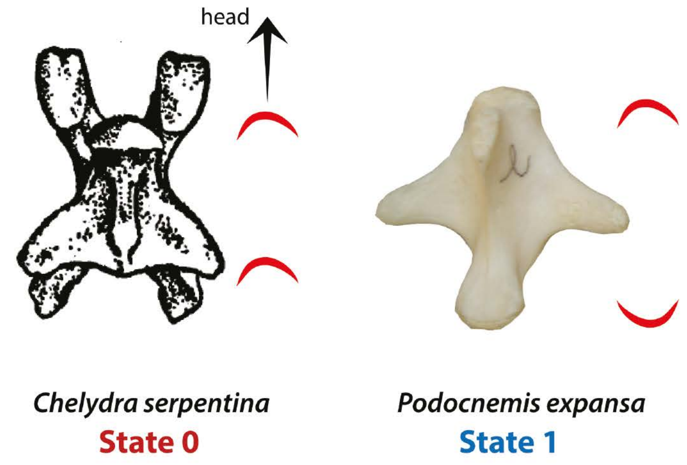

Cervicals (cervical 2, biconvex)

Character 125

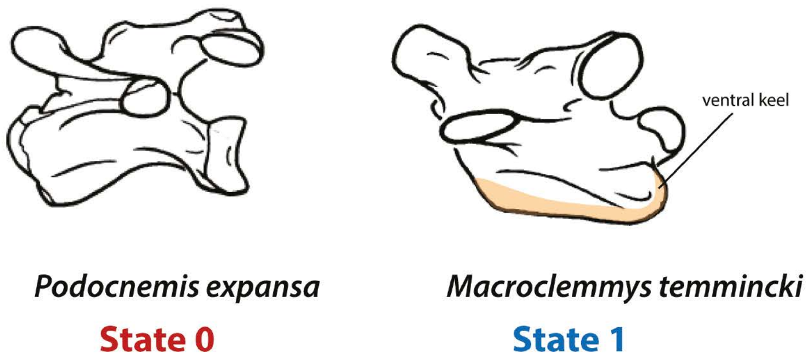

Cervicals (ventral keel)

Character 126

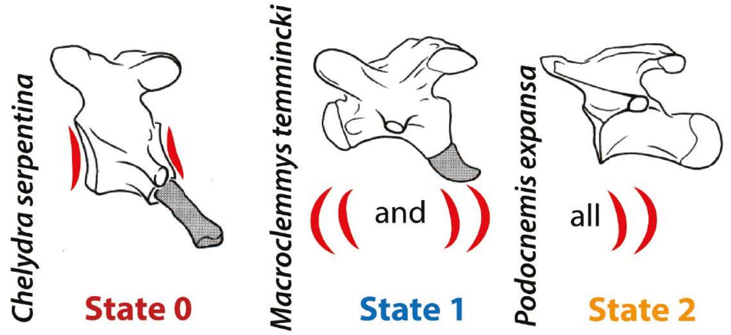

Caudals (centra articulations)

Character 127

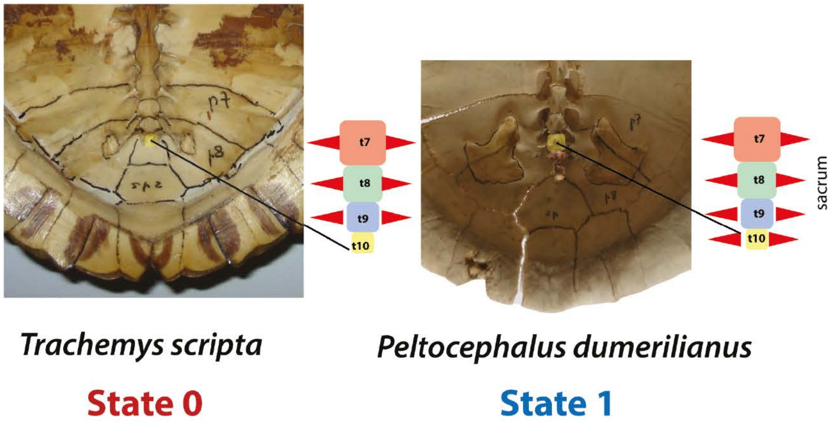

Pelvic girdle (tenth thoracic centrum)

Character 128

Plate 23. Characters 124 to 128

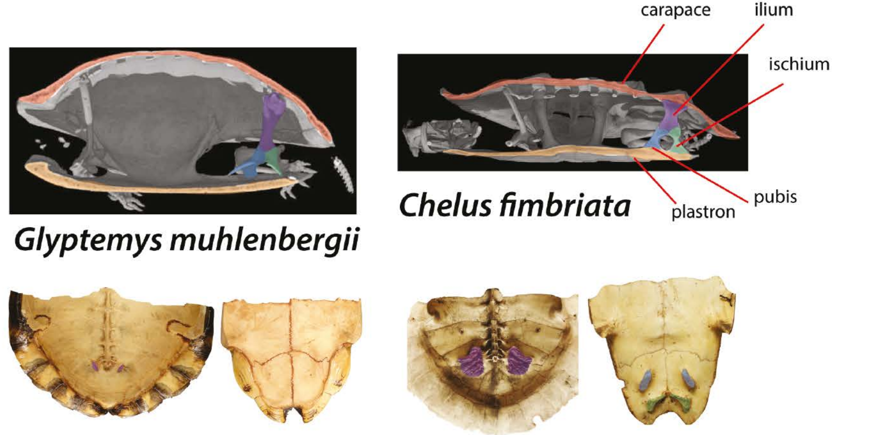

Glyptemys muhlenbergii *Chelus fimbriata*

Rhinoclemmys pulcherrima *Erymnochelys madagascariensis*

State 0 **State 1**

Pelvic girdle (pelvis sutured to shell)

Character 129

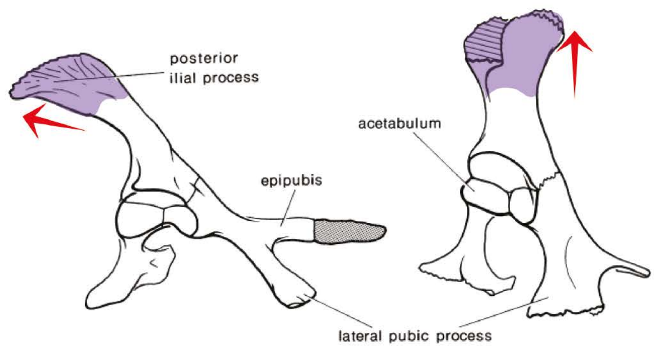

Macroclemys temmincki *Podocnemis expansa*

State 0 **State 1**

Pelvic girdle (dorsal part of illium)

Character 130

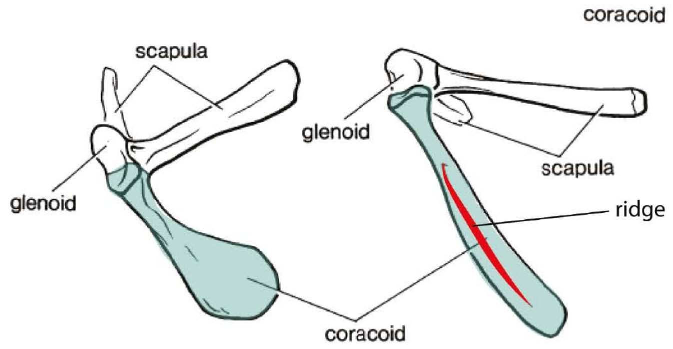

Macroclemys temmincki *Podocnemis expansa*

State 0 **State 1**

Shoulder girdle (coracoid dorsl. ridge)

Character 132

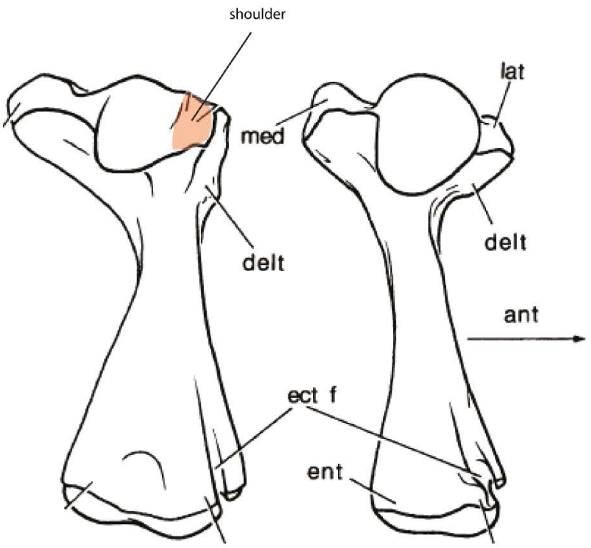

Macroclemys temmincki *Podocnemis expansa*

State 0 **State 1**

Humerus (shoulder on lateral side of head)

Character 133

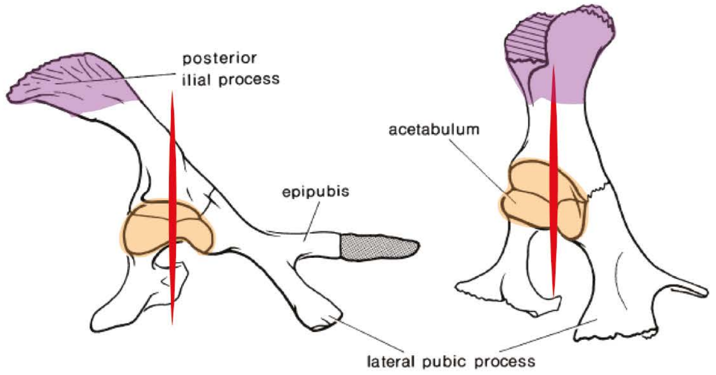

Macroclemys temmincki *Podocnemis expansa*

State 0 **State 1**

Pelvic girdle (acetabulum vs ilium)

Character 131

Plate 24. Characters 129 to 133

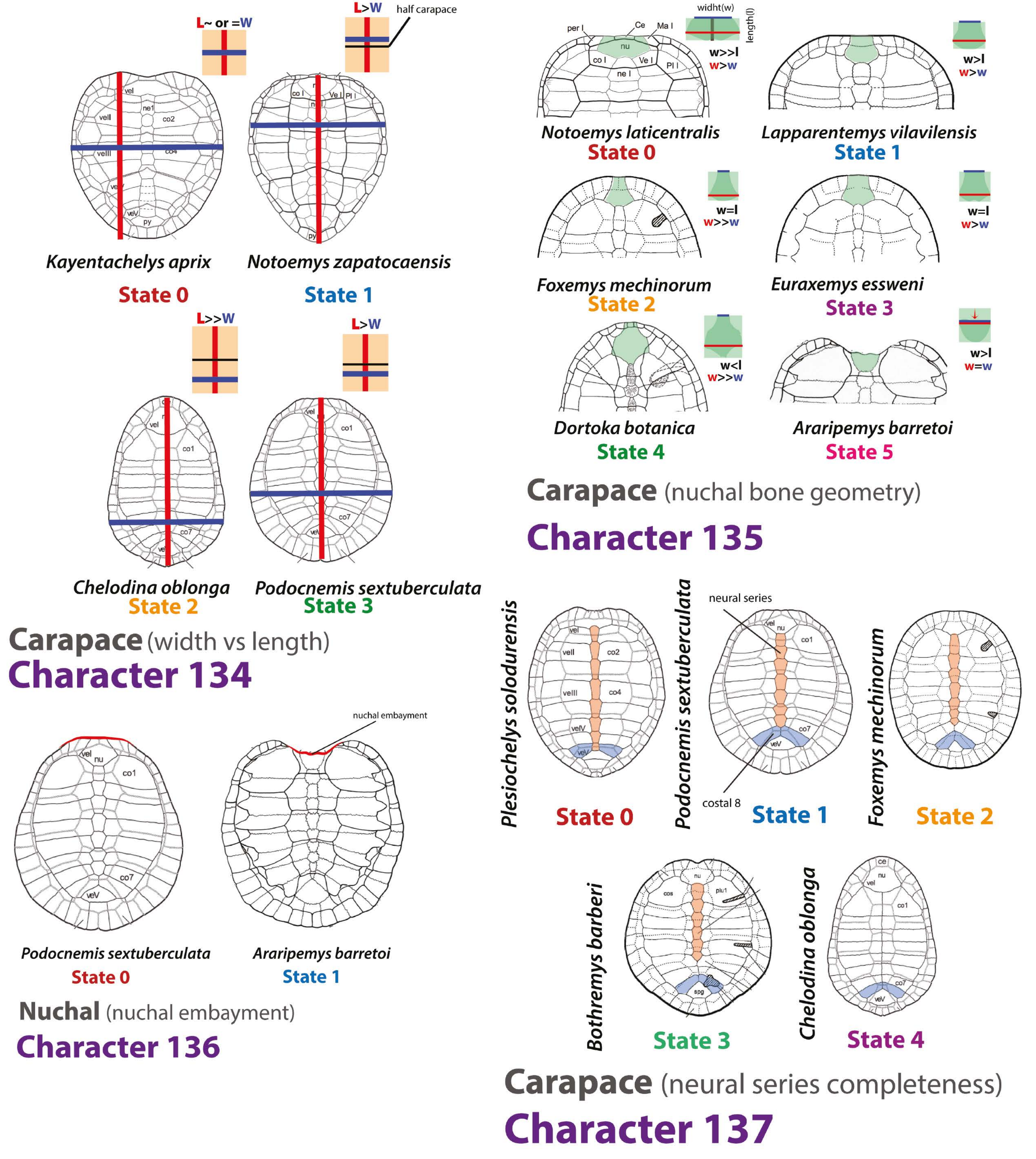

Plate 25. Characters 134 to 137

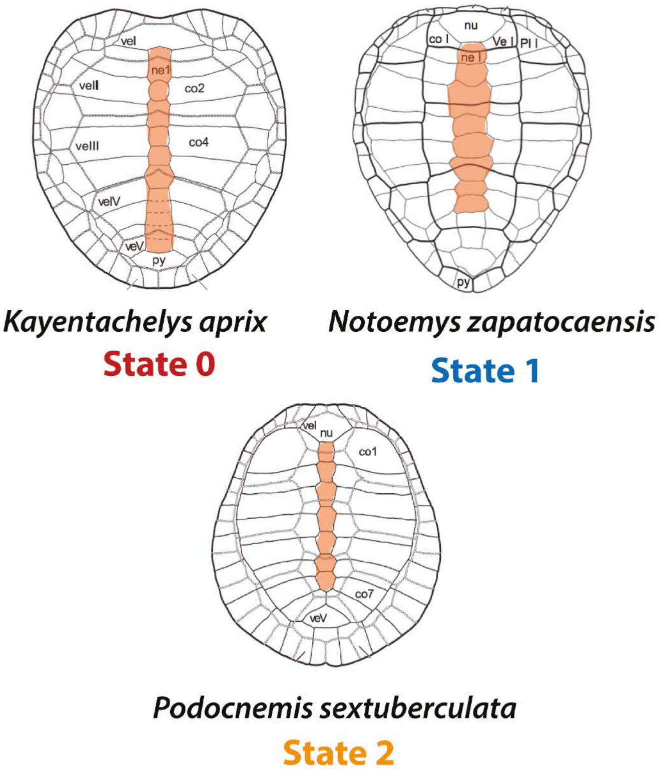

Neurals (neural series pattern)
Character 138

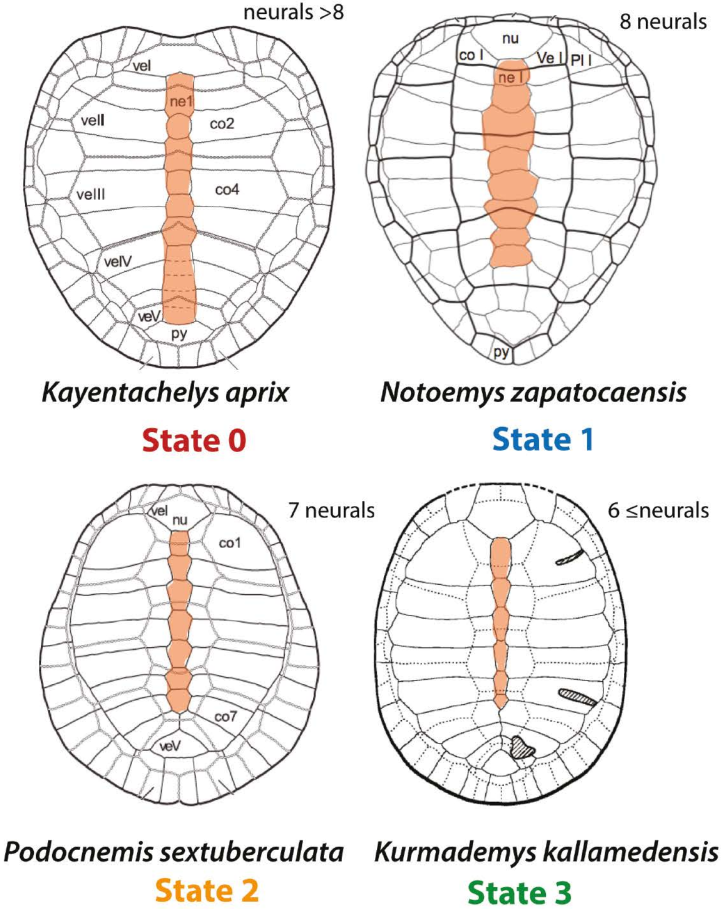

Neurals (neural number)
Character 139

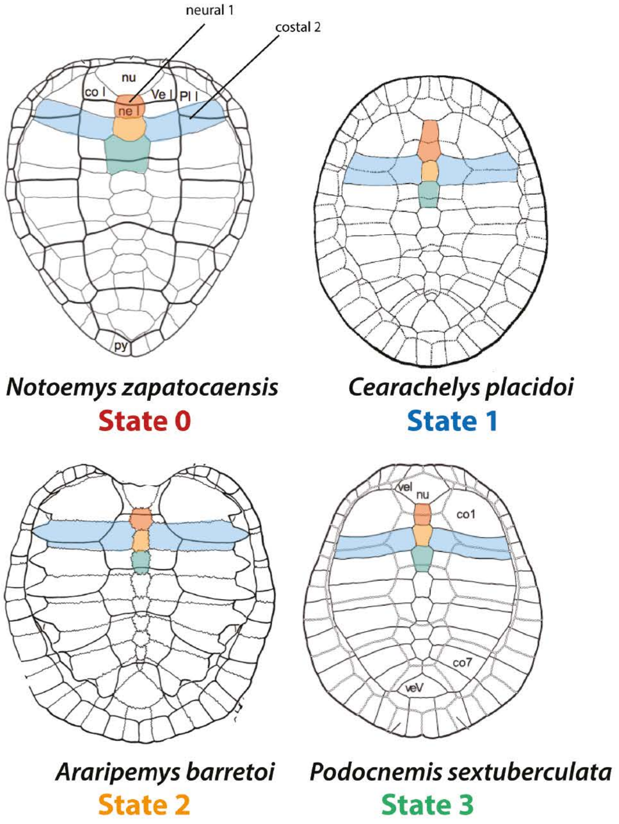

Neural 1 (neurals-costals 1, 2, 3 and 4)
Character 140

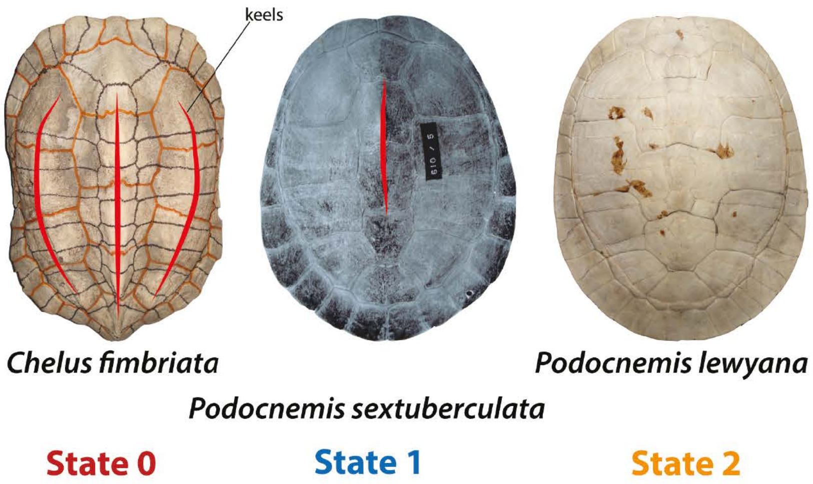

Neurals (keeled neurals and costals)
Character 141

Plate 26. Characters 138 to 141

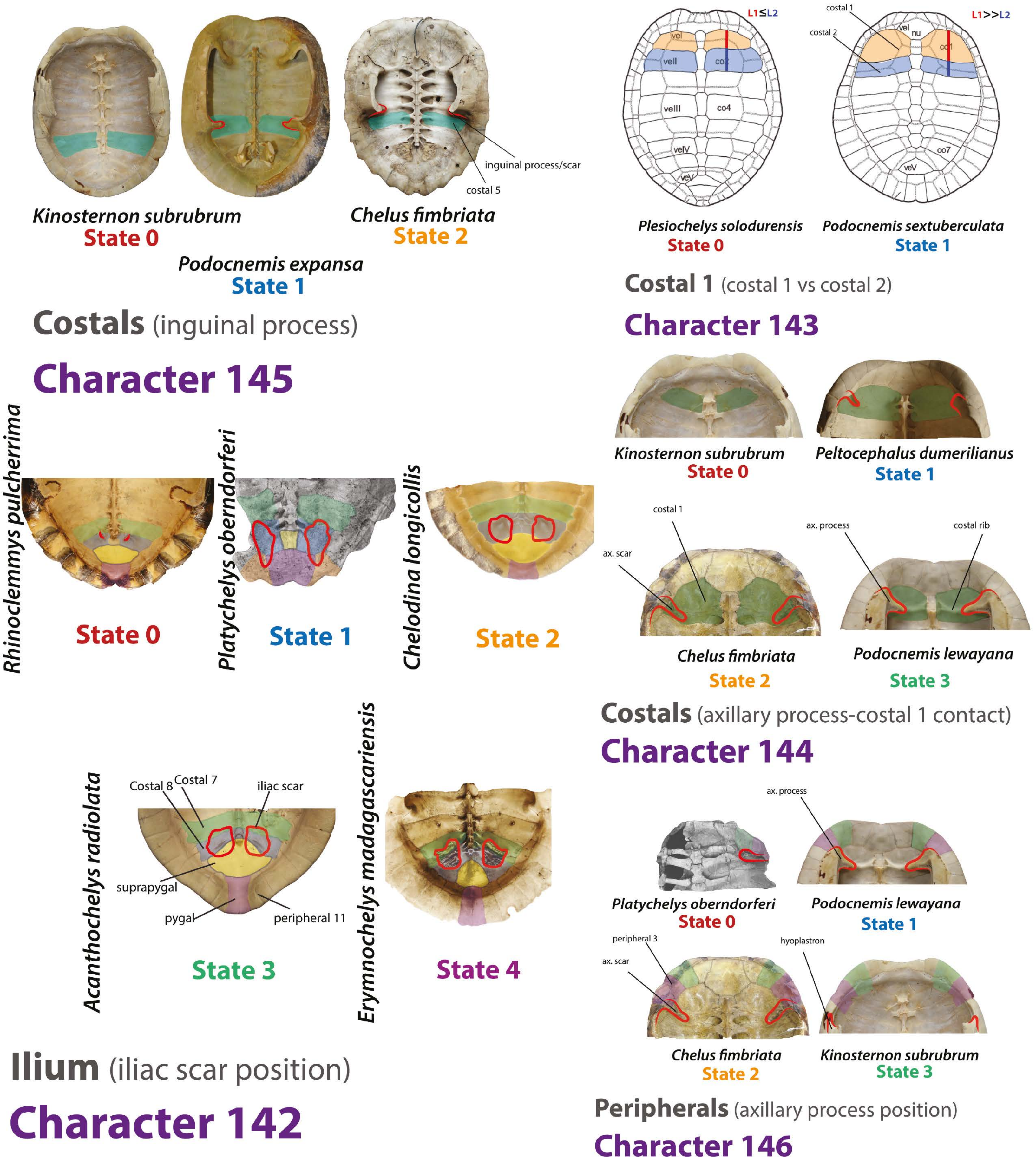

Plate 27. Characters 142 to 146

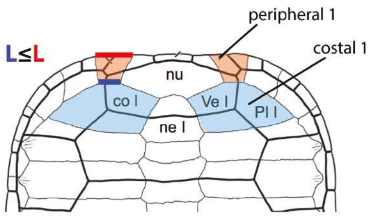

Notoemys laticentralis
State 0

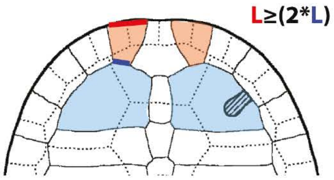

Foxemys mechinorum
State 1

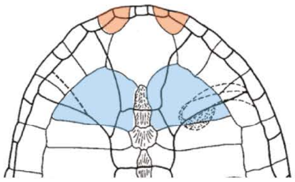

Dortoka botanica
State 2

Peripheral 1 (peripheral 1-costal 1)

Character 147

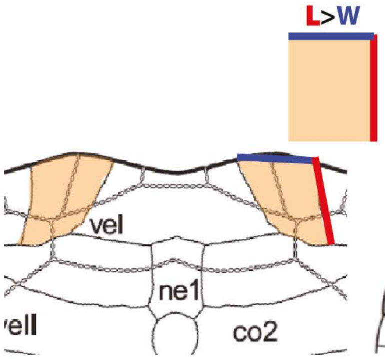

Kayentachelys
State 0

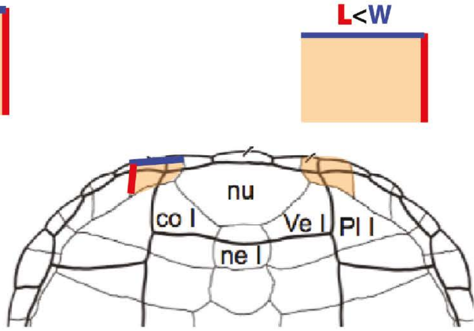

Notoemys zapatocaensis
State 1

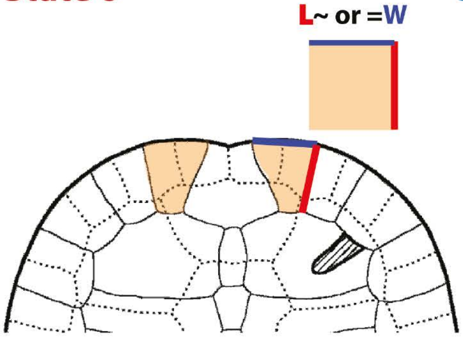

Polysternon provinciale
State 2

Peripheral 1 (width vs length)

Character 148

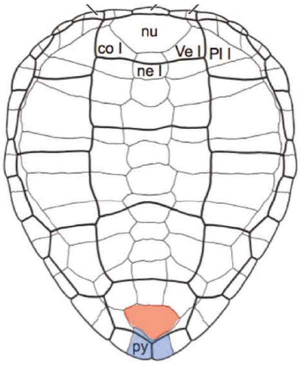

Notoemys zapatocaensis
State 0

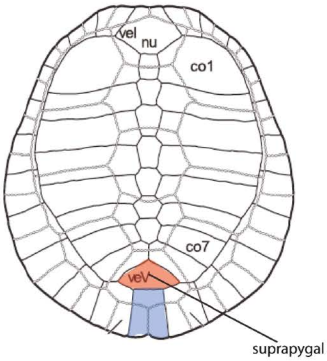

Podocnemis sextuberculata
State 1

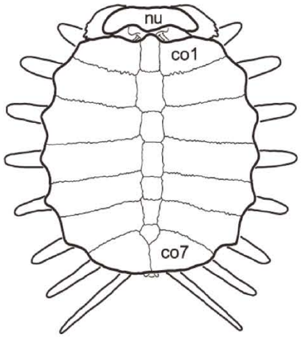

Amyda carilaginea
State 2

Suprapygal (shape of suprapygal)

Character 149

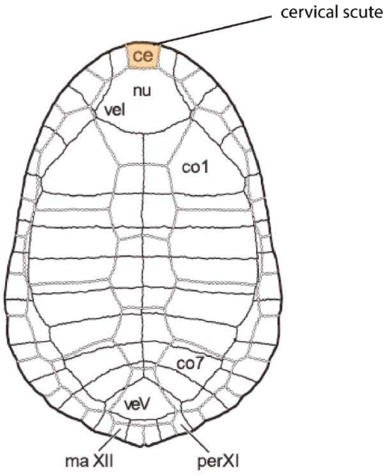

Chelodina oblonga
State 0

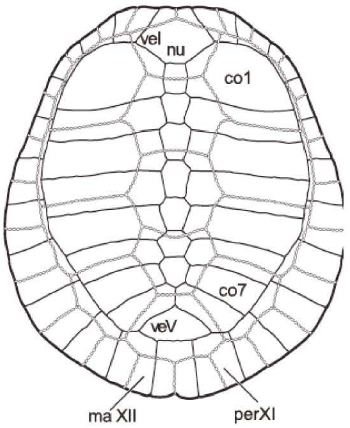

Podocnemis sextuberculata
State 1

Carapace (cervical scute)

Character 150

Plate 28. Characters 147 to 150

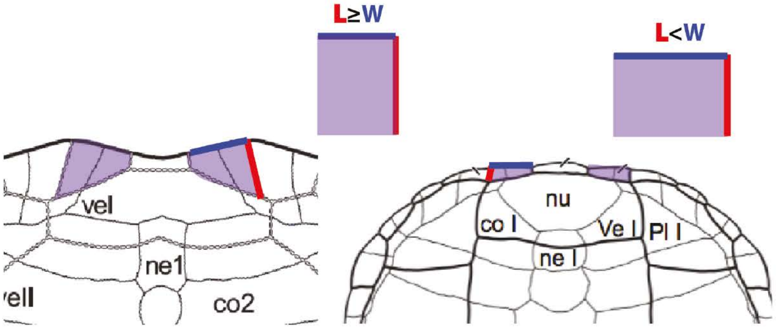

Kayentachelys aprix
State 0

Notoemys zapatocaensis
State 1

Marginals 1 (width vs length)

Character 151

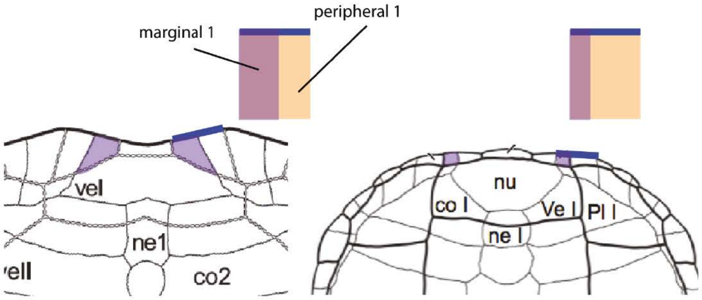

Kayentachelys aprix
State 0

Notoemys zapatocaensis
State 1

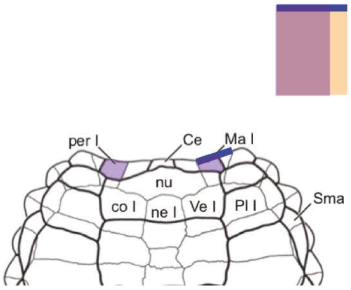

Platychelys oberdonferi
State 2

Marginals 1 (portion covering peripheral 1)

Character 152

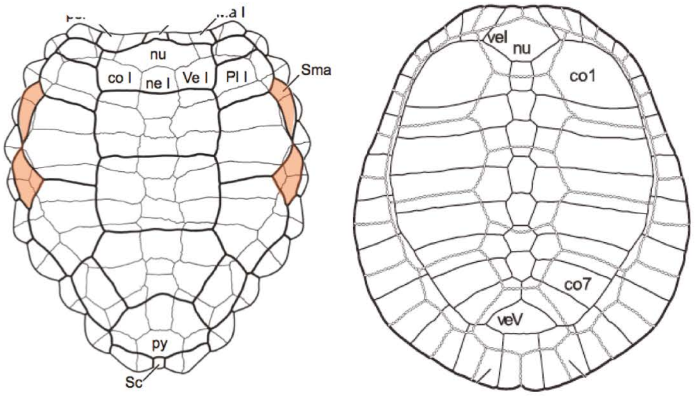

Platychelys oberndorferi
State 0

Podocnemis sextuberculata
State 1

Supramarginals (absence/presence)

Character 153

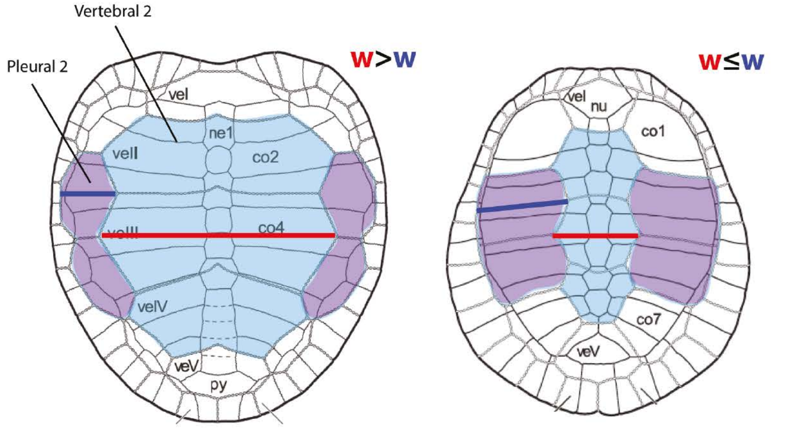

Kayentachelys aprix
State 0

Podocnemis sextuberculata
State 1

Vertebrals (vertebral scute 2-4 width)

Character 154

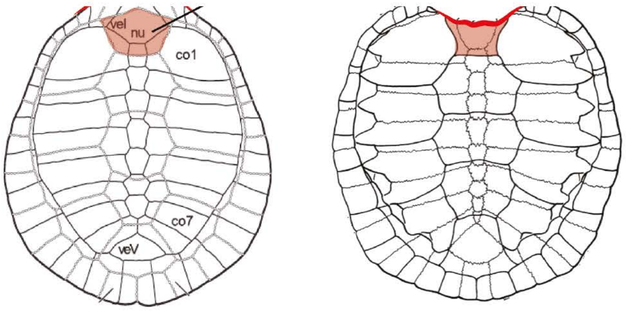

Podocnemis sextuberculata
State 0

Araripemys barretoi
State 1

Vertebrals (vertebral 1 vs ant. carp. marg.)

Character 155

Plate 29. Characters 151 to 155

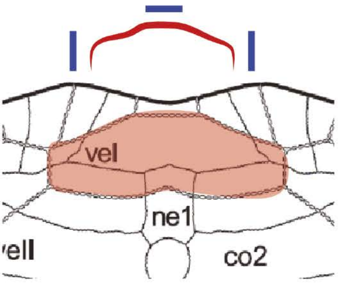

Kayentachelys aprix
State 0

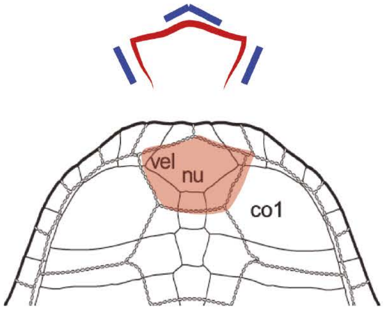

Podocnemis sextuberculata
State 1

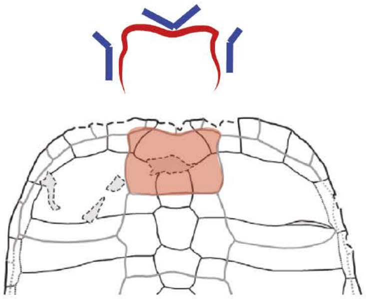

Neochelys liriae
State 2

Vertebral 1 (anterolateral margins)

Character 156

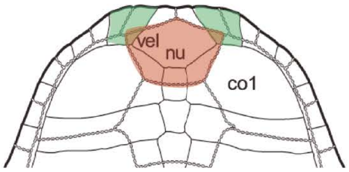

Podocnemis sextuberculata
State 0

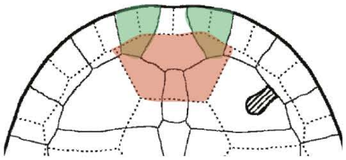

Foxemys mechinorum
State 1

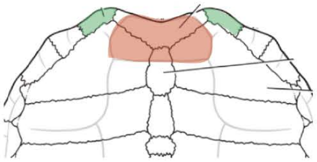

Langanemys tenerensis
State 2

Vertebral 1 (vertebral 1 vs. per. and nuchal)

Character 157

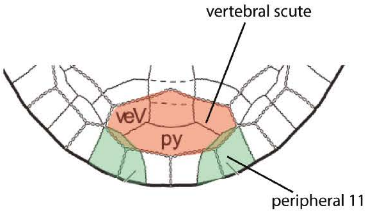

Kayentachelys aprix
State 0

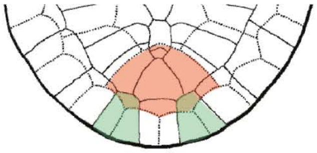

Cearachelys placidoi
State 1

Vertebrals (overlapping peripheral 10)

Character 159

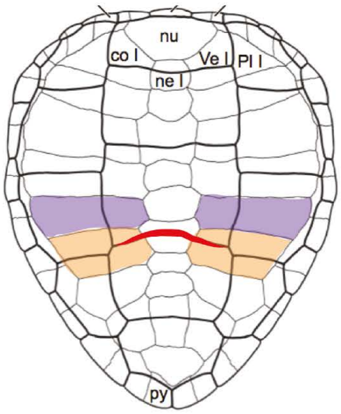

Notoemys zapatocaensis
State 0

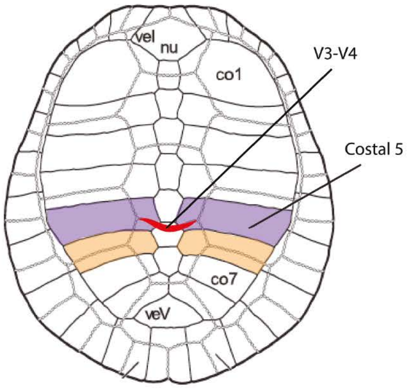

Podocnemis sextuberculata
State 1

Vertebrals 3-4 (vertebrals 3-4 contact)

Character 158

Plate 30. Characters 156 to 159

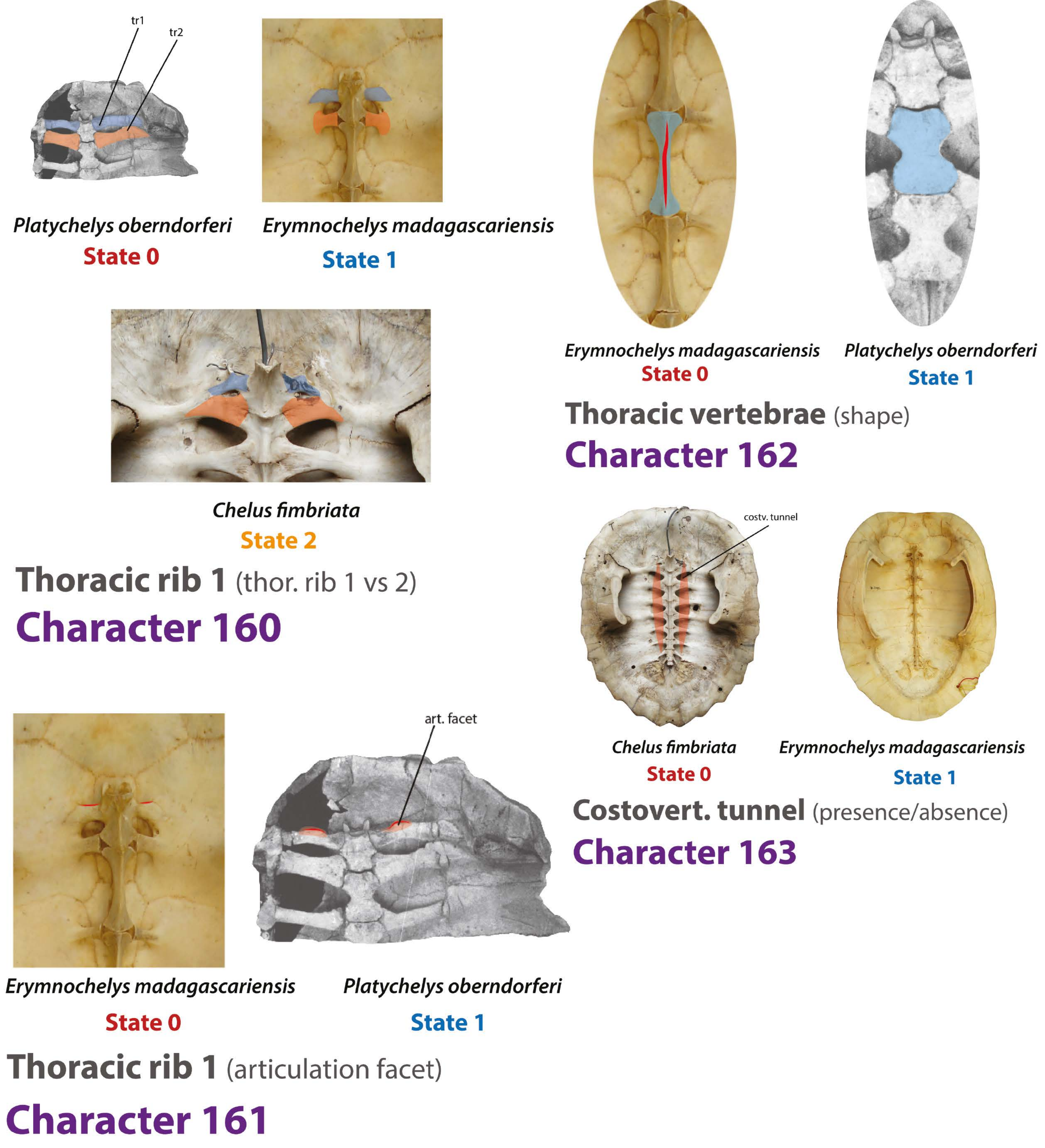

Plate 31. Characters 160 to 163

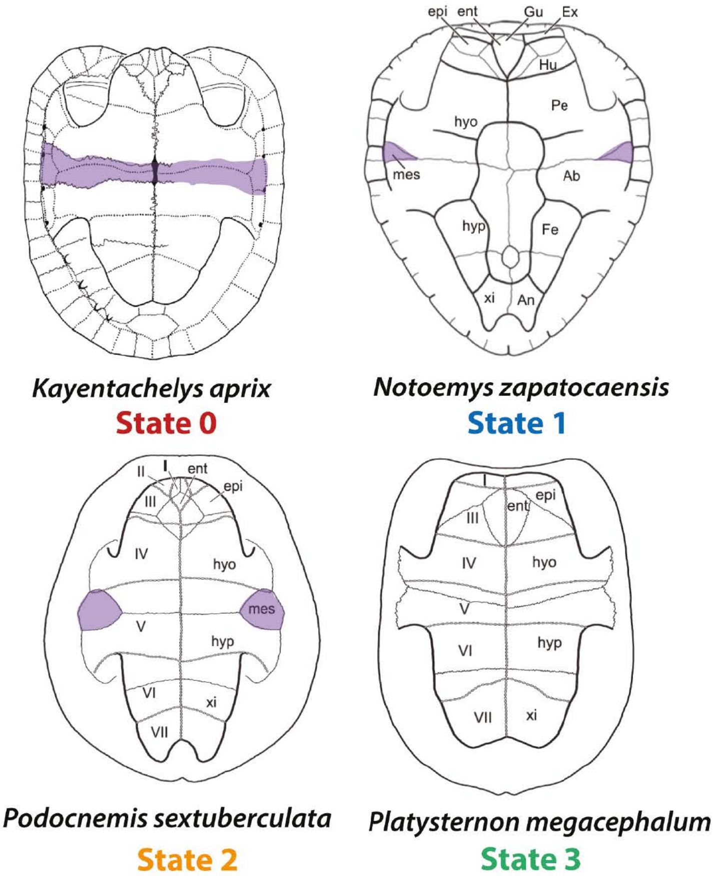

Mesoplastron (absence/presence)

Character 164

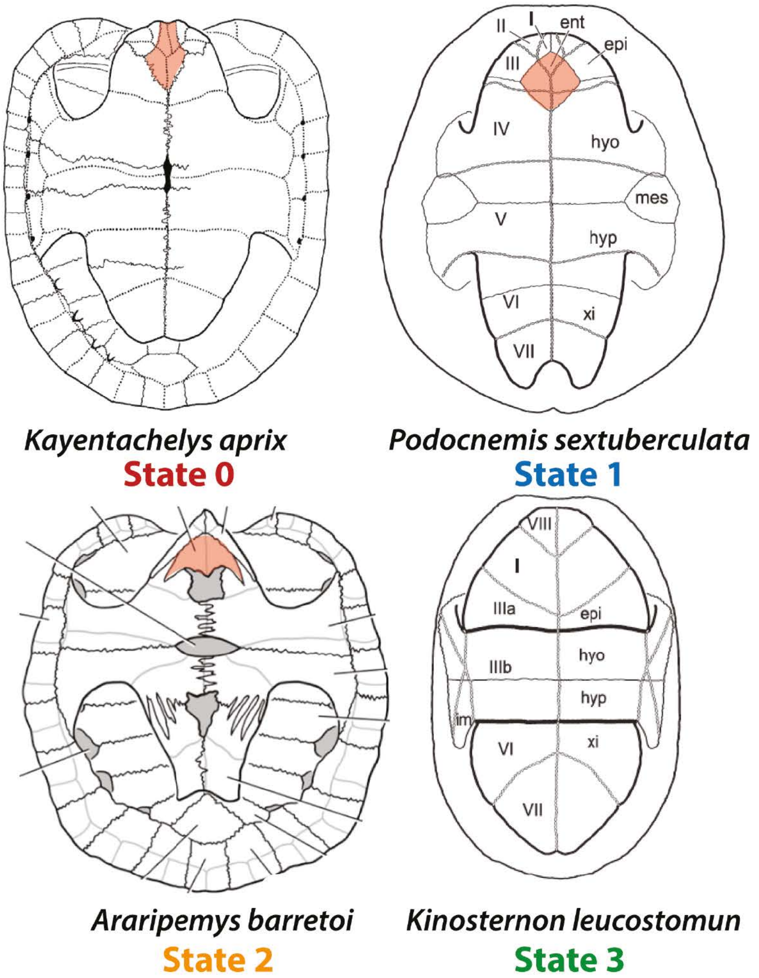

Entoplastron (shape of the entoplastron)

Character 165

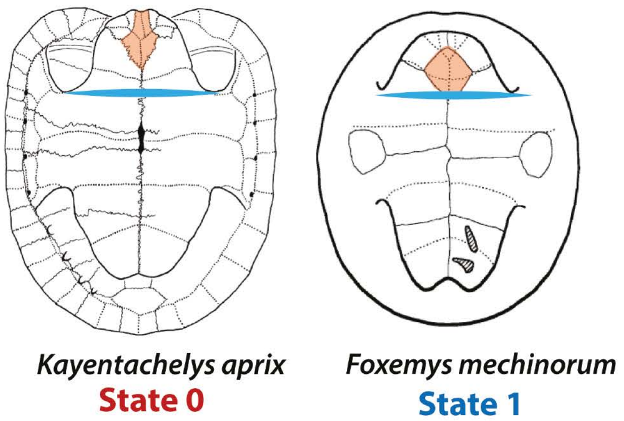

Entoplastron (entoplastron vs axil. notch)

Character 166

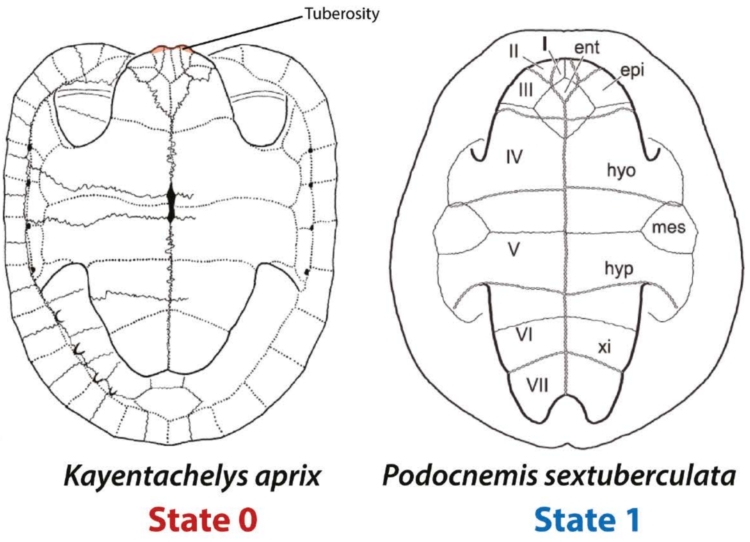

Epiplastron (anter. projected tuberosities)

Character 167

Plate 32. Characters 164 to 167

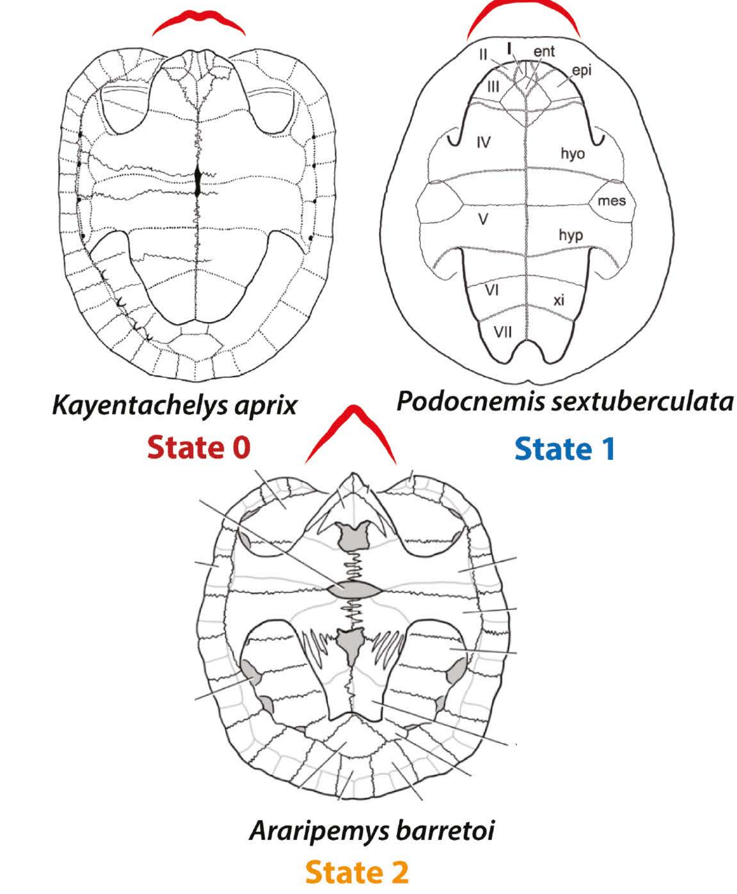

Epiplastron (anterior plastral lobe shape)

Character 168

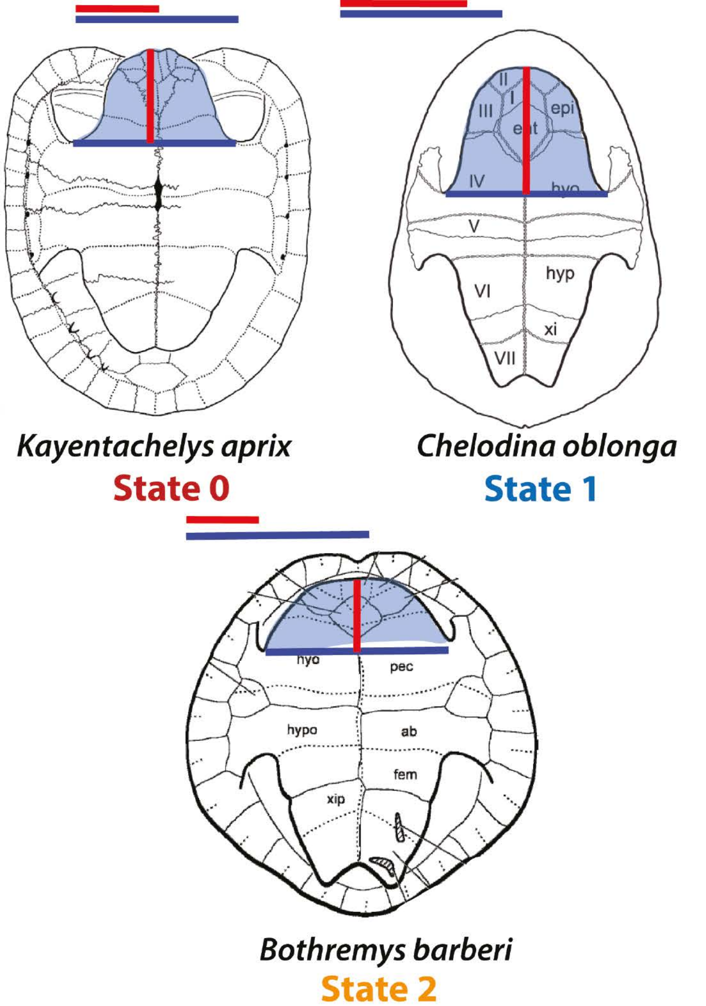

Epiplastron (ant. plast. lobe w/l ratio)

Character 169

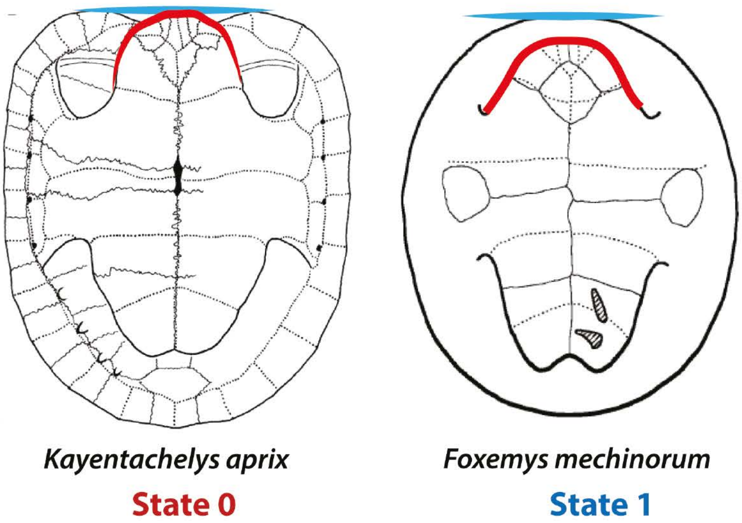

Epiplastron (ant. plast. vs ant. carapace)

Character 170

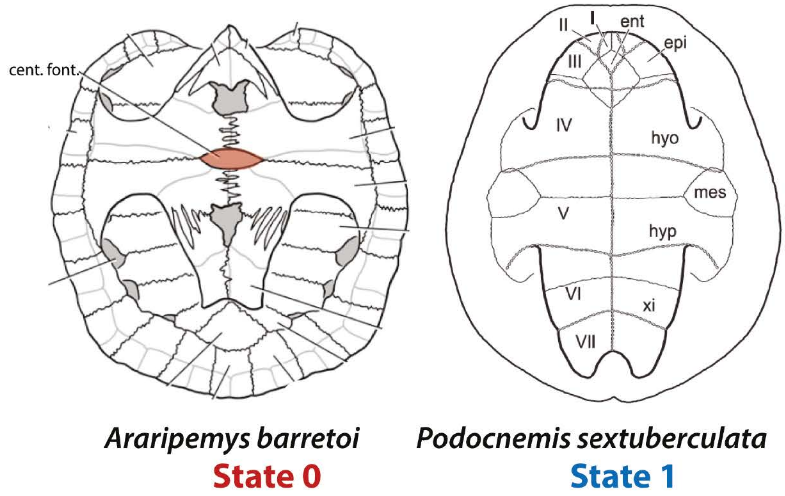

Hyoplastron (central fontanelle)

Character 171

Plate 33. Characters 168 to 171

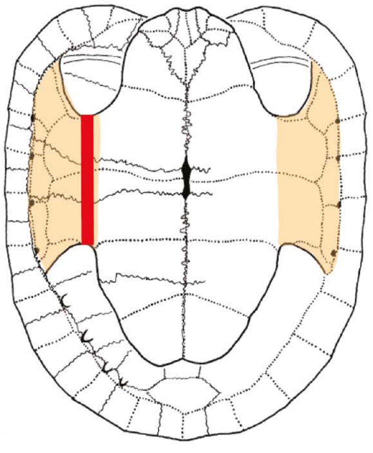

Kayentachelys aprix
State 0

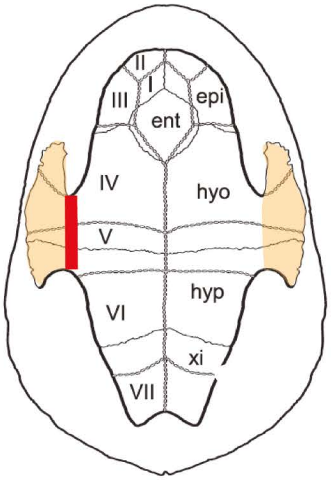

Chelodina oblonga
State 1

Hyoplastron (plastral bridge length)

Character 172

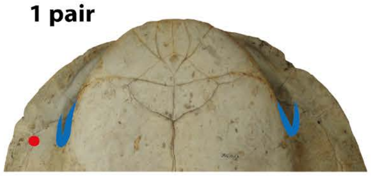

Podocnemis expansa
State 0

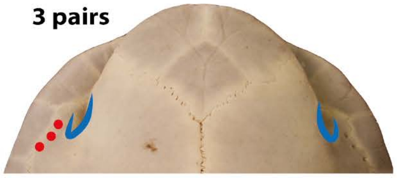

Podocnemis unifilis
State 1

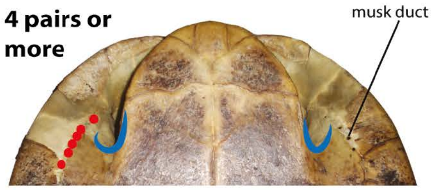

Podocnemis lewyana
State 2

Hyoplastron (number of musk duct)

Character 174

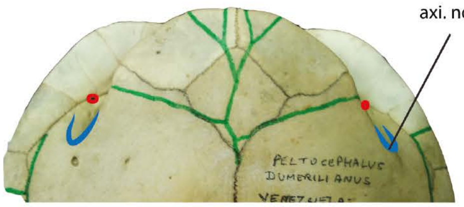

Peltocephalus dumerilianus
State 0

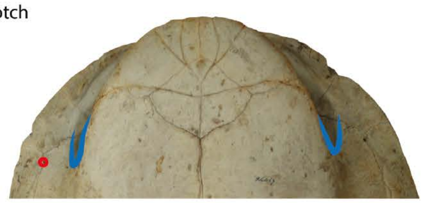

Podocnemis expansa
State 1

Hyoplastron (axillary musk duct location)

Character 173

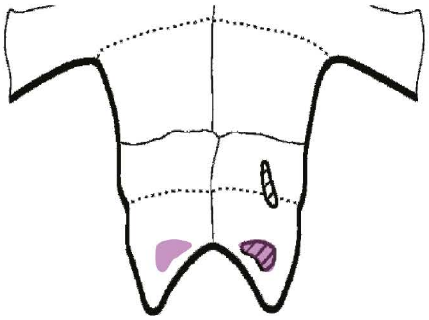

Araiochelys hirayamai
State 0

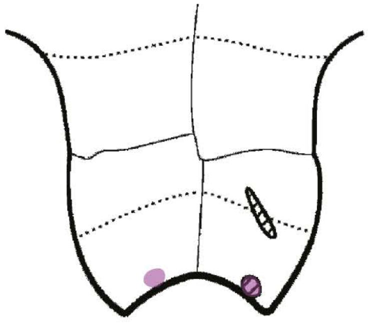

Taphrosphys sulcatus
State 1

Xiphiplastron (ischial scar)

Character 176

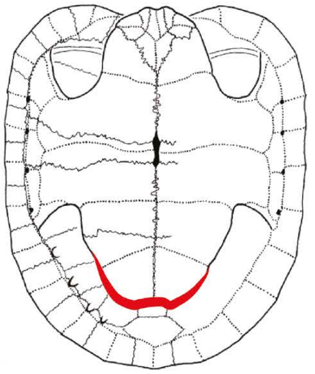

Kayentachelys aprix
State 0

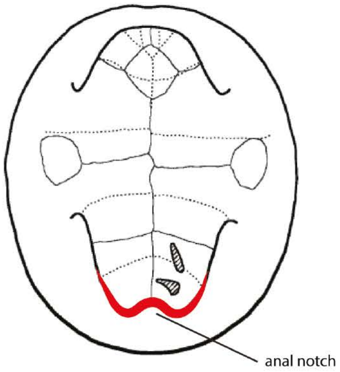

Foxemys mechinorum
State 1

Xiphiplastron (anal notch pres/abs)

Character 175

Plate 34. Characters 172 to 176

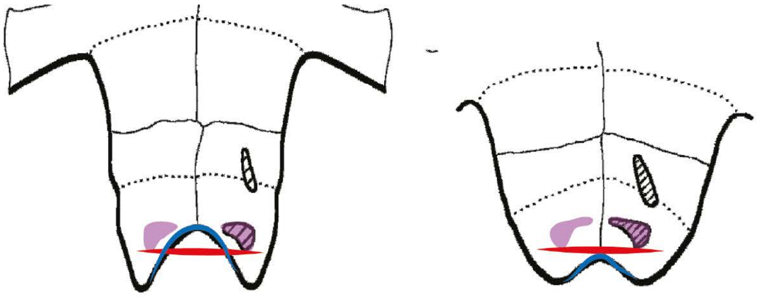

Araiochelys hirayamai
State 0

Foxemys mechinorum
State 1

Xiphiplastron (ischial scar vs anal notch)

Character 177

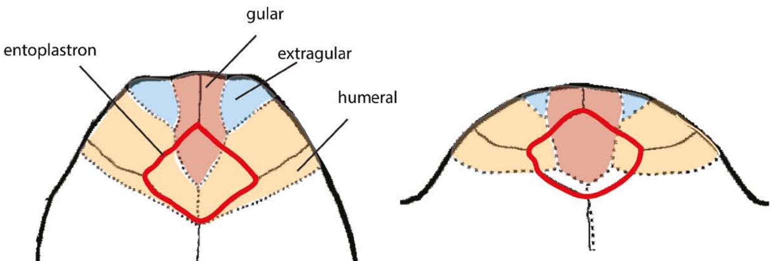

Cearachelys placidoi
State 0

Taphrosphys congolensis
State 1

Humeral (medial contact)

Character 179

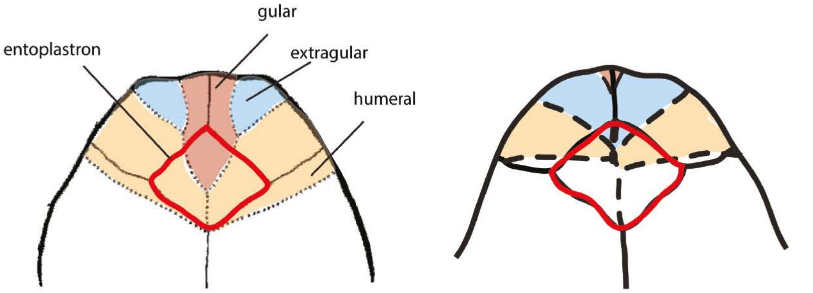

Cearachelys placidoi
State 0

Erymnochelys madagascariensis
State 1

Extragulars (medial contact)

Character 180

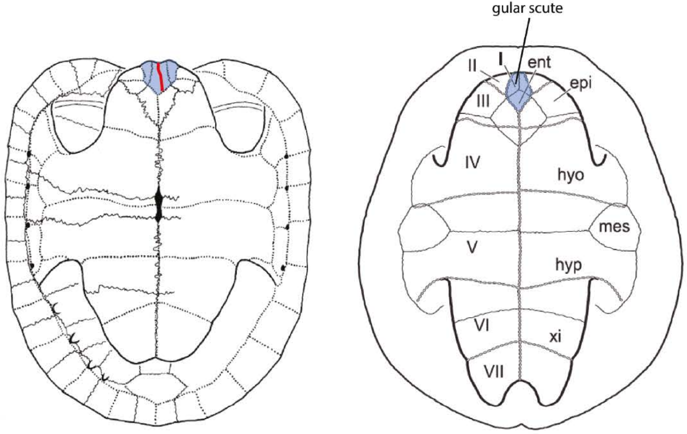

Kayentachelys aprix
State 0

Podocnemis sextuberculata
State 1

Gular (number of gular scute(s))

Character 178

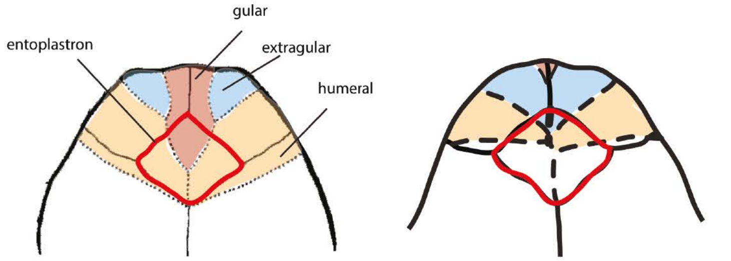

Cearachelys placidoi
State 0

Erymnochelys madagascariensis
State 1

Extragulars (reaching entoplastron)

Character 181

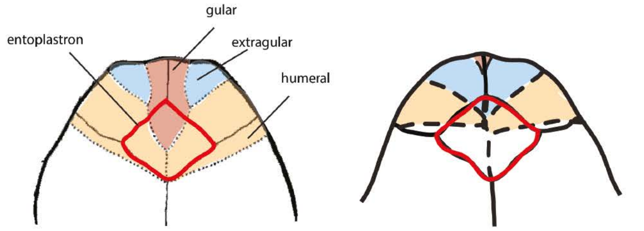

Cearachelys placidoi
State 0

Erymnochelys madagascariensis
State 1

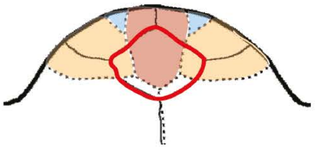

Taphrosphys congolensis
State 2

Extragulars (anterior margin width)

Character 182

Plate 35. Characters 177 to 182

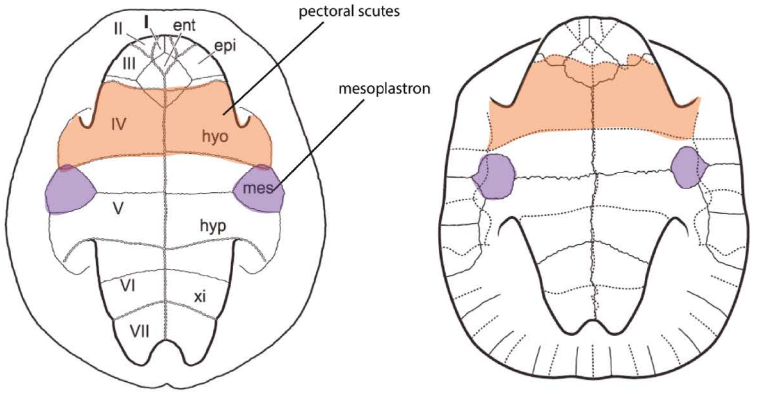

Podocnemis sextuberculata — State 0

Bauremys elegans — State 1

Pectoral (pectoral scute on entoplastron)

Character 183

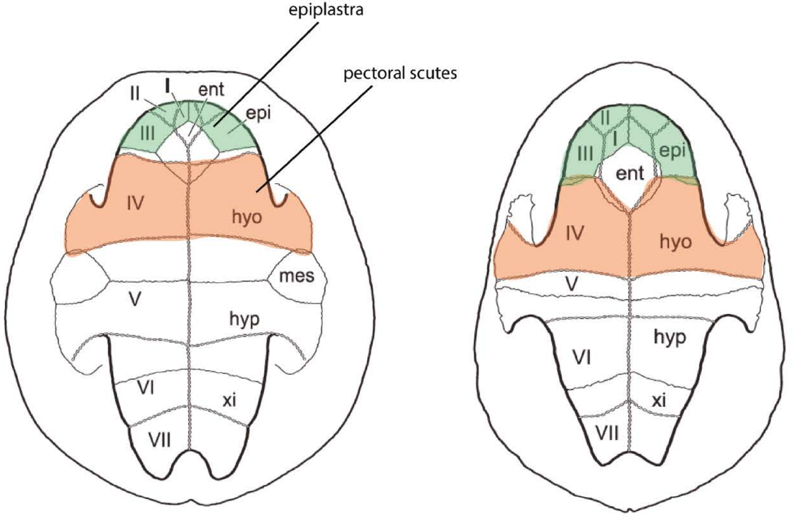

Podocnemis sextuberculata — State 0

Chelodina oblonga — State 1

Pectoral (pectoral scute on epiplastron)

Character 184

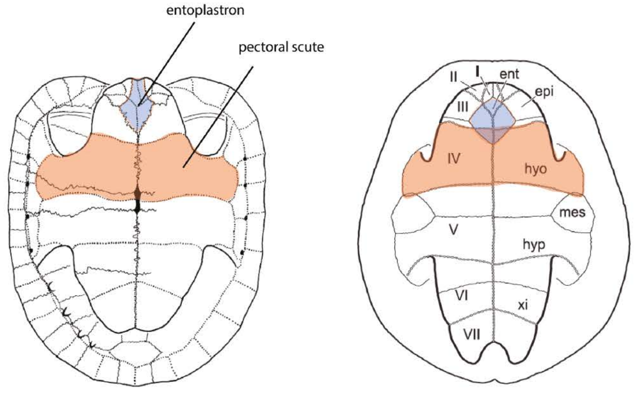

Kayentachelys aprix — State 0

Podocnemis sextuberculata — State 1

Pectoral (pectoral scutes on entoplastron)

Character 185

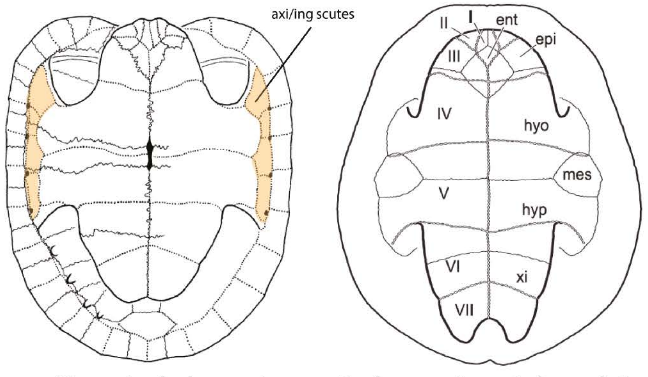

Kayentachelys aprix — State 0

Podocnemis sextuberculata — State 1

Inframarginals (inframarginal scutes)

Character 186

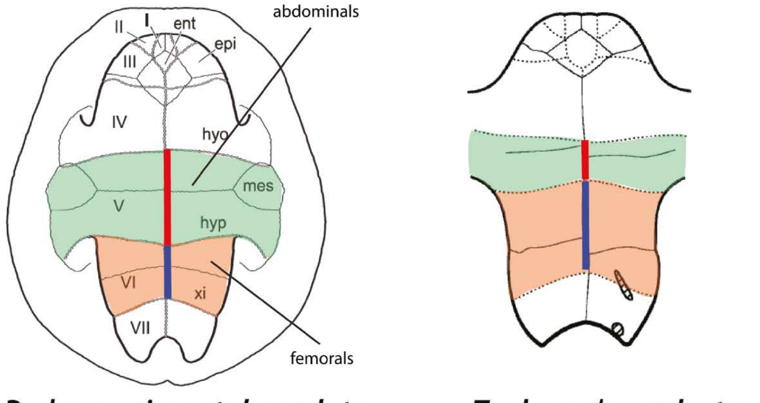

Podocnemis sextuberculata — State 0

Taphrosphys sulcatus — State 1

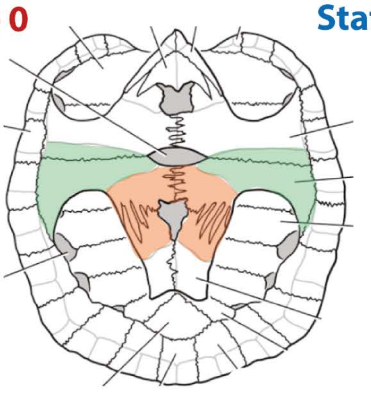

Araripemys barretoi — State 2

Abdominal (length of abd. vs femorals

Character 187

Plate 36. Characters 183 to 187
